# A tissue-specific protein purification approach in *Caenorhabditis elegans* identifies novel interaction partners of DLG-1/Discs large

**DOI:** 10.1186/s12915-016-0286-x

**Published:** 2016-08-09

**Authors:** Selma Waaijers, Javier Muñoz, Christian Berends, João J. Ramalho, Soenita S. Goerdayal, Teck Y. Low, Adja D. Zoumaro-Djayoon, Michael Hoffmann, Thijs Koorman, Roderick P. Tas, Martin Harterink, Stefanie Seelk, Jana Kerver, Casper C. Hoogenraad, Olaf Bossinger, Baris Tursun, Sander van den Heuvel, Albert J. R. Heck, Mike Boxem

**Affiliations:** 1 Developmental Biology, Department of Biology, Faculty of Science, Utrecht University, Padualaan 8, 3584 CH Utrecht, The Netherlands; 2 Biomolecular Mass Spectrometry and Proteomics, Bijvoet Center for Biomolecular Research and Utrecht Institute for Pharmaceutical Sciences, Utrecht University, Padualaan 8, 3584 CH Utrecht, The Netherlands; 3 Netherlands Proteomics Centre, Padualaan 8, 3584 CH Utrecht, The Netherlands; 4 Institut für Wissenschaftliche Medizin, D-40591 Düsseldorf, Germany; 5 Cell Biology, Department of Biology, Faculty of Science, Utrecht University, Padualaan 8, 3584 CH Utrecht, The Netherlands; 6 Berlin Institute for Medical Systems Biology (BIMSB), Max Delbrueck Center for Molecular Medicine (MDC) in the Helmholtz Association, Robert-Roessle-Strasse 10, Berlin, 13125 Germany; 7 Molecular Cell Biology, Anatomy I, University of Cologne, D-50937 Cologne, Germany; 8 Present address: Department of Physiology, Radboud University Medical Center, Geert Grooteplein 26, 6525 GA Nijmegen, The Netherlands; 9 Present address: Proteomics Unit, Spanish National Cancer Research Centre (CNIO), ProteoRed-ISCIII, 28029 Madrid, Spain; 10 Present address: Center for Cancer Research and Department of Pathology, Massachusetts General Hospital and Harvard Medical School Department of Pathology, 149 13th Street, 02129 Charlestown, MA USA

**Keywords:** *C. elegans*, Mass spectrometry, Tissue-specific, Protein complex, Affinity purification, Microtubule-associated, Discs large

## Abstract

**Background:**

Affinity purification followed by mass spectrometry (AP/MS) is a widely used approach to identify protein interactions and complexes. In multicellular organisms, the accurate identification of protein complexes by AP/MS is complicated by the potential heterogeneity of complexes in different tissues. Here, we present an in vivo biotinylation-based approach for the tissue-specific purification of protein complexes from *Caenorhabditis elegans*. Tissue-specific biotinylation is achieved by the expression in select tissues of the bacterial biotin ligase BirA, which biotinylates proteins tagged with the Avi peptide.

**Results:**

We generated N- and C-terminal tags combining GFP with the Avi peptide sequence, as well as four BirA driver lines expressing BirA ubiquitously and specifically in the seam and hyp7 epidermal cells, intestine, or neurons. We validated the ability of our approach to identify bona fide protein interactions by identifying the known LGL-1 interaction partners PAR-6 and PKC-3. Purification of the Discs large protein DLG-1 identified several candidate interaction partners, including the AAA-type ATPase ATAD-3 and the uncharacterized protein MAPH-1.1. We have identified the domains that mediate the DLG-1/ATAD-3 interaction, and show that this interaction contributes to *C. elegans* development. MAPH-1.1 co-purified specifically with DLG-1 purified from neurons, and shared limited homology with the microtubule-associated protein MAP1A, a known neuronal interaction partner of mammalian DLG4/PSD95. A CRISPR/Cas9-engineered GFP::MAPH-1.1 fusion was broadly expressed and co-localized with microtubules.

**Conclusions:**

The method we present here is able to purify protein complexes from specific tissues. We uncovered a series of DLG-1 interactors, and conclude that ATAD-3 is a biologically relevant interaction partner of DLG-1. Finally, we conclude that MAPH-1.1 is a microtubule-associated protein of the MAP1 family and a candidate neuron-specific interaction partner of DLG-1.

**Electronic supplementary material:**

The online version of this article (doi:10.1186/s12915-016-0286-x) contains supplementary material, which is available to authorized users.

## Background

Physical interactions between proteins are essential for most, if not all, cellular processes. Hence, many small-scale studies focus on finding binding partners of individual proteins, and much effort has been put into identifying protein interactions on a large scale. One of the most commonly used techniques to identify protein complex components is affinity purification followed by mass spectrometry (AP/MS). Insight into protein complex composition obtained by AP/MS has already had a major impact on our understanding of cellular processes and signal transduction pathways [1]. Systematic AP/MS efforts have mostly focused on single-cell systems, such as yeast, bacteria, and cultured *Drosophila melanogaster* cells [2–8]. In multicellular organisms, the composition and function of protein complexes containing a particular protein of interest may differ between cell types or tissues [9]. Purifying a protein from whole-animal lysates will result in the identification of members of all complexes, which complicates the interpretation of the biological meaning of the identified interactors. One approach to overcome this limitation is to purify specific tissues or cell types. However, the purification of specific cell types from model organisms is challenging at the scale needed for AP/MS.

Here, we present a method for the tissue-specific purification of protein complexes from the nematode *Caenorhabditis elegans,* based on cell type-specific biotinylation*. C. elegans* is a widely used multicellular model organism that contains many differentiated cell types and tissues, including epithelia, neurons, and muscle. Several studies have used AP/MS approaches to purify *C. elegans* proteins and identify interaction partners [10–22]. However, tissue-specific AP/MS approaches are not widely used.

To purify proteins from specific *C. elegans* tissues, we adapted a system based on in vivo biotinylation of a protein of interest. In this approach, a protein of interest is tagged with the 15-amino acid Avi-tag, which can be biotinylated in vivo by the BirA biotin ligase from *Escherichia coli* [23, 24]. The biotinylated bait protein and any associated proteins are then purified with streptavidin-coated beads, and their identities are determined by mass spectrometry (MS). We demonstrate the applicability of this approach by showing tissue-specific biotinylation of Avi-tagged GFP, and by identifying the well-known LGL-1/PAR-6/PKC-3 complex in two epithelial tissues. We also identify a novel ubiquitous interaction between the *C. elegans* Discs large protein DLG-1, and the mitochondrial AAA-type ATPase ATAD-3. We confirm this surprising interaction by immunoprecipitation, map the ATAD-3 interaction domain by yeast two-hybrid assays, and confirm that this domain mediates the interaction in vivo*.* Finally, we identify a candidate neuron-specific interaction between DLG-1 and MAPH-1.1, an uncharacterized protein that shares some sequence similarity with mammalian microtubule-associated proteins MAP1A, MAP1B, and MAP1S. We show that MAPH-1.1 is a microtubule-associated protein, and likely represents a *C. elegans* MAP1 family member.

## Results

### A biotinylation-based tissue-specific protein purification approach

**Fig. 1:**
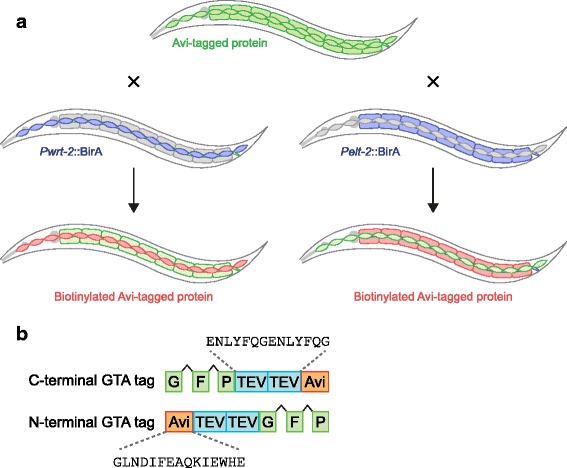
Principles of the approach. **a** By crossing a transgenic *C. elegans* strain expressing the Avi-tagged gene of interest (*green*) with different BirA driver lines (*blue*), biotinylation of the tagged protein (*red*) is accomplished in distinct tissues. **b** Schematic drawing of the N- and C-terminal tags designed. Tags contain the sequences encoding GFP (with introns), two Tobacco etch virus (*TEV*) cleavage sites, and the Avi-tag sequence

To purify protein from specific tissues, we express Avi-tagged proteins of interest from their native regulatory sequences, while biotinylation in a specific tissue is accomplished by expressing BirA from tissue-specific promoters (Fig. 1a). Expression of the bait protein from its native regulatory sequences has several advantages. First, expression will closely mimic the endogenous expression pattern. Second, functionality of the tagged protein can be tested by crossing the transgenic strain with a strain carrying a mutation in the corresponding gene. This simultaneously creates a strain that does not express the untagged endogenous protein. Finally, only a single transgenic strain needs to be created, which can then be crossed to multiple BirA driver strains to purify the protein of interest from different tissues (Fig. 1a). As a first step, we generated four distinct BirA driver strains expressing *C. elegans* codon-optimized and Myc-tagged BirA ligase under the control of different promoters. Two transgenic strains express BirA in epithelial tissues, one expresses BirA in the intestine from the *elt-2* promoter, and one expresses BirA in the seam and hyp7 epidermal cells from the *wrt-2* promoter (*wrt-2* expresses predominantly in seam cells, with weak expression in hyp7) [25]. In addition, we generated strains expressing BirA ubiquitously (using the *rps-27* promoter), and in neuronal cells (using the *rgef-1* promoter). Reverse transcription (RT)-PCR of transgenic animals expressing BirA from the *rgef-1* promoter showed that the *BirA* transgene was properly transcribed and spliced (Additional file 1: Figure S1).

We designed N- and C-terminal tags consisting of GFP and the Avi sequence, separated by two Tobacco etch virus (TEV) cleavage sites, which we term the GTA tag (for GFP, TEV, Avi) (Fig. 1b, Additional file 2: Figure S2, and Additional file 3: Figure S3). The presence of GFP enables the examination of the expression pattern and subcellular localization of the protein of interest, either to confirm that the tagged protein localizes as expected or to study the localization of proteins whose localization pattern has not yet been (fully) described. Tags combining GFP with affinity purification, including a similar tag incorporating the Avi sequence, have been used successfully in *C. elegans* [10, 19]. The use of TEV cleavage sites is necessary to eliminate biotinylated proteins naturally present in *C. elegans*, which will also bind to streptavidin-coated beads. TEV cleavage releases the bait protein and any associated proteins from the beads, while naturally biotinylated background proteins remain bound. GFP, TEV, and Avi are separated by short flexible linkers of five small amino acids, while GFP is separated from the bait protein by a longer flexible linker of 13 small amino acids (Additional file 1: Figure S1 and Additional file 2: Figure S2).

To add the tags to genes encoding proteins of interest, we used fosmid recombineering, a homologous recombination-based genetic engineering technique in bacteria [26]. This approach enabled us to integrate the tag into large regions of genomic DNA (30–40 kb) that likely contain all the native regulatory sequences of the gene of interest, including the promoter, 3′ UTR, and introns (Fig. 1b).

**Fig. 2:**
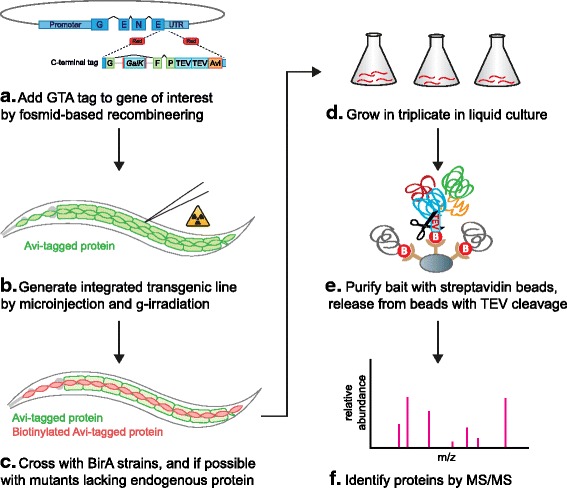
Schematic of the workflow. **a** The GTA tag is added to a gene of interest using recombineering. **b** Transgenic *C. elegans* strains expressing the GTA-tagged protein are generated by injection followed by gamma irradiation-mediated integration of the extrachromosomal array. **c** Transgenic strains are crossed with strains expressing BirA from a tissue-specific promoter, and with a genetic null mutant if appropriate. **d** The transgenic strains are grown in triplicate in liquid culture. **e** Affinity purification is performed on whole-animal lysates. The bait protein with any interacting proteins is subsequently cleaved off the beads by Tobacco etch virus (TEV) protease. **f** The samples are analyzed by tandem mass spectrometry (MS/MS) to identify the proteins they contain

An overview of the entire tagging and AP/MS procedure is shown in Fig. 2. Transgenic strains expressing GTA-tagged proteins are generated by germline injection of engineered fosmids, and the transgenic array is integrated into the genome by gamma irradiation. Transgenic strains expressing the GTA-tagged bait protein are then crossed to BirA-expressing strains. Whenever possible, transgenic strains are also crossed to strains carrying a mutation in the endogenous protein of interest. This allows testing to determine if the tagged protein is fully functional and eliminates the presence of wild-type untagged protein, which would otherwise reduce the fraction of complexes incorporating a tagged protein. The strains are grown at a large scale in liquid culture before harvesting, lysis, and purification of biotinylated proteins with streptavidin-coated beads. The bait protein and any bound proteins are then cleaved off the beads using TEV protease, and analyzed by tandem mass spectrometry (MS/MS) to determine their identities.

### *In vivo* biotinylation and purification are highly tissue-specific

Tissue-specific expression of BirA should result in purification of the bait protein specifically from that tissue. However, at least two potential problems might give rise to biotinylation – and thus purification – of proteins from unintended tissues. First, if the expression of BirA is not tightly limited to the tissue of interest, BirA might be expressed at low levels in other tissues. However, many promoters with well-documented and highly tissue-specific expression patterns have been identified in *C. elegans*. Second, BirA might biotinylate Avi-tagged proteins after the lysis procedure, when proteins from the entire animal are mixed together. The risk of this is low, as BirA activity requires the presence of bivalent ions, which are chelated by the EDTA in the lysis buffer [27]. Nevertheless, we first wished to demonstrate the specificity of biotinylation and purification from a specific tissue.

**Fig. 3:**
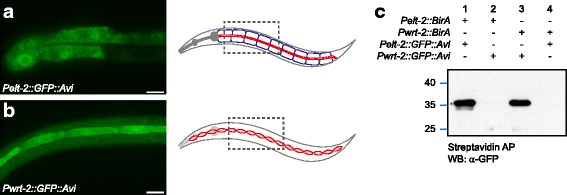
In vivo biotinylation is highly tissue-specific. **a** Expression of GFP::TEV::Avi from the intestinal *elt-2* promoter. **b** Expression of GFP::TEV::Avi from the seam and hyp7 epidermal cell-specific *wrt-2* promoter. In a and b, the tissue and approximate region imaged are indicated in the cartoon worm. **c** Western blot detection of GFP::TEV::Avi purified with streptavidin beads from lysates obtained from animals expressing GFP::TEV::Avi and BirA in the same tissue (lanes 1 and 3) or in different tissues (lanes 2 and 4). Scale bars are 10 μm

To test the tissue specificity of our approach, we generated transgenic *C. elegans* strains expressing cytoplasmic Avi-tagged GFP from the intestinal *elt-2* promoter and the seam and hyp7 epidermal *wrt-2* promoter. Both GTA strains showed expression of cytoplasmic GFP in the expected cells (Fig. 3a, b). We crossed each GTA strain with both epithelial BirA driver strains. Of the four resulting strains, two expressed the biotin ligase and GTA in the same tissue (intestine or epidermal cells), while the other two expressed BirA and GTA in two different tissues. Animals of all four strains were lysed, biotinylated proteins were purified using streptavidin-coated beads, and the presence of GFP was examined by western blot with an antibody directed against GFP. All samples showed the expected purification pattern: when BirA and GTA were present in the same tissue, GTA was purified by the streptavidin beads; when BirA and GTA were expressed in separate tissues, no GTA was purified (Fig. 3c). Thus, in vivo biotinylation and protein purification are highly tissue specific.

### GTA-tagged bait proteins are functional and recapitulate known localization patterns

**Fig. 4:**
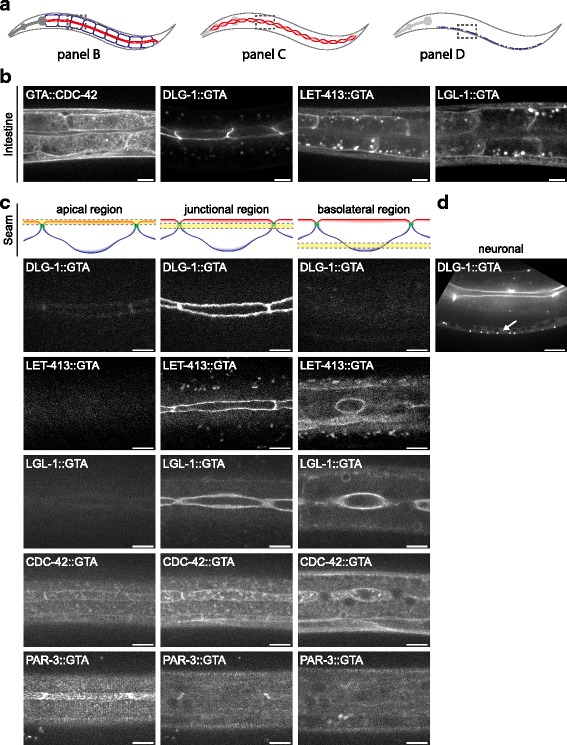
Spinning disc confocal immunofluorescence microscopy images of subcellular localization patterns of GTA-tagged bait proteins. **a** Schematic representation of the areas imaged. **b** Localization in intestinal cells. **c** Localization in seam cells. In both tissues, CDC-42 localizes to cortex, DLG-1 localizes to cell junctions, and LET-413 and LGL-1 localize to basolateral cortex. PAR-3 localizes apically in seam cells, while no PAR-3 expression was detected in intestine. **d** Expression of DLG-1::GTA in ventral cord neurons (e.g., *white arrow*). Scale bars are 10 μm

To test the tissue-specific purification approach, we tagged five polarity regulators that show distinct subcellular localization patterns with the GTA tag: the apical protein PAR-3, the junctional protein DLG-1, the basolateral proteins LET-413 and LGL-1, and the cortical protein CDC-42. These five proteins all contain multiple protein–protein interaction domains and for some of these proteins, interacting partners have already been identified in *C. elegans* or other systems. For example, DLG-1 interacts with the *C. elegans*-specific protein AJM-1, whereas PAR-3 and LGL-1 can both form a complex with PKC-3 and PAR-6, and CDC-42 binds to PAR-6 [28–33]. For PAR-3, DLG-1, LET-413, and LGL-1, we added the GTA tag to the C-terminus, as C-terminal tags are more often compatible with protein function than N-terminal tags [34]. For CDC-42, we used the N-terminal tag, as it has already been shown that *C. elegans* GFP::CDC-42 is able to localize to the cortex, similar to wild-type CDC-42 [28]. For all five genes, we successfully generated integrated transgenic strains using the procedure outlined in Fig. 2. We examined the expression pattern of the tagged bait proteins in the intestine and seam cells and found that all proteins localized to the expected subcellular domains (Fig. 4). In both tissues we observed junctional localization of DLG-1::GTA, basolateral localization of LET-413::GTA and LGL-1::GTA, and cortical localization of GTA::CDC-42. PAR-3::GTA was visible at the apical surface of the seam cells. Unexpectedly, no tagged PAR-3 was detected in the intestinal epithelium. In addition to the epithelial localization, we also observed expression of DLG-1::GTA in ventral cord neurons (Fig. 4d), which is consistent with the expression in neurons of the human homolog DLG4 (PSD-95) [35].

We crossed the *dlg-1*, *lgl-1*, and *let-413* GTA-tagged transgenic strains with strains carrying a mutant allele of the corresponding endogenous gene. *cdc-42* and *par-3* null alleles are early embryonic lethal [36, 37]. Transgenes are usually silenced in the germline and are therefore unlikely to rescue these phenotypes. Consequently, we did not use mutant backgrounds for these two bait proteins.

The *dlg-1::GTA* transgenic strain was crossed with a strain carrying the *dlg-1(ok318)* deletion allele, which causes embryonic arrest at the 2-fold stage. Homozygous *dlg-1(ok318)* mutants carrying the *dlg-1::GTA* transgene were fully viable, demonstrating that the DLG-1::GTA protein is functional. Loss of *lgl-1* does not affect cell polarity or viability, preventing a complementation test of the functionality of the *lgl-1::GTA* transgene [38, 39]. Nevertheless, we crossed our *lgl-1::GTA* transgenic strain with the *lgl-1(tm2616)* deletion allele to prevent incorporation of untagged LGL-1 protein into complexes. For *let-413*, no molecular null allele is available, and the *let-413::GTA* transgenic strain was crossed with a strain carrying the *let-413(s128)* missense mutation, which causes lethality during elongation of the developing embryo [40]. While expression of LET-413::GTA restored embryonic viability, this strain still grew slowly. LET-413 purifications where therefore performed in a *let-413* wild-type background.

In summary, the five tagged proteins localize to the appropriate subcellular domains, and the two transgenes that we could test in complementation assays rescue the lethality caused by the corresponding mutant alleles. Thus, the experimental design appears well suited for the tagging of proteins of interest, without interfering with their function.

### Large-scale culturing and purification of protein complexes

Each of the five transgenic strains were crossed with the *Prps-27::BirA* (ubiquitous)*, Pelt-2::BirA* (intestine), and *Pwrt-2::BirA* (epidermal) BirA driver strains. Because of the observed neuronal localization of DLG-1, the *dlg-1::GTA* strain was also crossed with the pan-neuronal *Prgef-1::BirA* transgenic strain (Table 1). As described above, the *dlg-1::GTA* and *lgl-1::GTA* transgenic strains also carried a putative null mutation in the corresponding endogenous gene. As a negative control to identify common contaminant proteins or proteins that bind to the GTA tag, we generated a transgenic strain expressing cytoplasmic GFP::TEV::Avi from the ubiquitous *rps-27* promoter, which was crossed with each of the four BirA driver strains. In total 20 transgenic strains were generated for affinity purification: strains expressing one of the five tagged bait proteins or the *GFP::TEV::Avi* control construct, combined with BirA expression from one of three different promoters, and in addition, two strains expressing *dlg-1::GTA* or *GFP::TEV::Avi* transgenes combined with BirA expression under the control of the neuronal *rgef-1* promoter.

**Table 1:** Lines generated for purification

BirA driver	GTA-tagged bait	BirA driver	GTA-tagged bait
*Prps-27::BirA*	*par-3::GTA* *dlg-1::GTA* *let-413::GTA* *lgl-1::GTA* *GTA::cdc-42*	*Pwrt-2::BirA*	*par-3::GTA* *dlg-1::GTA* *let-413::GTA* *lgl-1::GTA* *GTA::cdc-42*
*Pelt-2::BirA*	*par-3::GTA* *dlg-1::GTA* *let-413::GTA* *lgl-1::GTA* *GTA::cdc-42*	*Prgef-1::BirA*	*dlg-1::GTA*

Each of the 20 strains was grown in large-scale liquid culture, starting from semi-synchronized L1 cultures generated by starvation on plates. After growth in liquid culture, animals were harvested when most larvae were at the third or fourth larval stage. All strains were grown and analyzed in triplicate to enhance the possibility of separating bona fide protein–protein interactions from nonspecific interactions. All of the harvested cultures were lysed by sonication, and biotinylated proteins were purified using streptavidin-coated beads. Next, bait proteins and binding partners were released from the streptavidin beads through TEV protease cleavage.

**Fig. 5:**
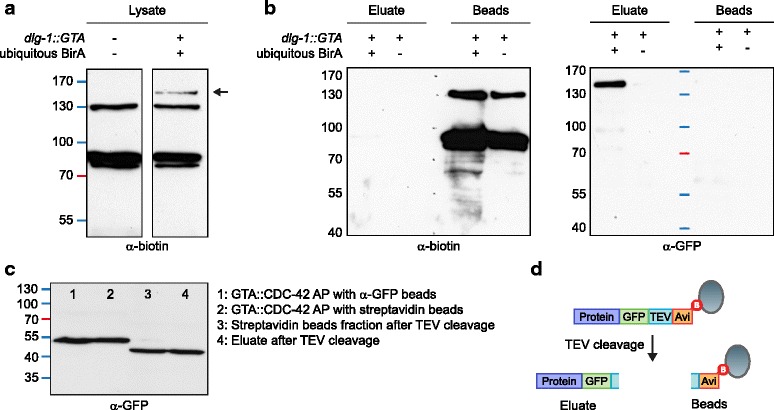
In vivo biotinylation of GTA-tagged protein and removal of endogenously biotinylated proteins by Tobacco etch virus (TEV) cleavage. **a** Western blot detection of biotinylated proteins in lysed wild-type N2 animals (*left lane*) and lysed animals expressing GTA-tagged DLG-1 and ubiquitously expressed BirA (*right lane*). An additional band of the correct estimated molecular weight for DLG-1::GTA is detected upon expression of BirA (*arrow*). **b** Western blot analysis of eluate and beads after purification and release of bound bait protein by TEV cleavage. Both blots contain the same samples. The background of naturally biotinylated proteins remains bound to the streptavidin beads and is visible on the α-biotin western blot in the beads remainder, while tagged DLG-1 is cleaved off and visible on the α-GFP western blot in the eluate. No DLG-1 protein is purified in samples from animals not expressing BirA. **c** *Lanes 1 and 2*: Western blot analysis of the levels of GTA::CDC-42 purified with GFP-Trap beads (*lane 1*) and streptavidin beads (*lane 2*) from animals expressing BirA in seam and hyp7 epidermal cells. *Lanes 3 and 4*: Western blot analysis of beads (*lane 3*) and eluate (*lane 4*) after cleavage of GTA::CDC-42 purified with streptavidin beads. **d** Schematic of the TEV cleavage procedure

To examine the specificity of the biotinylation by BirA, and the efficiency of the purification and subsequent release by TEV cleavage, we analyzed expression and purification of DLG-1::GTA (Fig. 5). Western blot analysis of biotinylated proteins in whole lysates showed a band of the correct molecular weight in samples expressing DLG-1::GTA and ubiquitous BirA, which was absent from the wild-type N2 control (Fig. 5a). No additional biotinylated bands were detected upon expression of BirA, indicating that BirA specifically biotinylates the Avi-tag. Analysis of beads and eluate after purification with streptavidin beads and TEV cleavage showed that DLG-1::GFP was released from the beads, while endogenously biotinylated proteins remained bound (Fig. 5b). We also analyzed the biotinylation efficiency and recovery by TEV cleavage of GTA::CDC-42. We purified GTA::CDC-42 from a strain expressing BirA in the seam and hyp7 epidermal cells using GFP-Trap beads as well as streptavidin beads (Fig. 5c). The equal levels of recovery (compare lanes 1 and 2) indicate efficient biotinylation of GTA::CDC-42. Analysis of beads and eluate after TEV cleavage of GTA::CDC-42 purified with streptavidin beads indicate efficient TEV cleavage. However, approximately half of the CDC-42 remained bound to the beads, despite having been cleaved by TEV (as indicated by the shift in apparent molecular weight). Thus, the overall efficiency of recovery can vary between bait proteins.

### Tissue-specific protein purification recovers known LGL-1 interactors

We next analyzed the samples by high-resolution MS. Each sample was run on a sodium dodecyl sulfate polyacrylamide gel electrophoresis (SDS-PAGE) gel for a length of ~1 cm, followed by in-gel digestion with trypsin and liquid chromatography (LC) MS/MS analysis. Raw data are available via ProteomeXchange with identifier PXD002139. To distinguish genuine interacting proteins from nonspecific binding proteins, we analyzed the data using the SAINT tool, which was developed to assign confidence scores to protein–protein interactions identified in AP/MS experiments [41]. Based on label-free quantification data, and taking into account the number of replicates in which a protein is observed, as well as negative control data, SAINT assigns a probability score to each bait–prey pair identified [41]. Scores range from 0 to 1, and interactions scoring >0.8 can be considered to represent high confidence interactions [41]. We analyzed our samples using SAINT Express embedded within the CRAPome online interface [42]. Spectral counts for the triplicate experiments and negative control samples were uploaded and processed using the default settings. The four tissues analyzed (ubiquitous, epidermal, intestine, neuronal) were processed separately. The results are presented in Additional file 4: Table S1 and Additional file 5: Table S2.

The bait proteins LGL-1, CDC-42, DLG-1, and LET-413 were each themselves identified with high confidence: SAINT probability scores were ≥0.98 in all tissues examined, with the exception of LET-413 in the intestine (score of 0.86). Bait protein PAR-3 was only identified in the ubiquitous sample, with a SAINT score of 0.65. Because PAR-3::GTA was readily detected by microscopy, the protein is likely not efficiently recovered in our purification procedure. PAR-3 samples were therefore excluded from further analysis. The remaining four baits together identified 55 high confidence interactions, which were identified in at least one tissue with a SAINT score >0.8. However, 24 of these were ribosomal proteins or proteins associated with ribosome biogenesis, and most of these were identified with multiple bait proteins (22/24 were found by all four baits). Disregarding interactions with these ribosome-associated proteins, we identified 25 interactions with DLG-1, two interactions with LET-413, and three interactions with LGL-1 (Table 2 and Additional file 6: Table S3). No interactions with a SAINT score >0.8 were identified with CDC-42. However, in an independent experiment, CDC-42 co-purified with the RhoGDI RHI-1, a known interactor of CDC-42 [43] (not shown), which indicates that adjusting purification conditions may yield additional interacting proteins.

**Table 2:** Candidate interactors identified

Bait	Interactor	SAINT confidence scores				Description
		Ubiquitous	Intestine	Seam/hyp7	Neuron	
CDC-42	CDC-42	1.00	1.00	1.00	-	RHO GTPase
DLG-1	VIT-5	0.60	0.00	1.00	0.33	Vitellogenin
DLG-1	UNC-87	1.00	0.54	0.36	0.61	Maintains structure of myofilaments
DLG-1	DLG-1	1.00	1.00	1.00	1.00	Discs large
DLG-1	VIT-6	0.65	0.00	0.99	0.33	Vitellogenin
DLG-1	ATAD-3	0.99	0.46	0.71	0.49	Mitochondrial protein ATAD3
DLG-1	DIM-1	0.22	0.00	0.04	0.98	Uncharacterized
DLG-1	C39H7.4	0.65	-	0.98	0.33	Uncharacterized
DLG-1	COR-1	0.97	0.33	0.33	0.33	Coronin
DLG-1	LEC-1	0.96	0.06	0.84	0.62	Tandem-repeat type galectin
DLG-1	MAPH-1.1	0.00	-	-	0.93	MAP1 homolog
DLG-1	IFB-2	0.66	0.00	0.90	0.93	Intermediate filament protein
DLG-1	F49H12.5	0.56	0.03	0.01	0.93	Thioredoxin domain containing 12
DLG-1	LARP-1	0.00	0.00	0.21	0.92	La-related protein
DLG-1	IFC-2	0.00	0.00	0.91	0.33	Intermediate filament protein
DLG-1	LEC-2	0.90	0.00	0.91	-	Tandem-repeat type galectin
DLG-1	R09H10.5	0.89	0.25	0.91	0.32	Uncharacterized
DLG-1	MUP-2	0.90	0.63	0.28	0.66	Muscle contractile protein troponin T
DLG-1	ATN-1	-	0.31	0.90	-	Alpha-actinin homolog
DLG-1	GPD-2	0.83	0.01	0.89	0.39	GAPDH
DLG-1	QARS-1	0.00	0.00	0.88	0.39	Glutaminyl (Q) tRNA synthetase
DLG-1	VARS-2	0.00	-	0.00	0.88	Valyl-tRNA synthetase
DLG-1	NMT-1	0.00	-	0.58	0.87	N-myristoyl transferase
DLG-1	MRG-1	0.00	0.00	0.12	0.87	Homolog of MRG15
DLG-1	EPS-8	0.85	0.05	0.82	0.66	Cell signaling adaptor protein
DLG-1	T25F10.6	0.83	0.64	0.22	0.33	Calponin
DLG-1	LEC-4	0.65	-	0.81	0.33	Tandem-repeat type galectin
LET-413	LET-413	1.00	0.86	1.00	-	Scribble
LET-413	B0303.3	0.82	0.00	-	-	Beta-ketothiolase
LET-413	QARS-1	0.82	0.01	-	-	Glutaminyl (Q) tRNA synthetase
LGL-1	LGL-1	1.00	1.00	1.00	-	Lethal giant larvae (Lgl)
LGL-1	PAR-6	0.99	1.00	0.98	-	Par-6
LGL-1	PKC-3	0.98	0.97	0.91	-	Atypical protein kinase C
LGL-1	QARS-1	0.82	0.00	0.00	-	Glutaminyl (Q) tRNA synthetase

Shown are putative interactors identified with a SAINT confidence score >0.8 in at least one of the four tissues examined. Ribosomal proteins are not shown. An expanded copy of this table including spectra counts is shown in Additional file 5: Table S2

The proteins that co-purified with LET-413 appear unlikely to be specifically involved in cell polarity: B0303.3 is a homolog of β-ketothiolase, and QARS-1 is a glutaminyl tRNA synthetase. In contrast, LGL-1 co-purified with PAR-6 and PKC-3. *C. elegans* LGL-1 was previously found to be associated with PAR-6 and PKC-3 in embryos [39], and *Drosophila* and mammalian Lgl form a complex with Par-6 and aPKC [29, 32, 33]. In our experiments, PAR-6 and PKC-3 both co-purified with LGL-1 biotinylated specifically in the intestine or seam and hyp7 epidermal cells, as well as with ubiquitously biotinylated LGL-1. These results demonstrate that our biotinylation-based approach is able to identify biologically relevant interactions from specific tissues with high confidence.

### Identification of a novel interaction between DLG-1 and the AAA ATPase-family protein ATAD-3

**Fig. 6:**
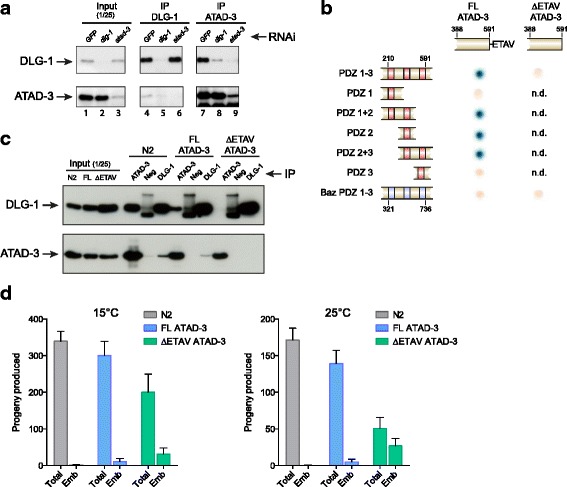
Confirmation of the DLG-1/ATAD-3 interaction and mapping of the interaction domain. **a** Western blots showing co-immunoprecipitation (*IP*) of DGL-1 and ATAD-3. To demonstrate specificity of the detection, samples were treated with control RNAi (GFP RNAi) or RNAi against *dlg-1* or *atad-3. Left panels:* input lysates before IP. *Middle panels:* DLG-1 IP. *Right panels:* ATAD-3 IP. **b** ATAD-3 interacts with DLG-1 in yeast two-hybrid experiments. This interaction depends on the C-terminal ETAV motif of ATAD-3 and the second PDZ domain of DLG-1. Note that ATAD-3 fragments lacking this C-terminal ETAV motif fail to interact with DLG-1 PDZ domains. A fragment containing PDZ 1–3 domains from *Drosophila* Bazooka was used as a negative control. *n.d.* not determined. **c** IP/western blotting experiment shows that, in vivo, the DLG-1/ATAD-3 interaction requires the C-terminal ETAV motif of ATAD-3. Lanes on the *left* show input signals of DLG-1 and ATAD-3. The lanes on the *right* show IPs in corresponding lysates. **d** Progeny produced by N2 animals, or animals expressing full-length ATAD-3 (*ATAD-3* ^*FL*^) and ATAD-3 lacking the C-terminal four amino acids (*ATAD* ^*ΔETAV*^) at 15 °C and 25 °C. Total: average total progeny produced. Emb: average number of embryonic lethal progeny produced. Bars represent average values, and error bars the standard deviation. n = 4

To validate the ability of our approach to identify novel interactors, we focused on the candidate DLG-1 interaction partner ATAD-3, which was identified with high confidence using ubiquitously biotinylated DLG-1::GTA, as well as with lower confidence using BirA expressed in epidermal cells, intestine, and neurons (Table 2). Moreover, ATAD-3 was not identified with any confidence with the other bait proteins (Additional file 5: Table S2). ATAD-3 is an evolutionarily conserved AAA-family ATPase that is essential for mitochondrial activity and development of *C. elegans* [44]. We verified the ubiquitous nature of the DLG-1/ATAD-3 interaction by immunoprecipitating ATAD-3 and DLG-1 from embryonic lysates using specific antibodies. Purification of ATAD-3 revealed co-immunoprecipitation of DLG-1 at a level that was comparable to that observed after DLG-1 immunoprecipitation (Fig. 6a, compare lane 7 and 4). This suggests that a major fraction of DLG-1 interacts with ATAD-3 in the embryo. Conversely, purification of DLG-1 resulted in the co-immunoprecipitation of only a small amount of ATAD-3 (Fig. 6a, lane 4). Purification of ATAD-3 or DLG-1 from lysates of embryos treated with RNAi directed against *dlg-1* or *atad-3* failed to co-immunoprecipitate substantial amounts of DLG-1 or ATAD-3. Thus, the co-immunoprecipitation of DLG-1 is dependent on the presence of ATAD-3, and vice versa. Together, these data indicate that DLG-1 specifically interacts with ATAD-3.

DLG-1 contains three PDZ domains, which are known to function as protein interaction modules. Yeast two-hybrid experiments with fragments of DLG-1 revealed that ATAD-3 binds to the second PDZ domain of DLG-1 (Fig. 6b). PDZ domains frequently bind to short C-terminal motifs in interacting proteins, and the final four amino acids of ATAD-3 (ETAV) match a predicted PDZ domain-binding-specificity class [45]. Indeed, removal of the ETAV sequence rendered ATAD-3 incapable of binding to DLG-1 in the yeast two-hybrid system (Fig. 6b). This indicates that ATAD-3 binds with its C-terminal ETAV motif to the second PDZ domain of DLG-1. To investigate whether the ETAV motif is necessary in vivo to mediate the interaction of ATAD-3 with DLG-1, we generated strains expressing full-length ATAD-3 (ATAD-3^FL^), or ATAD-3 lacking the C-terminal four amino acids (ATAD^ΔETAV^). We inserted single-copy transgenes in chromosome IV by MosSCI [46], and crossed these animals with the *atad-3(ok3093)* strain to obtain animals expressing only the transgene. Both strains express ATAD-3 at levels comparable to wild type (Fig. 6c and Additional file 7: Figure S4). Co-immunoprecipitation experiments showed that ATAD^FL^ interacts with DLG-1 in embryos (Fig. 6c). In contrast, truncation of the C-terminal ETAV motif of ATAD-3 completely prevented the DLG-1 interaction in vivo (Fig. 6c). These data strongly support the presence of a direct physical interaction between ATAD-3 and DLG-1, mediated by the second PDZ domain of DLG-1 and the C-terminal ETAV motif of ATAD-3.

Both the ATAD-3^FL^ and the ATAD^ΔETAV^ strains are viable. However, ATAD^ΔETAV^ animals displayed a dramatically reduced fertility compared to wild type, as well as an increase in embryonic lethality (Fig. 6d). This phenotype was exacerbated at 25 °C compared to 15 °C. These findings indicate that the interaction between ATAD-3 and DLG-1 is physiologically relevant.

### Identification of a *C. elegans* MAP1-family member as a candidate neuron-specific DLG-1 interacting protein

Four of the DLG-1 interactors co-purified exclusively with DLG-1::GTA biotinylated in neurons, and may therefore represent proteins that function with DLG-1 specifically in this tissue. BLAST searches with one of these proteins, F32A7.5, identified the MAP1 family of microtubule-associated proteins (MAPs) as the closest homologs of F32A7.5. Mammalian genomes generally encode three MAP1 proteins: MAP1A, MAP1B, and the shorter family member MAP1S. In *Drosophila*, a single homolog termed Futsch has been described, which shares homology with mammalian MAP1A and MAP1B in the N- and C-terminal regions [47]. Interestingly, MAP1A localizes to postsynaptic densities, and interacts with the DLG-1 homologs DLG1 (SAP-97), DLG2 (PSD-93), and DLG4 (PSD-95) [48, 49]. To date, no *C. elegans* MAP1-family proteins have been described. We hypothesized that F32A7.5 represents a *C. elegans* MAP1-family member, and examined this protein in more detail.

**Fig. 7:**
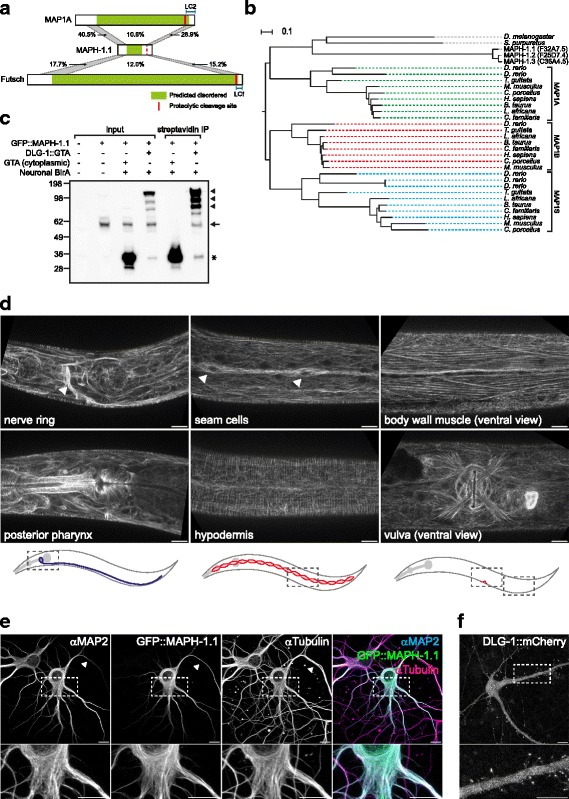
MAPH-1.1 is a microtubule-associated protein related to mammalian MAP1 proteins. **a** Sequence similarity between MAPH-1.1 and human MAP1A, and between MAPH-1.1 and *Drosophila* Futsch. For each protein the predicted disordered region is indicated in *green*. Protein sizes are to scale. For MAP1A and Futsch, a *red line* indicates the proteolytic cleavage site that is used to generate a light chain (LC2 and LCf). A *dotted red line* indicates the homologous position in MAPH-1.1. Sequence similarity is indicated for three regions: two conserved N- and C-terminal regions (indicated in *gray*) and the intervening less conserved region. Amino acid coordinates for these regions are: MAP-1 N-terminal region 274–544, MAP-1 C-terminal region 2847–3041, MAPH-1.1 N-terminal region 1–230, MAPH-1.1 C-terminal region 742–878, Futsch N-terminal region 291–956, and Futsch C-terminal region 5082–5495. **b** Phylogenetic tree of MAP1-related proteins. Color coding indicates groups containing human MAP1A (*green*), MAP1B (*red*), and MAP1S (*blue*). **c** Western blots showing co-immunoprecipitation of DGL-1::GTA and GFP::MAPH-1.1. Input lysates (150 μg protein, lanes 1–4) show expression of DLG-1::GTA (*arrowheads*), GFP::MAPH-1 (*arrow*), and the empty GTA tag (*asterisk*). MAPH-1.1 co-purifies with DLG-1::GTA (lane 6) but not with the GTA tag alone (lane 5). Strains used are N2 (lane 1), BOX188 (lane 2), BOX209 (lanes 3 and 5), BOX212 (lanes 4 and 6). **d** Expression of GFP::MAPH-1 from an endogenously tagged *GFP::maph-1.1* locus. Nerve ring, pharynx, seam cells, and hypodermis panels were taken from the same L4 stage animal. Body wall muscle and vulva panels are ventral views from a second animal. *Arrowheads* indicate the nerve ring an two seam cells. Cartoons are schematic representations of the areas imaged. **e** Expression of an N-terminal GFP::MAPH-1.1 fusion in 6-day-old primary rat hippocampal neuron cultures. Cells were co-stained for MAP2, which localizes to microtubules in dendrites, and for α-tubulin. The *right panel* is a merge with MAP2 in *cyan*, MAPH-1.1 in *green*, and microtubules in *magenta*. The *arrowhead* indicates the axon. **f** Expression of a C-terminal DLG-1::mCherry fusion in 24-day-old primary hippocampal neuron cultures. All scale bars are 10 μm

MAP1A, MAP1B, and Futsch are large proteins (predicted molecular weights 305 kDa, 270 kDa, and 592 kDa, respectively) with no predicted folded domains and extensive predicted unstructured regions. All three proteins are proteolytically cleaved into a heavy chain and a 26–30 kDa light chain derived from the C-terminus [50]. *C. elegans* F32A7.5 is smaller (predicted molecular weight before cleavage 92 kDa), but also contains a large predicted unstructured region. Analogous to Futsch, the similarity of F32A7.5 with the mammalian MAP1 proteins was largely limited to the N- and C-terminus (Fig. 7a). Phylogenetic analysis indicated that F32A7.5 is equally related to MAP1A, MAP1B, and MAP1S (Fig. 7b and Additional file 8: Figure S5). The *C. elegans* genome encodes two other proteins that are highly similar to F32A7.5, termed F25D7.4 and C36A4.5. The high degree of similarity between these proteins (>85 % amino acid identity) indicates that they are paralogs, and in the EggNOG 4.1 database of orthologous groups all three proteins are placed in the same orthologous group as mammalian MAP1A, MAP1B, and MAP1S [51]. Based on phylogeny and the results presented below, we name these proteins MAPH-1.1, MAPH-1.2, and MAPH-1.3, for “microtubule-associated protein 1 homolog 1.”

To examine the expression pattern and subcellular localization of MAPH-1.1, and to validate the interaction with DLG-1, we used CRISPR/Cas9-based genome editing to engineer a GFP-tagged *maph-1* locus (*GFP::maph-1.1*). By western blot, GFP::MAPH-1.1 has an apparent molecular weight of ~60 kDa, much lower than the predicted 119 kDa molecular weight of an unprocessed GFP::MAPH-1.1 fusion (Fig. 7c lane 2). This indicates that, like the mammalian Map1 proteins, MAPH-1.1 is proteolytically cleaved. To confirm the co-affinity purification of MAPH-1.1 with DLG-1, we crossed the *GFP::maph-1.1* strain with a strain expressing DLG-1::GTA from its own promoter and BirA from the neuronal *rgef-1* promoter. As a control, we crossed the *GFP::maph-1.1* strain with a strain ubiquitously expressing cytoplasmic GFP::TEV::Avi, and expressing BirA in neurons. We purified DLG-1::GTA using streptavidin-coated beads, and tested for co-purification of MAPH-1.1 by western blotting using an antibody detecting GFP. We observed co-purification of MAPH-1.1 with DLG-1::GTA, but not with the GFP::TEV::Avi control (Fig. 7c). This confirms the identification of MAPH-1.1 in neuronal DLG-1 purifications by MS.

We observed broad expression of GFP::MAPH-1.1 in multiple somatic postembryonic tissues. This included neurons, but also body wall muscle cells, the vulva, vulval muscle cells, the hypodermis, seam cells, and intestinal cells (Fig. 7d). The fact that we did not identify MAPH-1.1 in DLG-1 purifications from seam or intestinal cells implies that this interaction is either occurring only in neurons, or stabilized in neurons by a neuron-specific protein.

The subcellular localization pattern of GFP::MAPH-1.1 closely resembled that of previously described non-centrosomal microtubule arrays in uterine muscle cells [52], circumferential microtubule arrays in the hypodermis [53], and body wall muscle cells [54] (Fig. 7d). Furthermore, live imaging revealed a highly dynamic behavior of the observed arrays, including shrinkage events (Additional file 9: Movie S1; Additional file 10: Movie S2; Additional file 11: Movie S3; Additional file 12: Movie S4; Additional file 13: Movie S5; and Additional file 14: Movie S6). We measured the shrinkage rate of several arrays in the hypodermis and body wall muscle cells, and obtained an average rate of 0.76 μm/s and 0.85 ± 0.14 μm/s respectively (Additional file 15: Figure S6). These rates are consistent with the previously observed shrinkage rate of 0.85 μm/s for non-centrosomal microtubule bundles in differentiated uterine muscle cells [52]. Finally, we examined the dynamics of GFP::MAPH-1.1 using fluorescence recovery after photobleaching (FRAP). GFP::MAPH-1.1 showed a half-time of recovery of 6 s, which is similar to the rapid dynamics observed for the microtubule-binding domain of ensconsin (MAP7) [55]. All of these observations are consistent with MAPH-1.1 acting as a microtubule-binding protein. Nevertheless, in body wall muscle cells and the hypodermids, tubulin and actin can form bundles that are similar in appearance. Given that mammalian MAP1 proteins can associate with actin [50], we cannot exclude that MAPH-1.1 also localizes to actin structures.

To further confirm the localization of MAPH-1.1 to microtubules, particularly in neurons, we expressed a translational GFP::MAPH-1.1 fusion protein in cultured primary rat hippocampal neurons, in which microtubules are easily visualized. Co-staining with an antibody directed against α-tubulin showed clear co-localization of MAPH-1.1 with microtubules in the neuronal cell body, axon, and dendrites (Fig. 7e). We also expressed a DLG-1:: mCherry fusion protein in cultured hippocampal neurons, and observed localization to synaptic structures in dendrites (Fig. 7f). We conclude that, like mammalian MAP1A, MAPH-1.1 is a microtubule-associated protein, and a candidate neuron-specific interaction partner of DLG-1. In contrast to MAP1A, however, *maph-1.1* is broadly, if not ubiquitously, expressed.

## Discussion

Here, we describe an in vivo biotinylation-based AP/MS approach to purify protein complexes from specific tissues in *C. elegans*. Proteins of interest were expressed with a GTA tag under the control of their normal regulatory sequences, and biotinylation in a single tissue was accomplished by expressing BirA from a tissue-specific promoter. Expression of GFP::TEV::Avi and BirA in two different tissues confirmed that biotinylation and purification are indeed tissue-specific. The ability of our approach to identify bona fide protein interactions from specific tissues was demonstrated by the identification of the known LGL-1 interaction partners PAR-6 and PKC-3 from two distinct epithelial tissues. We also detected a prominent interaction between the junctional scaffold protein DLG-1 and the mitochondrial AAA ATPase ATAD-3. We confirmed this interaction through affinity purification, identified the interacting motif by yeast two-hybrid assays, and demonstrate the requirement for this motif for the ATAD-3/DLG-1 interaction in vivo. Finally, through neuron-specific purification of DLG-1 we identified MAPH-1.1 as an interactor of DLG-1, and demonstrate that this protein represents a *C. elegans* member of the MAP1 family of MAPs.

As is generally the case with interaction detection assays, not all known interactions were recovered. In some cases, this may have been due to the stringency of the SAINT threshold of 0.8, as potentially relevant interactions were observed below this threshold. For example, AJM-1 is a known interaction partner of DLG-1 [31], and co-purified with DLG-1 from neurons with a SAINT score of 0.33. Similarly, in MDCK cells, Scribble has been reported to associate with Lgl2 [56], and in our experiments LGL-1 co-purified with LET-413 from intestine with a SAINT score of 0.33. Neither AJM-1 nor LGL-1 co-purified with other baits, which indicates that these may represent valid and specific interactions. We also observed that not all bait proteins were recovered at equal levels, which may contribute to the lack of recovery of some known interaction partners. A number of different reasons may cause inefficient bait protein recovery. For example, some proteins may be present at low levels, or may not be effectively released in soluble form into the supernatant by the lysis procedure. It is also possible that some proteins are biotinylated less efficiently in vivo, or that the efficiency of TEV cleavage varies. In part, such issues may be overcome by optimization of lysis and purification conditions for specific bait proteins.

An affinity purification tag incorporating GFP and Avi has been used previously in *C. elegans* to purify a protein complex [19]. However, tissue specificity of the biotinylation and purification procedure had not been demonstrated, and no tissue-specific protein interactions had been identified to date. Two other proteomics approaches using BirA-mediated in vivo biotinylation have also been described in *C. elegans*. One study used in vivo biotinylation of the histone H3.3 protein for the purification of chromatin and epigenetic profiling [57]. Another study used biotinylation of the nuclear pore complex component NPP-9 to purify nuclei from specific tissues [58]. Thus, in vivo biotinylation is proving to be a versatile method for tissue-specific proteomics approaches in *C. elegans*.

The GTA tag includes GFP to visualize the subcellular localization of tagged proteins. The expression patterns of the GTA-tagged proteins we observed largely confirmed known localizations, though we failed to observe intestinal localization of PAR-3::GTA. Possibly, the regulatory sequences of PAR-3 extend beyond the region included in the fosmid, or the *par-3* splice variants we tagged are not expressed in the intestine. Two alternative start sites have already been documented to affect the expression pattern of *par-3*, and it is conceivable that different 3′-ends can also affect *par-3* expression [59]. In addition to previously described localization patterns, we observed expression of DLG-1::GTA in ventral cord neurons. Although the localization did not clearly resemble a synaptic pattern, this observation is consistent with the identification of mammalian DLG-1 homologs as postsynaptic density (PSD) proteins.

We identified a novel interaction between the junctional scaffold protein DLG-1 and the evolutionarily conserved mitochondrial AAA ATPase ATAD-3. ATAD-3 and its homologs in other species (ATAD3A, ATAD3B) have been described as mitochondrial proteins with roles in tumor progression [44, 60–63]. Inactivation of *atad-3* by RNAi causes defects in the development and functioning of mitochondria, and results in embryonic lethality and early larval arrest [44]. Immunopurification followed by western blotting indicated that a substantial fraction of DLG-1 associates with ATAD-3, while only a small sub-fraction of ATAD-3 is in complex with DLG-1. ATAD-3 contains a putative transmembrane domain and has been reported to be inserted into the inner membrane of mitochondria, with its C-terminus located in the matrix and the N-terminus facing the cytosol [61, 64, 65]. Given the specific binding of DLG-1 to the C-terminal end of ATAD-3, the DLG-1/ATAD-3 interaction should either occur in the matrix of mitochondria, or involve a subpopulation of ATAD-3 that is not present in the mitochondria. Results from several studies support that mammalian ATAD3A does not reside exclusively in mitochondria [60, 66, 67]. In fact, a substantial number of mitochondrial proteins are dually localized [68]. In particular, proteins that have a less well-defined mitochondrial targeting sequence are more likely to be found at multiple subcellular locations. ATAD-3 has no recognizable mitochondrial targeting sequence and has been found in the plasma-membrane in certain cancer cells [67], and in the endoplasmic reticulum mitochondria-associated membrane (MAM) fraction [60]. These findings indicate the possibility for interactions with ATAD-3 outside of mitochondria. Our finding that expression of ATAD-3^FL^ rescues the *atad-3(ok3093)* mutation to a greater extent than expression of ATAD^ΔETAV^ suggests that the DLG-1/ATAD-3 interaction plays an important role in *C. elegans* development. In epithelial cells, DLG-1 localizes to cell junctions [69], a location that has not been observed for ATAD-3. Although the exact contribution of the interaction with ATAD-3 to DLG-1 remains to be discovered, it is possible that this interaction is of importance before the formation of cell junctions, or affects a non-junctional pool of DLG-1.

MAPs control the organization, stability, and function of the microtubule cytoskeleton [50]. The mammalian MAP1 family of MAPs contains three family members: MAP1A, MAP1B, and the smaller MAP1S [50]. In *Drosophila*, a single MAP1 protein, termed Futsch, has been identified [47]. MAP1A, MAP1B, and *Drosophila* Futsch are predominantly expressed in neurons, and have been shown to play a role in several aspects of neuronal development and functioning [47, 50]. MAP-1A is found in PSDs, where it interacts with the mammalian DLG1 (SAP-97), DLG2 (PSD-93), and DLG4 (PSD-95) [48, 49]. To date, no *C. elegans* MAP1 proteins had been described, and no clear orthologs are present in the genome. Purification of *C. elegans* DLG-1 from neurons resulted in the identification of F32A7.5 as a candidate interaction partner. Based on sequence similarity to mammalian MAP1 proteins, we postulate that F32A7.5 is a *C. elegans* MAP1 protein, which we named MAPH-1.1 for “microtubule-associated protein 1 homolog 1.” MAPH-1.1 did not co-purify with DLG-1 biotinylated in the intestine or epidermal cells, nor with ubiquitously biotinylated DLG-1, despite expression of MAPH-1.1 in many non-neuronal tissues, including seam and intestine. Despite the high SAINT score with which MAPH-1.1 was identified in neuron-specific DLG-1 purifications, the actual number of peptides identified was low. This may reflect that only a minor fraction of DLG-1 and MAPH-1.1 associate in neurons, or that a neuron-specific protein stabilizes the DLG-1/MAPH-1.1 interaction, which would illustrate the value of using a tissue-specific approach. However, further study will be needed to confirm a biological role of this interaction in neurons in vivo*.*

The localization of MAPH-1.1 to dynamic arrays resembling previously published microtubule arrays, the shrinkage parameters of these arrays, and the co-localization with microtubules in cultured rat primary hippocampal neurons all support a function for MAPH-1.1 as a MAP. The *C. elegans* genome encodes two other proteins that are highly similar to F32A7.5, termed F25D7.4, and C36A4.5, which we termed MAPH-1.2 and MAPH-1.3. We did not identify MAPH-1.2 in any of our purifications, but MAPH-1.3 also interacted with DLG-1 specifically in neurons, though at a much lower confidence level (SAINT score 0.33).

MAP1A binds to the guanylate kinase (GK) domain of DLG4 through a short peptide motif [49], which does not appear to be conserved in the *C. elegans* MAPH-1 proteins. However, mutational analysis of the GK binding motif showed that it is highly permissive of changes, and GK binding sites do not appear to be well conserved across different proteins [49]. Thus, it is possible that the mammalian consensus sequence has diverged from the MAPH-1.1 binding site for DLG-1. Mammalian MAP1A and MAP1B, as well as Futsch, are proteolytically cleaved into heavy and light chains [50, 70]. Residues surrounding the cleavage site are poorly conserved between MAP1A/B and Futsch, and we could not identify a candidate cleavage site in MAPH-1.1 by sequence analysis. Thus, it remains to be determined if the *C. elegans* MAPH-1 proteins are proteolytically cleaved. In mammals, MAP1A and MAP1B heavy and light chains assemble into a trimeric complex together with the separately encoded MAP1 light chain 3 (LC3) [50]. The *C. elegans* protein LGG-2, which plays an important role in the generation of autolysosomes, is highly similar to LC3 [71], consistent with conservation of the mechanism of MAP1 functioning in *C. elegans*. However, a role for LGG-2 in neuronal function or microtubule regulation has not been examined.

## Conclusions

The approach we presented here should be widely applicable for the identification of protein complexes for many proteins, provided that the bait protein is soluble after lysis and can be tagged at either the N-terminus or C-terminus without interfering with the function of the protein. In addition, our approach can be used to identify tissue-specific protein modifications of the bait protein, such as phosphorylations, and to reveal differences in protein complex composition over time by using synchronized cultures. Here, we used fosmid-based recombineering to generate the transgenic strains. With the development of CRISPR/Cas9-based genome engineering, it has become possible to endogenously tag any *C. elegans* gene of interest [72]. Thus, genes can now be endogenously tagged with the GTA tag, which eliminates the need for crosses with a null mutant background, and should result in even more reliable expression patterns.

## Methods

### Cloning of GTA tags

To generate the N- and C-terminal GTA tag constructs pMB41 and pMB72, we inserted TEV and Avi sequences obtained as synthetic DNA constructs from GenScript (http://www.genscript.com), and codon-optimized GFP/GalK sequences from vector pBALU1 [26] into vector pUC19 (NEB, http://www.neb.com) by Gibson assembly. Vector maps and sequences are available in Additional file 16. pMB41 and pMB72 are available through Addgene (see “Availability of data and materials” below).

### Recombineering

The recombineering approach we used has been described previously [26]. Briefly, the fosmid to be engineered is transformed into *E. coli* strain SW105, a *galK*-defective strain carrying a heat shock-inducible phage λ Red recombinase, and an arabinose-inducible Flp recombinase. Bacteria carrying the fosmid are transformed with a PCR product consisting of the tag to be introduced, which also carries the wild-type *galK* sequence, flanked on both sides by 50 nucleotides identical to the insertion site. Expression of λ Red induces homologous recombination between the fosmid and the PCR product, and successful recombinants are selected on media with galactose as the only carbon source. Finally, *galK* sequences, which are flanked by FRT sites, are eliminated from the fosmid by expression of the Flp recombinase.

We used the following fosmids and primers to generate the PCR products used as templates for homologous recombination: For tagging *dlg-1* we used the C-terminal tag, fosmid WRM067dB05, and primers rec_dlg-1_C-term_F (5′-actccatcatcagccgtgaatcgcagacgccaatttgggtgccacgtcatggaggaggatctggaggaggaggatctggaggagga) and rec_dlg-1_C-term_R (5′-acatatttcttgaagaaacgattatttgtctaaaaaatatccaatttcatctattcatgccattcaatcttctgagcttcg); for *let-413* the C-terminal tag, fosmid WRM0640dF02, and primers rec_let-413_C-term_F (5′-ggtccccatcgccagtttcgagaacatctgtgagtaggccatgtgagtatggaggaggatctggaggaggaggatctggaggagga) and rec_let-413_C-term_R (5′-gaatgtcaaaaaaaaaacgtctaatgtctagttttcagccaaaatcggcctcattcatgccattcaatcttctgagcttcg); for *lgl-1* the C-terminal tag, fosmid WRM065bB11, and primers rec_lgl-1_C-term_F (5′-gaagtacggtgaatttgaactttcgcggttggagcagtacgcacaagtcaggaggaggatctggaggaggaggatctggaggagga) and rec_lgl-1_C-term_R (5′-aaaattaatatatatcaacaggaaaacgatttttaaaaaaaatgcatctattcatgccattcaatcttctgagcttcg); for *par-3* the C-terminal tag, fosmid WRM064bG02, and primers rec_par-3_C-term_F (5′-gccaataccgtcgcagagatcagggaccgcctcatcgttttccccagtacggaggaggatctggaggaggaggatctggaggagga) and rec_par-3_C-term_R (5′-gattccgtatttttcgcggctgcgtaatataactttgagaaaaaactgacctattcatgccattcaatcttctgagcttcg); and for *cdc-42* the N-terminal tag, fosmid WRM0612bG08, and primers rec_cdc-42_N-term_F (5′-ctataaagacgtaattttaatacttttattcattttttttttcaggcgaaaaaaatgggacttaatgatattttcgaagctcag) and rec_cdc-42_N-term_R (5′-gttttaccgacagctccatctccaacgacgacgcacttgatcgtctgcatacctcctcctccagatcctcctcct). To check whether the recombineering procedure was successful we performed a PCR reaction on the constructs with primers rec_dlg-1_C-term_check_F (5′-aagctcaagcgcagtattcc) and rec_dlg-1_C-term_check_R (5′-tttcttgaattgagaacttggaaa) for *dlg-1*, primers rec_let-413_C-term_check_F (5′-cgattggtattccgattggt) and rec_let-413_C-term_check_R (5′-gccgaacagtaacggagatt) for *let-413*, primers rec_lgl-1_C-term_check_F (5′-gggagttatgtacaggcatctagta) and rec_lgl-1_C-term_check_R (5′-taagccagccgctagcac) for *lgl-1*, primers rec_par-3_C-term_check_F (5′-tatgccgcgaaggagaagta) and rec_par-3_C-term_check_R (5′-ttcgctcagcggaattatc) for *par-3*, and primers rec_cdc-42_N-term_check_F (5′-tcgtttattaaggcgtttaccg) and rec_cdc-42_N-term_check_R (5′-cgatcgtaatcttcctgtcc) for *cdc-42*.

### Cloning the BirA constructs

The *C. elegans* codon-optimized BirA sequence was obtained as a synthetic DNA construct from GenScript (http://www.genscript.com). To generate the BirA expression constructs pMB37 (*Prps-27::BirA*), pMB71 (*Pelt-2::BirA*), and pMB73 (*Pwrt-2::BirA*), the BirA sequence was cloned into vector pPD158.87 (Addgene #1709) using KpnI and EcoRI restriction sites. For vector pBT331 (*Prgef-1∷BirA*), the BirA sequence was cloned into vector backbone pPD95.77 (Addgene plasmid #1495) using KpnI and EcoRI. Next, promoter regions were amplified from *C. elegans* genomic DNA and cloned into appropriate restriction sites. For pMB37, the *rps-27* promoter was amplified using primers Prps-27F (5′-aaa CTGCAGttcaatcggtttttccttgcttgc) and Prps-27R (5′-aaaGGTACCattccacttgttgagcggggctg), and cloned using PstI/KpnI. For pMB71, the *elt-2* promoter was amplified using primers Pelt-2F (5′-aaaCTGCAGtaatttcgaaatgtatgaactccaattc) and Pelt-2R (5′-aaaCCCGGGctataatctattttctagtttc), and cloned using PstI/SmaI. For pMB73, the *wrt-2* promoter was amplified using primers Pwrt-2F (5′-aaaCTGCAGcaggtcgactccacgtaatttc) and Pwrt-2R (5′-aaaCCCGGGGATCCccgagaaacaattggcaggttg), and cloned using PstI/SmaI. For pBT331, the *rgef-1* promoter was amplified using primers Prgef-1F (5′- aaaCTGCAGcgtttccgatacccccttatatc) and Prgef-1R (5′-aaaCCCGGGgatcctttactgctgatcgtcg), and cloned using PstI/SmaI. Vector maps and sequences are available in Additional file 16. The BirA expression constructs are available through Addgene (see “Availability of data and materials” below).

### Cloning the control constructs

To generate expression constructs pMB43 (*Prps-27::GTA*), pMB76 (*Pelt-2::GTA*), and pMB77 (*Pwrt-2::GTA*), we first removed the *GalK* sequences from the GTA tag vector pMB71 by expression of Flp recombinase. The resulting GFP-2xTEV-Avi sequence was then amplified using primers GTA_F (5′-aaaGGTACCggtagaaaaaatgagtaaaggagaagaacttttc) and GTA_R (5′-aaaGCTAGCttattcatgccattcaatcttctgag), and cloned into vector pPD158.87 (Addgene #1709) using KpnI and NheI. Next, promoter regions were amplified from *C. elegans* genomic DNA and cloned into appropriate restriction sites. For pMB43, the *rps-27* promoter was amplified using primers Prps-27F (5′-aaaCTGCAGttcaatcggtttttccttgcttgc) and Prps-27R (5′-aaaGGTACCattccacttgttgagcggggctg), and cloned using PstI/KpnI. For pMB76, the *elt-2* promoter was amplified using primers Pelt-2F (5′-aaaCTGCAGtaatttcgaaatgtatgaactccaattc) and Pelt-2R (5′-aaaCCCGGGctataatctattttctagtttc), and cloned using PstI/SmaI. For pMB77, the *wrt-2* promoter was amplified using primers Pwrt-2F (5′-aaaCTGCAGcaggtcgactccacgtaatttc) and Pwrt-2R (5′-aaaCCCGGGGATCCccgagaaacaattggcaggttg), and cloned using PstI/SmaI. For pMB43, the *let-858* 3′-UTR was replaced with the *tbb-2* 3′-UTR by PCR amplifying the *tbb-2* 3′-UTR with primers tbb-2U_F (5′-aaaGCTAGCatgcaagatcctttcaagc) and tbb-2U_R (5′-aaaGGGCCCtgatccacgatctggaagatttc), and cloning using NheI and ApaI restriction sites. Vector maps and sequences are available in Additional file 16.

### *C. elegans* strains and culture conditions

Unless otherwise indicated, strains were maintained at 15 °C as previously described [73]. Transgenic strains were generated by injecting constructs into the gonad of young adult N2 animals using conventional micro-injection procedures. N2 animals were obtained from the Caenorhabditis Genetics Center. The amounts of each construct injected are indicated for each strain, and were supplemented to a final DNA concentration of 80 ng/μl with PstI digested phage λ DNA. The resulting transgenic strains carrying extrachromosomal arrays were subjected to gamma irradiation to integrate the construct into the *C. elegans* genome. The following strains were generated:

BAT5: *barIs3 [Prgef-1::BirA, Pceh-36::mCherry] X*

BOX20: *mibIs7[Pwrt-2::BirA 10 ng/μl + Pmyo-2::mCherry 2.5 ng/μl]II*

BOX27: *mibIs14[Pelt-2::BirA 10 ng/μl + Pmyo-2::mCherry 2.5 ng/μl]I*

BOX41: *mibIs23[lgl-1::GFP-2xTEV-Avi 10 ng/μl, Pmyo-3::mCherry 5 ng/μl]V*

BOX43: *mibIs25[Avi-2xTEV-GFP::cdc-42 10 ng/μl, Pmyo-3::mCherry 5 ng/μl]X*

BOX51: *mibIs26[par-3::GFP-2xTEV-Avi 10 ng/μl, Pmyo-3::mCherry 5 ng/μl]V*

BOX55: *mibIs30[let-413::GFP-2xTEV-Avi 10 ng/μl, Pmyo-3::mCherry 5 ng/μl]X*

BOX56: *mibIs31[dlg-1::GFP-2xTEV-Avi 10 ng/μl, Pmyo-3::mCherry 5 ng/μl]V*

BOX58: *mibIs33[Prps-27::BirA 10 ng/μl, Pmyo-2::mCherry 2.5 ng/μl]I*

BOX60: *mibIs35 [Prgef-1::BirA 10 ng/μl, Pmyo-2::mCherry 2.5 ng/μl] II*

BOX61: *mibIs36[Pwrt-2::GFP-2xTEV-Avi 10 ng/μl, Prab-3::mCherry 5 ng/μl]X*

BOX62: *mibIs37[Pelt-2::GFP-2xTEV-Avi 10 ng/μl, Prab-3::mCherry 5 ng/μl]X*

BOX65: *mibIs40[Prps-27::GFP-2xTEV-Avi 10 ng/μl, Prab-3::mCherry 5 ng/μl]III*

BOX99: *mibIs14[Pelt-2::BirA 10 ng/μl, Pmyo-2::mCherry 2.5 ng/μl]I; mibIs37[Pelt-2::GFP-2xTEV-Avi 10 ng/μl, Prab-3::mCherry 5 ng/μl]X*

BOX100: *mibIs7[Pwrt-2::BirA 10 ng/μl, Pmyo-2::mCherry 2.5 ng/μl]II; mibIs37[Pelt-2::GFP-2xTEV-Avi 10 ng/μl, Prab-3::mCherry 5 ng/μl]X*

BOX101: *mibIs14[Pelt-2::BirA 10 ng/μl, Pmyo-2::mCherry 2.5 ng/μl]I; mibIs36[Pwrt-2::GFP-2xTEV-Avi 10 ng/μl, Prab-3::mCherry 5 ng/μl]X*

BOX102: *mibIs7[Pwrt-2::BirA 10 ng/μl, Pmyo-2::mCherry 2.5 ng/μl]II; mibIs36[Pwrt-2::GFP-2xTEV-Avi 10 ng/μl, Prab-3::mCherry 5 ng/μl]X*

BOX103: *mibIs33[Prps-27::BirA 10 ng/μl, Pmyo-2::mCherry 2.5 ng/μl]I; mibIs26[par-3::GFP-2xTEV-Avi 10 ng/μl, Pmyo-3::mCherry 5 ng/μl]V*

BOX104: *mibIs7[Pwrt-2::BirA 10 ng/μl, Pmyo-2::mCherry 2.5 ng/μl]II; mibIs26[par-3::GFP-2xTEV-Avi 10 ng/μl, Pmyo-3::mCherry 5 ng/μl]V*

BOX105: *mibIs14[Pelt-2::BirA 10 ng/μl, Pmyo-2::mCherry 2.5 ng/μl]I; mibIs26[par-3::GFP-2xTEV-Avi 10 ng/μl, Pmyo-3::mCherry 5 ng/μl]V*

BOX106: *mibIs33[Prps-27::BirA 10 ng/μl, Pmyo-2::mCherry 2.5 ng/μl]I; mibIs31[dlg-1::GFP-2xTEV-Avi 10 ng/μl, Pmyo-3::mCherry 5 ng/μl]V; dlg-1(ok318)X*

BOX107: *mibIs7[Pwrt-2::BirA 10 ng/μl, Pmyo-2::mCherry 2.5 ng/μl]II; mibIs31[dlg-1::GFP-2xTEV-Avi 10 ng/μl, Pmyo-3::mCherry 5 ng/μl]V; dlg-1(ok318)X*

BOX108: *mibIs14[Pelt-2::BirA 10 ng/μl, Pmyo-2::mCherry 2.5 ng/μl]I; mibIs31[dlg-1::GFP-2xTEV-Avi 10 ng/μl, Pmyo-3::mCherry 5 ng/μl]V; dlg-1(ok318)X*

BOX109: *mibIs35 [Prgef-1::BirA 10 ng/μl, Pmyo-2::mCherry 2.5 ng/μl] II; mibIs31 [dlg-1::GFP-2xTEV-Avi 10 ng/μl, Pmyo-3::mCherry 10 ng/μl] V ; dlg-1(ok318) X*

BOX110: *mibIs33[Prps-27::BirA 10 ng/μl, Pmyo-2::mCherry 2.5 ng/μl]I; mibIs30[let-413::GFP-2xTEV-Avi 10 ng/μl, Pmyo-3::mCherry 5 ng/μl]X*

BOX111: *mibIs7[Pwrt-2::BirA 10 ng/μl, Pmyo-2::mCherry 2.5 ng/μl]II; mibIs30[let-413::GFP-2xTEV-Avi 10 ng/μl, Pmyo-3::mCherry 5 ng/μl]X*

BOX112: *mibIs14[Pelt-2::BirA 10 ng/μl, Pmyo-2::mCherry 2.5 ng/μl]I; mibIs30[let-413::GFP-2xTEV-Avi 10 ng/μl, Pmyo-3::mCherry 5 ng/μl]X*

BOX113: *mibIs33[Prps-27::BirA 10 ng/μl, Pmyo-2::mCherry 2.5 ng/μl]I; mibIs23[lgl-1::GFP-2xTEV-Avi 10 ng/μl, Pmyo-3::mCherry 5 ng/μl]V; lgl-1(tm2616)X*

BOX114: *mibIs7[Pwrt-2::BirA 10 ng/μl, Pmyo-2::mCherry 2.5 ng/μl]II; mibIs23[lgl-1::GFP-2xTEV-Avi 10 ng/μl, Pmyo-3::mCherry 5 ng/μl]V; lgl-1(tm2616)X*

BOX115: *mibIs14[Pelt-2::BirA 10 ng/μl, Pmyo-2::mCherry 2.5 ng/μl]I; mibIs23[lgl-1::GFP-2xTEV-Avi 10 ng/μl, Pmyo-3::mCherry 5 ng/μl]V; lgl-1(tm2616)X*

BOX116: *mibIs40[Prps-27::GFP-2xTEV-Avi 10 ng/μl, Prab-3::mCherry 5 ng/μl]III; mibIs33[Prps-27::BirA 10 ng/μl, Pmyo-2::mCherry 2.5 ng/μl]I*

BOX117: *mibIs7[Pwrt-2::BirA 10 ng/μl, Pmyo-2::mCherry 2.5 ng/μl]II; mibIs40[Prps-27::GFP-2xTEV-Avi 10 ng/μl, Prab-3::mCherry 5 ng/μl]III*

BOX118: *mibIs14[Pelt-2::BirA 10 ng/μl, Pmyo-2::mCherry 2.5 ng/μl]I; mibIs40[Prps-27::GFP-2xTEV-Avi 10 ng/μl, Prab-3::mCherry 5 ng/μl]III*

BOX119: *mibIs35 [Prgef-1::BirA 10 ng/μl, Pmyo-2::mCherry 2.5 ng/μl] II; mibIs40 [Prps-27::GFP-2xTEV-Avi 10 ng/μl, Prab-3::mCherry 5 ng/μl] III*

BOX133: *mibIs7 [Pwrt-2::BirA 10 ng/μl, Pmyo-2::mCherry] II; mibIs25 [cdc-42::GFP-2TEV-Avi 10 ng/μl, Pmyo-3::mCherry 10ngμl] X*

BOX134: *mibIs33 [Prps-27::BirA 10 ng/μl, Pmyo-2::mCherry 2.5 ng/μl] I; mibIs25 [cdc-42::GFP-2TEV-Avi 10 ng/μl, Pmyo-3::mCherry 10 ng/μl] X*

BOX135: *mibIs14 [Pelt-2::BirA 10 ng/μl, Pmyo-2::mCherry 10 ng/μl] I; mibIs25 [cdc-42::GFP-2TEV-Avi 10 ng/μl, Pmyo-3::mCherry 10 ng/μl] X*

BOX188: *maph-1.1(mib12[GFP::maph-1.1]) I*

BOX209: *maph-1.1(mib12[GFP::maph-1.1]) I; mibIs35 [Prgef-1::BirA 10 ng/μl, Pmyo-2::mCherry 2.5 ng/μl] II; mibIs40 [Prps-27::GFP-2xTEV-Avi 10 ng/μl, Prab-3::mCherry 5 ng/μl] III*

BOX212: *maph-1.1(mib12[GFP::maph-1.1]) I; mibIs35 [Prgef-1::BirA 10 ng/μl, Pmyo-2::mCherry 2.5 ng/μl] II; mibIs31 [dlg-1::GFP-2xTEV-Avi 10 ng/μl, Pmyo-3::mCherry 10 ng/μl] V*

SV1311: *atad-3(ok3093) II*; *hels97[Patad-3::ATAD-3, cb unc-119] IV* (referred to as ATAD-3^FL^)

SV1312: *atad-3(ok3093) II*; *hels98*[*atad-3::ATAD-3-ETAV, cb unc-119] IV* (referred to as ATAD-3^ΔETAV^).

### Western blot analysis

Protein samples were separated on 10 % acrylamide gels, and subjected to western blotting on polyvinylidene difluoride membrane (Immobilon-P; Millipore). Blots were blocked with 5 % skim milk in phosphate-buffered saline with Tween (PBST; 7 mM Na_2_HPO_4_, 3 mM NaH_2_PO_4_, 140 mM NaCl, 5 mM KCl, 0.05 % Tween-20) for 1 h at room temperature. For detection of GFP, blots were incubated with rabbit polyclonal anti-GFP (Abcam ab6556, 1:1000) or anti-biotin (Abcam ab1227, 1:1000) in PBST + 5 % skim milk for 1 h at room temperature, washed with PBST three times for 10 min each at room temperature, incubated with anti-rabbit IgG antibody conjugated to horseradish peroxidase (Jackson Immuno Research 111035003, 1:10,000) for 45 min at room temperature, washed with PBST three times for 10 min each at room temperature, and finally washed once with PBS at room temperature for 10 min. Blots were developed using enhanced chemiluminescent western blotting substrate (Bio-Rad Laboratories).

### *C. elegans* liquid culture

Liquid cultures were started with semi-synchronized L1 animals obtained by starvation. Depending on the growth rate of the transgenic strain to be cultured, 20–60 9 cm nematode growth media (NGM) plates with OP50 bacteria were seeded with 15–45 L4 animals per plate. After 6 or 7 days at 20 °C, no bacteria were left on the plates and the plates were covered with starved L1 animals. All animals were washed off the plates and transferred to a 2 l Erlenmeyer flask containing 500 ml S-medium supplemented with penicillin-streptomycin (5000 U/ml, Life Technologies 15070-63) diluted 1:100, and nystatin suspension (10,000 U/ml, Sigma N1638) diluted 1:1000 [74]. A pellet of OP50 *E. coli* bacteria obtained from a 0.5 l overnight culture in lysogeny broth (LB) was added as food source. Animals were allowed to develop until the L3/L4 stage in an incubator at 20 °C shaking at 200 rpm. To harvest the animals, the culture was transferred to 50 ml conical tubes and cooled on ice for 20 min. Animals were then pelleted by centrifugation (all centrifugation steps in this protocol were performed at 400 g for 2 min at 4 °C). After aspirating the supernatant, animals were pooled in a single 50 ml tube, and washed twice in ice-cold M9 lacking MgSO_4_ [74]. After the second wash step, animals were resuspended in 20 ml of ice-cold M9 lacking MgSO_4_, followed by the addition of 20 ml of ice-cold 60 % sucrose in H_2_O. After vigorous mixing of the sucrose/worm mixture, 4 ml of ice-cold M9 lacking MgSO_4_ was gently layered on top, and the worms were centrifuged at 400 g for 2 min at 4 °C. A layer of animals was now visible on top of the sucrose, while contaminants sedimented at the bottom. The sucrose float steps were performed as quickly as possible, as otherwise the layer of animals failed to form properly. To maximize recovery, 30 ml of supernatant was aspirated from the sucrose float, and distributed into four 50 ml tubes that were subsequently filled by addition of room-temperature M9 lacking MgSO_4_. The room temperature M9 allowed the animals to digest OP50 bacteria in their intestine. The four tubes were placed on ice to cool down for 30 min, after which the animals were washed twice in lysis buffer (150 mM NaCl, 20 mM Tris pH 7.8, 5 mM EDTA). During the first wash the animals were again pooled into one tube. After a final wash in lysis buffer supplemented with 1 % Triton X-100, as much lysis buffer as possible was removed, and the *C. elegans* pellet was frozen in liquid nitrogen and stored at −80 °C.

### Lysis

*C. elegans* pellets were lysed by sonication using a Diagenode Bioruptor (http://www.diagenode.com) fitted with a 15 ml tube holder. To each frozen pellet was added 5 ml of lysis buffer (150 mM NaCl, 20 mM Tris pH 7.8, 5 mM EDTA) supplemented with 1 % Triton X-100, 0.5 tablet of protease inhibitor (Roche, 05892791001), and 7 μl of β-mercaptoethanol. Pellets were gently swirled until thawed. Lysates were transferred to 15 ml TPX hard plastic tubes and placed in the Bioruptor filled with ice-water. Samples were lysed nine times for 30 s, with 30 s intervals. After the third and sixth lysis period, lysates were mixed by gently swirling the tube. To remove cellular debris, lysates were distributed to 2 ml Eppendorf tubes, centrifuged at 16,000 g for 15 min at 4 °C, and collected in a fresh conical-bottom 15 ml polypropylene tube. The concentration of protein in the lysates was determined by Bradford assay (Bio-Rad Laboratories) and the lysates were diluted to 1 mg protein/1 ml lysate.

### Affinity purification

Affinity purifications were performed with streptavidin-coated beads (Chromotek, HP57.1). Prior to use, beads were washed twice in lysis buffer (150 mM NaCl, 20 mM Tris pH 7.8, 5 mM EDTA) supplemented with 1 % Triton X-100 in a 1.5 ml Eppendorf tube, pelleting the beads by centrifugation at 7500 g for 30 s. After the final wash, beads were resuspended in lysis buffer, in the original volume. Next, 25 μl of beads were added to 15 ml of lysate (15 mg protein) in a 15 ml conical-bottom polypropylene tube, after which the tubes were rotated at 4 °C for 1.5 h. Following the incubation, beads were pelleted by centrifugation at 3220 g for 5 min at 4 °C, resuspended in 0.5 ml TEV buffer (20 mM Tris pH 8.0, 150 mM NaCl, and 0.3 % NP40), and transferred to an Eppendorf tube. Beads were then washed three times with 500 μl TEV buffer, pelleting the beads by centrifugation at 7500 g for 30 s at 4 °C. Finally, the beads were resuspended in 15 μl TEV buffer and 2 μl of TEV protease (Promega, V6101) was added to the samples. TEV cleavage was performed overnight at 4 °C in a shaking block for Eppendorf tubes, shaking at 800 rpm.

### Mass spectrometry

Protein reduction and alkylation was performed with 10 mM dithiothreitol (56 °C for 1 h) and 50 mM 2-chloro-iodoacetamide (30 min at room temperature in the dark), respectively, after which in-gel digestion was performed with trypsin overnight at 37 °C. Peptides were extracted with 100 % acetonitrile. The samples were analyzed on an LTQ Orbitrap Elite (Thermo Scientific, Bremen) connected to a Proxeon UHPLC system (Thermo Scientific, Odense). The nanoLC was equipped with a 20 mm × 100 μm internal diameter Reprosil C18 trap column and a 400 mm × 50 μm internal diameter Poroshell C18 analytical column (Zorbax, Agilent), all packed in-house. Solvent A consisted of 0.1 M acetic acid (Merck) in deionized water (Milli-Q, Millipore), and solvent B consisted of 0.1 M acetic acid in 80 % acetonitrile (Biosolve). Trapping was performed at a flow of 5 μl/min for 10 min and the fractions were eluted using a flow rate of 150 nl/min (120 min LC method). The mass spectrometer was operated in positive ion mode and in data-dependent mode to automatically switch between MS and MS/MS. The three most intense ions in the survey scan (350–1500 m/z, resolution 60,000, AGC target 1e6) were fragmented with higher energy collisional dissociation (HCD; AGC target 6e4), with the normalized collision energy set to 32 %. The signal threshold for triggering an MS/MS event was set to 500 counts. Charge state screening was enabled, and precursors with unknown charge state or a charge state of 1 were excluded. Dynamic exclusion was enabled (exclusion size list 500, exclusion duration 40 s).

### Mass spectrometry data analysis

Peak lists were generated from the raw data files using Proteome Discoverer version 1.4.1.14 (Thermo Scientific, Bremen). For each affinity purification, one peak list was generated per gel lane. Peak lists were searched against a *C. elegans* database (UniProt, Jan 2014, 25,863 entries) supplemented with frequently observed contaminants using Mascot software version 2.4.01 (Matrix Science, UK). Trypsin was chosen with two missed cleavages allowed. Carbamidomethylation (C) was set as a fixed modification and oxidation (M) was set as variable modification. The searches were performed using a peptide mass tolerance of 50 ppm and a product ion tolerance of 0.05 Da (HCD), followed by data filtering using percolator, resulting in 1 % false discovery rate (FDR). Only ranked 1 peptide spectrum matches with Mascot scores >20 were accepted. The spectral counts for each triplicate of controls and the four tissues were uploaded to the CRAPome online interface version 1.1 [42] for statistical validation.

### Lysis and immunoprecipitations for DLG-1/ATAD-3

Strains were grown in S-medium, either containing HB101 bacteria or bacterial feeding strains targeting *dlg-1*, *atad-3*, or *gfp* (control) to induce RNAi. Embryo pellets were obtained by hypochlorite treatment of adult worms. Embryo pellets were ground two times for 30 s at a frequency of 1500 beats/min using a Mikro-Dismembrator (Sartorius). Ground embryo pellets were lysed in lysis buffer [20 mM Tris-HCl pH 7.8, 250 mM NaCl, 15 % glycerol, 1 % Triton X-100, 0.5 mM EDTA, 1 mM β-mercaptoethanol, 10 mM 1-naphthyl phosphate monosodium salt monohydrate, 50 mM sodium fluoride, 10 mM sodium pyrophosphate decahydrate, 100 μM sodium orthovanadate, and protease inhibitors (Roche complete, Mini, EDTA-free)] for 15 min at 4 °C. The lysate was cleared at 13,000 rpm for 15 min at 4 °C. For immunoprecipitations, 1 mg of total protein was used with either 1 μl mouse anti-PSD95 antibody (Abcam ab2723, RRID AB_303248) non-covalently bound to 5 μl protein G Sepharose beads, 1 μl rabbit anti-ATAD-3 antibody [44] non-covalently bound to 7.5 μl protein A Sepharose beads, 2 μl rabbit anti-GFP antibody (ThermoFisher A-11122, RRID AB_10073917) non-covalently bound to 7.5 μl protein A Sepharose beads (negative control), or 2 μl rabbit anti-eIF4E antibodies [75] non-covalently bound to 7.5 μl protein A Sepharose beads (negative control). Immunoprecipitations were performed for 1 h at 4 °C. Input lysates (1/25) and immunoprecipitations were loaded on gel. Standard procedures were used for SDS-PAGE and western blotting. Mouse anti-PSD95 (1:1000) and rabbit anti-ATAD-3 (1:500) were used for detection. Horseradish peroxidase-conjugated protein A (VWR International) was used at 1:5000 for ATAD-3 probed blots. The signal was revealed with chemiluminesence (Bio-Rad Laboratories). To examine protein levels in ATAD-3^FL^, and ATAD-3^ΔETAV^ strains, 40 L4 staged larvae grown at indicated temperatures were collected and boiled for 5 min in 1 × Laemmli sample buffer. Samples were run on an SDS-PAGE gel, and blotted according to standard procedures. Immunoblots were probed with rabbit anti-ATAD-3 (1:500) and mouse anti-actin (1:1000) (MP Biomedicals).

### Immunoprecipitations for DLG-1/MAPH-1.1

Animals were grown and lysed, and GTA-tagged proteins were purified as described above for the MS experiments. Input lysates (1/100) and immunoprecipitations were loaded on gel. Standard procedures were used for SDS-PAGE and western blotting. For detection of GFP, blots were incubated with rabbit polyclonal anti-GFP (Abcam ab6556, 1:1000) in PBST + 5 % skim milk for 1 h at room temperature, washed with PBST three times for 10 min at room temperature, incubated with anti-rabbit IgG antibody conjugated to horseradish peroxidase (Jackson Immuno Research 111035003, 1:10,000) for 45 min at room temperature, washed with PBST three times for 10 min at room temperature, and finally washed once with PBS at room temperature for 10 min.

### Yeast two-hybrid assays

To generate DB::DLG-1 and DB::BAZ fusions, relative fragments were PCR amplified with primers containing EcoRI and BamHI restriction sites in their tails, and cloned into vector pGBTK7 (Clontech, Palo Alto, CA, USA). The following primers were used: PDZ1_F (5′-agGAATTCgtcttggagaaaggtcac), PDZ1_R (5′-tggtggGGATCCcggagccgatgg), PDZ2_F (5′-tccatcgGAATTCattcatccacc), PDZ2_R (5′-tcccatGGATCCgcggttgtag), PDZ3_F (5′-gactacGAATTCtctcaaatgg), PDZ3_R (5′-atGGATCCctcttgtggtctgtactg), BAZ_PDZ1-3_F (5′-tgGAATTCgagagcaagcgaaaggagccc), and BAZ_PDZ1-3_R (5′-gcGGATCCcaagatcttgcggcctaccagc).

To generate AD∷ATAD-3 aa388-595 and AD::ATAD-3 aa388-591 (ΔETAV) fusions, relative fragments were PCR amplified with primers containing BamHI and XhoI restriction sites in their tails, and cloned into vector pACT2 (Clontech). The following primers were used: ATAD-3_F (5′-tcggcGGATCCcaattcataaag), ATAD-3_R595 (5′-taaacCTCGAGttaaacagcagtttctctcttc), and ATAD-3_R591 (5′-taaacCTCGAGttatctcttcaacgta). DB and AD plasmids were co-transformed into yeast strain Y190 (Clontech) using the LiAc transformation method [76]. For X-gal assays, yeast were grown overnight at 30 °C on a nitrocellulose filter on top of a yeast extract peptone dextrose (YEPD) agar plate. A Whatman filter paper was placed in a petri dish containing 2 ml of Z-buffer (60 mM Na_2_HPO_4_, 40 M NaH_2_PO_4_, 10 M KCl, 1 M MgSO_4_, pH 7), 5.4 μl β-mercaptoethanol, and 33.4 μl X-Gal (stock solution 20 mg/ml in N,N-dimethyl formamide). The nitrocellulose filter with yeast was then fixed in liquid nitrogen for 10 s, thawed at room temperature, and placed on the Whatman filter paper for 30 min at 30 °C.

### Generation of *atad-3* transgenic strains

For MosSCI integration of *atad-3* constructs on chromosome IV, we generated *Patad-3::atad-3::atad-3 3′UTR* and *Patad-3::atad-3ΔETAV::atad-3 3′UTR* constructs*,* and cloned these into the pCFJ1178 vector (a gift from E. Jorgensen, HHMI, University of Utah, USA). For *Patad-3* we used a 1050 bp 5′ region of *atad-3*. As a 3′ UTR we used a 300 bp flanking region of *atad-3. Patad-3::atad-3-ETAV::atad-3 3′UTR* lacked 12 bp at the 3′-end of the coding region. MosSCI integration was performed as previously described [46, 77]. Briefly: the following injection mixture was injected into *unc-119* animals: 10 ng/μl targeting vector, 10 ng/μl pCFJ601 (P*eft-3*:Mos1 transposase), 2.5 ng/μl pCFJ90 (P*myo-2*:mCherry:*unc-54* UTR), and 10 ng/μl pGH8 (P*rab-3*:mCherry:*unc-54* UTR), and wild-type non-fluorescent animals were selected from the F2 progeny.

### Progeny counting and scoring of embryonic lethality

Starting at the L4 stage, individual animals were cultured at 15 °C or 25 °C and transferred to a fresh plate every 24 h. Hatched and unhatched progeny were counted 24 h after removal of the P0. Bars in Fig. 6 represent mean values, and error bars the standard deviation. The progeny of four animals was counted for each genotype and temperature.

### Phylogenetic analysis and protein alignments

To identify proteins related to MAPH-1.1, MAPH-1.2, and MAPH-1.3, we performed an iterative JackHMMER search with each of the three proteins against the Reference Proteomes dataset (three iterations). To generate the phylogenetic tree, we selected MAP1 homologs from a subset of species. These sequences were aligned using the online version of MAFFT with the settings E-INS-i iterative refinement method (http://mafft.cbrc.jp/alignment/server/) [78]. From the aligned sequences, a phylogenetic tree was produced using FastTree 2 with the default settings [79]. The online Interactive Tree of Life tool was used to visualize the phylogenetic tree (http://itol.embl.de/) [80]. To calculate similarity of MAPH-1.1 to human MAP1S and *Drosophila* Futsch, we performed pairwise alignments with MAFFT as described above, and calculated protein similarity using the online Sequence Manipulation Suite (http://www.bioinformatics.org/sms2/ident_sim.html) [81].

### Genome engineering of *GFP::maph-1.1*

To engineer the *GFP::maph-1* locus, we used homology directed repair of a CRISPR/Cas9 induced double strand break (DSB). To increase the efficiency of DSB generation we generated a new subgenomic RNA (sgRNA) expression vector (pJJR50) that contains the A-U flipped and hairpin extended sgRNA sequence described in Chen *et al.* [82], under control of the R07E5.16 U6 promoter [83]. Target sequences are cloned into BbsI digested vector as pairs of annealed oligonucleotides with a 5′-TCTT overhang added to the forward oligo, and a 5′-AAAC overhang added to the reverse oligo. The *maph-1.1* sgRNA target sequence was GCGTGCATCTACGTCTTGGG, and oligos used to insert the sequence into pJJR50 were 5′- tcttGCGTGCATCTACGTCTTGGG and 5′- aaacCCCAAGACGTAGATGCACGC. To generate the repair template, we inserted 450–650 bp sequences flanking the start codon of *maph-1.1* into the self-excising selection cassette vector pDD282 as described [84]. Several mutations were introduced into the sgRNA target site to prevent cutting by Cas9 after repair. The following mixture was injected into 20 N2 adults: 50 ng/ml Peft-3::Cas9 (Addgene #46168) [85], 100 ng/ml sgRNA in pJJR50, 20 ng/ml *maph-1.1* repair template, and 2.5 ng/ml pCFJ90 [46]. Injected animals were placed on individual NGM plates. After 2–3 days at 25 °C, 500 μl of 5 mg/ml hygromycin in water was added to each plate, and healthy non-red-fluorescent Rol animals were selected after 3–5 days. To eliminate the marker cassette, 8–16 L2 animal were heat shocked for 4 h at 34 °C, and non-Rol progeny were selected. We obtained two independent lines, both of which displayed the same expression pattern. Vector maps and sequences are available in Additional file 16.

### Expression in hippocampal neurons and fixation

To express MAPH-1.1 and DLG-1 in hippocampal neurons, we generated constructs consisting of MAPH-1.1 N-terminally tagged with GFP, and DLG-1 C-terminally tagged with mCherry, driven by the βactin promoter. MAPH-1.1 and DLG-1 were PCR amplified from a mixed-stage *C. elegans* cDNA library, and inserted into a vector containing the pβactin promoter and GFP or mCherry [86]. MAPH-1.1 was amplified using primers 5′-aaaGGCGCGCCAatgccggaggaatatatcatg and 5′-tttGCGGCCGCttagagcaaatcgactctggcc, and cloned using AscI and NotI. DLG-1 was amplified using primers 5′-aaaAAGCTTatgtcccacgagtcatcgg and 5′-tttGGTCTCGTCGACttatgacgtggcacccaaattggcg, digested with HindIII and BsaI, and ligated into vector digested with HindIII and SalI. Vector maps and sequences are available in Additional file 16. Primary hippocampal neurons prepared from embryonic day 18 rat brains [86] were transfected at 4 days in vitro (DIV4) for MAPH-1 and microtubule imaging, or DIV22 for DLG-1 imaging, using Lipofectamine 2000 (Life Technologies) and cultured for 2 additional days. For microtubule imaging, cells were first extracted with 0.3 % glutaraldehyde (GA) in PEM80-buffer (80 mM PIPES, 1 mM EGTA, 4 mM MgCl_2_, pH 6.9) for 1 min at 37 °C. Next, fixation was performed in 4 % paraformaldehyde (PFA) for 10 min. Subsequently, cells were washed twice for 5 min in PBS (Lonza BE17-517Q, w/o Ca and Mg) and cells were further permeabilized for 10 min in PBS + 0.2 % Triton-X100. Cells were then washed three times for 5 min in PBS and incubated for 45 min in blocking solution (2 % w/v bovine serum albumin [BSA], 0.2 % w/v gelatin, 10 mM glycine, 50 mM NH_4_Cl in PBS, pH 7.4). Primary antibodies to α-tubulin (Sigma, mouse clone B-5-1-2, 1:800) and MAP2 (Abcam ab5392, chicken, 1:1000) were incubated overnight at 4 °C in blocking solution. Cells were washed three times in PBS and incubated with secondary antibodies anti-Mouse Alexa Fluor568 (Life Technologies, goat, 1:800) and anti-Chicken Alexa Fluor647 (Life Technologies, goat, 1:800) for 1.5 h at room temperature. Finally, cells were washed three times in PBS and mounted in Mowiol 4-88 (Sigma-Aldrich). For imaging of postsynaptic densities, fixation (without prior extraction) was performed in 4 % PFA for 10 min. Subsequently, cells were washed three times for 10 min in PBS (Lonza BE17-517Q, w/o Ca and Mg), and mounted in Mowiol 4-88.

### Microscopy and image processing

Still images of live animals were captured on a spinning disc platform consisting of a Nikon Ti-U inverted microscope with a motorized XY stage and a Piezo Z stage, ×60 and × 100 PLAN APO 1.4 numerical aperture (NA) oil objectives, a Yokogawa CSU-X1 spinning disc unit equipped with a dual dichroic mirror set for laser wavelengths 488 nm and 561 nm, 488 nm and 561 nm solid state 50 mW lasers controlled by an Andor revolution 500 series AOTF Laser modulator and combiner, Semrock 512/23 + 630/91 dual band pass emission filter, Semrock 525/30 single band pass emission filter, Semrock 617/73 single band pass filter, Semrock 4800 long pass filter (500–1200 pass), and an Andor iXON DU-885 monochrome EMCCD+ camera. All components were controlled by MetaMorph Microscopy Automation & Image Analysis Software. Live imaging was performed on a Nikon Eclipse-Ti microscope with a Plan Apo VC, ×60, 1.40 NA oil objective (Nikon). The microscope was equipped with an ASI motorized stage MS-2000-XYZ with Piezo Top Plate and a Perfect Focus System (Nikon), and used MetaMorph 7.8 to control the camera and all motorized parts. Confocal excitation and detection was achieved using a 100 mW Cobolt Calypso laser, Yokogawa spinning disc confocal scanning unit (CSU-X1-A1), a GFP emission filter [ET-GFP (49002); Chroma], and a Photometrics Evolve 512 EMCCD camera at a final magnification of 110 nm per pixel, including an additional magnification introduced by an extra intermediate lens 2.0X (Edmund Optics). For FRAP experiments we used a similar microscope setup equipped with an ILas system (Roper Scientific France/PICT-IBiSA, Institut Curie), with the laser set at 100 % laser power and a Plan Apo × 60 NA 1.40 oil lens. Imaging was performed at 1 or 2 frames per second. Microscopy of fixed samples was performed on a Zeiss LSM700 laser scanning confocal microscope equipped with a × 63 Plan-Apochromat 1.4 NA objective; 405 nm, 488 nm, 555 nm, and 633 nm lasers; and the following emission filters: SP490 (400–490 nm), SP555 (400–555 nm), SP640 (400–640 nm), BP490-555 (490–555 nm), LP560 (560–750 nm), LP640 (640–750 nm), and BP592-662 (592–662 nm). The LSM700 was controlled by the Zen software package. Maximum projections were generated from a series of slices of a Z-stack with ImageJ and processed with Adobe Photoshop CS6 and Adobe Illustrator CS6.

### FRAP analysis

Images were processed and analyzed in ImageJ. For the FRAP analysis, the average gray value of a 100 × 10 pixel region in the FRAP region or a similar non-bleach area was calculated and background subtracted frame-by-frame by subtracting the average intensity of an empty, non-bleached area. FRAP recovery was calculated as the recovery from the first frame after bleach (set to 0) normalized to the average of the five frames before bleach.

### Microtubule depolymerization speed measurement

Microtubule depolymerization speeds were calculated by making kymographs of the acquired movies using the KymoResliceWide plugin in ImageJ and measuring the distance and time of a depolymerization event. Graphs in Additional file 15: Figure S6 show the mean ± standard deviation, as well as individual measurements.

## Additional files

Additional file 1: Figure S1.
*C. elegans* optimized BirA. **a** DNA sequence of BirA, codon optimized for *C. elegans* and including four artificial introns, as well as a Myc tag. **b** Analysis of the expression and splicing of BirA expressed from the neuronal *rgef-1* promoter by RT-PCR. Primer locations relative to the BirA coding sequence are indicated. Each RT-PCR results in a band with the expected size for a properly expressed and spliced BirA cDNA. (PDF 195 kb) Additional file 2: Figure S2. DNA sequence of the C-terminal GTA tag. Relevant regions are highlighted in color. (PDF 88 kb) Additional file 3: Figure S3. DNA sequence of the N-terminal GTA tag. Relevant regions are highlighted in color. (PDF 88 kb) Additional file 4: Table S1. All proteins identified, organized by tissue examined. (XLSX 118 kb) Additional file 5: Table S2. All proteins identified, organized by bait protein. (XLSX 140 kb) Additional file 6: Table S3. Expanded copy of Table 2 in the main text including peptide numbers. (XLSX 14 kb) Additional file 7: Figure S4. ATAD-3^FL^ and ATAD^ΔETAV^ are expressed at similar levels. Western blot analysis showing expression levels of ATAD-3 in ATAD-3^FL^ and ATAD^ΔETAV^ transgenic animals at three different temperatures. (PDF 244 kb) Additional file 8: Figure S5. 
**a** Phylogenetic tree of MAP1-related proteins. Proteins were identified through iterative HMMER searches as implemented in JackHMMER. Three major groups containing the human MAP1A, MAP1B, and MAP1S proteins are color coded. **b** Sequence alignment of MAPH-1.1 with human MAP1A. (PDF 231 kb) Additional file 9: Movie S1. Live imaging of GFP::MAPH-1.1 in body wall muscle cells. (MP4 4269 kb) Additional file 10: Movie S2. Live imaging of GFP::MAPH-1.1 in the hypodermis. (MP4 2275 kb) Additional file 11: Movie S3. Live imaging of GFP::MAPH-1.1 in head region showing nerve ring. (MP4 2266 kb) Additional file 12: Movie S4. Live imaging of GFP::MAPH-1.1 in posterior pharynx bulb. (MP4 2252 kb) Additional file 13: Movie S5. Live imaging of GFP::MAPH-1.1 in seam cells. (MP4 2276 kb) Additional file 14: Movie S6. Example of FRAP analysis in hypodermis. (MP4 3024 kb) Additional file 15: Figure S6: GFP::MAPH-1 dynamics. **a** Fluorescence recovery of GFP::MAPH-1.1 after photobleaching in the hypodermis. Shown is the mean ± SEM of seven measurements. Dotted horizontal line indicates the maximum recovery (average of the final 10 time points, 0.724). Dotted vertical line indicates the time point (6 s) of 50 % recovery of the maximum. See Additional file 14: Movie S6 for an example. **b** Microtubule depolymerization rates in the hypodermis (Hyp) and body wall muscle (BWM). Shown is the mean ± SD, as well as individual measurements. Hyp n = 14, BWM n = 18. (PDF 139 kb) Additional file 16: PDF document with all vector maps and sequences. (PDF 1724 kb)
