# Supplementary material for: A tissue-specific protein purification approach in Caenorhabditis elegans identifies novel interaction partners of DLG-1/Discs large
Source: BMC Biol. 2016 Aug 9;14:66. doi: 10.1186/s12915-016-0286-x (PMC4977824; doi:10.1186/s12915-016-0286-x)

Fig. S1

A

C. elegans optimized BirA

```
   1 ATGGAGCAGA AGCTCATCTC TGAGGAAGAC CTCGGAGGAG AGCAGAAACT CATCTCTGAA
  61 GAGGACCTCA TGAAGGATAA TACTGTTCCA TTGAAGTTGA TTGCTTTGCT TGCAAATGGA
 121 GAATTTCATT CTGGAGAACA ACTTGGAGAG ACGTTGGGAA TGTCGCGTGC TGCAATTAAC
 181 AAGCATATTC AAACAgtaag tttaaacagt tcggtactaa ctaaccatac atatttaaat
 241 tttcagTTGA GAGACTGGGG AGTTGACGTT TTTACGGTTC CAGGAAAGGG ATATTCGCTT
 301 CCAGAGCCAA TTCAGCTCTT GAATGCAAAG CAAATTTTGG GACAGCTTGA TGGTGGTAGT
 361 GTTGCAGTAC TTCCGGTTAT AGATAGTACA AATCAGTACT TGCTTGATCG AATAGGAGAA
 421 TTGAAATCAG GAGATGCTTG CATCGCTGAG TATCAGCAGG CTGGACGAGG ACGAAGAGGA
 481 CGTAAGTGGT TCTgtaagtt taaacatata tatactaact aaccctgatt atttaaattt
 541 tcagCGCCTT TCGGAGCCAA TCTCTATTTG AGTATGTTCT GGCGTCTTGA ACAAGGTCCG
 601 GCCGCTGCTA TCGGACTTTC ATTGGTTATT GGAATTGTGA TGGCAGAAGT TCTCCGAAAA
 661 CTTGGTGCAG ATAAGGTGAG AGTCAAGTGG CCTAACGATC TTTATCTTCA AGATAGAAAA
 721 TTGGCCGGAA TATTGgtaag tttaaacata tatatactaa ctaaccctga ttatttaaat
 781 tttcagGTTG AGCTTACTGG AAAGACGGGA GATGCTGCTC AAATTGTGAT TGGAGCTGGT
 841 ATCAATATGG CAATGAGAAG AGTAGAAGAG TCTGTTGTTA ATCAAGGTTG GATCACACTT
 901 CAAGAGGCAG GAATCAACCT TGATAGAAAT ACGTTGGCCG CCATGCTTAT CCGAGAATTG
 961 CGAGCTGCAC TCGAACTCTT CGAACAAGAA GGTCTTgtaa gtttaaacat atatatacta
1021 actaaccctg attatttaaa ttttcagGCC CCTTACCTCA GTAGATGGGA GAAACTTGAC
1081 AATTTTATCA ATCGACCAGT GAAGTTGATA ATTGGAGATA AGGAAATTTT TGGTATCAGT
1141 CGAGGAATTG ATAAGCAAGG AGCTCTTCTC CTTGAGCAAG ATGGAATCAT TAAACCATGG
1201 ATGGGTGGAG AGATTTCTTT GCGATCTGCT GAAAAGTAA
```

Myc tag
BirA (codon optimized, with introns)

B

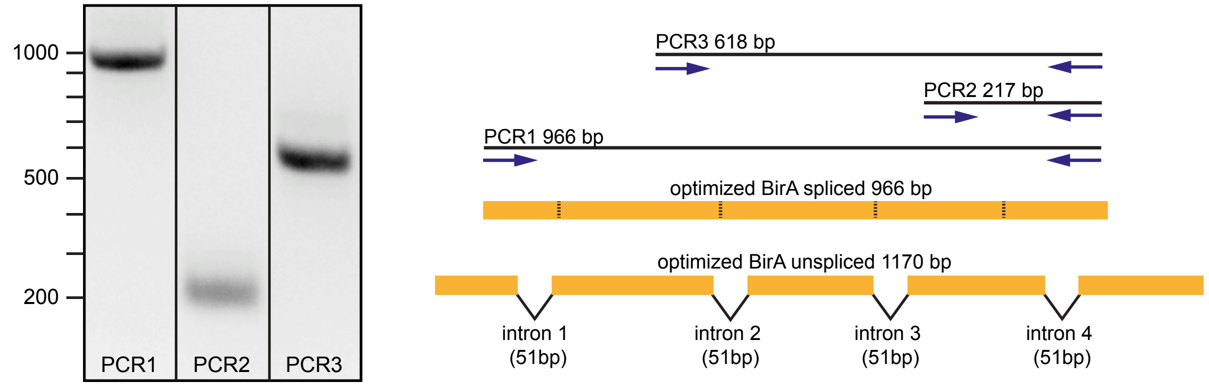

Supplement: Additional file 1: Figure S1. — C. elegans optimized BirA. a DNA sequence of BirA, codon optimized for C. elegans and including four artificial introns, as well as a Myc tag. b Analysis of the expression and splicing of BirA expressed from the neuronal rgef-1 promoter by RT-PCR. Primer locations relative to the BirA coding sequence are indicated. Each RT-PCR results in a band with the expected size for a properly expressed and spliced BirA cDNA. (PDF 195 kb) [file 12915_2016_286_MOESM1_ESM.pdf]
