# Supplementary material for: A tissue-specific protein purification approach in Caenorhabditis elegans identifies novel interaction partners of DLG-1/Discs large
Source: BMC Biol. 2016 Aug 9;14:66. doi: 10.1186/s12915-016-0286-x (PMC4977824; doi:10.1186/s12915-016-0286-x)
Supplement: Additional file 2: Figure S2. — DNA sequence of the C-terminal GTA tag. Relevant regions are highlighted in color. (PDF 88 kb) [file 12915_2016_286_MOESM2_ESM.pdf]

Fig. S2

C-terminal GTA tag

```
   1 GGAGGAGGAT CTGGAGGAGG AGGATCTGGA GGAGGAGGAA TGAGTAAAGG AGAAGAACTT
  61 TTCACTGGAG TTGTCCCAAT TCTTGTTGAA TTAGATGGTG ATGTTAATGG GCACAAATTT
 121 TCTGTCAGTG GAGAGGGTGA AGGTGATGCA ACATACGGAA AACTTACCCT TAAATTTATT
 181 TGCACTACTG GAAAACTACC TGTTCCATGG gtaagtttaa acatatatat actaactaac
 241 cctgattatt taaattttca gCCAACACTT GTCACTACTT TCTGTTATGG TGTTCAATGC
 301 TTCTCGAGAT ACCCAGATCA TATGAAACGG CATGACTTTT TCAAGAGTGC CATGCCCGAA
 361 GGTTATGTAC AGGAAAGAAC TATATTTTTC AAAGATGACG GGAACTACAA GACACgtaag
 421 tttaaacagt tcggtacgaa gttcctatac tttctagaga ataggaactt ccctgttgac
 481 aattaatcat cggcatagta tatcggcata gtataatacg acaaggtgag gaactaaacc
 541 caggaggcag atcatgagtc tgaaagaaaa aacacaatct ctgtttgcca acgcatttgg
 601 ctaccctgcc actcacacca ttcaggcgcc tggccgcgtg aatttgattg gtgaacacac
 661 cgactacaac gacggtttcg ttctgccctg cgcgattgat tatcaaaccg tgatcagttg
 721 tgcaccacgc gatgaccgta aagttcgcgt gatggcagcc gattatgaaa atcagctcga
 781 cgagttttcc ctcgatgcgc ccattgtcgc acatgaaaac tatcaatggg ctaactacgt
 841 tcgtggcgtg gtgaaacatc tgcaactgcg taacaacagc ttcggcggcg tggacatggt
 901 gatcagcggc aatgtgccgc agggtgccgg gttaagttct tccgcttcac tggaagtcgc
 961 ggtcggaacc gtattgcagc agctttatca tctgccgctg gacggcgcac aaatcgcgct
1021 taacggtcag gaagcagaaa accagtttgt aggctgtaac tgcgggatca tggatcagct
1081 aatttccgcg ctcggcaaga aagatcatgc cttgctgatc gattgccgct cactggggac
1141 caaagcagtt tccatgccca aaggtgtggc tgtcgtcatc atcaacagta acttcaaacg
1201 taccctggtt ggcagcgaat acaacacccg tcgtgaacag tgcgaaaccg gtgcgcgttt
1261 cttccagcag ccagccctgc gtgatgtcac cattgaagag ttcaacgctg ttgcgcatga
1321 actggacccg atcgtggcaa aacgcgtgcg tcatatactg actgaaaacg cccgcaccgt
1381 tgaagctgcc agcgcgctgg agcaaggcga cctgaaacgt atgggcgagt tgatggcgga
1441 gtctcatgcc tctatgcgcg atgatttcga aatcaccgtg ccgcaaattg acactctggt
1501 agaaatcgtc aaagctgtga ttggcgacaa aggtggcgta cgcatgaccg gcggcggatt
1561 tggcggctgt atcgtcgcgc tgatcccgga agagctggtg cctgccgtac agcaagctgt
1621 cgctgaacaa tatgaagcaa aaacaggtat taaagagact tttacgttt gtaaaccatc
1681 acaaggagca ggacagtgct gaggatccac tagttctaga gcggccgcga agttcctata
1741 ctttctagag aataggaact tctaactaac catacatatt taaattttca gGTGCTGAAG
1801 TCAAGTTTGA AGGTGATACC CTTGTTAATA GAATCGAGTT AAAAGGTATT GATTTTAAAG
1861 AAGATGGAAA CATTCTTGGA CACAAATTGG AATACAACTA TAACTCACAC AATGTATACA
1921 TCATGGCAGA CAAACAAAAG AATGGAATCA AAGTTgtaag tttaaacatg attttactaa
1981 ctaactaatc tgatttaaat tttcagAACT TCAAAATTAG ACACAACATT GAAGATGGAA
2041 GCGTTCAACT AGCAGACCAT TATCAACAAA ATACTCCAAT TGGCGATGGC CCTGTCCTTT
2101 TACCAGACAA CCATTACCTG TCCACACAAT CTGCCCTTTC GAAAGATCCC AACGAAAAGA
2161 GAGACCACAT GGTCCTTCTT GAGTTTGTAA CAGCTGCTGG GATTACACAT GGCATGGATG
2221 AACTATACAA ATCTGGAGGA GGATCTGAAA ATCTTTATTT CCAAGGATCC GAGAACCTTT
2281 ACTTCCAAGG ATCTGGAGGA GGATCTGGAC TTAATGATAT TTTCGAAGCT CAGAAGATTG
2341 AATGGCATGA ATAA
```

flexible linker
GFP
FRT site
galK sequence
2 TEV sites
Avi tag
stop/start codon
