# Supplementary material for: A tissue-specific protein purification approach in Caenorhabditis elegans identifies novel interaction partners of DLG-1/Discs large
Source: BMC Biol. 2016 Aug 9;14:66. doi: 10.1186/s12915-016-0286-x (PMC4977824; doi:10.1186/s12915-016-0286-x)
Supplement: Additional file 3: Figure S3. — DNA sequence of the N-terminal GTA tag. Relevant regions are highlighted in color. (PDF 88 kb) [file 12915_2016_286_MOESM3_ESM.pdf]

Fig. S3

N-terminal GTA tag

```
   1 aaaaATGGGA CTTAATGATA TTTTCGAAGC TCAGAAGATT GAATGGCATG AATCTGGAGG
  61 AGGATCTGAG AACCTTTACT TCCAAGGAGA GAACCTTTAC TTCCAAGGAT CTGGAGGAGG
 121 ATCTATGAGT AAAGGAGAAG AACTTTTCAC TGGAGTTGTC CCAATTCTTG TTGAATTAGA
 181 TGGTGATGTT AATGGGCACA AATTTTCTGT CAGTGGAGAG GGTGAAGGTG ATGCAACATA
 241 CGGAAAACTT ACCCTTAAAT TTATTTGCAC TACTGGAAAA CTACCTGTTC CATGGgtaag
 301 tttaaacata tatatactaa ctaaccctga ttatttaaat tttcagCCAA CACTTGTCAC
 361 TACTTTCTGT TATGGTGTTC AATGCTTCTC GAGATACCCA GATCATATGA AACGGCATGA
 421 CTTTTTCAAG AGTGCCATGC CCGAAGGTTA TGTACAGGAA AGAACTATAT TTTTCAAAGA
 481 TGACGGGAAC TACAAGACAC gtaagtttaa acagttcggt acgaagttcc tatactttct
 541 agagaatagg aacttccctg ttgacaatta atcatcggca tagtatatcg gcatagtata
 601 atacgacaag gtgaggaact aaacccagga ggcagatcat gagtctgaaa gaaaaaacac
 661 aatctctgtt tgccaacgca tttggctacc ctgccactca caccattcag gcgcctggcc
 721 gcgtgaattt gattggtgaa cacaccgact acaacgacgg tttcgttctg ccctgcgcga
 781 ttgattatca aaccgtgatc agttgtgcac cacgcgatga ccgtaaagtt cgcgtgatgg
 841 cagccgatta tgaaaatcag ctcgacgagt tttccctcga tgcgcccatt gtcgcacatg
 901 aaaactatca atgggctaac tacgttcgtg gcgtggtgaa acatctgcaa ctgcgtaaca
 961 acagcttcgg cggcgtggac atggtgatca gcggcaatgt gccgcagggt gccgggttaa
1021 gttcttccgc ttcactggaa gtcgcggtcg gaaccgtatt gcagcagctt tatcatctgc
1081 cgctggacgg cgcacaaatc gcgcttaacg gtcaggaagc agaaaaccag tttgtaggct
1141 gtaactgcgg gatcatggat cagctaattt ccgcgctcgg caagaaagat catgccttgc
1201 tgatcgattg ccgctcactg gggaccaaag cagtttccat gcccaaaggt gtggctgtcg
1261 tcatcatcaa cagtaacttc aaacgtaccc tggttggcag cgaatacaac acccgtcgtg
1321 aacagtgcga aaccggtgcg cgtttcttcc agcagccagc cctgcgtgat gtcaccattg
1381 aagagttcaa cgctgttgcg catgaactgg acccgatcgt ggcaaaacgc gtgcgtcata
1441 tactgactga aaacgcccgc accgttgaag ctgccagcgc gctggagcaa ggcgacctga
1501 aacgtatggg cgagttgatg gcggagtctc atgcctctat gcgcgatgat ttcgaaatca
1561 ccgtgccgca aattgacact ctggtagaaa tcgtcaaagc tgtgattggc gacaaaggtg
1621 gcgtacgcat gaccggcggc ggatttggcg gctgtatcgt cgcgctgatc ccggaagagc
1681 tggtgcctgc cgtacagcaa gctgtcgctg aacaatatga agcaaaaaca ggtattaaag
1741 agacttttta cgtttgtaaa ccatcacaag gagcaggaca gtgctgagga tccactagtt
1801 ctagagcggc cgcgaagttc ctatactttc tagagaatag gaacttctaa ctaaccatac
1861 atatttaaat tttcagGTGC TGAAGTCAAG TTTGAAGGTG ATACCCTTGT TAATAGAATC
1921 GAGTTAAAAG GTATTGATTT TAAAGAAGAT GGAAACATTC TTGGACACAA ATTGGAATAC
1981 AACTATAACT CACACAATGT ATACATCATG GCAGACAAAC AAAAGAATGG AATCAAAGTT
2041 gtaagtttaa acatgatttt actaactaac taatctgatt taaattttca gAACTTCAAA
2101 ATTAGACACA ACATTGAAGA TGGAAGCGTT CAACTAGCAG ACCATTATCA ACAAAATACT
2161 CCAATTGGCG ATGGCCCTGT CCTTTTACCA GACAACCATT ACCTGTCCAC ACAATCTGCC
2221 CTTTCGAAAG ATCCCAACGA AAAGAGAGAC CACATGGTCC TTCTTGAGTT TGTAACAGCT
2281 GCTGGGATTA CACATGGCAT GGATGAACTA TACAAAGGAG GAGGATCTGG AGGAGGAGGA
2341 TCTGGAGGAG GAGGT
```

flexible linker
GFP
FRT site
galK sequence
2 TEV sites
Avi tag
stop/start codon
