# Supplementary figures and images for: A tissue-specific protein purification approach in Caenorhabditis elegans identifies novel interaction partners of DLG-1/Discs large
Source: BMC Biol. 2016 Aug 9;14:66. doi: 10.1186/s12915-016-0286-x (PMC4977824; doi:10.1186/s12915-016-0286-x)

**Fig. S4**

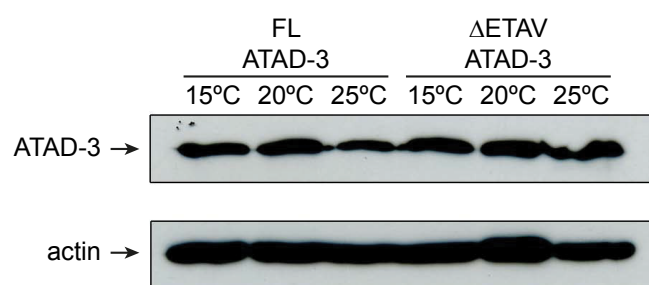

Supplement: Additional file 7: Figure S4. — ATAD-3FL and ATADΔETAV are expressed at similar levels. Western blot analysis showing expression levels of ATAD-3 in ATAD-3FL and ATADΔETAV transgenic animals at three different temperatures. (PDF 244 kb) [file 12915_2016_286_MOESM7_ESM.pdf]

**Fig. S6**

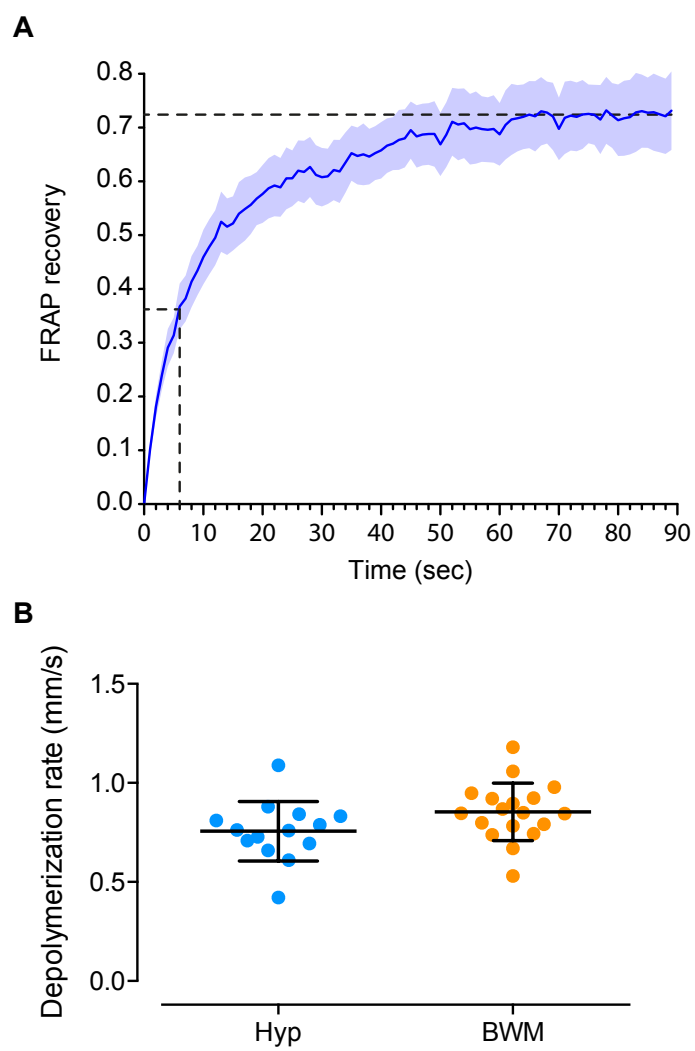

Supplement: Additional file 15: Figure S6: — GFP::MAPH-1 dynamics. a Fluorescence recovery of GFP::MAPH-1.1 after photobleaching in the hypodermis. Shown is the mean ± SEM of seven measurements. Dotted horizontal line indicates the maximum recovery (average of the final 10 time points, 0.724). Dotted vertical line indicates the time point (6 s) of 50 % recovery of the maximum. See Additional file 14: Movie S6 for an example. b Microtubule depolymerization rates in the hypodermis (Hyp) and body wall muscle (BWM). Shown is the mean ± SD, as well as individual measurements. Hyp n = 14, BWM n = 18. (PDF 139 kb) [file 12915_2016_286_MOESM15_ESM.pdf]
