# Supplementary material for: A tissue-specific protein purification approach in Caenorhabditis elegans identifies novel interaction partners of DLG-1/Discs large
Source: BMC Biol. 2016 Aug 9;14:66. doi: 10.1186/s12915-016-0286-x (PMC4977824; doi:10.1186/s12915-016-0286-x)
Supplement: Additional file 8: Figure S5. — a Phylogenetic tree of MAP1-related proteins. Proteins were identified through iterative HMMER searches as implemented in JackHMMER. Three major groups containing the human MAP1A, MAP1B, and MAP1S proteins are color coded. b Sequence alignment of MAPH-1.1 with human MAP1A. (PDF 231 kb) [file 12915_2016_286_MOESM8_ESM.pdf]

Fig. S5

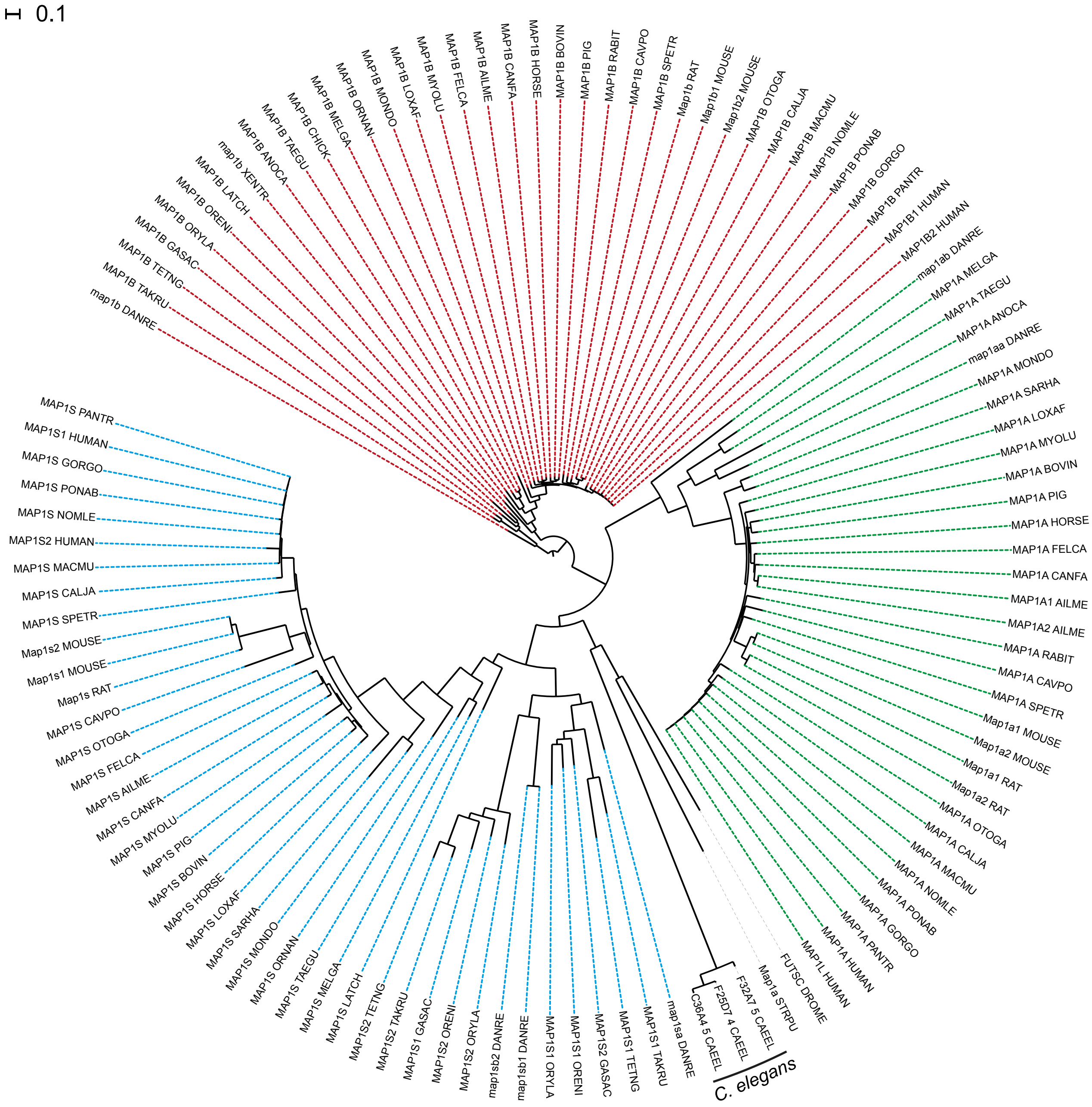
0.1
map1b DANRE
MAP1B TAKRU
MAP1B TETNG
MAP1B GASAC
MAP1B ORYLA
MAP1B ORENI
MAP1B LATCH
map1b XENTR
MAP1B ANOCA
MAP1B TAEGU
MAP1B CHICK
MAP1B MELGA
MAP1B ORNAN
MAP1B MONDO
MAP1B LOXAF
MAP1B MYOLU
MAP1B FELCA
MAP1B AILME
MAP1B CANFA
MAP1B HORSE
MAP1B BOVIN
MAP1B PIG
MAP1B RABIT
MAP1B CAVPO
MAP1B SPETR
Map1b RAT
Map1b1 MOUSE
Map1b2 MOUSE
MAP1B OTOGA
MAP1B CALJA
MAP1B MACMU
MAP1B NOMLE
MAP1B PONAB
MAP1B GORGO
MAP1B PANTR
MAP1B1 HUMAN
MAP1B2 HUMAN
map1ab DANRE
MAP1A MELGA
MAP1A TAEGU
MAP1A ANOCA
map1aa DANRE
MAP1A MONDO
MAP1A SARHA
MAP1A LOXAF
MAP1A MYOLU
MAP1A BOVIN
MAP1A PIG
MAP1A HORSE
MAP1A FELCA
MAP1A CANFA
MAP1A1 AILME
MAP1A2 AILME
MAP1A RABIT
MAP1A CAVPO
MAP1A SPETR
Map1a1 MOUSE
Map1a2 MOUSE
Map1a1 RAT
Map1a2 RAT
MAP1A OTOGA
MAP1A CALJA
MAP1A MACMU
MAP1A NOMLE
MAP1A PONAB
MAP1A GORGO
MAP1A PANTR
MAP1A HUMAN
MAP1L HUMAN
FUTSC DROME
Map1a STRPU
F32A7 5 CAEEL
F25D7 4 CAEEL
C36A4 5 CAEEL
C. elegans
map1sa DANRE
MAP1S1 TAKRU
MAP1S1 TETNG
MAP1S2 GASAC
MAP1S1 ORENI
MAP1S1 ORYLA
map1sb1 DANRE
map1sb2 DANRE
MAP1S2 ORYLA
MAP1S2 ORENI
MAP1S1 GASAC
MAP1S2 TAKRU
MAP1S2 TETNG
MAP1S LATCH
MAP1S MELGA
MAP1S TAEGU
MAP1S ORNAN
MAP1S MONDO
MAP1S SARHA
MAP1S LOXAF
MAP1S HORSE
MAP1S BOVIN
MAP1S PIG
MAP1S MYOLU
MAP1S CANFA
MAP1S AILME
MAP1S FELCA
MAP1S OTOGA
MAP1S CAVPO
Map1s RAT
Map1s1 MOUSE
Map1s2 MOUSE
MAP1S SPETR
MAP1S CALJA
MAP1S MACMU
MAP1S2 HUMAN
MAP1S NOMLE
MAP1S PONAB
MAP1S GORGO
MAP1S1 HUMAN
MAP1S PANTR

```
MAPH-1.1     1 M...........................................................
MAP1A        1 METEAEPARPHGVAMETTPGLGLRSPGAPLAQNPAELLCEAGAAVAAARWDLQKHSLLIV

MAPH-1.1     2 ............................................................
MAP1A       61 IGDIGTESQLRAVRAHLEQGILSWNIDLSSFDLNQQLRLFITRHLAHFSSEVKGQRTLCH

MAPH-1.1     2 ............................................................
MAP1A      121 QSEILETIILVNPSADSISSEVHHLLSSSSAYKLLILSGQSLEPGGDLILQSGTYSYENF

MAPH-1.1     2 ......PE....................................................
MAP1A      181 AQVLHNPEISQLLSNRDPGIQAFLTVSCLGEGDWSHLGLSSSQETLHLRLNPEPTLPTMD

MAPH-1.1     4 ......EYIMS......................SKACIYVLGGAANTAALFDFDGVYILD
MAP1A      241 GVAEFSEYVSETVDVPSPFDLLEPPTSGGFLKLSKPCCYIFPGGRGDSALFAVNGFNILV

MAPH-1.1    36 GGFAEKNPGF...VAHVRDVSAVLLAAPTLGNLGTTSALLEQ..................
MAP1A      301 DGGSDRKSCFWKLVRHLDRIDSVLLTHIGADNLPGINGLLQRKVAELEEEQSQGSSSYSD

MAPH-1.1    75 ......GKPLPVFTNTKPFKTAKPGSSGEIAKAIQEANSKILSV......APPLF....N
MAP1A      361 WVKNLISPELGVVFFNVPEKLRLPDASRKAKRSIEEACLTLQHLNRLGIQAEPLYRVVSN

MAPH-1.1   119 PKYPANIIYQSAAKGVLSLYIL..AGDVKDAEVITKALAG............GNEAEVEK
MAP1A      421 TIEPLTLFHKMGV.GRLDMYVLNPVKDSKEMQFLMQKWAGNSKAKTGIVLPNGKEAEISV

MAPH-1.1   165 AAAEHGTIGVLLWRPAMTDQSVVRVLISGTSSLSRIQQSLDKAAKSLPFLNVPTVKSKDA
MAP1A      480 PYLTSIT.ALVVWLPANPTEKIVRVLFPGNAPQNKILEGLEK.LRHLDFLRYPVATQKDL

MAPH-1.1   225 LSD.IPA.....................................................
MAP1A      538 ASGAVPTNLKPSKIKQRADSKESLKATTKTAVSKLAKREEVVEEGAKEARSELAKELAKT

MAPH-1.1   231 ..............PAVPRPVA......................................
MAP1A      598 EKKAKESSEKPPEKPAKPERVKTESSEALKAEKRKLIKDKVGKKHLKEKISKLEEKKDKE

MAPH-1.1   239 ........................................GKPSARPATTT.........
MAP1A      658 KKEIKKERKELKKDEGRKEEKKDAKKEEKRKDTKPELKKISKPDLKPFTPEVRKTLYKAK

MAPH-1.1   250 ............................................................
MAP1A      718 VPGRVKIDRSRAIRGEKELSSEPQTPPAQKGTVPLPTISGHRELVLSSPEDLTQDFEEMK

MAPH-1.1   250 ..............................GTATRPTRPAVPA.................
MAP1A      778 REERALLAEQRDTGLGDKPFPLDTAEEGPPSTAIQGTPPSVPGLGQEEHVMKEKELVPEV

MAPH-1.1   263 ....ASAPRALTSRA.............................................
MAP1A      838 PEEQGSKDRGLDSGAETEEEKDTWEEKKQREAERLPDRTEAREESEPEVKEDVIEKAELE

MAPH-1.1   274 ............................................................
MAP1A      898 EMEEVHPSDEEEEDATKAEGFYQKHMQEPLKVTPRSREAFGGRELGLQGKAPEKETSLFL

MAPH-1.1   274 .....PAG.....................................PSRPTTTRNAAPAPR
MAP1A      958 SSLTTPAGATEHVSYIQDETIPGYSETEQTISDEEIHDEPEERPAPPRFHTSTYDLPGPE

MAPH-1.1   292 TAVP...........................................SRATVLTK.....
MAP1A     1018 GAGPFEASQPADSAVPATSGKVYGTPETELTYPTNIVAAPLAEEEHVSSATSITECDKLS
```

```
MAPH-1.1   304  ...............TAPASK...................................APTR
MAP1A     1078  SFATSVAEDQSVASLTAPQTEETGKSSLLLDTVTSIPSSRTEATQGLDYVPSAGTISPTS

MAPH-1.1   314  APVPARSAPAPP............RGA.................................
MAP1A     1138  SLEEDKGFKSPPCEDFSVTGESEKRGEIIGKGLSGERAVEEEEEETANVEMSEKLCSQYG

MAPH-1.1   329  ............PAKPAANTAKAEPIAQKKTVGKVQGTAPSKPAPAAPASAATSPA....
MAP1A     1198  TPVFSAPGHALHPGEPALGEAEERCLSPDDSTVKMASPPPSGPPSATHTPFHQSPVEEKS

MAPH-1.1   373  ...............................PAPE.........APRRDP..........
MAP1A     1258  EPQDFQEADSWGDTKRTPGVGKEDAAEETVKPGPEEGTLEKEEKVPPPRSPQAQEAPVNI

MAPH-1.1   383  .......................................................NNVTI
MAP1A     1318  DEGLTGCTIQLLPAQDKAIVFEIMEAGEPTGPILGAEALPGGLRTLPQEPGKPQKDEVLR

MAPH-1.1   388  VLDDSLSPEDFNQGSAPMDIVVIP........PTPEPPRHEVAQATH....PEESIIDAE
MAP1A     1378  YPDRSLSPED....AESLSVLSVPSPDTANQEPTPKSPCGLTEQYLHKDRWPEVSPEDTQ

MAPH-1.1   436  DLA..............................KAPLEVDDVANLADVED........EI
MAP1A     1434  SLSLSEESPSKETSLDVSSKQLSPESLGTLQFGELNLGKEEMGHLMQAEDTSHHTAPMSV

MAPH-1.1   458  P........PPVDAF.............................................
MAP1A     1494  PEPHAATASPPTDGTTRYSAQTDITDDSLDRKSPASSFSHSTPSGNGKYLPGAITSPDEH

MAPH-1.1   465  .........KKPEPHPEPNVSG................GSEEDKIPEPVD..........
MAP1A     1554  ILTPDSSFSKSPESLPGPALEDIAIKWEDKVPGLKDRTSEQKKEPEPKDEVLQQKDKTLE

MAPH-1.1   490  ........................AFKKPD..............................
MAP1A     1614  HKEVVEPKDTAIYQKDEALHVKNEAVKQQDKALEQKGRDLEQKDTALEQKDKALEPKDKD

MAPH-1.1   496  ............................................................
MAP1A     1674  LEEKDKALEQKDKIPEEKDKALEQKDTALEQKDKALEPKDKDLEQKDRVLEQKEKIPEEK

MAPH-1.1   496  ............................................................
MAP1A     1734  DKALDQKVRSVEHKAPEDTVAEMKDRDLEQTDKAPEQKHQAQEQKDKVSEKKDQALEQKY

MAPH-1.1   496  ............................................................
MAP1A     1794  WALGQKDEALEQNIQALEENHQTQEQESLVQEDKTRKPKMLEEKSPEKVKAMEEKLEALL

MAPH-1.1   496  ............................................................
MAP1A     1854  EKTKALGLEESLVQEGRAREQEEKYWRGQDVVQEWQETSPTREEPAGEQKELAPAWEDTS

MAPH-1.1   496  .................................PVELD......................
MAP1A     1914  PEQDNRYWRGREDVALEQDTYWRELSCERKVWFPHELDGQGARPHYTEERESTFLDEGPD

MAPH-1.1   501  ..................DFDPLKPSHPE...............................
MAP1A     1974  DEQEVPLREHATRSPWASDFKDFQESSPQKGLEVERWLAESPVGLPPEEEDKLTRSPFEI

MAPH-1.1   512  .........................................................PSA
MAP1A     2034  ISPPASPPEMVGQRVPSAPGQESPIPDPKLMPHMKNEPTTPSWLADIPPWVPKDRPLPPA

MAPH-1.1   515  PVVPSDHIIIATPDPELP..........DIVAAV..........................
MAP1A     2094  PLSPAPGPPTPAPESHTPAPFSWGTAEYDSVVAAVQEGAAELEGGPYSPLGKDYRKAEGE
```

```
MAPH-1.1   539 ............................................................
MAP1A     2154 REEEGRAEAPDKSSHSSKVPEASKSHATTEPEQTEPEQREPTPYPDERSFQYADIYEQMM

MAPH-1.1   539 ............................................................
MAP1A     2214 LTGLGPACPTREPPLGAAGDWPPCLSTKEAAAGRNTSAEKELSSPISPKSLQSDTPTFSY

MAPH-1.1   539 .....PFVSPSP.......................KGPNDGL..................
MAP1A     2274 AALAGPTVPPRPEPGPSMEPSLTPPAVPPRAPILSKGPSPPLNGNILSCSPDRRSPSPKE

MAPH-1.1   553 ........VKLDDELEK.................................IAPGFEEPLI
MAP1A     2334 SGRSHWDDSTSDSELEKGAREQPEKEAQSPSPPHPIPMGSPTLWPETEAHVSPPLDSHLG

MAPH-1.1   572 PQAPRDD........GTLAECSEEVSKLVEISMDTDNSAEVAAD..LAKAVGEVTQLSAD
MAP1A     2394 PARPSLDFPASAFGFSSLQPAPPQLPSPAEPRSAPCGSLAFSGDRALALAPGPPTR....

MAPH-1.1   622 LQNLGLDEKTDEYVRKLSNQMIEDATLP..........................FTSALA
MAP1A     2450 .......TRHDEYLEVTKAPSL.DSSLPQLPSPSSPGAPLLSNLPRPASPALSEGSSSEA

MAPH-1.1   656 SSIVTSNGSETNGHGEQAHAAQNGGIDHQKEIPKHDL.......................
MAP1A     2502 TTPVISSVAERFSPSLEAAEQESGELDPGMEPAAHSLWDLTPLSPAPPASLDLALAPAPS

MAPH-1.1   693 .............MQSRSSVIENGAAVQYEKTD...........................
MAP1A     2562 LPGDMGDGILPCHLECSEAATEKPSPFQVPSEDCAANGPTETSPNPPGPAPAKAENEEAA

MAPH-1.1   713 ............................................................
MAP1A     2622 ACPAWERGAWPEGAERSSRPDTLLSPEQPVCPAGGSGGPPSSASPEVEAGPQGCATEPRP

MAPH-1.1   713 ...............PALDD........................................
MAP1A     2682 HRGELSPSFLNPPLPPSIDDRDLSTEEVRLVGRGGRRRVGGPGTTGGPCPVTDETPPTSA

MAPH-1.1   718 ......................VLNACAQESEKID............ASHPDNLHMPA..
MAP1A     2742 SDSGSSQSDSDVPPETEECPSITAEAALDSDEDGDFLPVDKAGGVSGTHHPRPGHDPPPL

MAPH-1.1   742 .............................................APGSAAPAKPVKF..
MAP1A     2802 PQPDPRPSPPRPDVCMADPEGLSSESGRVERLREKEKVQGRVGRRAPGKAKPASPARRLD

MAPH-1.1   755 .....................ARPYYFDVVTVPRNEKLETSVAADGL.............
MAP1A     2862 LRGKRSPTPGKGPADRASRAPPRPRSTTSQVTPAEEKDGHSPMSKGLVNGLKAGPMALSS

MAPH-1.1   781 .........................QEFISKVRSRNVILASKDISGEQ.....LQAILCG
MAP1A     2922 KGSSGAPVYVDLAYIPNHCSGKTADLDFFRRVRASYYVVSGNDPANGEPSRAVLDALLEG

MAPH-1.1   811 KQTWCDSAHPCNVIPTHSSPMLLDFRQKNEEQFAANNLQFSIPVEKQRTTV.....SSDA
MAP1A     2982 KAQWGENLQ.VTLIPTHDTEVTREWYQQTHEQ....QQQLNVLVLASSSTVVMQDESFPA

MAPH-1.1   866 GAIEYELARVDLL
MAP1A     3037 CKIEF........
```
