# Supplementary material for: A tissue-specific protein purification approach in Caenorhabditis elegans identifies novel interaction partners of DLG-1/Discs large
Source: BMC Biol. 2016 Aug 9;14:66. doi: 10.1186/s12915-016-0286-x (PMC4977824; doi:10.1186/s12915-016-0286-x)
Supplement: Additional file 16: — PDF document with all vector maps and sequences. (PDF 1724 kb) [file 12915_2016_286_MOESM16_ESM.pdf]

ATAD-3 (-ETAV) in pCFJ1178 (10767 bp)

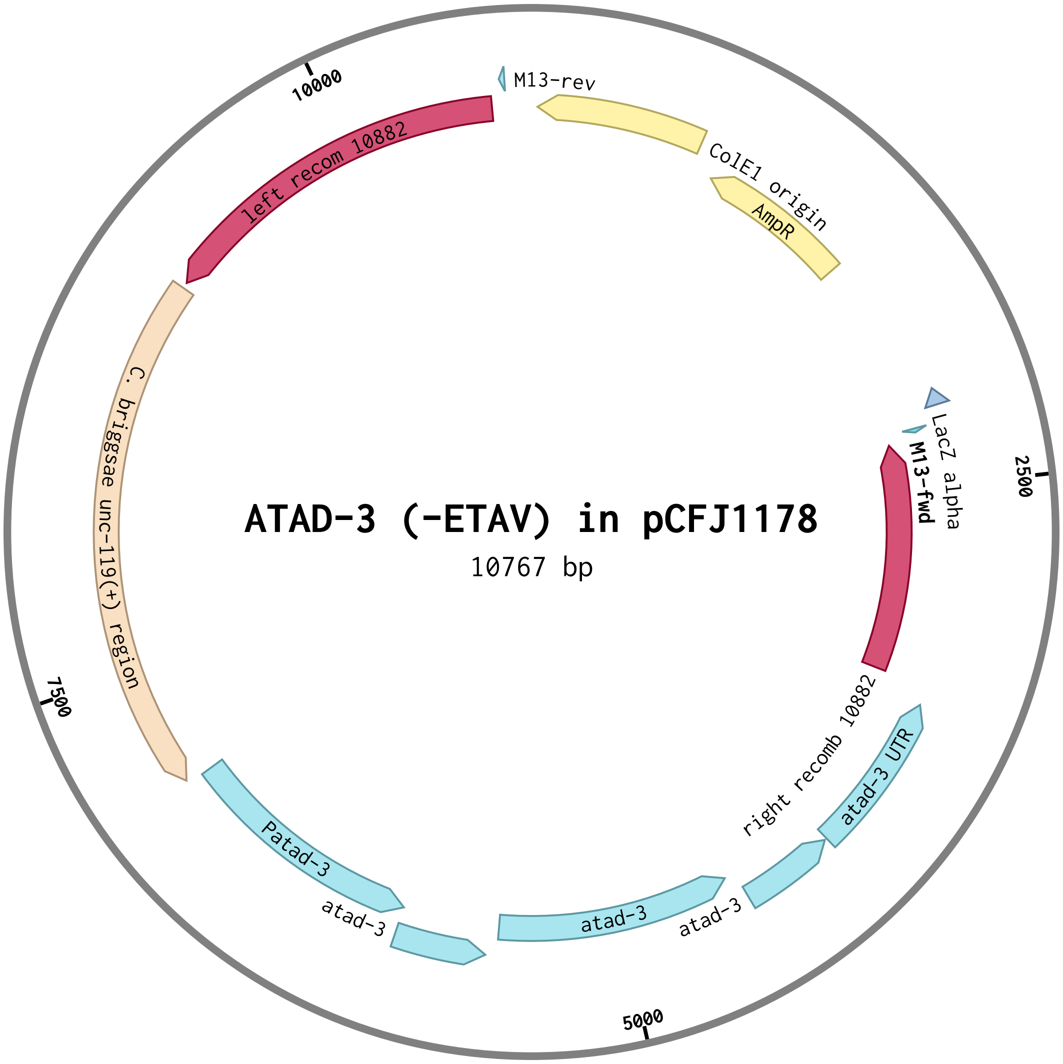

ATAD-3 in pCFJ1178 (10862 bp)

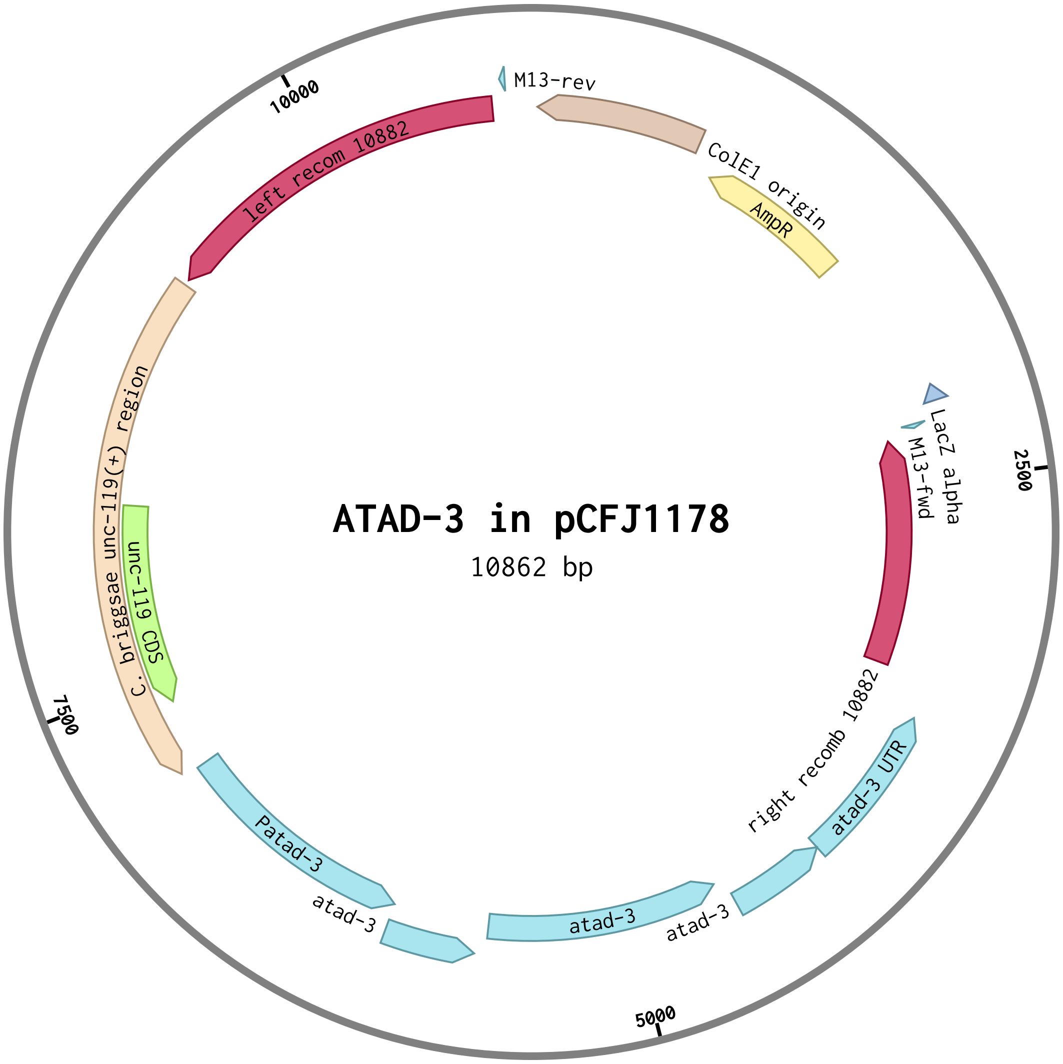

maph-1.1::GFP repair template (10528 bp)

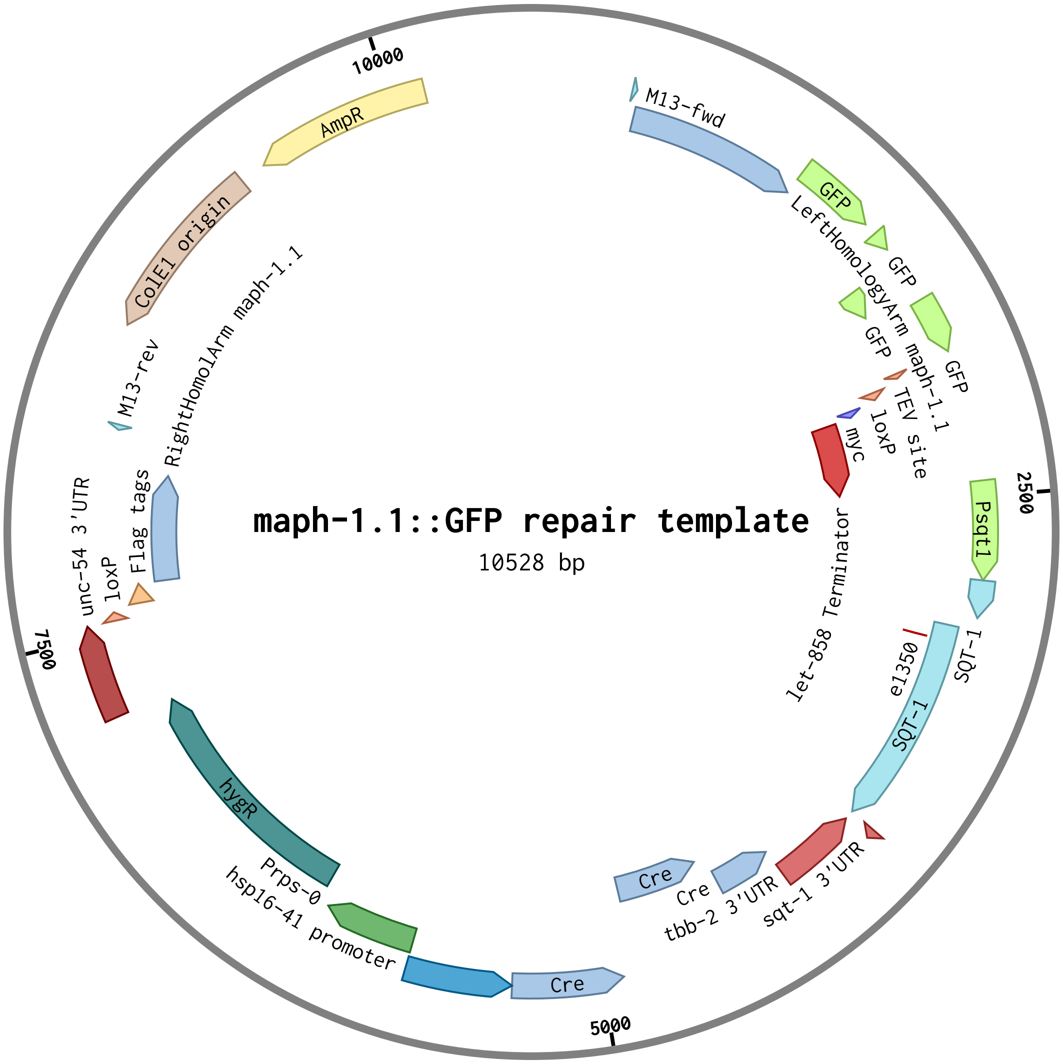

Pbeta actin eGFP::MAPH-1.1 (9407 bp)

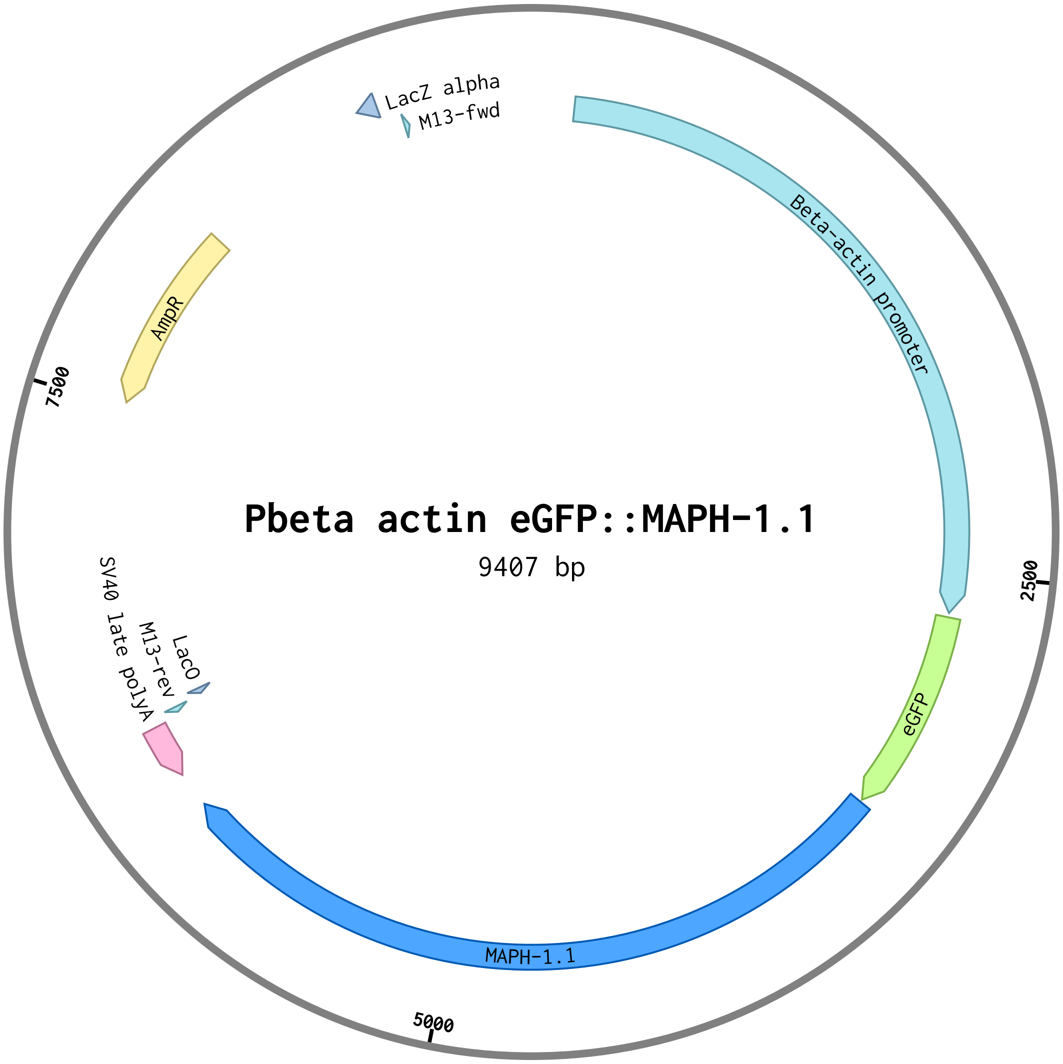

Pbeta actin DLG-1::mCherry (9662 bp)

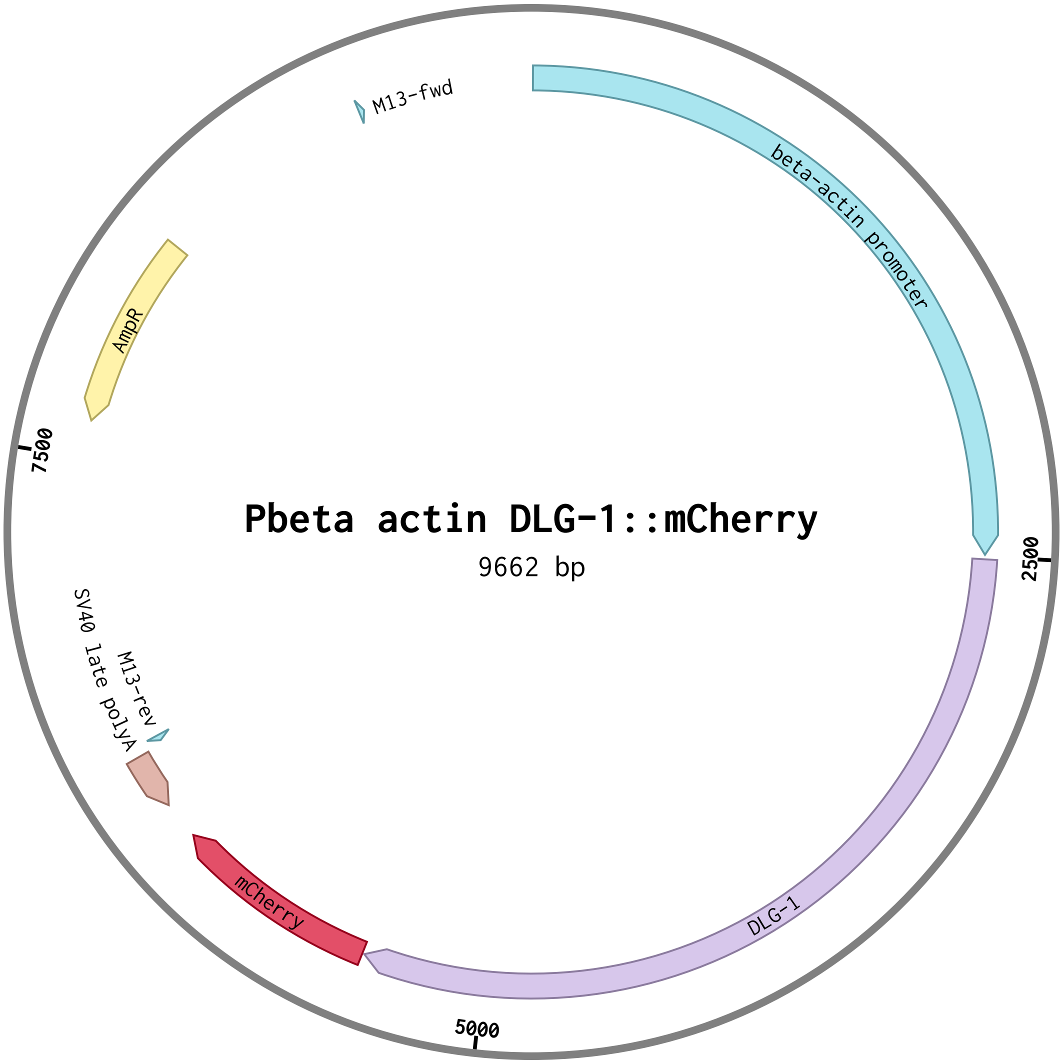

pBT331 (Prgef-1::BirA) (8349 bp)

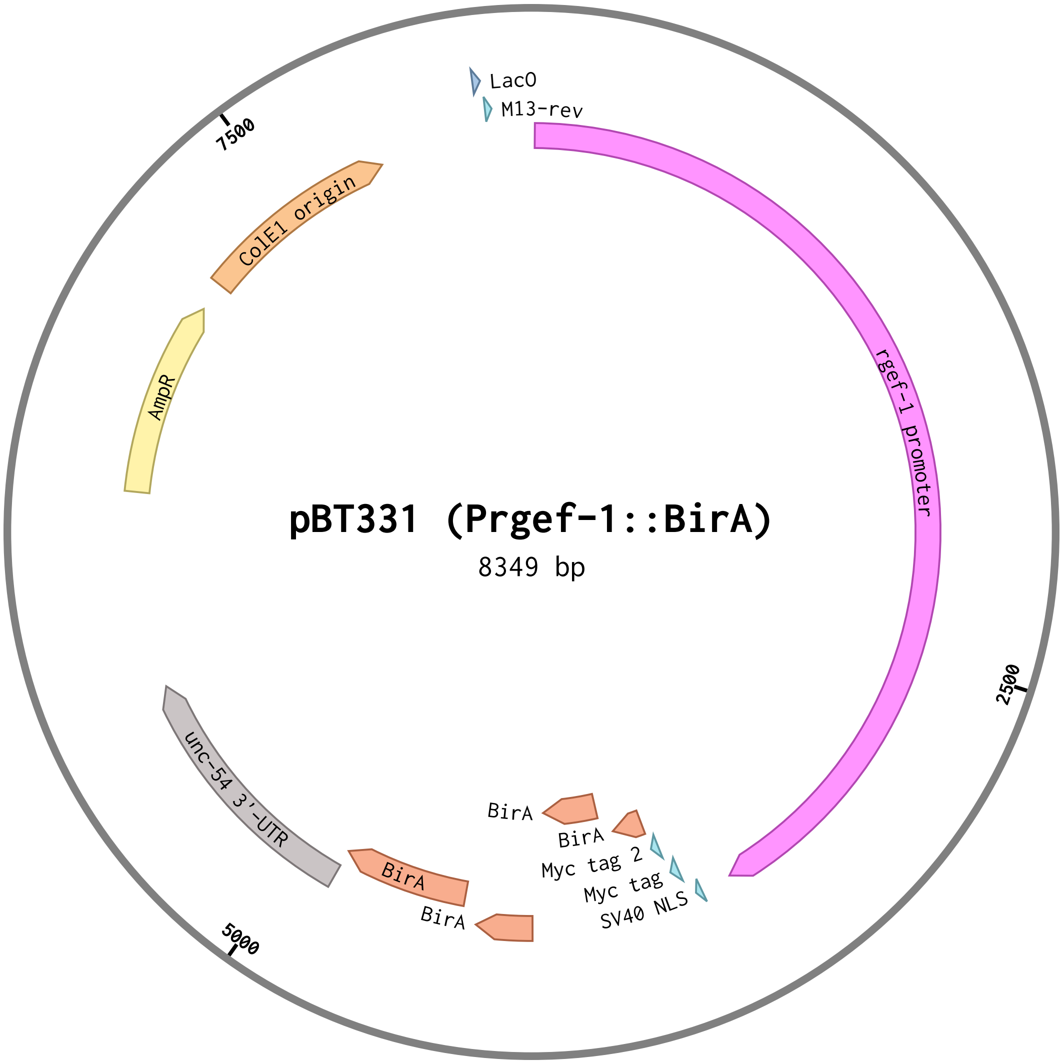

pJJR50 (3425 bp)

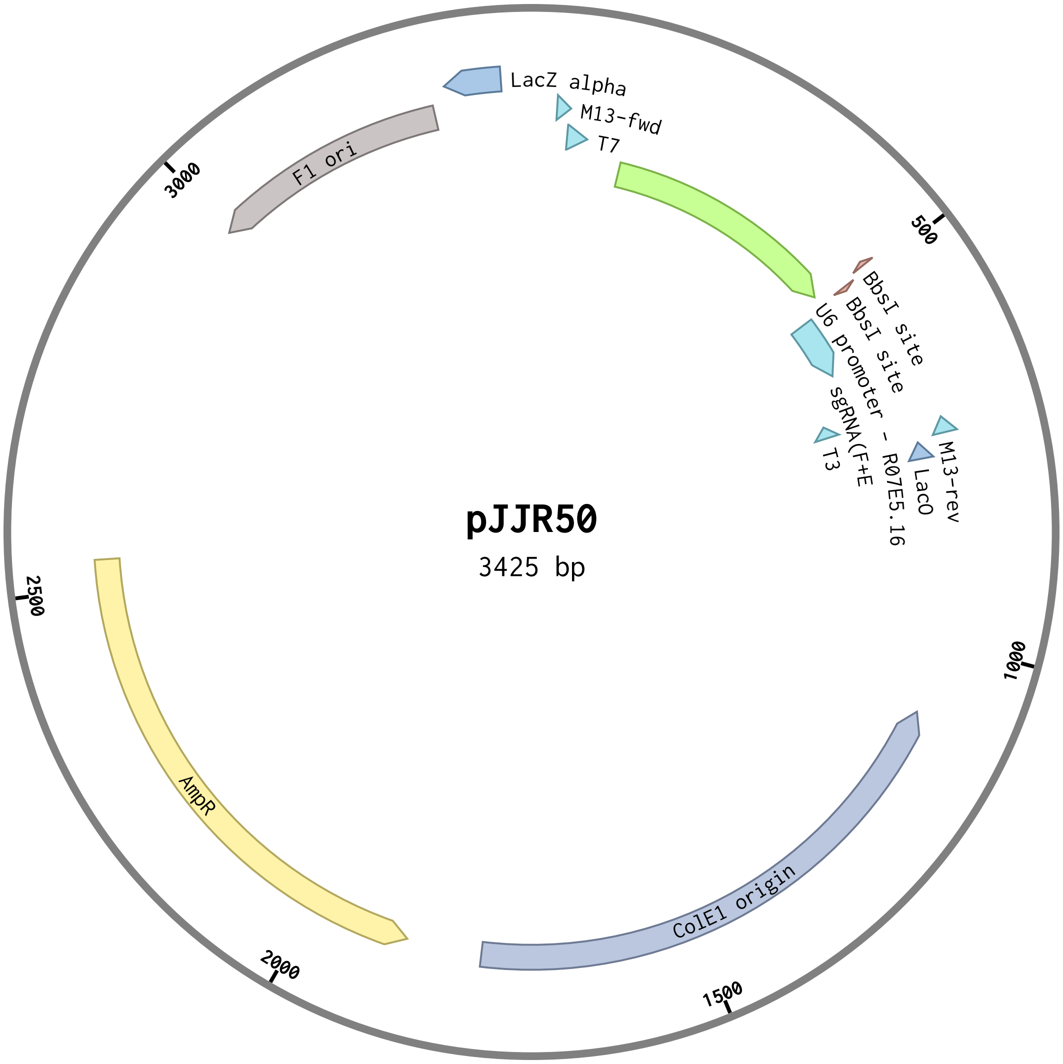

pMB37 (Prps-27::BirA) (5164 bp)

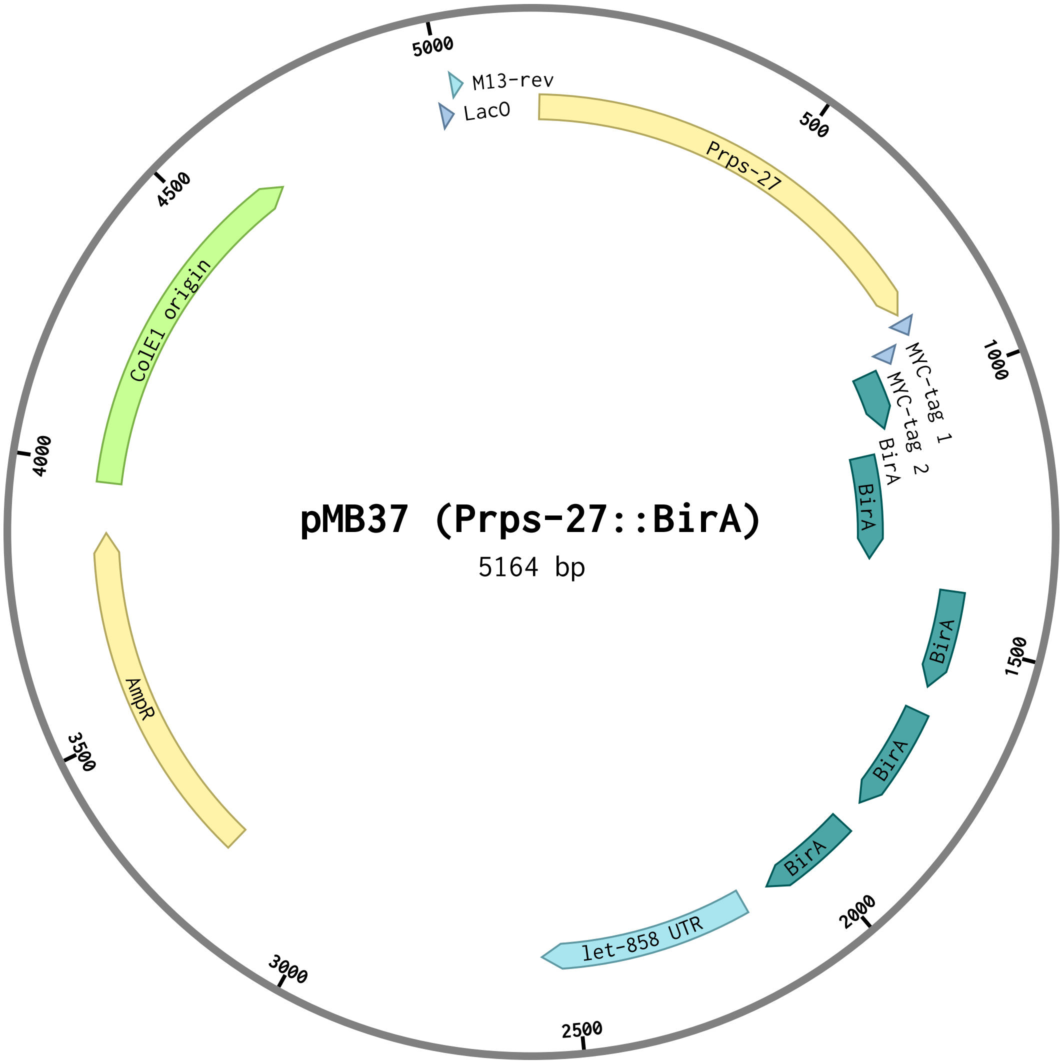

pMB41 (N-term Avi-2xTEV-GFP) (5052 bp)

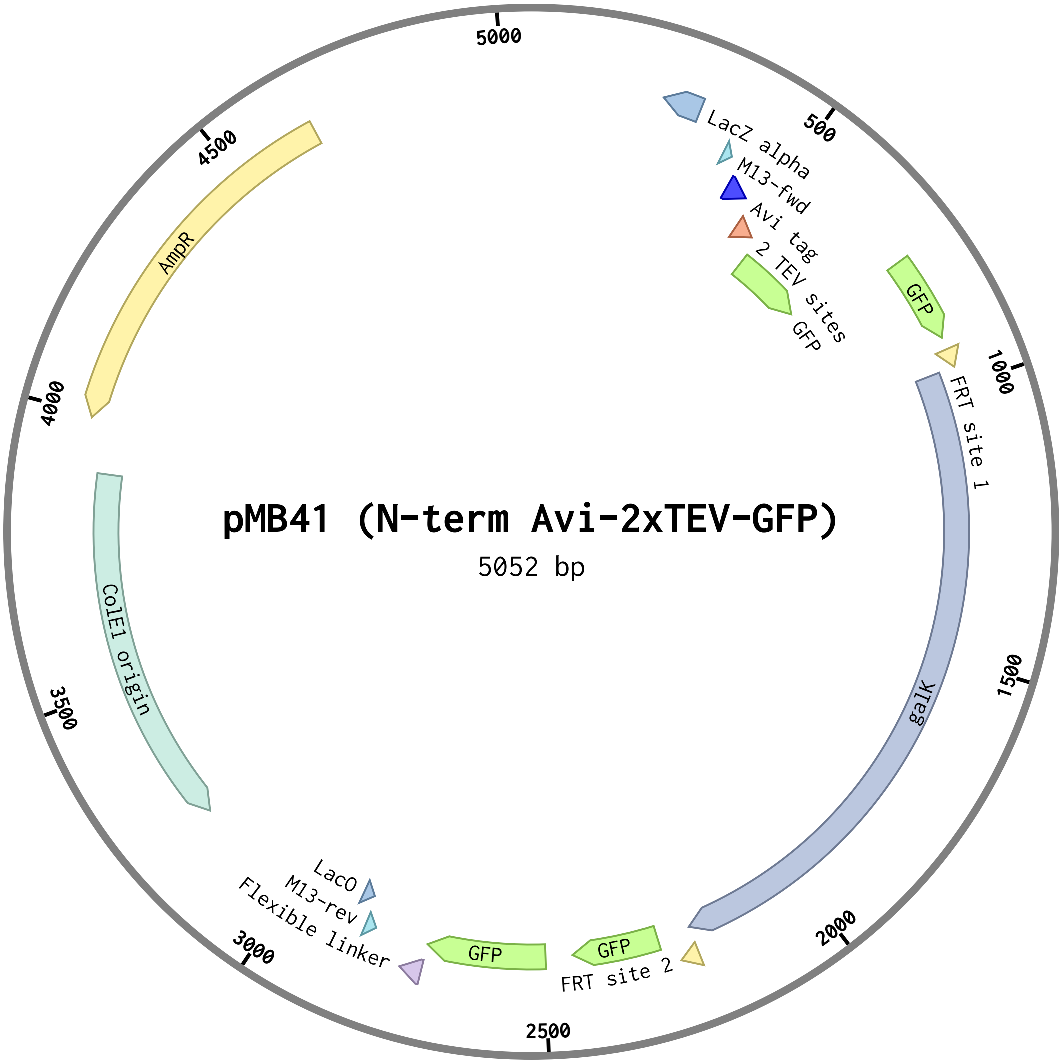

pMB43 (4876 bp)

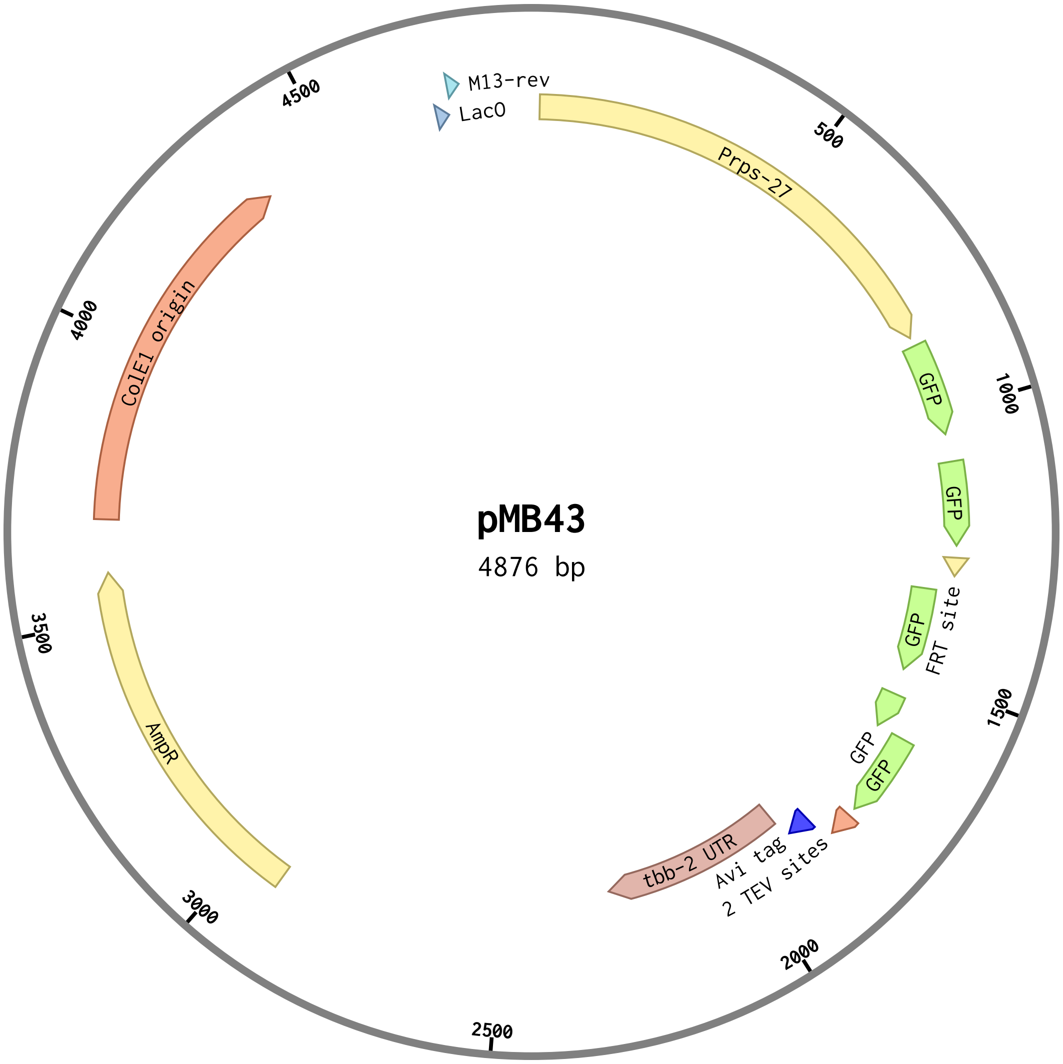

pMB71 (Pelt-2::BirA) (7993 bp)

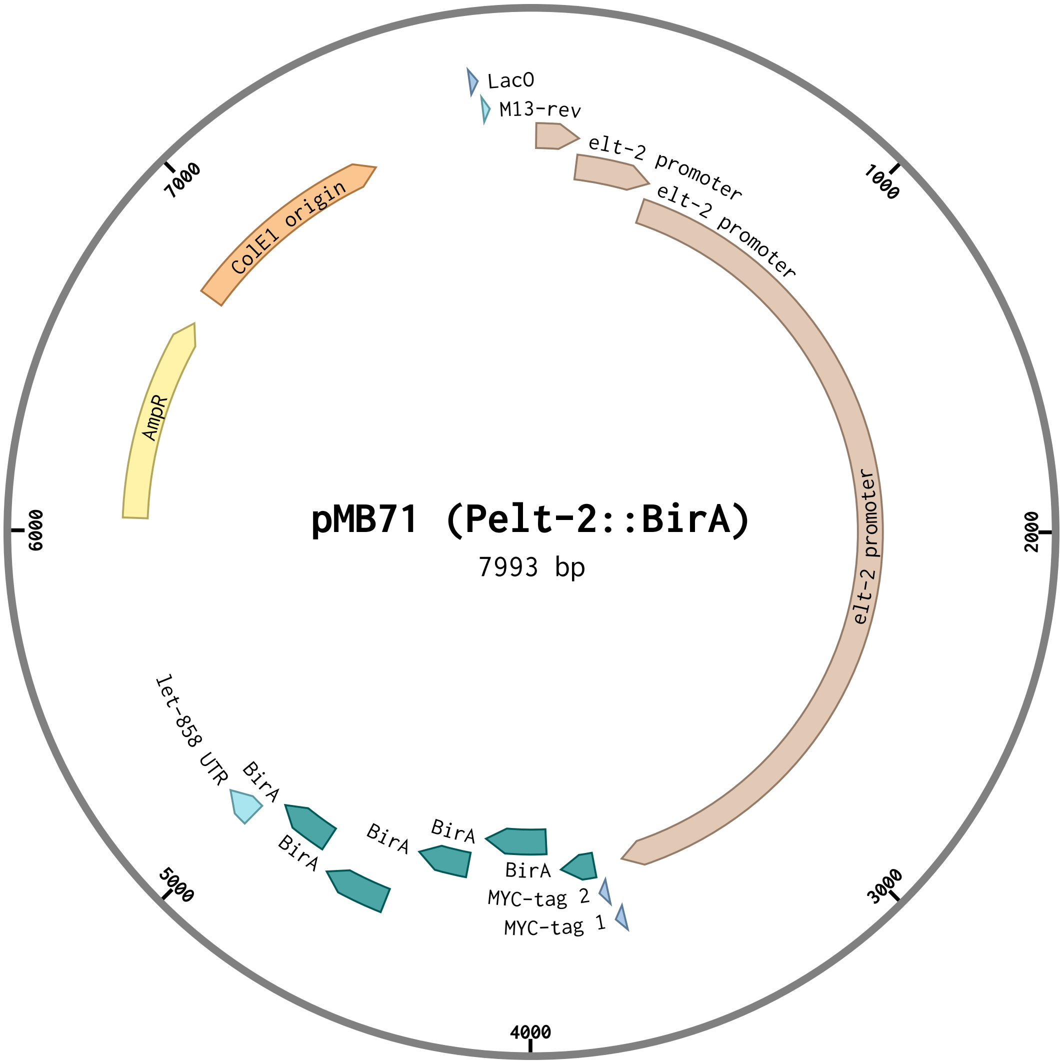

pMB72 (C-term GFP-2xTEV-Avi) (5052 bp)

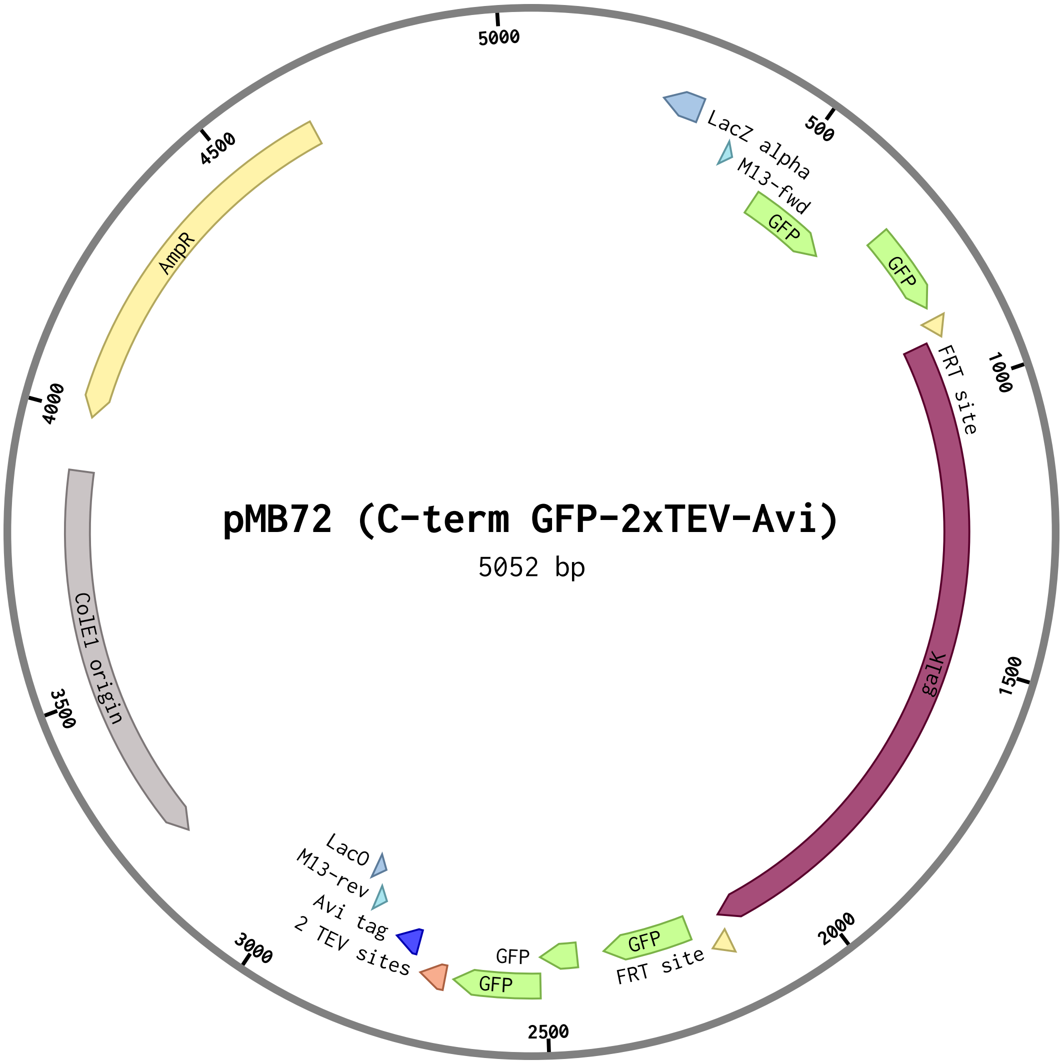

pMB73 (Pwrt-2::BirA) (5999 bp)

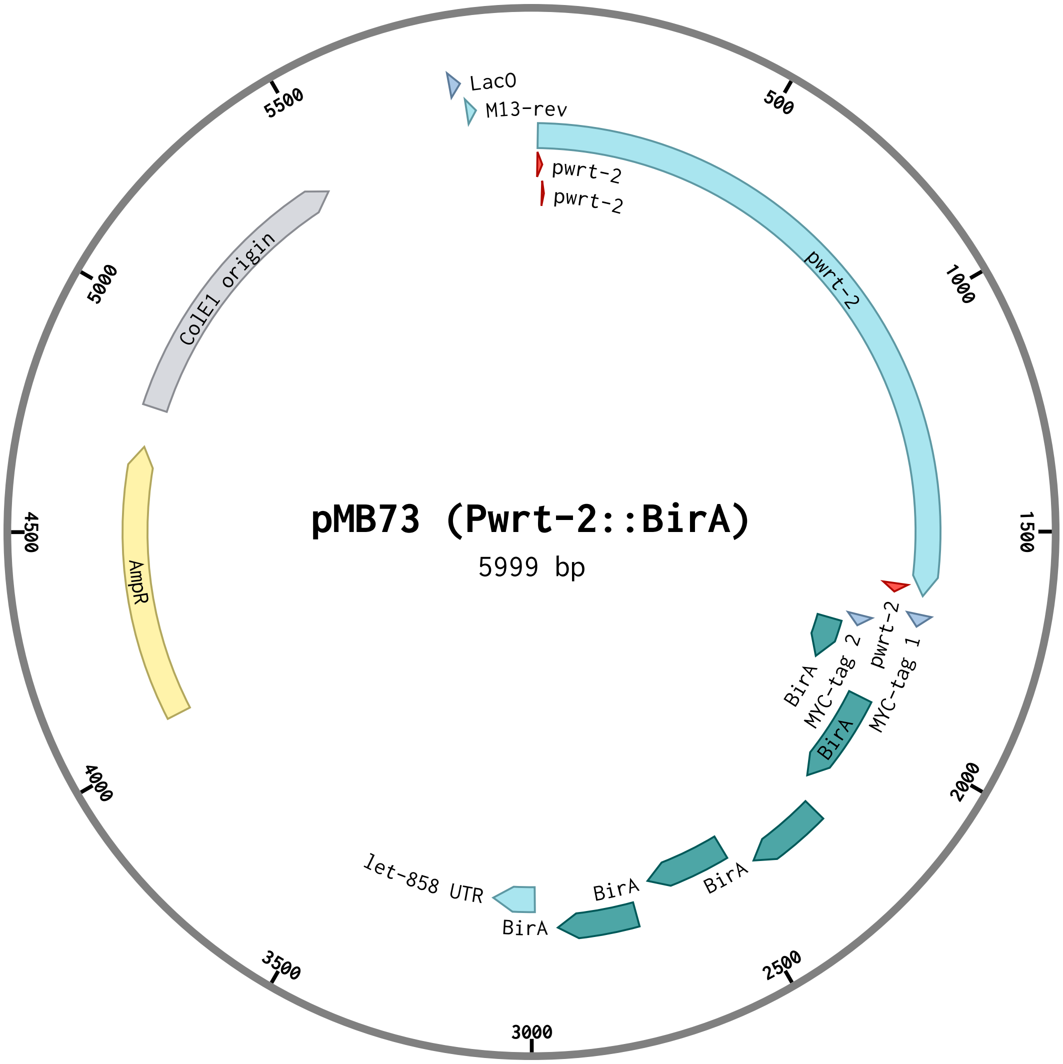

pMB76 (7778 bp)

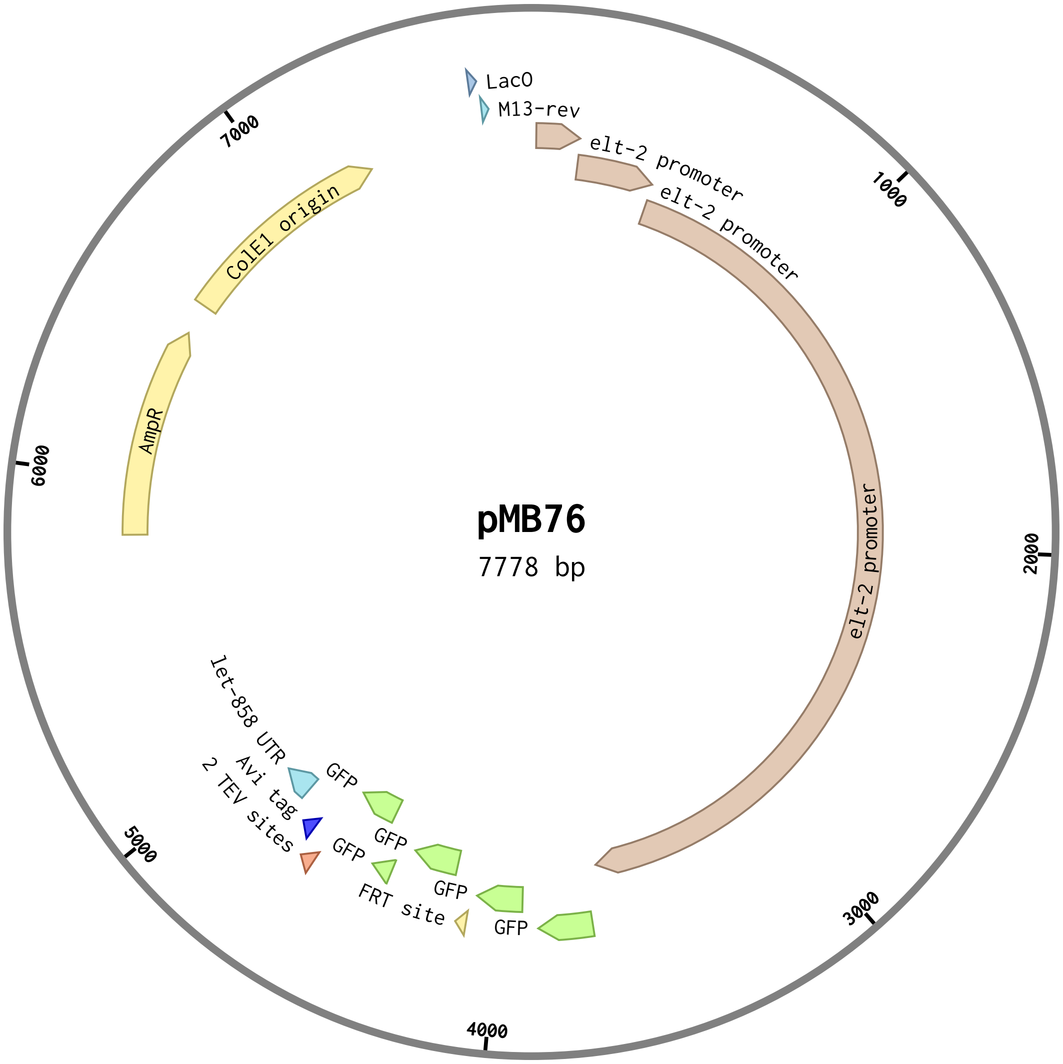

pMB77 (5784 bp)

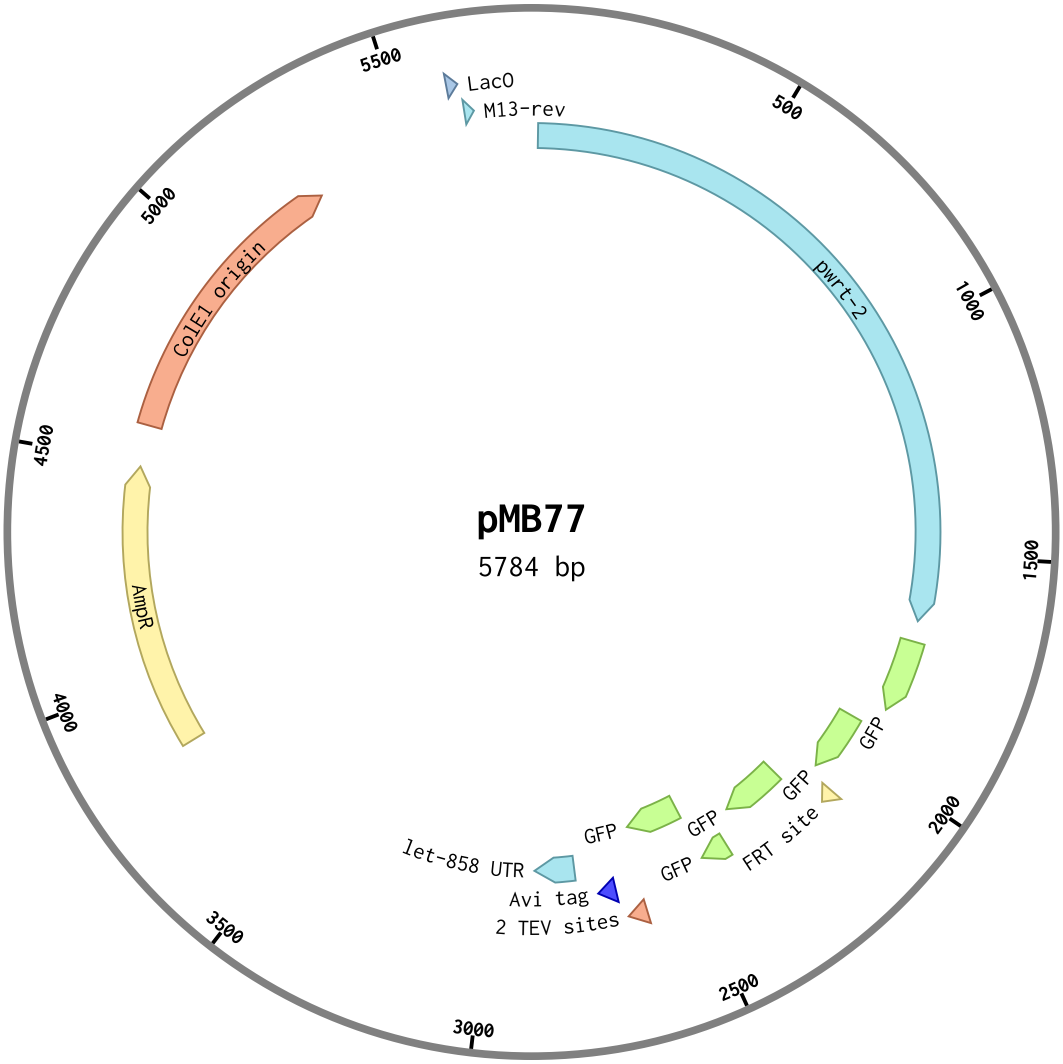

ATAD-3 (-ETAV) in pCFJ1178 (10767 bp)

gcaaaagaccaggaaccgtaaaaaggccgcgttgctggcgtttttccataggctccgcccccctgacgagcatcacaaaaatcgacgctcaagtcagaggtggcgaa
cgttttctggtccttggcatttttccggcgcaacgaccgcaaaaaggtatccgaggcggggggactgctcgtagtgtttttagctgcgagttcagtctccaccgctt
ColE1 origin
20 40 60 80 100

acccgacaggactataaagataccaggcgtttccccctggaagctccctcgtgcgctctcctgttccgaccctgccgcttaccggatacctgtccgcctttctccct
tgggctgtcctgatatttctatggtccgcaaagggggaccttcgagggagcacgcgagaggacaaggctgggacggcgaatggcctatggacaggcggaaagaggga
ColE1 origin
120 140 160 180 200

tcgggaagcgtggcgctttctcatagctcacgctgtaggtatctcagttcggtgtaggtcgttcgctccaagctgggctgtgtgcacgaaccccccgttcagcccga
agcccttcgcaccgcgaaagagtatcgagtgcgacatccatagagtcaagccacatccagcaagcgaggttcgacccgacacacgtgcttggggggcaagtcgggct
ColE1 origin
220 240 260 280 300 320

ccgctgcgccttatccggtaactatcgtcttgagtccaacccggtaagacacgacttatcgccactggcagcagccactggtaacaggattagcagagcgaggtatg
ggcgacgcggaataggccattgatagcagaactcaggttgggccattctgtgctgaatagcggtgaccgtcgtcggtgaccattgtcctaatcgtctcgctccatac
ColE1 origin
340 360 380 400 420

taggcggtgctacagagttcttgaagtggtggcctaactacggctacactagaaggacagtatttggtatctgcgctctgctgaagccagttaccttcggaaaaaga
atccgccacgatgtctcaagaacttcaccaccggattgatgccgatgtgatcttcctgtcataaaccatagacgcgagacgacttcggtcaatggaagcctttttct
ColE1 origin
440 460 480 500 520

gttggtagctcttgatccggcaaacaaaccaccgctggtagcggtggtttttttgtttgcaagcagcagattacgcgcagaaaaaaaggatctcaagaagatccttt
caaccatcgagaactaggccgtttgtttggtggcgaccatcgccaccaaaaaaacaaacgttcgtcgtctaatgcgcgtctttttttcctagagttcttctaggaaa
ColE1 origin
540 560 580 600 620 640

gatcttttctacggggtctgacgctcagtggaacgaaaactcacgttaagggattttggtcatgagattatcaaaaaggatcttcacctagatccttttaaattaaa
ctagaaaagatgccccagactgcgagtcaccttgcttttgagtgcaattccctaaaaccagtactctaatagtttttcctagaagtggatctaggaaaatttaattt
ColE1 origin
660 680 700 720 740

aatgaagttttaaatcaatctaaagtatatatgagtaaacttggtctgacagttaccaatgcttaatcagtgaggcacctatctcagcgatctgtctatttcgttca
ttacttcaaaatttagttagatttcatatatactcatttgaaccagactgtcaatggttacgaattagtcactccgtggatagagtcgctagacagataaagcaagt
AmpR
760 780 800 820 840

ATAD-3 (-ETAV) in pCFJ1178 (10767 bp) (from 857-1819 bp)

tccatagttgcctgactccccgtcgtgtagataactacgatacgggagggcttaccatctggccccagtgctgcaatgataccgcgagacccacgctcaccggctcc
aggtatcaacggactgaggggcagcacatctattgatgctatgccctcccgaatggtagaccggggtcacgacgttactatggcgctctgggtgcgagtggccgagg

AmpR

860 880 900 920 940 960

agatttatcagcaataaaccagccagccggaagggccgagcgcagaagtggtcctgcaactttatccgcctccatccagtctattaattgttgccgggaagctagag
tctaaatagtcgttatttggtcggtcggccttcccggctcgcgtcttcaccaggacgttgaaataggcggaggtaggtcagataattaacaacggcccttcgatctc

AmpR

980 1,000 1,020 1,040 1,060

taagtagttcgccagttaatagtttgcgcaacgttgttgccattgctacaggcatcgtggtgtcacgctcgtcgtttggtatggcttcattcagctccggttcccaa
attcatcaagcggtcaattatcaaacgcgttgcaacaacggtaacgatgtccgtagcaccacagtgcgagcagcaaaccataccgaagtaagtcgaggccaagggtt

AmpR

1,080 1,100 1,120 1,140 1,160

cgatcaaggcgagttacatgatcccccatgttgtgcaaaaaagcggttagctccttcggtcctccgatcgttgtcagaagtaagttggccgcagtgttatcactcat
gctagttccgctcaatgtactagggggtacaacacgttttttcgccaatcgaggaagccaggaggctagcaacagtcttcattcaaccggcgtcacaatagtgagta

AmpR

1,180 1,200 1,220 1,240 1,260 1,280

ggttatggcagcactgcataattctcttactgtcatgccatccgtaagatgcttttctgtgactggtgagtactcaaccaagtcattctgagaatagtgtatgcggc
ccaataccgtcgtgacgtattaagagaatgacagtacggtaggcattctacgaaaagacactgaccactcatgagttggttcagtaagactcttatcacatacgccg

AmpR

1,300 1,320 1,340 1,360 1,380

gaccgagttgctcttgcccggcgtcaatacgggataataccgcgccacatagcagaactttaaaagtgctcatcattggaaaacgttcttcggggcgaaaactctca
ctggctcaacgagaacgggccgcagttatgccctattatggcgcggtgtatcgtcttgaaattttcacgagtagtaaccttttgcaagaagccccgcttttgagagt

AmpR

1,400 1,420 1,440 1,460 1,480

aggatcttaccgctgttgagatccagttcgatgtaacccactcgtgcacccaactgatcttcagcatcttttactttcaccagcgtttctgggtgagcaaaaacagg
tcctagaatggcgacaactctaggtcaagctacattgggtgagcacgtgggttgactagaagtcgtagaaaatgaaagtggtcgcaaagacccactcgtttttgtcc

1,500 1,520 1,540 1,560 1,580 1,600

aaggcaaaatgccgcaaaaaagggaataagggcgacacggaaatgttgaatactcatactcttcctttttcaatattattgaagcatttatcagggttattgtctca
ttccgttttacggcgttttttcccttattcccgctgtgcctttacaacttatgagtatgagaaggaaaaagttataataacttcgtaaatagtcccaataacagagt

1,620 1,640 1,660 1,680 1,700

tgagcggatacatatttgaatgtatttagaaaaataaacaaataggggttccgcgcacatttccccgaaaagtgccacctgacgtctaagaaaccattattatcatg
actcgcctatgtataaacttacataaatctttttatttgtttatccccaaggcgcgtgtaaaggggcttttcacggtggactgcagattctttggtaataatagtac

1,720 1,740 1,760 1,780 1,800

ATAD-3 (-ETAV) in pCFJ1178 (10767 bp) (from 1820-2782 bp)

acattaacctataaaaataggcgtatcacgaggccctttcgtctcgcgcgtttcggtgatgacggtgaaaacctctgacacatgcagctcccggagacggtcacagc
tgtaattggatatttttatccgcatagtgctccgggaaagcagagcgcgcaaagccactactgccacttttggagactgtgtacgtcgagggcctctgccagtgtcg

1,820 1,840 1,860 1,880 1,900 1,920

ttgtctgtaagcggatgccgggagcagacaagcccgtcagggcgcgtcagcgggtgttggcgggtgtcggggctggcttaactatgcggcatcagagcagattgtac
aacagacattcgcctacggccctcgtctgttcgggcagtcccgcgcagtcgcccacaaccgcccacagccccgaccgaattgatacgccgtagtctcgtctaacatg

1,940 1,960 1,980 2,000 2,020

tgagagtgcaccatatgcggtgtgaaataccgcacagatgcgtaaggagaaaataccgcatcaggcgccattcgccattcaggctgcgcaactgttgggaagggcga
actctcacgtggtatacgccacactttatggcgtgtctacgcattcctcttttatggcgtagtccgcggtaagcggtaagtccgacgcgttgacaacccttcccgct

LacZ alpha

2,040 2,060 2,080 2,100 2,120 2,140

tcggtgcgggcctcttcgctattacgccagctggcgaaagggggatgtgctgcaaggcgattaagttgggtaacgccagggttttcccagtcacgacgttgtaaaac
agccacgcccggagaagcgataatgcggtcgaccgctttccccctacacgacgttccgctaattcaacccattgcggtcccaaaagggtcagtgctgcaacattttg

LacZ alpha

2,160 2,180 2,200 2,220 2,240

gacggccagtgaattatcaactatgtataataaagttgcggcccattaggtcagacaaaaatttctgatctttaaaccttattgattaaaactaatttttgcccgac
ctgccggtcacttaatagttgatacatattatttcaacgccgggtaatccagtctgtttttaaagactagaaatttggaataactaatttttgattaaaaacgggctg

right recomb 10882

2,260 2,280 2,300 2,320 2,340

tttttgacacatgcactagacgcttgtactcaaaatttggaaaccttgctcaaatccactactcagatatgacagtagcatcgataattgaaccattatcctgaaa
aaaaaactgtgtacgtgatctgcgaacatgagttttaaacctttggaacgagtttaggtgatgagtctatactgtcatcgtagctattaacttggtaataggacttt

right recomb 10882

2,360 2,380 2,400 2,420 2,440 2,460

tccccttttcctgtcccggttcattttccaatttttcaacggtggaagaaggggcaaattttataccaaaacgtctccgtttatgacccacccccctatgcaaaataa
aggggaaaggacagggccaagtaaaaggttaaaaagttgccaccttcttccccgtttaaaatatggttttgcagaggcaaatactgggtgggggatacgttttatt

right recomb 10882

2,480 2,500 2,520 2,540 2,560

acggctctgggagaaatagtacagaaaacgcggagcctgcttgattccttctccgaggcgacaaaaaacgcatcattcgtattgtaagatgcatgtgtggctggcct
tgccgagaccctctttatcatgtcttttgcgcctcggacgaactaaggaagaggctccgctgttttttgcgtagtaagcataacattctacgtacacaccgaccgga

right recomb 10882

2,580 2,600 2,620 2,640 2,660

cttttgaattttactggtttttttatatttctactaatatttcgaaaaattttgtaagtaaatcattgattacgtttacaaaaatttagaaacatttcaaaatgatta
gaaaacttaaaatgaccaaaaaaatataaagatgattataaagctttttaaaacattcatttagtaactaatgcaaatgtttttaaatctttgtaaagttttactaat

right recomb 10882

2,680 2,700 2,720 2,740 2,760 2,780

ATAD-3 (-ETAV) in pCFJ1178 (10767 bp) (from 2783-3638 bp)

cttctaaattaattatatttacaggttagttcgagttacttatgaccttccaatccgccatatccgccaaggacgctccaattaatttttagaattattttttaac
gaagatttaattaatataaatgtccaatcaagctcaatgaatactggaaggttaggcggtataggcggttcctgcgaggttaattaaaaatcttaataaaaaattg

right recomb 10882

2,800 2,820 2,840 2,860 2,880

acatgtattatttttaacaataaattattcatgaaatcatcaaatgtatatgttctactataatatttaggtcaaactatagaaaagaaatccttgtttttggaac
tgtacataataaaaattgttatttaataagtactttagtagtttacatatacaagatgatattataaatccagtttgatatcttttctttaggaacaaaaaccttg

right recomb 10882

2,900 2,920 2,940 2,960 2,980

aaaggagttcagatcctgtgcatttgatcttggatccacatctttggtccatgggaaagcttggcactgatcaagaaggattcttcaaatatctaaaattgccggct
tttcctcaagtctaggacacgtaaactagaacctaggtgtagaaaccaggtaccctttcgaaccgtgactagttcttcctaagaagtttatagattttaacggccga

right recomb 10882

3,000 3,020 3,040 3,060 3,080 3,100

gcccgtagaattccatcagttttccagagttttagtgaaaatttgcatcccgttagaaacaatctacacaacggtaaaaaggacccattatttttaccgtaccgttt
cgggcatcttaaggtagtcaaaaggtctcaaaatcacttttaaacgtagggcaatctttgttagatgtgttgccatttttcctgggtaataaaaatggcatggcaaa

right recomb 10882

3,120 3,140 3,160 3,180 3,200

tcctttatggccctctatgcaaaaaattatagcgtggaggggcagtacagaataataaagtgtgagaaaaaaaactgctctgcgagaaatagtacagcaaacgcgga
aggaaataccgggagatacgtttttaatatcgcacctccccgtcatgtcttattatttcacactcttttttttgacgagacgctctttatcatgtcgtttgcgcct

right recomb 10882

3,220 3,240 3,260 3,280 3,300

gcatgcttgattctatccactttgtacaagaaagctgggttaatacgactcactagtgggcagatcttcgaatgcatcGcgcgcaccgtacgggatgcacggaagat
cgtacgaactaagataggtgaaacatgttctttcgacccaattatgctgagtgatcacccgtctagaagcttacgtagCgcgcgtggcatgccctacgtgccttcta

atad-3 UTR

3,320 3,340 3,360 3,380 3,400 3,420

tctgaaattattgtttttcaggatttctgatattgaaaattgtggtttctagcttttttattgttgcttttctggaaatattaggctaaaagctcataaacgtttaat
agactttaataacaaaaagtcctaaagactataacttttaacaccaaagatcgaaaaataacaacgaaaagacctttataatccgattttcgagtatttgcaaatta

atad-3 UTR

3,440 3,460 3,480 3,500 3,520

ttcaattgatttcttattttctgaaaaaaccatatattgccagctttttccttcttacctgtgcataaacagaaagacctctccggcctcccgcaagtttttttgttt
aagttaactaaagaataaaagactttttttggtatataacggtcgaaaaaggaagaatggacacgtatttgtctttctggagaggccggagggcgttcaaaaaaacaaa

atad-3 UTR

3,540 3,560 3,580 3,600 3,620

ATAD-3 (-ETAV) in pCFJ1178 (10767 bp) (from 3639-4494 bp)

cagtatccatttgatcgttttaatcgaacatttgggctgaaaattatttttttgctttttctcttcagaagctttcaaaatatgaaaaattgaagtgcaacaaaag
gtcataggtaaactagcaaaattagcttgtaaacccgacttttaataaaaaaaacgaaaaagagaagtcttcgaaagttttatactttttaacttcacgttgttttc

atad-3 UTR

3,640 3,660 3,680 3,700 3,720 3,740

cgattattagttagtattacattaaaatgaagagtttctttattaacaaaaaaaaaacaataatctttcactctacttaaattactacacaaaactaaataaaaat
gctaataatcaatcataatgtaattttacttctcaaagaaataattgttttttttttgttattagaaagtgagatgaatttaatgatgtgttttgatttattttta

atad-3 UTR

3,760 3,780 3,800 3,820 3,840

gactgacctagaaaataaggggaaaacgaaaaaaaacaatagaaaaacaagggcctaattgaattatctaaaaaaattatattatcagcaaaatttctgattaagaa
ctgactggatcttttattccccttttgcttttttttgttatctttttgttcccggattaacttaatagatttttttaatataatagtcgttttaaagactaattctt

atad-3 UTR

3,860 3,880 3,900 3,920 3,940

tgaaattacgaaattaccggaagtttgaagatttcagaaaagaaaaaacaacaaaaaaacgtaaatagaaaacaatagagtatcatgaacaaggaaatgaatatttt
actttaatgctttaatggccttcaaacttctaaagtcttttcttttttgttgtttttttgcatttatcttttgttatctcatagtacttgttcctttacttataaaa

atad-3 UTR

3,960 3,980 4,000 4,020 4,040 4,060

gaaacgtacgttaTCTCTTCAACGTAGTTCCGAACTTGACTTCTTGGTTACGAGCTTTCAATTGCTCCTTCTCAAGCCATTCCATCTTATGTTCATGTTGAACCATT
ctttgcatgcaatAGAGAAGTTGCATCAAGGCTTGAACTGAAGAACCAATGCTCGAAAGTTAACGAGGAAGAGTTCGGTAAGGTAGAATACAAGTACAACTTGGTAA

atad-3

4,080 4,100 4,120 4,140 4,160

GCGTCAGCAGTATTACGATCAACGATAGCCTCGGTGAGCACTCCGGTTTCAGATGCATATGCAGAAGCTTGCCATCCGATGACAAGTTTACTCAATTCTCTTCCACT
CGCAGTCGTCATAATGCTAGTTGCTATCGGAGCCACTCGTGAGGCCAAAGTCTACGTATACGTCTTCGAACGGTAGGCTACTGTTCAAATGAGTTAAGAGAAGGTGA

atad-3

4,180 4,200 4,220 4,240 4,260 4,280

CATTCCAGATGTCTTTTTGGCAACTTCATTACACTTAGCAACCCAATCAAAGTTATCGAGTTTCAGTCGTTGAGAACGAGATCCACTAGTTGCTGGAGTGACGATGT
GTAAGGTCTACAGAAAAACCGTTGAAGTAATGTGAATCGTTGGGTTAGTTTCAATAGCTCAAAGTCAGCAACTCTTGCTCTAGGTGATCAACGACCTCACTGCTACA

atad-3

4,300 4,320 4,340 4,360 4,380

GCTCGTTGAAGTATTGCAGAAGAATTCGTTCACGTTCTTCCATTCCTGGCAGTGTGAATTCGACAAGCTGATCGAAActgaaaagaataataaattctgtttttttat
CGAGCAACTTCATAACGTCTTCTTAAGCAAGTGCAAGAAGGTAAGGACCGTCACACTTAAGCTGTTCGACTAGCTTTgacttttcttattatttaagacaaaaaata

atad-3

4,400 4,420 4,440 4,460 4,480

ATAD-3 (-ETAV) in pCFJ1178 (10767 bp) (from 4495-5350 bp)

```
ttaaaaaaaaggattacCGATCGTTAACTGCCCAATCGAACTGCTCTGGCTGATTAGAAGCCACGACAAGCATGAACTTTCTCGACTGCTCTCCAGTTCTGAAGAGG
aatttttttttcctaatgGCTAGCAATTGACGGGTTAGCTTGACGAGACCGACTAATCTTCGGTGCTGTTCGTACTTGAAAGAGCTGACGAGAGGTCAAGACTTCTCC
atad-3
4,500 4,520 4,540 4,560 4,580 4,600

AAAGCGTTGAGAGCTGCACGTGTGTCTTCAGACATTCCATTCTTTGATCTCTTTTGCAAAAAAGCGTCAGCTTCGTCAATAAAAACAATCAACCCTTTGCGACTTTT
TTTCGCAACTCTCGACGTGCACACAGAAGTCTGTAAGGTAAGAAACTAGAGAAAACGTTTTTTCGCAGTCGAAGCAGTTATTTTTGTTAGTTGGGAAACGCTGAAAA
atad-3
4,620 4,640 4,660 4,680 4,700

TGAAGCCCAGTCGAATACTTTATGAATTGCAGATACTCCATCACGTCCAAGTGGAGCAATATCTCCTCCAGTGAGAACTGCGTAATCGAGTCCGGAATGTTGAGCCA
ACTTCGGGTCAGCTTATGAAATACTTAACGTCTATGAGGTAGTGCAGGTTCACCTCGTTATAGAGGAGGTCACTCTTGACGCATTAGCTCAGGCCTTACAACTCGGT
atad-3
4,720 4,740 4,760 4,780 4,800

AACTCTTTGCGAAAAGTGTCTTTCCGGTTCCTGGTGGTCCGTAGAACATTACATTACGGAAAAGTCCATTATTTCTCTTTGTGTTTGAGGTGGTAATGGCAATATCA
TTGAGAAACGCTTTTCACAGAAAGGCCAAGGACCACCAGGCATCTTGTAATGTAATGCCTTTTCAGGTAATAAAGAGAAACACAAACTCCACCATTACCGTTATAGT
atad-3
4,820 4,840 4,860 4,880 4,900 4,920

CGAAGACGACGTTCCAAAGCAGGTGGTAATACAACTCCATTCAGTGGATCCTTCTTCTGACGAGTCATCATTTGAACACTTTTAATTGGGTGTTTGAGTACTTCAAG
GCTTCTGCTGCAAGGTTTCGTCCACCATTATGTTGAGGTAAGTCACCTAGGAAGAAGACTGCTCAGTAGTAAACTTGTGAAAATTAACCCACAAACTCATGAAGTTC
atad-3
4,940 4,960 4,980 5,000 5,020

TGGAGTGATTCTGGATGTTTCTCTTACAAGACTTGGTTTTCCGAGACGAGATTCGATGTATCTTGCGGTGACTCCAGTTCCTCGTTTAGCGGTATACCAGCCGACAG
ACCTCACTAAGACCTACAAAGAGAATGTTCTGAACCAAAAGGCTCTGCTCTAAGCTACATAGAACGCCACTGAGGTCAAGGAGCAAATCGCCATATGGTCGGCTGTC
atad-3
5,040 5,060 5,080 5,100 5,120

CCAAAGCAGTCAATCCTCCTACTGCAGCAGCGATTTTCGTTTTATCATTCAAAAATTGATTGAGTCCAGAACCGATAAGTTCTCCACTGGTTTTAATTTTTTCAATG
GGTTTCGTCAGTTAGGAGGATGACGTCGTCGCTAAAAGCAAAATAGTAAGTTTTTAACTAACTCAGGTCTTGGCTATTCAAGAGGTGACCAAAATTAAAAAAGTTAC
atad-3
5,140 5,160 5,180 5,200 5,220 5,240

ACAGTTTTTCGGTTCTCTTCTTCGTGAAGCTTCATTTGCTCTAAGTTTACATCTCTGTTATCTCGAGCAGCCTTTGCCCTGGCTCTGGTCTCTGCGTCAATTTTCTC
TGTCAAAAAGCCAAGAGAAGAAGCACTTCGAAGTAAACGAGATTCAAATGTAGAGACAATAGAGCTCGTCGGAAACGGGACCGAGACCAGAGACGCAGTTAAAAGAG
atad-3
5,260 5,280 5,300 5,320 5,340
```

ATAD-3 (-ETAV) in pCFJ1178 (10767 bp) (from 5351-6206 bp)

GAGCTCGTATTTGTGTTTTAATGCTAACTCATGCTCAATAGTTTGCTTGCGAAGTTGTTCCTGCTTCTTGACAGACTCTTCTTGTTTTCGGAGGCTTTCTTCCTGCA
CTCGAGCATAAACACAAAATTACGATTGAGTACGAGTTATCAAACGAACGCTTCAACAAGGACGAAGAACTGTCTGAGAAGAACAAAAGCCTCCGAAAGAAGGACGT
atad-3
5,360 5,380 5,400 5,420 5,440

TTCTAGCTTTCATGGCAAGCTCCTCTTCGGCTCTCTTTCTGGCCAATTGATCTTGATATTCAGCACGctgaaattatttagttgttatgtattcagatgtttggaaa
AAGATCGAAAGTACCGTTCGAGGAGAAGCCGAGAGAAAGACCGGTTAACTAGAACTATAAGTCGTGCgactttaataaatcaacaatacataagtctacaaaccttt
atad-3
5,460 5,480 5,500 5,520 5,540 5,560

aattacCGAATGCGCATGTTTAGTCTCTTCTCCCAAAGTCTTCCTCCGTTCTTCTTCAGCAACACGAATGTGCTCTGATTTCATATTGGCCAATTGAGCTTCGATCT
ttaatgGCTTACGCGTACAAATCAGAGAAGAGGGTTTCAGAAGGAGGCAAGAAGAAGTCGTTGTGCTTACACGAGACTAAAGTATAACCGGTTAACTCGAAGCTAGA
atad-3
5,580 5,600 5,620 5,640 5,660

TCTTGGTCTCGTTCTCGACCTCTTTTTGGCGTGTAACTTCTTGCATTCTCGACAATTCAAGAGCTTCTTTGGCATTTGGAAATTTCTCTAGATCACGAGCAGCCTTG
AGAACCAGAGCAAGAGCTGGAGAAAAACCGCACATTGAAGAACGTAAGAGCTGTTAAGTTCTCGAAGAAACCGTAAACCTTTAAAGAGATCTAGTGCTCGTCGGAAC
atad-3
5,680 5,700 5,720 5,740 5,760

GCGGCACGCTCCAAAGCTGTTGAGTCGAAGGAATATGCCATTTTGCTGTTTCCCTCTTGCCGTTGGCCTTGACCTGGTTGTTGTGGACCGCCAGGAGCAGCACCTGC
CGCCGTGCGAGGTTTCGACAACTCAGCTTCCTTATACGGTAAAACGACAAAGGGAGAACGGCAACCGGAACTGGACCAACAACACCTGGCGGTCCTCGTCGTGGACG
atad-3
5,780 5,800 5,820 5,840 5,860 5,880

TTGAAAATCATCCGGAATCTGCGGAGTAGCGTTTTTTTGAACACCAAATAACCAAGACATctgaaagaatagattgaagcttgggatggtgcaaaaatattctaagg
AACTTTTAGTAGGCCTTAGACGCCTCATCGCAAAAAAACTTGTGGTTTATTGGTTCTGTAgactttcttatctaacttcgaaccctaccacgtttttataagattcc
atad-3 Patad-3
5,900 5,920 5,940 5,960 5,980

aatattgattaaaggaaatgatttatatatgtacttatgccaaagtatgaaacaataatttctttaaaggaatgaaattcggagagactttgtgtaatccttgaaga
ttataactaatttcctttactaaatatatacatgaatacggtttcatactttgttattaaagaaatttccttactttaagcctctctgaaacacattaggaacttct
Patad-3
6,000 6,020 6,040 6,060 6,080

aaacgtagtattaaaacagagttgcaaccttttcagtaaaattagatgatgcaaaacagattttccggctcattttccctgaatttaaaggacctgccgtcaaaaaa
tttgcatcataattttgtctcaacgttggaaaagtcattttaatctactacgttttgtctaaaaggccgagtaaaagggacttaaatttcctggacggcagtttttt
Patad-3
6,100 6,120 6,140 6,160 6,180 6,200

ATAD-3 (-ETAV) in pCFJ1178 (10767 bp) (from 6207-7062 bp)

gacctgccgtcgttataattcacgaatttctatttttgaaacacattctgcattgaaaaataaactttaaaaaattaaattaagattgttggaaaaaaacagaaat
ctggacggcagcaatattaagtgcttaaagataaaaactttgtgtaagacgtaacttttttatttgaaattttttaatttaattctaacaacctttttttgtcttta
Patad-3
6,220 6,240 6,260 6,280 6,300

cgctaaggcagattatgctccaatgacattttctgacgcatttctcgatatcgtaaagtgcgccagcgttcaaaggccacatctgaggccccacgaaaaggggagca
gcgattccgtctaatacgaggttactgtaaaagactgcgtaaagagctatagcatttcacgcggtcgcaagtttccggtgtagactccggggtgcttttcccctcgt
Patad-3
6,320 6,340 6,360 6,380 6,400 6,420

gaacgaaaaggggatctgcaaaaaggggatctgcgaaaaggggagatacggaaaggggagatacgaaaaggggagcaacgaaaaggggatctggcactgtgccaaac
cttgcttttcccctagacgtttttcccctagacgcttttcccctctatgcctttcccctctatgcttttcccctcgttgcttttcccctagaccgtgacacggtttg
Patad-3
6,440 6,460 6,480 6,500 6,520

gctatttttctcgaagaaaacgatacaacgattgtcgcgaatacgcagaatactgatgttccgccgatcacgactgagaaatgatattaatacccaggaaatgcgtc
cgataaaaagagcttcttttgctatgttgctaacagcgcttatgcgtcttatgactacaaggcggctagtgctgactctttactataattatgggtcctttacgcag
Patad-3
6,540 6,560 6,580 6,600 6,620

aaggaaatattaaagaattattatgattattacgaagtctttccgtattctaaatgctcacgacaatccaccgaagtcatcaacaacgacaaagccgggaaactcct
ttcctttataatttcttaataatactaataatgcttcagaaaggcataagatttacgagtgctgttaggtggcttcagtagttgttgctgtttcggccctttgagga
Patad-3
6,640 6,660 6,680 6,700 6,720 6,740

gtttgaaagccgcgccaaaagacacacacgtcacgagtgttacagtactaccacacatgcacatcaaaccccaacacgtcgacaccgcgcaaggaaatcgtcacttc
caaactttcggcgcggttttctgtgtgtgcagtgctcacaatgtcatgatggtgtgtacgtgtagtttggggttgtgcagctgtggcgcgttcctttagcagtgaag
Patad-3
6,760 6,780 6,800 6,820 6,840

ttcgttttactcccggaccggcaggtcgcatgttaaatatgagctttctcatttttatcattcaacggatttatcagtaataaattatataattctaccacttttga
aagcaaaatgagggcctggccgtccagcgtacaatttatactcgaaagagtaaaaatagtaagttgcctaaatagtcattatttaatatattaagatggtgaaaact
Patad-3
6,860 6,880 6,900 6,920 6,940

acctataccgatccgaaacatggtgcatcggctcCCCGGGCCtagttctagacattctctaatgaaaaaatctttcagttgaaattgaaaatgagttaaagttggag
tggatatggctaggctttgtaccacgtagccgagGGGCCCGGatcaagatctgtaagagattacttttttagaaagtcaactttaacttttactcaatttcaacctc
Patad-3
C. briggsae unc-119(+) region
6,960 6,980 7,000 7,020 7,040 7,060

ATAD-3 (-ETAV) in pCFJ1178 (10767 bp) (from 7063-7918 bp)

tttttattgaaaacagatttccgtgtgattagtgtttttagcgagtgtgacaggacagcgaaaaaatatagaaacaaggggggaactgaaaagcttaggaatgcatt
aaaaataacttttgtctaaaggcacactaatcacaaaaatcgctcacactgtcctgtcgcttttttatatctttgttccccccttgacttttcgaatccttacgtaa
C. briggsae unc-119(+) region
7,080 7,100 7,120 7,140 7,160

gaacatgagaaggggaaggggaaggaacaaactagacaggaattattggaatttaatcacatttggagtttttttctattcgacagaataattatccagaacattt
cttgtactcttccccttccccttccttgtttgatctgtccttaataaccttaaattagtgtaaacctcaaaaaaagataagctgtcttattaataggtcttgtaaa
C. briggsae unc-119(+) region
7,180 7,200 7,220 7,240 7,260

ttgtattaaatatttatgcatcatatgagtagtcggctttgttgtgcatgacgagtttgttatcgacgaaatagaagctgtcagaacgagtctcgtttggattgttg
aacataatttataaatacgtagtatactcatcagccgaaacaacacgtactgctcaaacaatagctgctttatcttcgacagtcttgctcagagcaaacctaacaac
C. briggsae unc-119(+) region
7,280 7,300 7,320 7,340 7,360 7,380

atcatgtcgtccactgaaaaagagattagtcttttgaattgtactttttagaataatgactcactgagttgttgagagagttgagggaactcatagatatgttcaca
tagtacagcaggtgactttttctctaatcagaaaacttaacatgaaaaatcttattactgagtgactcaacaactctctcaactcccttgagtatctatacaagtgt
C. briggsae unc-119(+) region
7,400 7,420 7,440 7,460 7,480

gttgtttcgtgaattcggaatacagaatccgaattcaaagtcaaaacacttcagaaggcgatctttgaagaagtgacgttcgatcattcggaaatgatggattggga
caacaaagcacttaagccttatgtcttaggcttaagtttcagttttgtgaagtcttccgctagaaacttcttcactgcaagctagtaagcctttactacctaaccct
C. briggsae unc-119(+) region
7,500 7,520 7,540 7,560 7,580

tgtctcctactttgaattccacagttgctccgaccgttttgagtttcaaaaagtttggagcaaatctataacgcacgtatcttgccgactcctgtggcgactcatca
acagaggatgaaacttaaggtgtcaacgaggctggcaaaactcaaagtttttcaaacctcgtttagatattgcgtgcatagaacggctgaggacaccgctgagtagt
C. briggsae unc-119(+) region
7,600 7,620 7,640 7,660 7,680 7,700

ttctcctgatcgttctccggtttggcgatctcgaaaagcacttgctcagtgtccagatcacggatttggaacttggtgaactcgatgttatagatgttcgcagatgg
aagaggactagcaagaggccaaaccgctagagcttttcgtgaacgagtcacaggtctagtgcctaaaccttgaaccacttgagctacaatatctacaagcgtctacc
C. briggsae unc-119(+) region
7,720 7,740 7,760 7,780 7,800

ggagcataagaatcctaaatttatgttttaaactgaaatccaaagggagcaagataccttgagtgattcccggaagtgctaaaacgtcgttcggagtgatttgagct
cctcgtattcttaggatttaaatacaaaatttgactttaggtttccctcgttctatggaactcactaagggccttcacgattttgcagcaagcctcactaaactcga
C. briggsae unc-119(+) region
7,820 7,840 7,860 7,880 7,900

ATAD-3 (-ETAV) in pCFJ1178 (10767 bp) (from 7919-8774 bp)

ttcttcgcaagctccgattccgttgtgattccttgttcggtgcttggtggtggccgtggcatctggaaatatggaaaagttcaacaaaaagaaaagagaaaagaatg
aagaagcgttcgaggctaaggcaacactaaggaacaagccacgaaccaccaccggcaccgtagacctttataccttttcaagttgtttttcttttctcttttcttac
C. briggsae unc-119(+) region
7,920 7,940 7,960 7,980 8,000 8,020

aaatcggatatcaagagttagttgagcggtttctctagttttctgagtctcacctgcgacgggaaggtcgccgagccgggtggaatcgatCgttgttgctcggcttt
tttagcctatagttctcaatcaactcgccaaagagatcaaaagactcagagtggacgctgcccttccagcggctcggcccaccttagctaGcaacaacgagccgaaa
C. briggsae unc-119(+) region
8,040 8,060 8,080 8,100 8,120

catatcggtttggttggaagcggctgaaaacggaaagaagtggaagaaggaaaagagtgtggtgtgacaggaaaatggtaattagagggtgccaaataaccagctat
gtatagccaaaccaaccttcgccgacttttgcctttcttcaccttcttccttttctcacaccacactgtccttttaccattaatctcccacggtttattggtcgata
C. briggsae unc-119(+) region
8,140 8,160 8,180 8,200 8,220

attttgttttttttgaaaacatttttaaaaagaaaaatacgataatgatatcagatggatttccggaaaactggtatgaaaaatttcaacctttttgagtacatgt
taaaacaaaaaaaaacttttgtaaaaatttttctttttatgctattactatagtctacctaaaggcctttttgaccatactttttaaagttggaaaaactcatgtaca
C. briggsae unc-119(+) region
8,240 8,260 8,280 8,300 8,320 8,340

aatcaaaatacactttgtaaattatcatttttattgaaactccaccatttttctatttataacgctaataatttgaaaaagaaacctAttgcgaaccgcggggtgaa
ttagttttatgtgaaacatttaatagtaaaaataactttgaggtggtaaaaagataaatattgcgattattaaactttttctttggaTaacgcttggcgccccactt
C. briggsae unc-119(+) region
8,360 8,380 8,400 8,420 8,440

tcccaaaaacgaatgcgttttggtggagtgattgattcgaatcgaagaagaaaaagaagaagacgtggaatagagagctcactcttaaccgagcagcacacaccgac
agggtttttgcttacgcaaaaccacctcactaactaagcttagcttcttctttttcttcttctgcaccttatctctcgagtgagaattggctcgtcgtgtgtggctg
C. briggsae unc-119(+) region
8,460 8,480 8,500 8,520 8,540 8,560

agaaaaaaaaatgaaatgaatgagggtcttcttcttcttcttcttcgaatgattgacagaaatgggaaaaagaggaagattgagaagggaaaaaggaaggagaaaag
tcttttttttttactttacttactcccagaagaagaagaagaagaagcttactaactgtctttacccttttttctccttctaactcttccctttttccttcctcttttc
C. briggsae unc-119(+) region
8,580 8,600 8,620 8,640 8,660

aagcagaagaagacgtcagagaggagaggaacgagcggaaaagcagcgggcgcaagtcatagaagtagcagagctggggagaagaagacactatccaagaaaggaat
ttcgtcttcttctgcagtctctcctctccttgctcgccttttcgtcgcccgcgttcagtatcttcatcgtctcgacccctcttcttctgtgataggttctttcctta
C. briggsae unc-119(+) region
8,680 8,700 8,720 8,740 8,760

ATAD-3 (-ETAV) in pCFJ1178 (10767 bp) (from 8775-9630 bp)

gacgagagagtatgcaaaggggtatagggtgcagacagaataggaacagaataacagatgatgagccaagaagagttgaaaagggcgatgaatttgtcatgtaactt
ctgctctctcatacgtttccccatatcccacgtctgtcttatccttgtcttattgtctactactcggttcttctcaacttttcccgctacttaaacagtacattgaa

C. briggsae unc-119(+) region

8,780 8,800 8,820 8,840 8,860 8,880

aatttgggtcaatttgagcatgatgaattgaaatcatcccttgttgggagttaataaccggtttgttatcagaaaccctgtaatagaagggcgccctaactttgagc
ttaaacccagttaaactcgtactacttaactttagtagggaacaaccctcaattattggccaaacaatagtctttgggacattatcttcccgcgggattgaaactcg

C. briggsae unc-119(+) region

8,900 8,920 8,940 8,960 8,980

caattcatcccggtttctgtcaaatatatcaaaaagtggtcaactgacaaattgtttttgatattataataaacattttatccgttaacaattttcgaatacttttt
gttaagtagggccaaagacagtttatatagtttttcaccagttgactgtttaacaaaaactataatattatttgtaaaataggcaattgttaaaagcttatgaaaaa

C. briggsae unc-119(+) region

9,000 9,020 9,040 9,060 9,080

acaaggacttggataaattggctcaaagagcctgcttttttgtacaaacttgaattcaaaaacgcagaactcgtactgttggatgcctgtgtagcggtcctctattg
tgttcctgaacctatttaaccgagtttctcggacgaaaaaacatgtttgaacttaagtttttgcgtcttgagcatgacaacctacggacacatcgccaggagataac

left recom 10882

9,100 9,120 9,140 9,160 9,180 9,200

atttttgctattttttgataattttactgtaaattatgagttattccaattaacattcctagctaaatgtaagttagcgaccaattttttagcaaccccattttatg
taaaaacgataaaaaactattaaaatgacatttaatactcaataaggttaattgtaaggatcgatttacattcaatcgctggttaaaaaatcgttggggtaaaatac

left recom 10882

9,220 9,240 9,260 9,280 9,300

acttttcagaatatcgcctactgggtcaacgacacttgtttggaactaagggtcttcccaattttttttaaatcttcataggcttgacagaatcagggttcttcaag
tgaaaagtcttatagcggatgacccagttgctgtgaacaaaccttgattcccagaagggttaaaaaaaatttagaagtatccgaactgtcttagtcccaagaagttc

left recom 10882

9,320 9,340 9,360 9,380 9,400

cagctttgagagggaatcaattgcaacatttgatgttaaatttatattttttatagttctctggtgaaatggggaccaccgcaaagccccttttatgtttttaactt
gtcgaaactctcccttagttaacgttgtaaactacaatttaaatataaaaaatatcaagagaccactttacccctggtggcgtttcggggaaaatacaaaaattgaa

left recom 10882

9,420 9,440 9,460 9,480 9,500 9,520

gaataaaatcttgtattaaattgtgatttatagatctgtatgggtaatttgaaggaaaagaaagggagctcccaaaagtgccttacagatccaggcccaataaatgt
cttattttagaacataatttaacactaaatatctagacatacccattaaacttccttttctttccctcgagggttttcacggaatgtctaggtccgggttatttaca

left recom 10882

9,540 9,560 9,580 9,600 9,620

ATAD-3 (-ETAV) in pCFJ1178 (10767 bp) (from 9631-10486 bp)

aaactattgggcagtagtatggcaaaaatcgtgcggaatttgaaaaacctagaattaaaataaatattcaacacaaatgttcgagatattttaagttcggagtgtat
tttgataacccgtcatcataccgtttttagcacgccttaaactttttggatcttaattttatttataagttgtgtttacaagctctataaaattcaagcctcacata
left recom 10882
9,640 9,660 9,680 9,700 9,720

atctagaacatattgtacaagtaggtctacgggatcgtctacgggaaaatttgatttcctcgaagatgtatttaatgaattctctaatgttgcaggtctttgtattt
tagatcttgtataacatgttcatccagatgccctagcagatgcccttttaaactaaaggagcttctacataaattacttaagagattacaacgtccagaaacataaa
left recom 10882
9,740 9,760 9,780 9,800 9,820 9,840

gacagaatgttggtttttgaaacttattcgccataattgcttggctactacatgtctcatgccattgacactatcctgtactcgaaatttgttaagtaactacccac
ctgtcttacaaccaaaaactttgaataagcggtattaacgaaccgatgatgtacagagtacggtaactgtgataggacatgagctttaaacaattcattgatgggtg
left recom 10882
9,860 9,880 9,900 9,920 9,940

cgtccacttatgacgagagggtcaattaaggtcactccacaaaagtaaatttgaaaacacatgagaaaaagtgatctgaattcccttgaagtggctaaaacaaagca
gcaggtgaatactgctctcccagttaattccagtgaggtgttttcatttaaacttttgtgtactctttttcactagacttaagggaacttcaccgattttgtttcgt
left recom 10882
9,960 9,980 10,000 10,020 10,040

gatgagggatacgacgtatgcgactactgagagataaggtcgaaacttgataaggatgctgcttacctgcttagcaagtttcttcgcacaggagaaagtgacaagct
ctactccctatgctgcatacgctgatgactctctattccagctttgaactattcctacgacgaatggacgaatcgttcaaagaagcgtgtcctctttcactgttcga
left recom 10882
10,060 10,080 10,100 10,120 10,140 10,160

aaactatcaaattcatctaaaacctcaaaaaattattttggatgaaataatgttttattttgagcaacgcataaggcaaagaataaattaaaggcgatccgcttagc
tttgatagtttaagtagattttggagttttttaataaaacctactttattacaaaataaaactcgttgcgtattccgtttcttatttaatttccgctaggcgaatcg
left recom 10882
10,180 10,200 10,220 10,240 10,260

ggcgagttccgtcctcgaagaagattcctccatcgatggcgcagtactttggacagattccacgctctcctggggttcctggtggtccgactggtcctggctgtcct
ccgctcaaggcaggagcttcttctaaggaggtagctaccgcgtcatgaaacctgtctaaggtgcgagaggaccccaaggaccaccaggctgaccaggaccgacagga
left recom 10882
10,280 10,300 10,320 10,340 10,360

gggtttccgtcggctcctggcttgccgtctggtcctggtggtccctttggtccaggttgtcctgaaagatgtggtgattagatatggattgaaaaactatgaaatga
cccaaaggcagccgaggaccgaacggcagaccaggaccaccagggaaaccaggtccaacaggactttctacaccactaatctatacctaactttttgatactttact
left recom 10882
10,400 10,420 10,440 10,460 10,480

ATAD-3 (-ETAV) in pCFJ1178 (10767 bp) (from 10487-10767 bp)

ttacctggggttccgtcgtttcctggttgtccggatggtcctggtggtccgactggtcctggctctccggtggatcctggctctcccttttggactggctcgcagac
aatggaccccaaggcagcaaaggaccaacaggcctaccaggaccaccaggctgaccaggaccgagaggccacctaggaccgagagggaaaacctgaccgagcgtctg

left recom 10882

10,500 10,520 10,540 10,560 10,580

agctggaagtccctccaactttctatacaaagttgatagcttggcgtaatcatggtcatagctgtttcctgtgtgaaattgttatccgctggtatcagctcactcaa
tcgaccttcagggaggttgaaagatatgtttcaactatcgaaccgcattagtaccagtatcgacaaaggacacactttaacaataggcgaccatagtcgagtgagtt

M13-rev

10,600 10,620 10,640 10,660 10,680 10,700

aggcggtaatacggttatccacagaatcaggggataacgcaggaaagaacatgtgagcaaaaggcca
tccgccattatgccaataggtgtcttagtcccctattgcgtcctttcttgtacactcgttttccggt

10,710 10,720 10,730 10,740 10,750 10,760

ATAD-3 in pCFJ1178 (10862 bp)

gcaaaagaccaggaaccgtaaaaaggccgcgttgctggcgtttttccataggctccgcccccctgacgagcatcacaaaaatcgacgctcaagtcagaggtggcgaa
cgttttctggtccttggcatttttccggcgcaacgaccgcaaaaaggtatccgaggcggggggactgctcgtagtgtttttagctgcgagttcagtctccaccgctt

ColE1 origin

20 40 60 80 100

acccgacaggactataaagataccaggcgtttccccctggaagctccctcgtgcgctctcctgttccgaccctgccgcttaccggatacctgtccgcctttctccct
tgggctgtcctgatatttctatggtccgcaaagggggaccttcgagggagcacgcgagaggacaaggctgggacggcgaatggcctatggacaggcggaaagaggga

ColE1 origin

120 140 160 180 200

tcgggaagcgtggcgctttctcatagctcacgctgtaggtatctcagttcggtgtaggtcgttcgctccaagctgggctgtgtgcacgaaccccccgttcagcccga
agcccttcgcaccgcgaaagagtatcgagtgcgacatccatagagtcaagccacatccagcaagcgaggttcgacccgacacacgtgcttggggggcaagtcgggct

ColE1 origin

220 240 260 280 300 320

ccgctgcgccttatccggtaactatcgtcttgagtccaacccggtaagacacgacttatcgccactggcagcagccactggtaacaggattagcagagcgaggtatg
ggcgacgcggaataggccattgatagcagaactcaggttgggccattctgtgctgaatagcggtgaccgtcgtcggtgaccattgtcctaatcgtctcgctccatac

ColE1 origin

340 360 380 400 420

taggcggtgctacagagttcttgaagtggtggcctaactacggctacactagaaggacagtatttggtatctgcgctctgctgaagccagttaccttcggaaaaaga
atccgccacgatgtctcaagaacttcaccaccggattgatgccgatgtgatcttcctgtcataaaccatagacgcgagacgacttcggtcaatggaagcctttttct

ColE1 origin

440 460 480 500 520

gttggtagctcttgatccggcaaacaaaccaccgctggtagcggtggtttttttgtttgcaagcagcagattacgcgcagaaaaaaaggatctcaagaagatccttt
caaccatcgagaactaggccgtttgtttggtggcgaccatcgccaccaaaaaaacaaacgttcgtcgtctaatgcgcgtctttttttcctagagttcttctaggaaa

ColE1 origin

540 560 580 600 620 640

gatcttttctacggggtctgacgctcagtggaacgaaaactcacgttaagggattttggtcatgagattatcaaaaaggatcttcacctagatccttttaaattaaa
ctagaaaagatgccccagactgcgagtcaccttgcttttgagtgcaattccctaaaaccagtactctaatagtttttcctagaagtggatctaggaaaatttaattt

ColE1 origin

660 680 700 720 740

aatgaagttttaaatcaatctaaagtatatatgagtaaacttggtctgacagttaccaatgcttaatcagtgaggcacctatctcagcgatctgtctatttcgttca
ttacttcaaaatttagttagatttcatatatactcatttgaaccagactgtcaatggttacgaattagtcactccgtggatagagtcgctagacagataaagcaagt

AmpR

760 780 800 820 840

ATAD-3 in pCFJ1178 (10862 bp) (from 857-1819 bp)

tccatagttgcctgactccccgtcgtgtagataactacgatacgggagggcttaccatctggccccagtgctgcaatgataccgcgagacccacgctcaccggctcc
aggtatcaacggactgaggggcagcacatctattgatgctatgccctcccgaatggtagaccggggtcacgacgttactatggcgctctgggtgcgagtggccgagg
AmpR
860 880 900 920 940 960

agatttatcagcaataaaccagccagccggaagggccgagcgcagaagtggtcctgcaactttatccgcctccatccagtctattaattgttgccgggaagctagag
tctaaatagtcgttatttggtcggtcggccttcccggctcgcgtcttcaccaggacgttgaaataggcggaggtaggtcagataattaacaacggcccttcgatctc
AmpR
980 1,000 1,020 1,040 1,060

taagtagttcgccagttaatagtttgcgcaacgttgttgccattgctacaggcatcgtggtgtcacgctcgtcgtttggtatggcttcattcagctccggttcccaa
attcatcaagcggtcaattatcaaacgcgttgcaacaacggtaacgatgtccgtagcaccacagtgcgagcagcaaaccataccgaagtaagtcgaggccaagggtt
AmpR
1,080 1,100 1,120 1,140 1,160

cgatcaaggcgagttacatgatcccccatgttgtgcaaaaaagcggttagctccttcggtcctccgatcgttgtcagaagtaagttggccgcagtgttatcactcat
gctagttccgctcaatgtactagggggtacaacacgttttttcgccaatcgaggaagccaggaggctagcaacagtcttcattcaaccggcgtcacaatagtgagta
AmpR
1,180 1,200 1,220 1,240 1,260 1,280

ggttatggcagcactgcataattctcttactgtcatgccatccgtaagatgcttttctgtgactggtgagtactcaaccaagtcattctgagaatagtgtatgcggc
ccaataccgtcgtgacgtattaagagaatgacagtacggtaggcattctacgaaaagacactgaccactcatgagttggttcagtaagactcttatcacatacgccg
AmpR
1,300 1,320 1,340 1,360 1,380

gaccgagttgctcttgcccggcgtcaatacgggataataccgcgccacatagcagaactttaaaagtgctcatcattggaaaacgttcttcggggcgaaaactctca
ctggctcaacgagaacgggccgcagttatgccctattatggcgcggtgtatcgtcttgaaattttcacgagtagtaaccttttgcaagaagccccgcttttgagagt
AmpR
1,400 1,420 1,440 1,460 1,480

aggatcttaccgctgttgagatccagttcgatgtaacccactcgtgcacccaactgatcttcagcatcttttactttcaccagcgtttctgggtgagcaaaaacagg
tcctagaatggcgacaactctaggtcaagctacattgggtgagcacgtgggttgactagaagtcgtagaaaatgaaagtggtcgcaaagacccactcgtttttgtcc
1,500 1,520 1,540 1,560 1,580 1,600

aaggcaaaatgccgcaaaaaagggaataagggcgacacggaaatgttgaatactcatactcttcctttttcaatattattgaagcatttatcagggttattgtctca
ttccgttttacggcgttttttcccttattcccgctgtgcctttacaacttatgagtatgagaaggaaaaagttataataacttcgtaaatagtcccaataacagagt
1,620 1,640 1,660 1,680 1,700

tgagcggatacatatttgaatgtatttagaaaaataaacaaataggggttccgcgcacatttccccgaaaagtgccacctgacgtctaagaaaccattattatcatg
actcgcctatgtataaacttacataaatctttttatttgtttatccccaaggcgcgtgtaaaggggcttttcacggtggactgcagattctttggtaataatagtac
1,720 1,740 1,760 1,780 1,800

ATAD-3 in pCFJ1178 (10862 bp) (from 1820-2782 bp)

acattaacctataaaaataggcgtatcacgaggccctttcgtctcgcgcgtttcggtgatgacggtgaaaacctctgacacatgcagctcccggagacggtcacagc
tgtaattggatatttttatccgcatagtgctccgggaaagcagagcgcgcaaagccactactgccacttttggagactgtgtacgtcgagggcctctgccagtgtcg

1,820 1,840 1,860 1,880 1,900 1,920

ttgtctgtaagcggatgccgggagcagacaagcccgtcagggcgcgtcagcgggtgttggcgggtgtcggggctggcttaactatgcggcatcagagcagattgtac
aacagacattcgcctacggccctcgtctgttcgggcagtcccgcgcagtcgcccacaaccgcccacagccccgaccgaattgatacgccgtagtctcgtctaacatg

1,940 1,960 1,980 2,000 2,020

tgagagtgcaccatatgcggtgtgaaataccgcacagatgcgtaaggagaaaataccgcatcaggcgccattcgccattcaggctgcgcaactgttgggaagggcga
actctcacgtggtatacgccacactttatggcgtgtctacgcattcctcttttatggcgtagtccgcggtaagcggtaagtccgacgcgttgacaacccttcccgct

LacZ alpha

2,040 2,060 2,080 2,100 2,120 2,140

tcggtgcgggcctcttcgctattacgccagctggcgaaagggggatgtgctgcaaggcgattaagttgggtaacgccagggttttcccagtcacgacgttgtaaaac
agccacgcccggagaagcgataatgcggtcgaccgctttccccctacacgacgttccgctaattcaacccattgcggtcccaaaagggtcagtgctgcaacattttg

LacZ alpha

2,160 2,180 2,200 2,220 2,240

gacggccagtgaattatcaactatgtataataaagttgcggcccattaggtcagacaaaaatttctgatctttaaaccttattgattaaaactaatttttgcccgac
ctgccggtcacttaatagttgatacatattatttcaacgccgggtaatccagtctgtttttaaagactagaaatttggaataactaatttttgattaaaaacgggctg

right recomb 10882

2,260 2,280 2,300 2,320 2,340

tttttgacacatgcactagacgcttgtactcaaaatttggaaaccttgctcaaatccactactcagatatgacagtagcatcgataattgaaccattatcctgaaa
aaaaaactgtgtacgtgatctgcgaacatgagttttaaacctttggaacgagtttaggtgatgagtctatactgtcatcgtagctattaacttggtaataggacttt

right recomb 10882

2,360 2,380 2,400 2,420 2,440 2,460

tccccttttcctgtcccggttcattttccaatttttcaacggtggaagaaggggcaaattttataccaaaacgtctccgtttatgacccacccccctatgcaaaataa
aggggaaaggacagggccaagtaaaaggttaaaaagttgccaccttcttccccgtttaaaatatggttttgcagaggcaaatactgggtgggggatacgttttatt

right recomb 10882

2,480 2,500 2,520 2,540 2,560

acggctctgggagaaatagtacagaaaacgcggagcctgcttgattccttctccgaggcgacaaaaaacgcatcattcgtattgtaagatgcatgtgtggctggcct
tgccgagaccctctttatcatgtcttttgcgcctcggacgaactaaggaagaggctccgctgttttttgcgtagtaagcataacattctacgtacacaccgaccgga

right recomb 10882

2,580 2,600 2,620 2,640 2,660

cttttgaattttactggtttttttatatttctactaatatttcgaaaaattttgtaagtaaatcattgattacgtttacaaaaatttagaaacatttcaaaatgatta
gaaaacttaaaatgaccaaaaaaatataaagatgattataaagctttttaaaacattcatttagtaactaatgcaaatgtttttaaatctttgtaaagttttactaat

right recomb 10882

2,680 2,700 2,720 2,740 2,760 2,780

ATAD-3 in pCFJ1178 (10862 bp) (from 2783-3638 bp)

cttctaaattaattatatttacaggttagttcgagttacttatgaccttccaatccgccatatccgccaaggacgctccaattaatttttttagaattattttttaac
gaagatttaattaatataaatgtccaatcaagctcaatgaatactggaaggttaggcggtataggcggttcctgcgaggttaattaaaaaatcttaataaaaaattg
right recomb 10882
2,800 2,820 2,840 2,860 2,880

acatgtattattttttaacaataaattattcatgaaatcatcaaatgtatatgttctactataatatttaggtcaaactatagaaaagaaatccttgtttttggaac
tgtacataataaaaaattgttatttaataagtactttagtagtttacatatacaagatgatattataaatccagtttgatatcttttctttaggaacaaaaaccttg
right recomb 10882
2,900 2,920 2,940 2,960 2,980

aaaggagttcagatcctgtgcatttgatcttggatccacatctttggtccatgggaaagcttggcactgatcaagaaggattcttcaaatatctaaaattgccggct
tttcctcaagtctaggacacgtaaactagaacctaggtgtagaaaccaggtaccctttcgaaccgtgactagttcttcctaagaagtttatagattttaacggccga
right recomb 10882
3,000 3,020 3,040 3,060 3,080 3,100

gcccgtagaattccatcagttttccagagttttagtgaaaatttgcatcccgttagaaacaatctacacaacggtaaaaaggacccattatttttaccgtaccgttt
cgggcatcttaaggtagtcaaaaggtctcaaaatcacttttaaacgtagggcaatctttgttagatgtgttgccatttttcctgggtaataaaaatggcatggcaaa
right recomb 10882
3,120 3,140 3,160 3,180 3,200

tcctttatggccctctatgcaaaaaattatagcgtggaggggcagtacagaataataaagtgtgagaaaaaaaactgctctgcgagaaatagtacagcaaacgcgga
aggaaataccgggagatacgttttttaatatcgcacctccccgtcatgtcttattatttcacactcttttttttgacgagacgctctttatcatgtcgtttgcgcct
right recomb 10882
3,220 3,240 3,260 3,280 3,300

gcatgcttgattctatccactttgtacaagaaagctgggttaatacgactcactagtgggcagatcttcgaatgcatcGcgcgcaccgtacgtctcgaggaattcct
cgtacgaactaagataggtgaaacatgttctttcgacccaattatgctgagtgatcacccgtctagaagcttacgtagCgcgcgtggcatgcagagctccttaagga
3,320 3,340 3,360 3,380 3,400 3,420

gcaggatatctggatccacgaagcttcccatggtgacgtcaccggttctagatacctaggtgagctctggtaccggatgcacggaagattctgaaattattgttttt
cgtcctatagacctaggtgcttcgaagggtaccactgcagtggccaagatctatggatccactcgagaccatggcctacgtgccttctaagactttaataacaaaaa
atad-3 UTR
3,440 3,460 3,480 3,500 3,520

caggatttctgatattgaaaattgtggtttctagctttttattgttgcttttctggaaatattaggctaaaagctcataaacgtttaatttcaattgatttcttatt
gtcctaaagactataacttttaacaccaaagatcgaaaaataacaacgaaaagacctttataatccgattttcgagtatttgcaaattaaagttaactaaagaataa
atad-3 UTR
3,540 3,560 3,580 3,600 3,620

ATAD-3 in pCFJ1178 (10862 bp) (from 3639-4494 bp)

ttctgaaaaaaccatatattgccagctttttccttcttacctgtgcataaacagaaagacctctccggcctcccgcaagttttttgtttcagtatccatttgatcgt
aagactttttggtatataacggtcgaaaaaggaagaatggacacgtatttgtctttctggagaggccggagggcgttcaaaaaacaaagtcataggtaaactagca

atad-3 UTR

3,640 3,660 3,680 3,700 3,720 3,740

tttaatcgaacatttgggctgaaaattattttttttgctttttctcttcagaagctttcaaaatatgaaaaattgaagtgcaacaaaagcgattattagttagtatt
aaattagcttgtaaacccgacttttaataaaaaaaacgaaaaagagaagtcttcgaaagttttatactttttaacttcacgttgttttcgctaataatcaatcataa

atad-3 UTR

3,760 3,780 3,800 3,820 3,840

acattaaaatgaagagtttctttattaacaaaaaaaaaacaataatctttcactctacttaaattactacacaaaaactaaataaaaatgactgacctagaaaataa
tgtaattttacttctcaaagaaataattgttttttttttgttattagaaagtgagatgaatttaatgatgtgtttttgatttatttttactgactggatcttttatt

atad-3 UTR

3,860 3,880 3,900 3,920 3,940

ggggaaaacgaaaaaaaacaatagaaaaacaagggcctaattgaattatctaaaaaaattatattatcagcaaaatttctgattaagaatgaaattacgaaattacc
ccccttttgcttttttttgttatctttttgttcccggattaacttaatagatttttttaatataatagtcgttttaaagactaattcttactttaatgctttaatgg

atad-3 UTR

3,960 3,980 4,000 4,020 4,040 4,060

ggaagtttgaagatttcagaaaagaaaaaacaacaaaaaaacgtaaatagaaaacaatagagtatcatgaacaaggaaatgaatattttgaaaTTAAACAGCAGTTT
ccttcaaacttctaaagtcttttcttttttgttgtttttttgcatttatcttttgttatctcatagtacttgttcctttacttataaaactttAATTTGTCGTCAAA

atad-3 UTR | atad-3

4,080 4,100 4,120 4,140 4,160

CTCTCTTCAACGTAGTTCCGAACTTGACTTCTTGGTTACGAGCTTTCAATTGCTCCTTCTCAAGCCATTCCATCTTATGTTCATGTTGAACCATTGCGTCAGCAGTA
GAGAGAAGTTGCATCAAGGCTTGAACTGAAGAACCAATGCTCGAAAGTTAACGAGGAAGAGTTCGGTAAGGTAGAATACAAGTACAACTTGGTAACGCAGTCGTCAT

atad-3

4,180 4,200 4,220 4,240 4,260 4,280

TTACGATCAACGATAGCCTCGGTGAGCACTCCGGTTTCAGATGCATATGCAGAAGCTTGCCATCCGATGACAAGTTTACTCAATTCTCTTCCACTCATTCCAGATGT
AATGCTAGTTGCTATCGGAGCCACTCGTGAGGCCAAAGTCTACGTATACGTCTTCGAACGGTAGGCTACTGTTCAAATGAGTTAAGAGAAGGTGAGTAAGGTCTACA

atad-3

4,300 4,320 4,340 4,360 4,380

CTTTTTGGCAACTTCATTACACTTAGCAACCCAATCAAAGTTATCGAGTTTCAGTCGTTGAGAACGAGATCCACTAGTTGCTGGAGTGACGATGTGCTCGTTGAAGT
GAAAAACCGTTGAAGTAATGTGAATCGTTGGGTTAGTTTCAATAGCTCAAAGTCAGCAACTCTTGCTCTAGGTGATCAACGACCTCACTGCTACACGAGCAACTTCA

atad-3

4,400 4,420 4,440 4,460 4,480

ATAD-3 in pCFJ1178 (10862 bp) (from 4495-5350 bp)

ATTGCAGAAGAATTCGTTCACGTTCTTCCATTCCTGGCAGTGTGAATTCGACAAGCTGATCGAAActgaaaagaataataaattctgtttttttatttaaaaaaaagg
TAACGTCTTCTTAAGCAAGTGCAAGAAGGTAAGGACCGTCACACTTAAGCTGTTCGACTAGCTTTgacttttcttattatttaagacaaaaaaataaattttttttcc

atad-3

4,500 4,520 4,540 4,560 4,580 4,600

attacCGATCGTTAACTGCCCAATCGAACTGCTCTGGCTGATTAGAAGCCACGACAAGCATGAACTTTCTCGACTGCTCTCCAGTTCTGAAGAGGAAAGCGTTGAGA
taatgGCTAGCAATTGACGGGTTAGCTTGACGAGACCGACTAATCTTCGGTGCTGTTCGTACTTGAAAGAGCTGACGAGAGGTCAAGACTTCTCCTTTCGCAACTCT

atad-3

4,620 4,640 4,660 4,680 4,700

GCTGCACGTGTGTCTTCAGACATTCCATTCTTTGATCTCTTTTGCAAAAAAGCGTCAGCTTCGTCAATAAAAACAATCAACCCTTTGCGACTTTTTGAAGCCCAGTC
CGACGTGCACACAGAAGTCTGTAAGGTAAGAAACTAGAGAAAACGTTTTTTCGCAGTCGAAGCAGTTATTTTTGTTAGTTGGGAAACGCTGAAAAACTTCGGGTCAG

atad-3

4,720 4,740 4,760 4,780 4,800

GAATACTTTATGAATTGCAGATACTCCATCACGTCCAAGTGGAGCAATATCTCCTCCAGTGAGAACTGCGTAATCGAGTCCGGAATGTTGAGCCAAACTCTTTGCGA
CTTATGAAATACTTAACGTCTATGAGGTAGTGCAGGTTCACCTCGTTATAGAGGAGGTCACTCTTGACGCATTAGCTCAGGCCTTACAACTCGGTTTGAGAAACGCT

atad-3

4,820 4,840 4,860 4,880 4,900 4,920

AAAGTGTCTTTCCGGTTCCTGGTGGTCCGTAGAACATTACATTACGGAAAAGTCCATTATTTCTCTTTGTGTTTGAGGTGGTAATGGCAATATCACGAAGACGACGT
TTTCACAGAAAGGCCAAGGACCACCAGGCATCTTGTAATGTAATGCCTTTTCAGGTAATAAAGAGAAACACAAACTCCACCATTACCGTTATAGTGCTTCTGCTGCA

atad-3

4,940 4,960 4,980 5,000 5,020

TCCAAAGCAGGTGGTAATACAACTCCATTCAGTGGATCCTTCTTCTGACGAGTCATCATTTGAACACTTTTAATTGGGTGTTTGAGTACTTCAAGTGGAGTGATTCT
AGGTTTCGTCCACCATTATGTTGAGGTAAGTCACCTAGGAAGAAGACTGCTCAGTAGTAAACTTGTGAAAATTAACCCACAAACTCATGAAGTTCACCTCACTAAGA

atad-3

5,040 5,060 5,080 5,100 5,120

GGATGTTTCTCTTACAAGACTTGGTTTTCCGAGACGAGATTCGATGTATCTTGCGGTGACTCCAGTTCCTCGTTTAGCGGTATACCAGCCGACAGCCAAAGCAGTCA
CCTACAAAGAGAATGTTCTGAACCAAAAGGCTCTGCTCTAAGCTACATAGAACGCCACTGAGGTCAAGGAGCAAATCGCCATATGGTCGGCTGTCGGTTTCGTCAGT

atad-3

5,140 5,160 5,180 5,200 5,220 5,240

ATCCTCCTACTGCAGCAGCGATTTTCGTTTTATCATTCAAAAATTGATTGAGTCCAGAACCGATAAGTTCTCCACTGGTTTTAATTTTTTCAATGACAGTTTTTCGG
TAGGAGGATGACGTCGTCGCTAAAAGCAAAATAGTAAGTTTTTAACTAACTCAGGTCTTGGCTATTCAAGAGGTGACCAAAATTAAAAAAGTTACTGTCAAAAAGCC

atad-3

5,260 5,280 5,300 5,320 5,340

ATAD-3 in pCFJ1178 (10862 bp) (from 5351-6206 bp)

```
TTCTCTTCTTCGTGAAGCTTCATTTGCTCTAAGTTTACATCTCTGTTATCTCGAGCAGCCTTTGCCCTGGCTCTGGTCTCTGCGTCAATTTTCTCGAGCTCGTATTT
AAGAGAAGAAGCACTTCGAAGTAAACGAGATTCAAATGTAGAGACAATAGAGCTCGTCGGAAACGGGACCGAGACCAGAGACGCAGTTAAAAGAGCTCGAGCATAAA
atad-3
5,360   5,380   5,400   5,420   5,440

GTGTTTTAATGCTAACTCATGCTCAATAGTTTGCTTGCGAAGTTGTTCCTGCTTCTTGACAGACTCTTCTTGTTTTCGGAGGCTTTCTTCCTGCATTCTAGCTTTCA
CACAAAATTACGATTGAGTACGAGTTATCAAACGAACGCTTCAACAAGGACGAAGAACTGTCTGAGAAGAACAAAAGCCTCCGAAAGAAGGACGTAAGATCGAAAGT
atad-3
5,460   5,480   5,500   5,520   5,540   5,560

TGGCAAGCTCCTCTTCGGCTCTCTTTCTGGCCAATTGATCTTGATATTCAGCACGctgaaattatttagttgttatgtattcagatgtttggaaaaattacCGAATG
ACCGTTCGAGGAGAAGCCGAGAGAAAGACCGGTTAACTAGAACTATAAGTCGTGCgactttaataaatcaacaatacataagtctacaaacctttttaatgGCTTAC
atad-3
5,580   5,600   5,620   5,640   5,660

CGCATGTTTAGTCTCTTCTCCCAAAGTCTTCCTCCGTTCTTCTTCAGCAACACGAATGTGCTCTGATTTCATATTGGCCAATTGAGCTTCGATCTTCTTGGTCTCGT
GCGTACAAATCAGAGAAGAGGGTTTCAGAAGGAGGCAAGAAGAAGTCGTTGTGCTTACACGAGACTAAAGTATAACCGGTTAACTCGAAGCTAGAAGAACCAGAGCA
atad-3
5,680   5,700   5,720   5,740   5,760

TCTCGACCTCTTTTTGGCGTGTAACTTCTTGCATTCTCGACAATTCAAGAGCTTCTTTGGCATTTGGAAATTTCTCTAGATCACGAGCAGCCTTGGCGGCACGCTCC
AGAGCTGGAGAAAAACCGCACATTGAAGAACGTAAGAGCTGTTAAGTTCTCGAAGAAACCGTAAACCTTTAAAGAGATCTAGTGCTCGTCGGAACCGCCGTGCGAGG
atad-3
5,780   5,800   5,820   5,840   5,860   5,880

AAAGCTGTTGAGTCGAAGGAATATGCCATTTTGCTGTTTCCCTCTTGCCGTTGGCCTTGACCTGGTTGTTGTGGACCGCCAGGAGCAGCACCTGCTTGAAAATCATC
TTTCGACAACTCAGCTTCCTTATACGGTAAAACGACAAAGGGAGAACGGCAACCGGAACTGGACCAACAACACCTGGCGGTCCTCGTCGTGGACGAACTTTTAGTAG
atad-3
5,900   5,920   5,940   5,960   5,980

CGGAATCTGCGGAGTAGCGTTTTTTTGAACACCAAATAACCAAGACATctgaaagaatagattgaagcttgggatggtgcaaaaatattctaaggaatattgattaa
GCCTTAGACGCCTCATCGCAAAAAAACTTGTGGTTTATTGGTTCTGTAgactttcttatctaacttcgaaccctaccacgtttttataagattccttataactaatt
atad-3   Patad-3
6,000   6,020   6,040   6,060   6,080

aggaaatgatttatatatgtacttatgccaaagtatgaaacaataatttctttaaaggaatgaaattcggagagactttgtgtaatccttgaagaaaacgtagtatt
tcctttactaaatatatacatgaatacggtttcatactttgttattaaagaaatttccttactttaagcctctctgaaacacattaggaacttcttttgcatcataa
Patad-3
6,100   6,120   6,140   6,160   6,180   6,200
```

ATAD-3 in pCFJ1178 (10862 bp) (from 6207-7062 bp)

aaaacagagttgcaaccttttcagtaaaattagatgatgcaaaacagattttccggctcattttccctgaatttaaaggacctgccgtcaaaaaagacctgccgtcg
ttttgtctcaacgttggaaaagtcattttaatctactacgttttgtctaaaaggccgagtaaaagggacttaaatttcctggacggcagttttttctggacggcagc

Patad-3

6,220 6,240 6,260 6,280 6,300

ttataattcacgaatttctatttttgaaacacattctgcattgaaaaataaactttaaaaaattaaattaagattgttggaaaaaaacagaaatcgctaaggcaga
aatattaagtgcttaaagataaaaactttgtgtaagacgtaactttttatttgaaatttttaatttaattctaacaacctttttttgtctttagcgattccgtct

Patad-3

6,320 6,340 6,360 6,380 6,400 6,420

ttatgctccaatgacattttctgacgcatttctcgatatcgtaaagtgcgccagcgttcaaaggccacatctgaggccccacgaaaaggggagcagaacgaaaaggg
aatacgaggttactgtaaaagactgcgtaaagagctatagcatttcacgcggtcgcaagtttccggtgtagactccggggtgcttttcccctcgtcttgcttttccc

Patad-3

6,440 6,460 6,480 6,500 6,520

gatctgcaaaaaggggatctgcgaaaaggggagatacggaaaggggagatacgaaaaggggagcaacgaaaaggggatctggcactgtgccaaacgctatttttctc
ctagacgtttttcccctagacgcttttcccctctatgcctttcccctctatgcttttcccctcgttgcttttcccctagaccgtgacacggtttgcgataaaaagag

Patad-3

6,540 6,560 6,580 6,600 6,620

gaagaaaacgatacaacgattgtcgcgaatacgcagaatactgatgttccgccgatcacgactgagaaatgatattaatacccaggaaatgcgtcaaggaaatatta
cttcttttgctatgttgctaacagcgcttatgcgtcttatgactacaaggcggctagtgctgactctttactataattatgggtcctttacgcagttcctttataat

Patad-3

6,640 6,660 6,680 6,700 6,720 6,740

aagaattattatgattattacgaagtctttccgtattctaaatgctcacgacaatccaccgaagtcatcaacaacgacaaagccgggaaactcctgtttgaaagccg
ttcttaataatactaataatgcttcagaaaggcataagatttacgagtgctgttaggtggcttcagtagttgttgctgtttcggccctttgaggacaaactttcggc

Patad-3

6,760 6,780 6,800 6,820 6,840

cgccaaaagacacacacgtcacgagtgttacagtactaccacacatgcacatcaaaccccaacacgtcgacaccgcgcaaggaaatcgtcacttcttcgttttactc
gcggttttctgtgtgtgcagtgctcacaatgtcatgatggtgtgtacgtgtagtttggggttgtgcagctgtggcgcgttcctttagcagtgaagaagcaaaatgag

Patad-3

6,860 6,880 6,900 6,920 6,940

ccggaccggcaggtcgcatgttaaatatgagctttctcatttttatcattcaacggatttatcagtaataaattatataattctaccacttttgaacctataccgat
ggcctggccgtccagcgtacaatttatactcgaaagagtaaaaatagtaagttgcctaaatagtcattatttaatatattaagatggtgaaaacttggatatggcta

Patad-3

6,960 6,980 7,000 7,020 7,040 7,060

ATAD-3 in pCFJ1178 (10862 bp) (from 7063-7918 bp)

```
ccgaaacatggtgcatcggctcCCCGGGCCtagttctagacattctctaatgaaaaaatctttcagttgaaattgaaaatgagttaaagttggagtttttattgaaa
ggctttgtaccacgtagccgagGGGCCCGGatcaagatctgtaagagattactttttagaaagtcaactttaacttttactcaatttcaacctcaaaaataacttt
Patad-3        C. briggsae unc-119(+) region
7,080  7,100  7,120  7,140  7,160

acagatttccgtgtgattagtgtttttagcgagtgtgacaggacagcgaaaaaatatagaaacaagggggaactgaaaagcttaggaatgcattgaacatgagaag
tgtctaaaggcacactaatcacaaaaatcgctcacactgtcctgtcgcttttttatatctttgttcccccttgacttttcgaatccttacgtaacttgtactcttc
C. briggsae unc-119(+) region
7,180  7,200  7,220  7,240  7,260

gggaaggggaaggaacaaactagacaggaattattggaatttaatcacatttggagtttttttctattcgacagaataattatccagaacatttttgtattaaata
cccttccccttccttgtttgatctgtccttaataaccttaaattagtgtaaacctcaaaaaaagataagctgtcttattaataggtcttgtaaaaacataatttat
C. briggsae unc-119(+) region
7,280  7,300  7,320  7,340  7,360  7,380

tttatgcatcatatgagtagtcggctttgttgtgcatgacgagtttgttatcgacgaaatagaagctgtcagaacgagtctcgtttggattgttgatcatgtcgtcc
aaatacgtagtatactcatcagccgaaacaacacgtactgctcaaacaatagctgctttatcttcgacagtcttgctcagagcaaacctaacaactagtacagcagg
unc-119 CDS
C. briggsae unc-119(+) region
7,400  7,420  7,440  7,460  7,480

actgaaaaagagattagtcttttgaattgtactttttagaataatgactcactgagttgttgagagagttgagggaactcatagatatgttcacagttgtttcgtga
tgactttttctctaatcagaaaacttaacatgaaaaatcttattactgagtgactcaacaactctctcaactcccttgagtatctatacaagtgtcaacaaagcact
unc-119 CDS
C. briggsae unc-119(+) region
7,500  7,520  7,540  7,560  7,580

attcggaatacagaatccgaattcaaagtcaaaacacttcagaaggcgatctttgaagaagtgacgttcgatcattcggaaatgatggattgggatgtctcctactt
taagccttatgtcttaggcttaagtttcagttttgtgaagtcttccgctagaaacttcttcactgcaagctagtaagcctttactacctaaccctacagaggatgaa
unc-119 CDS
C. briggsae unc-119(+) region
7,600  7,620  7,640  7,660  7,680  7,700

tgaattccacagttgctccgaccgttttgagtttcaaaaagtttggagcaaatctataacgcacgtatcttgccgactcctgtggcgactcatcattctcctgatcg
acttaaggtgtcaacgaggctggcaaaactcaaagtttttcaaacctcgtttagatattgcgtgcatagaacggctgaggacaccgctgagtagtaagaggactagc
unc-119 CDS
C. briggsae unc-119(+) region
7,720  7,740  7,760  7,780  7,800

ttctccggtttggcgatctcgaaaagcacttgctcagtgtccagatcacggatttggaacttggtgaactcgatgttatagatgttcgcagatggggagcataagaa
aagaggccaaaccgctagagcttttcgtgaacgagtcacaggtctagtgcctaaaccttgaaccacttgagctacaatatctacaagcgtctacccctcgtattctt
unc-119 CDS
C. briggsae unc-119(+) region
7,820  7,840  7,860  7,880  7,900
```

ATAD-3 in pCFJ1178 (10862 bp) (from 7919-8774 bp)

tcctaaatttatgttttaaactgaaatccaaagggagcaagataccttgagtgattcccggaagtgctaaaacgtcgttcggagtgatttgagctttcttcgcaagc
aggatttaaatacaaaatttgactttaggtttccctcgttctatggaactcactaagggccttcacgattttgcagcaagcctcactaaactcgaaagaagcgttcg

unc-119 CDS
C. briggsae unc-119(+) region

7,920 7,940 7,960 7,980 8,000 8,020

tccgattccgttgtgattccttgttcggtgcttggtggtggccgtggcatctggaaatatggaaaagttcaacaaaaagaaaagagaaaagaatgaaatcggatatc
aggctaaggcaacactaaggaacaagccacgaaccaccaccggcaccgtagacctttatacctttcaagttgtttttcttttctcttttcttactttagcctatag

unc-119 CDS
C. briggsae unc-119(+) region

8,040 8,060 8,080 8,100 8,120

aagagttagttgagcggtttctctagttttctgagtctcacctgcgacgggaaggtcgccgagccgggtggaatcgatCgttgttgctcggctttcatatcggtttg
ttctcaatcaactcgccaaagagatcaaaagactcagagtggacgctgcccttccagcggctcggcccaccttagctaGcaacaacgagccgaaagtatagccaaac

unc-119 CDS
C. briggsae unc-119(+) region

8,140 8,160 8,180 8,200 8,220

gttggaagcggctgaaaacggaaagaagtggaagaaggaaaagagtgtggtgtgacaggaaaatggtaattagagggtgccaaataaccagctatattttgtttttt
caaccttcgccgacttttgcctttcttcaccttcttccttttctcacaccacactgtccttttaccattaatctcccacggtttattggtcgatataaaacaaaaaa

unc-119 CDS
C. briggsae unc-119(+) region

8,240 8,260 8,280 8,300 8,320 8,340

tttgaaaacatttttaaaaagaaaaatacgataatgatatcagatggatttccggaaaactggtatgaaaaatttcaaccttttttgagtacatgtaatcaaaataca
aaacttttgtaaaaatttttctttttatgctattactatagtctacctaaaggccttttgaccatactttttaaagttggaaaaaactcatgtacattagttttatgt

C. briggsae unc-119(+) region

8,360 8,380 8,400 8,420 8,440

ctttgtaaattatcatttttattgaaactccaccatttttctatttataacgctaataatttgaaaaagaaacctAttgcgaaccgcggggtgaatcccaaaaacga
gaaacatttaatagtaaaaataactttgaggtggtaaaaagataaatattgcgattattaaactttttctttggaTaacgcttggcgccccacttagggtttttgct

C. briggsae unc-119(+) region

8,460 8,480 8,500 8,520 8,540 8,560

atgcgttttggtggagtgattgattcgaatcgaagaagaaaaagaagaagacgtggaatagagagctcactcttaaccgagcagcacacaccgacagaaaaaaaaat
tacgcaaaaccacctcactaactaagcttagcttcttctttttcttcttctgcaccttatctctcgagtgagaattggctcgtcgtgtgtggctgtcttttttttta

C. briggsae unc-119(+) region

8,580 8,600 8,620 8,640 8,660

gaaatgaatgagggtcttcttcttcttcttcttcgaatgattgacagaaatgggaaaaagaggaagattgagaagggaaaaaggaaggagaaaagaagcagaagaag
ctttacttactcccagaagaagaagaagaagaagcttactaactgtctttaccctttttctccttctaactcttcccttttttccttcctcttttcttcgtcttcttc

C. briggsae unc-119(+) region

8,680 8,700 8,720 8,740 8,760

ATAD-3 in pCFJ1178 (10862 bp) (from 8775-9630 bp)

acgtcagagaggagaggaacgagcggaaaagcagcgggcgcaagtcatagaagtagcagagctggggagaagaagacactatccaagaaaggaatgacgagagagta
tgcagtctctcctctccttgctcgccttttcgtcgcccgcgttcagtatcttcatcgtctcgacccctcttcttctgtgataggttctttccttactgctctctcat
C. briggsae unc-119(+) region
8,780 8,800 8,820 8,840 8,860 8,880

tgcaaaggggtatagggtgcagacagaataggaacagaataacagatgatgagccaagaagagttgaaaagggcgatgaatttgtcatgtaacttaatttgggtcaa
acgtttccccatatcccacgtctgtcttatccttgtcttattgtctactactcggttcttctcaacttttcccgctacttaaacagtacattgaattaaacccagtt
C. briggsae unc-119(+) region
8,900 8,920 8,940 8,960 8,980

tttgagcatgatgaattgaaatcatcccttgttgggagttaataaccggtttgttatcagaaaccctgtaatagaagggcgccctaactttgagccaattcatcccg
aaactcgtactacttaactttagtagggaacaaccctcaattattggccaaacaatagtctttgggacattatcttcccgcgggattgaaactcggttaagtagggc
C. briggsae unc-119(+) region
9,000 9,020 9,040 9,060 9,080

gtttctgtcaaatatatcaaaaagtggtcaactgacaaattgtttttgatattataataaacattttatccgttaacaattttcgaatactttttacaaggacttgg
caaagacagtttatatagtttttcaccagttgactgtttaacaaaaactataatattatttgtaaaataggcaattgttaaaagcttatgaaaaatgttcctgaacc
C. briggsae unc-119(+) region
9,100 9,120 9,140 9,160 9,180 9,200

ataaattggctcaaagagcctgctttttttgtacaaacttgaattcaaaaacgcagaactcgtactgttggatgcctgtgtagcggtcctctattgatttttgctatt
tatttaaccgagtttctcggacgaaaaaaacatgtttgaacttaagtttttgcgtcttgagcatgacaacctacggacacatcgccaggagataactaaaaacgataa
left recom 10882
9,220 9,240 9,260 9,280 9,300

ttttgataattttactgtaaattatgagttattccaattaacattcctagctaaatgtaagttagcgaccaatttttttagcaaccccattttatgacttttcagaat
aaaactattaaaatgacatttaatactcaataaggttaattgtaaggatcgatttacattcaatcgctggttaaaaaatcgttggggtaaaatactgaaaagtctta
left recom 10882
9,320 9,340 9,360 9,380 9,400

atcgcctactgggtcaacgacacttgtttggaactaagggtcttcccaattttttttaaatcttcataggcttgacagaatcagggttcttcaagcagctttgagag
tagcggatgacccagttgctgtgaacaaaccttgattcccagaagggttaaaaaaaatttagaagtatccgaactgtcttagtcccaagaagttcgtcgaaactctc
left recom 10882
9,420 9,440 9,460 9,480 9,500 9,520

ggaatcaattgcaacatttgatgttaaatttatattttttatagttctctggtgaaatggggaccaccgcaaagccccttttatgtttttaacttgaataaaatctt
ccttagttaacgttgtaaactacaatttaaatataaaaaatatcaagagaccactttacccctggtggcgtttcggggaaaatacaaaaattgaacttattttagaa
left recom 10882
9,540 9,560 9,580 9,600 9,620

ATAD-3 in pCFJ1178 (10862 bp) (from 9631-10486 bp)

gtattaaattgtgatttatagatctgtatgggtaatttgaaggaaaagaaagggagctcccaaaagtgccttacagatccaggcccaataaatgtaaactattgggc
cataatttaacactaaatatctagacatacccattaaacttccttttctttccctcgagggttttcacggaatgtctaggtccgggttatttacatttgataacccg
left recom 10882
9,640 9,660 9,680 9,700 9,720

agtagtatggcaaaaatcgtgcggaatttgaaaaacctagaattaaaataaatattcaacacaaatgttcgagatattttaagttcggagtgtatatctagaacata
tcatcataccgtttttagcacgccttaaactttttggatcttaattttatttataagttgtgtttacaagctctataaaattcaagcctcacatatagatcttgtat
left recom 10882
9,740 9,760 9,780 9,800 9,820 9,840

ttgtacaagtaggtctacgggatcgtctacgggaaaatttgatttcctcgaagatgtatttaatgaattctctaatgttgcaggtctttgtatttgacagaatgttg
aacatgttcatccagatgccctagcagatgcccttttaaactaaaggagcttctacataaattacttaagagattacaacgtccagaaacataaactgtcttacaac
left recom 10882
9,860 9,880 9,900 9,920 9,940

gtttttgaaacttattcgccataattgcttggctactacatgtctcatgccattgacactatcctgtactcgaaatttgttaagtaactacccaccgtccacttatg
caaaaactttgaataagcggtattaacgaaccgatgatgtacagagtacggtaactgtgataggacatgagctttaaacaattcattgatgggtggcaggtgaatac
left recom 10882
9,960 9,980 10,000 10,020 10,040

acgagagggtcaattaaggtcactccacaaaagtaaatttgaaaacacatgagaaaaagtgatctgaattcccttgaagtggctaaaacaaagcagatgagggatac
tgctctcccagttaattccagtgaggtgttttcatttaaacttttgtgtactctttttcactagacttaagggaacttcaccgattttgtttcgtctactccctatg
left recom 10882
10,060 10,080 10,100 10,120 10,140 10,160

gacgtatgcgactactgagagataaggtcgaaacttgataaggatgctgcttacctgcttagcaagtttcttcgcacaggagaaagtgacaagctaaactatcaaat
ctgcatacgctgatgactctctattccagctttgaactattcctacgacgaatggacgaatcgttcaaagaagcgtgtcctctttcactgttcgatttgatagttta
left recom 10882
10,180 10,200 10,220 10,240 10,260

tcatctaaaacctcaaaaaattattttggatgaaataatgttttattttgagcaacgcataaggcaaagaataaattaaaggcgatccgcttagcggcgagttccgt
agtagattttggagtttttaataaaacctactttattacaaaataaaactcgttgcgtattccgtttcttatttaatttccgctaggcgaatcgccgctcaaggca
left recom 10882
10,280 10,300 10,320 10,340 10,360

cctcgaagaagattcctccatcgatggcgcagtactttggacagattccacgctctcctggggttcctggtggtccgactggtcctggctgtcctgggtttccgtcg
ggagcttcttctaaggaggtagctaccgcgtcatgaaacctgtctaaggtgcgagaggaccccaaggaccaccaggctgaccaggaccgacaggacccaaaggcagc
left recom 10882
10,400 10,420 10,440 10,460 10,480

ATAD-3 in pCFJ1178 (10862 bp) (from 10487-10862 bp)

gctcctggcttgccgtctggtcctggtggtccctttggtccaggttgtcctgaaagatgtggtgattagatatggattgaaaaactatgaaatgattacctggggtt
cgaggaccgaacggcagaccaggaccaccagggaaaccaggtccaacaggactttctacaccactaatctatacctaactttttgatactttactaatggaccccaa

left recom 10882

10,500 10,520 10,540 10,560 10,580

ccgtcgtttcctggttgtccggatggtcctggtggtccgactggtcctggctctccggtggatcctggctctccctttggactggctcgcagacagctggaagtcc
ggcagcaaaggaccaacaggcctaccaggaccaccaggctgaccaggaccgagaggccacctaggaccgagagggaaaacctgaccgagcgtctgtcgaccttcagg

left recom 10882

10,600 10,620 10,640 10,660 10,680 10,700

ctccaactttctatacaaagttgatagcttggcgtaatcatggtcatagctgtttcctgtgtgaaattgttatccgctggtatcagctcactcaaaggcggtaatac
gaggttgaaagatatgtttcaactatcgaaccgcattagtaccagtatcgacaaaggacacactttaacaataggcgaccatagtcgagtgagtttccgccattatg

M13-rev

10,720 10,740 10,760 10,780 10,800

ggttatccacagaatcaggggataacgcaggaaagaacatgtgagcaaaaggcca
ccaataggtgtcttagtcccctattgcgtcctttcttgtacactcgttttccggt

10,810 10,820 10,830 10,840 10,850 10,860

maph-1.1::GFP repair template (10528 bp)

tcgcgcgtttcggtgatgacggtgaaaacctctgacacatgcagctcccggagacggtcacagcttgtctgtaagcggatgccgggagcagacaagcccgtcagggc
agcgcgcaaagccactactgccacttttggagactgtgtacgtcgagggcctctgccagtgtcgaacagacattcgcctacggccctcgtctgttcgggcagtcccg

20 40 60 80 100

gcgtcagcgggtgttggcgggtgtcggggctggcttaactatgcggcatcagagcagattgtactgagagtgcaccatatgcggtgtgaaataccgcacagatgcgt
cgcagtcgcccacaaccgcccacagccccgaccgaattgatacgccgtagtctcgtctaacatgactctcacgtggtatacgccacactttatggcgtgtctacgca

120 140 160 180 200

aaggagaaaataccgcatcaggcgccattcgccattcaggctgcgcaactgttgggaagggcgatcggtgcgggcctcttcgctattacgccagctggcgaaagggg
ttcctcttttatggcgtagtccgcggtaagcggtaagtccgacgcgttgacaacccttcccgctagccacgcccggagaagcgataatgcggtcgaccgctttcccc

220 240 260 280 300 320

gatgtgctgcaaggcgattaagttgggtaacgccagggttttcccagtcacgacgttgtaaaacgacggccagtcgccggcatcccactcattttcgctctcacaca
ctacacgacgttccgctaattcaacccattgcggtcccaaaagggtcagtgctgcaacattttgctgccggtcagcggccgtagggtgagtaaaagcgagagtgtgt

M13-fwd

340 360 380 400 420

ctcacacacacacaaaacgagaccaacacactctagatgcccgctcacaaccacatttgctcccttctcatgattggctaaatggtttttttttggtatttgtttgg
gagtgtgtgtgtgttttgctctggttgtgtgagatctacgggcgagtgttggtgtaaacgagggaagagtactaaccgatttaccaaaaaaaaaccataaacaaacc

LeftHomologyArm maph-1.1

440 460 480 500 520

acactaggcaacaaccaactattcgtctggattctttcattttccctagatgtttctcgtttcgttcccagtaacttttccaaaaagtatcataagtaatatcgtaa
tgtgatccgttgttggttgataagcagacctaagaaagtaaaagggatctacaaagagcaaagcaagggtcattgaaaaggtttttcatagtattcattatagcatt

LeftHomologyArm maph-1.1

540 560 580 600 620 640

aatagtttattgctgtgtttcggactagatttgagacttatttcaactttacaaggaagactgctatcacaaaactatctgaacctttttcattaaatttcatcatt
ttatcaaataacgacacaaagcctgatctaaactctgaataaagttgaaatgttccttctgacgatagtgttttgatagacttggaaaaagtaatttaaagtagtaa

LeftHomologyArm maph-1.1

660 680 700 720 740

ttttaacagaagatacggtacctgctctcgtcacgactgtaaattttcaactttagtgtgcatttttgccagaagccatgaatctttttttactctattttccctata
aaaattgtcttctatgccatggacgagagcagtgctgacatttaaaagttgaaatcacacgtaaaaacggtcttcggtacttagaaaaaatgagataaaagggatat

LeftHomologyArm maph-1.1

760 780 800 820 840

tttttgacgttattttgacatttttgattggttttggcacctttttagtcattaatatggttcttttactgaaaaatgaagtctacagaaaagcgtgaaaaactct
aaaaaactgcaataaaactgtaaaaactaaccaaaaccgtggaaaaatcagtaattataccaagaaaatgactttttacttcagatgtcttttcgcactttttgaga

LeftHomologyArm maph-1.1

860 880 900 920 940 960

maph-1.1::GFP repair template (10528 bp) (from 964-1819 bp)

gaaattagggtttttgtagtcattttgacataaaattcgccctaatttaatccttcagcgttgtttttcattttatacgtatacgcgtttaaaactcttgaaaagaa
ctttaatcccaaaaacatcagtaaaactgtattttaagcgggattaaattaggaagtcgcaacaaaaagtaaaatatgcatatgcgcaaattttgagaacttttctt
LeftHomologyArm maph-1.1
980 1,000 1,020 1,040 1,060

catttttccagATGAGTAAAGGAGAAGAATTGTTCACTGGAGTTGTCCCAATCCTCGTCGAGCTCGACGGAGACGTCAACGGACACAAGTTCTCCGTCTCCGGAGA
gtaaaaaaggtcTACTCATTTCCTCTTCTTAACAAGTGACCTCAACAGGGTTAGGAGCAGCTCGAGCTGCCTCTGCAGTTGCCTGTGTTCAAGAGGCAGAGGCCTCT
GFP
1,080 1,100 1,120 1,140 1,160

GGGAGAGGGAGACGCCACCTACGGAAAGCTCACCCTCAAGTTCATCTGCACCACCGGAAAGCTCCCAGTCCCATGGCCAACCCTCGTCACCACCTTCTGCTACGGAG
CCCTCTCCCTCTGCGGTGGATGCCTTTCGAGTGGGAGTTCAAGTAGACGTGGTGGCCTTTCGAGGGTCAGGGTACCGGTTGGGAGCAGTGGTGGAAGACGATGCCTC
GFP
1,180 1,200 1,220 1,240 1,260 1,280

TCCAATGCTTCTCCCGTTACCCAGACCACATGAAGCGTCACGACTTCTTCAAGTCCGCCATGCCAGAGGGATACGTCCAAGAGCGTACCATCTTCTTCAAGgtaagt
AGGTTACGAAGAGGGCAATGGGTCTGGTGTACTTCGCAGTGCTGAAGAAGTTCAGGCGGTACGGTCTCCCTATGCAGGTTCTCGCATGGTAGAAGAAGTTCcattca
GFP
1,300 1,320 1,340 1,360 1,380

ttaaacatatatatactaactactgattatttaaattttcagGACGACGGAAACTACAAGACCCGTGCCGAGGTCAAGTTCGAGGGAGACACCCTCGTCAACCGTAT
aatttgtatatatatgattgatgactaataaatttaaaagtcCTGCTGCCTTTGATGTTCTGGGCACGGCTCCAGTTCAAGCTCCCTCTGTGGGAGCAGTTGGCATA
GFP
1,400 1,420 1,440 1,460 1,480

CGAGCTCAAGgtaagtttaaacagttcggtactaactaaccatacatatttaaattttcagGGAATCGACTTCAAGGAGGACGGAAACATCCTCGGACACAAGCTCG
GCTCGAGTTCcattcaaatttgtcaagccatgattgattggtatgtataaatttaaaagtcCCTTAGCTGAAGTTCCTCCTGCCTTTGTAGGAGCCTGTGTTCGAGC
GFP GFP
1,500 1,520 1,540 1,560 1,580 1,600

AGTACAACTACAACTCCCACAACGTCTACATCATGGCCGACAAGCAAAAGAACGGAATCAAGGTCAACTTCAAGgtaagtttaaacatgattttactaactaactaa
TCATGTTGATGTTGAGGGTGTTGCAGATGTAGTACCGGCTGTTCGTTTTCTTGCCTTAGTTCCAGTTGAAGTTCcattcaaatttgtactaaaatgattgattgatt
GFP
1,620 1,640 1,660 1,680 1,700

tctgatttaaattttcagATCCGTCACAACATCGAGGACGGATCCGTCCAACTCGCCGACCACTACCAACAAAACACCCCAATCGGAGACGGACCAGTCCTCCTCCC
agactaaatttaaaagtcTAGGCAGTGTTGTAGCTCCTGCCTAGGCAGGTTGAGCGGCTGGTGATGGTTGTTTTGTGGGGTTAGCCTCTGCCTGGTCAGGAGGAGGG
GFP
1,720 1,740 1,760 1,780 1,800

maph-1.1::GFP repair template (10528 bp) (from 1820-2675 bp)

AGACAACCACTACCTCTCCACCCAATCCGCCCTCTCCAAGGACCCAAACGAGAAGCGTGACCACATGGTCCTCCTCGAGTTCGTCACCGCCGCCGGAATCACCCACG
TCTGTTGGTGATGGAGAGGTGGGTTAGGCGGGAGAGGTTCCTGGGTTTGCTCTTCGCACTGGTGTACCAGGAGGAGCTCAAGCAGTGGCGGCGGCCTTAGTGGGTGC

GFP

1,820 1,840 1,860 1,880 1,900 1,920

GAATGGACGAGCTCTACAAGGAGAATCTGTACTTTCAATCCGGAAAGgtaagtttaaaATAACTTCGTATAGCATACATTATACGAAGTTATtttcagGGAGAGCAA
CTTACCTGCTCGAGATGTTCCTCTTAGACATGAAAGTTAGGCCTTTCcattcaaatttTATTGAAGCATATCGTATGTAATATGCTTCAATAaaagtcCCTCTCGTT

GFP TEV site loxP

1,940 1,960 1,980 2,000 2,020

AAGCTTATCTCCGAGGAGGACCTCTAAGGATGATCGACGCCaACGTCGTTGAATTTTCAAATTTTAAATACTGAATATTTGTTTTTTTTCCTATTATTTATTTATTC
TTCGAATAGAGGCTCCTCCTGGAGATTCCTACTAGCTGCGGtTGCAGCAACTTAAAAGTTTAAAATTTATGACTTATAAACAAAAAAAAGGATAATAAATAAATAAG

myc let-858 Terminator

2,040 2,060 2,080 2,100 2,120 2,140

TCTTTGTGTTTTTTTTCTTGCTTTCTAAAAAATTAATTCAATCCAAATCTAAacatttttttttctctttccgtctcccaattcgtattccgctcctctcatctgaa
AGAAACACAAAAAAAAGAACGAAAGATTTTTTAATTAAGTTAGGTTTAGATTtgtaaaaaaaaagagaaaggcagagggttaagcataaggcgaggagagtagactt

let-858 Terminator

2,160 2,180 2,200 2,220 2,240

cacaatgtgcaagtttatttatcttctcgctttcatttcattaggacgtggggggaattggtggaagggggaaacacacaaaaggatgatggaaatgaaataaggac
gtgttacacgttcaaataaatagaagagcgaaagtaaagtaatcctgcaccccccttaaccaccttccccctttgtgtgttttcctactacctttactttattcctg

let-858 Terminator

2,260 2,280 2,300 2,320 2,340

acacaatatgcaacaacattcaattcagaaatatggaggaaggtttaaaagaaaacataaaaatatatagaggaggaaggaaactagctgccctcgattgtctatct
tgtgttatacgttgttgtaagttaagtctttatacctccttccaaattttcttttgtatttttatatatctcctccttcctttgatcgacgggagctaacagataga

let-858 Terminator Psqt1

2,360 2,380 2,400 2,420 2,440 2,460

tccattctcaccaagtttcaacaactttattcagtcattcttctctggttcatttcccaaccaatcttcccgaataaagcccatctgtcctttcggccacattcctc
aggtaagagtggttcaaagttgttgaaataagtcagtaagaagagaccaagtaaagggttggttagaagggcttatttcgggtagacaggaaagccggtgtaaggag

Psqt1

2,480 2,500 2,520 2,540 2,560

acctacatccgtactccttatctcccgttcttatccagtcttttatcgtctctgcgtctcgcacactgtctcgtcggtaatgtaaaaaaaaaacaaaaaaaacttga
tggatgtaggcatgaggaatagagggcaagaataggtcagaaaatagcagagacgcagagcgtgtgacagagcagccattacattttttttttgttttttttgaact

Psqt1

2,580 2,600 2,620 2,640 2,660

maph-1.1::GFP repair template (10528 bp) (from 2676-3531 bp)

```
aaacttgcggaaagatttcctaataaaggagacgaatccggtaggaaatcgacaagtctctgcttctcgaagactaggtgagttacatttctttggccgaattatt
tttgaacgcctttctaaaggattattttcctctgcttaggccatcctttagctgttcagagacgaagagcttctgatccactcaatgtaaagaaaccggcttaataa
                                              Psqt1
   2,680          2,700          2,720          2,740          2,760          2,780

ttgaaatcctaaatttgataatttcagagATGTCTGTAAAACTTGCGTGTTATGTGACGGCTTCGGTCACTGTCGCCACTCTTATGGTATGCTTCATGACCATGTCA
aactttaggatttaaactattaaagtctcTACAGACATTTTGAACGCACAATACACTGCCGAAGCCAGTGACAGCGGTGAGAATACCATACGAAGTACTGGTACAGT
         Psqt1                                SQT-1
             2,800          2,820          2,840          2,860          2,880

ACCATCTACTCGGAGGTTGATGGATTCAGAGAGAAGCTTGATACCGAGATGAATGTGTTCAGAgtgagttaaattatatttgaattttaaataatttttaatttc
TGGTAGATGAGCCTCCAACTACCTAAGTCTCTCTTCGAACTATGGCTCTACTTACACAAGTCTcactcaatttaatataaacttaaaatttattaaaaattaaaag
                    SQT-1
       2,900          2,920          2,940          2,960          2,980

tagCAATCTACCAATGGATTGTGGAAGGACATAGTTGTCATCGGAAGATCTAGCAAGCGTGTCCGTTGTCAATATGAAGAGACCAACGCTACCCCAACTCCACATGC
atcGTTAGATGGTTACCTAACACCTTCCTGTATCAACAGTAGCCTTCTAGATCGTTCGCACAGGCAACAGTTATACTTCTCTGGTTGCGATGGGGTTGAGGTGTACG
                                         SQT-1
3,000          3,020          3,040          3,060          3,080          3,100

TGATGGATCCCCATCTGCTCCACCAGGTCAACCACCAGCAGTTCCACCAGTCTTCAACCAGCCAAAGACTCCAAATGGAGCCAATGGAAATGGACCAACCTGCAACT
ACTACCTAGGGGTAGACGAGGTGGTCCAGTTGGTGGTCGTCAAGGTGGTCAGAAGTTGGTCGGTTTCTGAGGTTTACCTCGGTTACCTTTACCTGGTTGGACGTTGA
                                              SQT-1
           3,120          3,140          3,160          3,180          3,200

GCAATGCTGATAACAAGTGCCCAGCTGGACCATCCGGACCAAAGGGAGTTCCAGGAGTTCCAGGACTCGACGGAGTTCCAGGACTTGACGGTGTTCCAGGAGTTGGA
CGTTACGACTATTGTTCACGGGTCGACCTGGTAGGCCTGGTTTCCCTCAAGGTCCTCAAGGTCCTGAGCTGCCTCAAGGTCCTGAACTGCCACAAGGTCCTCAACCT
                                              SQT-1
      3,220          3,240          3,260          3,280          3,300

GCTGATGATATCGCTCCACAACGCGAGTCTGTCGGATGCTTCACTTGCCCACAAGGACCAGTTGGACCACCAGGAGCTCTTGGAAGACCAGGACCACGTGGACTTCC
CGACTACTATAGCGAGGTGTTGCGCTCAGACAGCCTACGAAGTGAACGGGTGTTCCTGGTCAACCTGGTGGTCCTCGAGAACCTTCTGGTCCTGGTGCACCTGAAGG
                                              SQT-1
3,320          3,340          3,360          3,380          3,400          3,420

AGGACCAAGAGGACAAAATGGAAACCCAGGAAGAGATGGACAACCAGGACATCCAGGAGAGCAAGGATCATCCGGTCAAATCGGAAAGATCGGAGAGCCAGGACCAC
TCCTGGTTCTCCTGTTTTACCTTTGGGTCCTTCTCTACCTGTTGGTCCTGTAGGTCCTCTCGTTCCTAGTAGGCCAGTTTAGCCTTTCTAGCCTCTCGGTCCTGGTG
                                              SQT-1
          3,440          3,460          3,480          3,500          3,520
```

maph-1.1::GFP repair template (10528 bp) (from 3532-4387 bp)

```
CAGGAGAGAAGGGACGCGACGCCGAGCATCCAATCGGAAGACCAGGACCAAAGGGACCAAGAGGAGATCAAGGACCAACAGGACCAGCTGGACAGAACGGTCTTCAC
GTCCTCTCTTCCCTGCGCTGCGGCTCGTAGGTTAGCCTTCTGGTCCTGGTTTCCCTGGTTCTCCTCTAGTTCCTGGTTGTCCTGGTCGACCTGTCTTGCCAGAAGTG
SQT-1
3,540 3,560 3,580 3,600 3,620

GGACCACCAGGAGAGCCAGGAACCGTTGGACCAGAAGGACCATCTGGAAAGCAAGGACGTCAAGGACCAGACGGAACCCAGGGAGAGACTGGACCAGACGGAAGACC
CCTGGTGGTCCTCTCGGTCCTTGGCAACCTGGTCTTCCTGGTAGACCTTTCGTTCCTGCAGTTCCTGGTCTGCCTTGGGTCCCTCTCTGACCTGGTCTGCCTTCTGG
SQT-1
3,640 3,660 3,680 3,700 3,720 3,740

AGGAAAGGATGCCGAGTACTGCCAGTGCCCAGACAAGTCTCCACCATCAGAGGCTGTCAACGCCAACCGTGGATACAGAAATATCTAAattgttggtgttttctaat
TCCTTTCCTACGGCTCATGACGGTCACGGGTCTGTTCAGAGGTGGTAGTCTCCGACAGTTGCGGTTGGCACCTATGTCTTTATAGATTtaacaaccacaaaagatta
SQT-1 sqt-1 3'UTR
3,760 3,780 3,800 3,820 3,840

aaaatatttgagattcagtgagactttttttcttggcggcacaataaagtttcctctttttgttgggtcactcaacgaacacaatacaaatatttgagcgctcgcatt
ttttataaactctaagtcactctgaaaaaagaaccgccgtgttatttcaaaggagaaaaacaacccagtgagttgcttgtgttatgtttataaactcgcgagcgtaa
sqt-1 3'UTR tbb-2 3'UTR
3,860 3,880 3,900 3,920 3,940

tttctcaacctttcgggtttaaagcacttcaaaatggaattgaagagagattgtgaaagtacatcagacagaatcacattttgatagcattcacttcactcagatgc
aaagagttggaaagcccaaatttcgtgaagttttaccttaacttctctctaacactttcatgtagtctgtcttagtgtaaaactatcgtaagtgaagtgagtctacg
tbb-2 3'UTR
3,960 3,980 4,000 4,020 4,040 4,060

aaacttttcagtgactataaaaagtcataaaataataacattaaaaaagcaaataaattagcgagagaatttttttgacaaaaagaaagaagagtgatagagaagaag
tttgaaaagtcactgatatttttcagtattttattattgtaattttttcgtttatttaatcgctctcttaaaaaaactgtttttctttcttctcactatctcttcttc
tbb-2 3'UTR
4,080 4,100 4,120 4,140 4,160

ggaatgcttgaaaggattttgcatttatcttaATCACCGTCTTCGAGAAGACGAACCATGGCGCCAGTTTCAGAATCCAAGTTTCTAATATAGTTCATCACAATGTT
ccttacgaactttcctaaaacgtaaatagaatTAGTGGCAGAAGCTCTTCTGCTTGGTACCGCGGTCAAAGTCTTAGGTTCAAAGATTATATCAAGTAGTGTTACAA
tbb-2 3'UTR Cre
4,180 4,200 4,220 4,240 4,260 4,280

GACGTTGGTCCAACCTCCGGCTTGCATAATCTCTGGGATGGAAACTCCAGCGCGAGCCATGTCACGGGCAGCTCCGACACGGGCGGAATGTCCAGACCAAGCCAAGT
CTGCAACCAGGTTGGAGGCCGAACGTATTAGAGACCCTACCTTTGAGGTCGCGCTCGGTACAGTGCCCGTCGAGGCTGTGCCCGCCTTACAGGTCTGGTTCGGTTCA
Cre
4,300 4,320 4,340 4,360 4,380
```

maph-1.1::GFP repair template (10528 bp) (from 4388-5243 bp)

AACGTTGTCCGGAATCATCCTTGGCTCCGTAGATGAGACGGTGGGTAGCTTCGAAGATTCctgaaaatttattttcagcttattcttagctaatatgttgtttaaac
TTGCAACAGGCCTTAGTAGGAACCGAGGCATCTACTCTGCCACCCATCGAAGCTTCTAAGgacttttaaataaaagtcgaataagaatcgattatacaacaaatttg
Cre
4,400 4,420 4,440 4,460 4,480

ttacCTTCAAGAGCTCGAGTCGAGAGTTGTGATGTTGCACTCGGGGCAGCAACTCCGTTCTTGCGAACACGACAGAACAGGTAGTTGTTTGGATCATCGGCGACTCC
aatgGAAGTTCTCGAGCTCAGCTCTCAACACTACAACGTGAGCCCCGTCGTTGAGGCAAGAACGCTTGTGCTGTCTTGTCCATCAACAAACCTAGTAGCCGCTGAGG
Cre
4,500 4,520 4,540 4,560 4,580 4,600

GGAAACCGAAATCCAGCGCTCAACGAGCTTGGTAACTCCAAGGGAGAGCGCTTTTTCGACTCCGGCAGTCGAGACCAGCGTTTTTGTACGTCCGATGTGGATGAGCA
CCTTTGGCTTTAGGTCGCGAGTTGCTCGAACCATTGAGGTTCCCTCTCGCGAAAAAGCTGAGGCCGTCAGCTCTGGTCGCAAAAACATGCAGGCTACACCTACTCGT
Cre
4,620 4,640 4,660 4,680 4,700

TTCTTCCTCCGTCTGTGCGACTAATATCCTTGACGCGAATGCGGGCAATTTCGGCGATACGGAGAAGTGTGTTGTAAGCAATTCCGAGGAAGGCGAGGTTTCGGATG
AAGAAGGAGGCAGACACGCTGATTATAGGAACTGCGCTTACGCCCGTTAAAGCCGCTATGCCTCTTCACACAACATTCGTTAAGGCTCCTTCCGCTCCAAAGCCTAC
Cre
4,720 4,740 4,760 4,780 4,800

TCCTGGCAACGATCGGAATTTTCCATAAGAGAACGGACCTGATCGAAGTCGGTTctgaaaatttcgtcaagtcttaggattgaatacattaatatttaaacttacCT
AGGACCGTTGCTAGCCTTAAAAGGTATTCTCTTGCCTGGACTAGCTTCAGCCAAgacttttaaagcagttcagaatcctaacttatgtaattataaatttgaatgGA
Cre
4,820 4,840 4,860 4,880 4,900 4,920

TTCGAAGGCGAGGGCTTGCTTGGCTCTCTCTCCAGCATCAACATTTTCCTTGCGGATACGTCTCATTACGAGAGATACGGCATTAGAATCTGATGGGCGTGGAAGTC
AAGCTTCCGCTCCCGAACGAACCGAGAGAGAGGTCGTAGTTGTAAAAGGAACGCCTATGCAGAGTAATGCTCTCTATGCCGTAATCTTAGACTACCCGCACCTTCAG
Cre
4,940 4,960 4,980 5,000 5,020

CAGATCGGCGATGGAGCATATTGAGCTGTCCGAGATGTTGTTGGATCGTCTTCACCGCCAATCCACGAGCTTGGAGGTAGAGCAAGTAGTCACGAACGTCTTCTGGC
GTCTAGCCGCTACCTCGTATAACTCGACAGGCTCTACAACAACCTAGCAGAAGTGGCGGTTAGGTGCTCGAACCTCCATCTCGTTCATCAGTGCTTGCAGAAGACCG
Cre
5,040 5,060 5,080 5,100 5,120

TCGGCTGGGAACCACTTGCGGTTGTTAAGCTTGCACCAGGCAGCCCAAGAACGGCACACTGAGAGAAGCATCTTCCAGGTGTGCTCGGAGAAGGCTTGTCTGTCACG
AGCCGACCCTTGGTGAACGCCAACAATTCGAACGTGGTCCGTCGGGTTCTTGCCGTGTGACTCTCTTCGTAGAAGGTCCACACGAGCCTCTTCCGAACAGACAGTGC
Cre
5,140 5,160 5,180 5,200 5,220 5,240

maph-1.1::GFP repair template (10528 bp) (from 5244-6099 bp)

```
GAACATATCCATGAGGTTCTTGCGAACTTCATCACTTGTCGCGTCAACTGGAAGTGCTGGAAGATTTTGATGAACAGTGAGAAGATTTGACATattttcgaagtttt
CTTGTATAGGTACTCCAAGAACGCTTGAAGTAGTGAACAGCGCAGTTGACCTTCACGACCTTCTAAAACTACTTGTCACTCTTCTAAACTGTAtaaaagcttcaaaa
Cre
    5,260     5,280     5,300     5,320     5,340

ttagatgcactagaacaaagcgtgttggcttcctctgagcccgctttccttatatacccgcattctgcagccttacagaatgttctagaaggtcctagatgcattcg
aatctacgtgatcttgtttcgcacaaccgaaggagactcgggcgaaaggaatatatgggcgtaagacgtcggaatgtcttacaagatcttccaggatctacgtaagc
hsp16-41 promoter
  5,360     5,380     5,400     5,420     5,440

tttgaaaatactcccggtgggtgcaaagagacgcagacggaaaatgtatctgggtctctttattgtgtacactacttttccatgtaccgaatgtgagtcgccctcct
aaacttttatgagggccacccacgtttctctgcgtctgccttttacatagacccagagaaataacacatgtgatgaaaaggtacatggcttacactcagcgggagga
hsp16-41 promoter
5,460     5,480     5,500     5,520     5,540     5,560

tttgcaacaagcagctcgaatgttctagaaaaaggtggaaaatagtataaataccgttgaaaataaataccgaacaacatttgctctaattgtgaaattagaaatct
aaacgttgttcgtcgagcttacaagatctttttccaccttttatcatatttatggcaacttttatttatggcttgttgtaaacgagattaacactttaatctttaga
hsp16-41 promoter
    5,580     5,600     5,620     5,640     5,660

tcaaactataatcatgtcactttaccactatttccgtccagctcaacgttccgtttttggtgatcATTTTTGCTTTCGTCGTAAATCTACACACGCGTCTCTTCCGT
agtttgatattagtacagtgaaatggtgataaaggcaggtcgagttgcaaggcaaaaaccactagTAAAAACGAAAGCAGCATTTAGATGTGTGCGCAGAGAAGGCA
hsp16-41 promoter                                     Prps-0
  5,680     5,700     5,720     5,740     5,760

GCGAGAGTCCAAGCCAGCAGCCAAATTCGTTGACTGAGTATTCAACGTTTATACGTTGTCGGCAACGAGAAATAGGAAAATGCATCGGGAAATGTTCTTTTTTCGAT
CGCTCTCAGGTTCGGTCGTCGGTTTAAGCAACTGACTCATAAGTTGCAAATATGCAACAGCCGTTGCTCTTTATCCTTTTACGTAGCCCTTTACAAGAAAAAAGCTA
Prps-0
5,780     5,800     5,820     5,840     5,860     5,880

TTTTTCCAAGGTTTTGACAAATTTTACCACGAATTTTGCTATGTTTTCAATTAAAAAATATGTTATTCAACTGTTTCTATGAGGAAAATAAGGCTTTGCATGTAATT
AAAAAGGTTCCAAAACTGTTTAAAATGGTGCTTAAAACGATACAAAAGTTAATTTTTTATACAATAAGTTGACAAAGATACTCCTTTTATTCCGAAACGTACATTAA
Prps-0
    5,900     5,920     5,940     5,960     5,980

TTCTTATTCAGCATAATTTTTAATTAATTTGAATTTTCTGTCCTAACGTTTATTTTGTTTTCTTGGTTATGACTGATCTGAAATTAATTTTTGAATTTTAAGGTAAT
AAGAATAAGTCGTATTAAAAATTAATTAAACTTAAAAGACAGGATTGCAAATAAAACAAAAGAACCAATACTGACTAGACTTTAATTAAAAACTTAAAATTCCATTA
Prps-0
  6,000     6,020     6,040     6,060     6,080
```

maph-1.1::GFP repair template (10528 bp) (from 6100-6955 bp)

ATGTCAGGCGGTGCCGCAAGTTTGTACAAAAAAGCAGGCTCCATGAAAAAGCCTGAACTCACCGCGACGTCTGTCGAGAAGTTTCTGATCGAAAAGTTCGACAGCGT
TACAGTCCGCCACGGCGTTCAAACATGTTTTTTCGTCCGAGGTACTTTTTCGGACTTGAGTGGCGCTGCAGACAGCTCTTCAAAGACTAGCTTTTCAAGCTGTCGCA

hygR

6,100 6,120 6,140 6,160 6,180 6,200

CTCCGACCTGATGCAGCTCTCGGAGGGCGAAGAATCTCGTGCTTTCAGCTTCGATGTAGGAGGGCGTGGATATGTCCTGCGGGTAAATAGCTGCGCCGATGGTTTCT
GAGGCTGGACTACGTCGAGAGCCTCCCGCTTCTTAGAGCACGAAAGTCGAAGCTACATCCTCCCGCACCTATACAGGACGCCCATTTATCGACGCGGCTACCAAAGA

hygR

6,220 6,240 6,260 6,280 6,300

ACAAAGATCGTTATGTTTATCGGCACTTTGCATCGGCCGCGCTCCCGATTCCGGAAGTGCTTGACATTGGGGAATTCAGCGAGAGCCTGACCTATTGCATCTCCCGC
TGTTTCTAGCAATACAAATAGCCGTGAAACGTAGCCGGCGCGAGGGCTAAGGCCTTCACGAACTGTAACCCCTTAAGTCGCTCTCGGACTGGATAACGTAGAGGGCG

hygR

6,320 6,340 6,360 6,380 6,400 6,420

CGTGCACAGGGTGTCACGTTGCAAGACCTGCCTGAAACCGAACTGCCCGCTGTTCTGCAGCCGGTCGCGGAGGCCATGGATGCGATCGCTGCGGCCGATCTTAGCCA
GCACGTGTCCCACAGTGCAACGTTCTGGACGGACTTTGGCTTGACGGGCGACAAGACGTCGGCCAGCGCCTCCGGTACCTACGCTAGCGACGCCGGCTAGAATCGGT

hygR

6,440 6,460 6,480 6,500 6,520

GACGAGCGGGTTCGGCCCATTCGGACCGCAAGGAATCGGTCAATACACTACATGGCGTGATTTCATATGCGCGATTGCTGATCCCCATGTGTATCACTGGCAAACTG
CTGCTCGCCCAAGCCGGGTAAGCCTGGCGTTCCTTAGCCAGTTATGTGATGTACCGCACTAAAGTATACGCGCTAACGACTAGGGGTACACATAGTGACCGTTTGAC

hygR

6,540 6,560 6,580 6,600 6,620

TGATGGACGACACCGTCAGTGCGTCCGTCGCGCAGGCTCTCGATGAGCTGATGCTTTGGGCCGAGGACTGCCCCGAAGTCCGGCACCTCGTGCACGCGGATTTCGGC
ACTACCTGCTGTGGCAGTCACGCAGGCAGCGCGTCCGAGAGCTACTCGACTACGAAACCCGGCTCCTGACGGGGCTTCAGGCCGTGGAGCACGTGCGCCTAAAGCCG

hygR

6,640 6,660 6,680 6,700 6,720 6,740

TCCAACAATGTCCTGACGGACAATGGCCGCATAACAGCGGTCATTGACTGGAGCGAGGCGATGTTCGGGGATTCCCAATACGAGGTCGCCAACATCTTCTTCTGGAG
AGGTTGTTACAGGACTGCCTGTTACCGGCGTATTGTCGCCAGTAACTGACCTCGCTCCGCTACAAGCCCCTAAGGGTTATGCTCCAGCGGTTGTAGAAGAAGACCTC

hygR

6,760 6,780 6,800 6,820 6,840

GCCGTGGTTGGCTTGTATGGAGCAGCAGACGCGCTACTTCGAGCGGAGGCATCCGGAGCTTGCAGGATCGCCGCGGCTCCGGGCGTATATGCTCCGCATTGGTCTTG
CGGCACCAACCGAACATACCTCGTCGTCTGCGCGATGAAGCTCGCCTCCGTAGGCCTCGAACGTCCTAGCGGCGCCGAGGCCCGCATATACGAGGCGTAACCAGAAC

hygR

6,860 6,880 6,900 6,920 6,940

maph-1.1::GFP repair template (10528 bp) (from 6956-7811 bp)

ACCAACTCTATCAGAGCTTGGTTGACGGCAATTTCGATGATGCAGCTTGGGCGCAGGGTCGATGCGACGCAATCGTCCGATCCGGAGCCGGGACTGTCGGGCGTACA
TGGTTGAGATAGTCTCGAACCAACTGCCGTTAAAGCTACTACGTCGAACCCGCGTCCCAGCTACGCTGCGTTAGCAGGCTAGGCCTCGGCCCTGACAGCCCGCATGT

hygR

6,960 6,980 7,000 7,020 7,040 7,060

CAAATCGCCCGCAGAAGCGCGGCCGTCTGGACCGATGGCTGTGTAGAAGTACTCGCCGATAGTGGAAACCGACGCCCCAGCACTCGTCCGAGGGCAAAGGAATAGAC
GTTTAGCGGGCGTCTTCGCGCCGGCAGACCTGGCTACCGACACATCTTCATGAGCGGCTATCACCTTTGGCTGCGGGGTCGTGAGCAGGCTCCCGTTTCCTTATCTG

hygR

7,080 7,100 7,120 7,140 7,160

CCAGCTTTCTTGTACAAAGTGGGTCCAATTACTCTTCAACATCCCTACATGCTCTTTCTCCCTGTGCTCCCACCCCCTATTTTTGTTATTATCAAAAAACTTCTCTT
GGTCGAAAGAACATGTTTCACCCAGGTTAATGAGAAGTTGTAGGGATGTACGAGAAAGAGGGACACGAGGGTGGGGGATAAAAACAATAATAGTTTTTTGAAGAGAA

unc-54 3'UTR

7,180 7,200 7,220 7,240 7,260

AATTTCTTTGTTTTTTAGCTTCTTTTAAGTCACCTCTAACAATGAAATTGTGTAGATTCAAAAATAGAATTAATTCGTAATAAAAAGTCGAAAAAAATTGTGCTCCC
TTAAAGAAACAAAAAATCGAAGAAAATTCAGTGGAGATTGTTACTTTAACACATCTAAGTTTTTATCTTAATTAAGCATTATTTTTCAGCTTTTTTTAACACGAGGG

unc-54 3'UTR

7,280 7,300 7,320 7,340 7,360 7,380

TCCCCCCATTAATAATAATTCTATCCCAAAATCTACACAATGTTCTGTGTACACTTCTTATGTTTTTTACTTCTGATAAATTTTTTTGAAACATCATAGAAAAAACC
AGGGGGGTAATTATTATTAAGATAGGGTTTTAGATGTGTTACAAGACACATGTGAAGAATACAAAAAATGAAGACTATTTAAAAAAACTTTGTAGTATCTTTTTTGG

unc-54 3'UTR

7,400 7,420 7,440 7,460 7,480

GCACACAAAATACCTTATCATATGTTACGTTTCAGTTTATGACCGCAATTTTTAATAACTTCGTATAGCATACATTATACGAAGTTATtttcagGGAGCCGGATCTG
CGTGTGTTTTATGGAATAGTATACAATGCAAAGTCAAATACTGGCGTTAAAAATTATTGAAGCATATCGTATGTAATATGCTTCAATAaaagtcCCTCGGCCTAGAC

unc-54 3'UTR loxP

7,500 7,520 7,540 7,560 7,580

ATTATAAAGACGATGACGATAAGCGTGACTACAAGGACGACGACGACAAGCGTGATTACAAGGATGACGATGACAAGAGAATGCCGGAGGAATATATCATGTCGTCA
TAATATTTCTGCTACTGCTATTCGCACTGATGTTCCTGCTGCTGCTGTTCGCACTAATGTTCCTACTGCTACTGTTCTCTTACGGCCTCCTTATATAGTACAGCAGT

Flag tags RightHomolArm maph-1.1

7,600 7,620 7,640 7,660 7,680 7,700

AAGGCcTGtATaTAtGTCcTcGGCGGTGCGGCCAACACGGCCGCCCTGTTTGATTTCGACGGCGTCTACATTCTCGATGGGGGATTCGCCGAAAAGAACCCGGGATT
TTCCGgACaTAtATaCAGgAgCCGCCACGCCGGTTGTGCCGGCGGGACAAACTAAAGCTGCCGCAGATGTAAGAGCTACCCCCTAAGCGGCTTTTCTTGGGCCCTAA

RightHomolArm maph-1.1

7,720 7,740 7,760 7,780 7,800

maph-1.1::GFP repair template (10528 bp) (from 7812-8774 bp)

```
TGTGGCTCACGTCAGGGACGTCTCTGCCGTTCTGCTCGCCGCTCCGACGCTGGGAAATCTTGGAACCACATCTGCTCTGTTGGAACAGgtttgtagcacctttagtg
ACACCGAGTGCAGTCCCTGCAGAGACGGCAAGACGAGCGGCGAGGCTGCGACCCTTTAGAACCTTGGTGTAGACGAGACAACCTTGTCcaaacatcgtggaaatcac
RightHomolArm maph-1.1
7,820 7,840 7,860 7,880 7,900

caatttttgataatattttttcagGGAAAACCGTTGCCGGTGTTCACCAACACGAAGCCGTTCAAGACTGCAAAGCCAGGAAGCAGTGGAGAGATTGCCAAAGCA
gttaaaaactattataaaaaagtcCCTTTTGGCAACGGCCACAAGTGGTTGTGCTTCGGCAAGTTCTGACGTTTCGGTCCTTCGTCACCTCTCTAACGGTTTCGT
RightHomolArm maph-1.1
7,920 7,940 7,960 7,980 8,000 8,020

ATTCAAGAAGCCAACTCGAAAATCCTCTCCGTGGCTCCACCACTGTTCAATCCAAAATACCCGGCAAACATCATATACCAGTCAGCTGCAAAAGGAGTTCTCTCACT
TAAGTTCTTCGGTTGAGCTTTTAGGAGAGGCACCGAGGTGGTGACAAGTTAGGTTTTATGGGCCGTTTGTAGTATATGGTCAGTCGACGTTTTCCTCAAGAGAGTGA
RightHomolArm maph-1.1
8,040 8,060 8,080 8,100 8,120

GTATATTCTGGCTGGTGACGTCAAGGATGCCGAAGTGATCACAAAGGCGTTGGCTGGAGGAAACGAGGCTGAAGTGGAGAAGGCGGCCGCTGAGCATGGAACCATCG
CATATAAGACCGACCACTGCAGTTCCTACGGCTTCACTAGTGTTTCCGCAACCGACCTCCTTTGCTCCGACTTCACCTCTTCCGCCGGCGACTCGTACCTTGGTAGC
8,140 8,160 8,180 8,200 8,220

GCGTCCTGCTCTGGAGACCTGCGATGACTGATCAATCGGTGGTCAGAGTGCTCATTTCGataacatggtcatagctgtttcctgtgtgaaattgttatccgctcaca
CGCAGGACGAGACCTCTGGACGCTACTGACTAGTTAGCCACCAGTCTCACGAGTAAAGCtattgtaccagtatcgacaaaggacacactttaacaataggcgagtgt
M13-rev
8,240 8,260 8,280 8,300 8,320 8,340

attccacacaacatacgagccggaagcataaagtgtaaagcctggggtgcctaatgagtgagctaactcacattaattgcgttgcgctcactgcccgctttccagtc
taaggtgtgttgtatgctcggccttcgtatttcacatttcggaccccacggattactcactcgattgagtgtaattaacgcaacgcgagtgacgggcgaaaggtcag
8,360 8,380 8,400 8,420 8,440

gggaaacctgtcgtgccagctgcattaatgaatcggccaacgcgcggggagaggcggtttgcgtattgggcgctcttccgcttcctcgctcactgactcgctgcgct
ccctttggacagcacggtcgacgtaattacttagccggttgcgcgcccctctccgccaaacgcataacccgcgagaaggcgaaggagcgagtgactgagcgacgcga
8,460 8,480 8,500 8,520 8,540 8,560

cggtcgttcggctgcggcgagcggtatcagctcactcaaaggcggtaatacggttatccacagaatcaggggataacgcaggaaagaacatgtgagcaaaaggccag
gccagcaagccgacgccgctcgccatagtcgagtgagtttccgccattatgccaataggtgtcttagtcccctattgcgtcctttcttgtacactcgttttccggtc
8,580 8,600 8,620 8,640 8,660

caaaaggccaggaaccgtaaaaaggccgcgttgctggcgtttttccataggctccgcccccctgacgagcatcacaaaaatcgacgctcaagtcagaggtggcgaaa
gttttccggtccttggcatttttccggcgcaacgaccgcaaaaaggtatccgaggcggggggactgctcgtagtgtttttagctgcgagttcagtctccaccgcttt
ColE1 origin
8,680 8,700 8,720 8,740 8,760
```

maph-1.1::GFP repair template (10528 bp) (from 8775-9630 bp)

cccgacaggactataaagataccaggcgtttccccctggaagctccctcgtgcgctctcctgttccgaccctgccgcttaccggatacctgtccgcctttctccctt
gggctgtcctgatatttctatggtccgcaaagggggaccttcgagggagcacgcgagaggacaaggctgggacggcgaatggcctatggacaggcggaaagagggaa
ColE1 origin
8,780 8,800 8,820 8,840 8,860 8,880

cgggaagcgtggcgctttctcatagctcacgctgtaggtatctcagttcggtgtaggtcgttcgctccaagctgggctgtgtgcacgaaccccccgttcagcccgac
gcccttcgcaccgcgaaagagtatcgagtgcgacatccatagagtcaagccacatccagcaagcgaggttcgacccgacacacgtgcttggggggcaagtcgggctg
ColE1 origin
8,900 8,920 8,940 8,960 8,980

cgctgcgccttatccggtaactatcgtcttgagtccaacccggtaagacacgacttatcgccactggcagcagccactggtaacaggattagcagagcgaggtatgt
gcgacgcggaataggccattgatagcagaactcaggttgggccattctgtgctgaatagcggtgaccgtcgtcggtgaccattgtcctaatcgtctcgctccataca
ColE1 origin
9,000 9,020 9,040 9,060 9,080

aggcggtgctacagagttcttgaagtggtggcctaactacggctacactagaagaacagtatttggtatctgcgctctgctgaagccagttaccttcggaaaaagag
tccgccacgatgtctcaagaacttcaccaccggattgatgccgatgtgatcttcttgtcataaaccatagacgcgagacgacttcggtcaatggaagcctttttctc
ColE1 origin
9,100 9,120 9,140 9,160 9,180 9,200

ttggtagctcttgatccggcaaacaaaccaccgctggtagcggtggtttttttgtttgcaagcagcagattacgcgcagaaaaaaaggatctcaagaagatcctttg
aaccatcgagaactaggccgtttgtttggtggcgaccatcgccaccaaaaaaacaaacgttcgtcgtctaatgcgcgtctttttttcctagagttcttctaggaaac
ColE1 origin
9,220 9,240 9,260 9,280 9,300

atcttttctacggggtctgacgctcagtggaacgaaaactcacgttaagggattttggtcatgagattatcaaaaaggatcttcacctagatccttttaaattaaaa
tagaaaagatgccccagactgcgagtcaccttgcttttgagtgcaattccctaaaaccagtactctaatagtttttcctagaagtggatctaggaaaatttaatttt
ColE1 origin
9,320 9,340 9,360 9,380 9,400

atgaagttttaaatcaatctaaagtatatatgagtaaacttggtctgacagttaccaatgcttaatcagtgaggcacctatctcagcgatctgtctatttcgttcat
tacttcaaaatttagttagatttcatatatactcatttgaaccagactgtcaatggttacgaattagtcactccgtggatagagtcgctagacagataaagcaagta
AmpR
9,420 9,440 9,460 9,480 9,500 9,520

ccatagttgcctgactccccgtcgtgtagataactacgatacgggagggcttaccatctggccccagtgctgcaatgataccgcgagacccacgctcaccggctcca
ggtatcaacggactgaggggcagcacatctattgatgctatgccctcccgaatggtagaccggggtcacgacgttactatggcgctctgggtgcgagtggccgaggt
AmpR
9,540 9,560 9,580 9,600 9,620

maph-1.1::GFP repair template (10528 bp) (from 9631-10528 bp)

gatttatcagcaataaaccagccagccggaagggccgagcgcagaagtggtcctgcaactttatccgcctccatccagtctattaattgttgccgggaagctagagt
ctaaatagtcgttatttggtcggtcggccttcccggctcgcgtcttcaccaggacgttgaaataggcggaggtaggtcagataattaacaacggcccttcgatctca
AmpR
9,640 9,660 9,680 9,700 9,720

aagtagttcgccagttaatagtttgcgcaacgttgttgccattgctacaggcatcgtggtgtcacgctcgtcgtttggtatggcttcattcagctccggttcccaac
ttcatcaagcggtcaattatcaaacgcgttgcaacaacggtaacgatgtccgtagcaccacagtgcgagcagcaaaccataccgaagtaagtcgaggccaagggttg
AmpR
9,740 9,760 9,780 9,800 9,820 9,840

gatcaaggcgagttacatgatcccccatgttgtgcaaaaaagcggttagctccttcggtcctccgatcgttgtcagaagtaagttggccgcagtgttatcactcatg
ctagttccgctcaatgtactagggggtacaacacgttttttcgccaatcgaggaagccaggaggctagcaacagtcttcattcaaccggcgtcacaatagtgagtac
AmpR
9,860 9,880 9,900 9,920 9,940

gttatggcagcactgcataattctcttactgtcatgccatccgtaagatgcttttctgtgactggtgagtactcaaccaagtcattctgagaatagtgtatgcggcg
caataccgtcgtgacgtattaagagaatgacagtacggtaggcattctacgaaaagacactgaccactcatgagttggttcagtaagactcttatcacatacgccgc
AmpR
9,960 9,980 10,000 10,020 10,040

accgagttgctcttgcccggcgtcaatacgggataataccgcgccacatagcagaactttaaaagtgctcatcattggaaaacgttcttcggggcgaaaactctcaa
tggctcaacgagaacgggccgcagttatgccctattatggcgcggtgtatcgtcttgaaattttcacgagtagtaaccttttgcaagaagccccgcttttgagagtt
AmpR
10,060 10,080 10,100 10,120 10,140 10,160

ggatcttaccgctgttgagatccagttcgatgtaacccactcgtgcacccaactgatcttcagcatcttttactttcaccagcgtttctgggtgagcaaaaacagga
cctagaatggcgacaactctaggtcaagctacattgggtgagcacgtgggttgactagaagtcgtagaaaatgaaagtggtcgcaaagacccactcgtttttgtcct
10,180 10,200 10,220 10,240 10,260

aggcaaaatgccgcaaaaaagggaataagggcgacacggaaatgttgaatactcatactcttcctttttcaatattattgaagcatttatcagggttattgtctcat
tccgttttacggcgttttttcccttattcccgctgtgcctttacaacttatgagtatgagaaggaaaaagttataataacttcgtaaatagtcccaataacagagta
10,280 10,300 10,320 10,340 10,360

gagcggatacatatttgaatgtatttagaaaaataaacaaataggggttccgcgcacatttccccgaaaagtgccacctgacgtctaagaaaccattattatcatga
ctcgcctatgtataaacttacataaatctttttatttgtttatccccaaggcgcgtgtaaaggggcttttcacggtggactgcagattctttggtaataatagtact
10,400 10,420 10,440 10,460 10,480

cattaacctataaaaataggcgtatcacgaggccctttcgtc
gtaattggatatttttatccgcatagtgctccgggaaagcag
10,490 10,500 10,510 10,520

Pbeta actin eGFP::MAPH-1.1 (9407 bp)

gagcacaaacgttaattaattccccagcaggcaggtaattaacagtgtgactccctttttgctgcgagtggggctgatacagagagatgtggcactatggagcccac
ctcgtgtttgcaattaattaaggggtcgtccgtccattaattgtcacactgagggaaaaacgacgctcaccccgactatgtctctctacaccgtgatacctcgggtg

20 40 60 80 100

ggggtcctggcactgggtgcccacggaggtccccatgtgctgcagtgtcaccgcctccgaggtgacagtattgtccctgcggtgtccctgcagctcagctctgtcca
ccccaggaccgtgacccacgggtgcctccaggggtacacgacgtcacagtggcggaggctccactgtcataacagggacgccacagggacgtcgagtcgagacaggt
Beta-actin promoter

120 140 160 180 200

cagggccacctccagtttggaggggacacaatgcagccccgatgcaacccatcctcgcagcatcccagggacaaagaccccactgcaagaccgcacacagggctggg
gtcccggtggaggtcaaacctcccctgtgttacgtcggggctacgttgggtaggagcgtcgtagggtccctgtttctggggtgacgttctggcgtgtgtcccgaccc
Beta-actin promoter

220 240 260 280 300 320

tcccgctcccctaatatctacagtgcttttgcatggccccttaatcaatgcagttaatcagcatgcgctcatgcaccgctctggagctgcaaagcccctcgcagcgc
agggcgaggggattatagatgtcacgaaaacgtaccggggaattagttacgtcaattagtcgtacgcgagtacgtggcgagacctcgacgtttcggggagcgtcgcg
Beta-actin promoter

340 360 380 400 420

tgctcaccaacaccgcgcaccgccccggcccagcctgcagcacgcgctgcaaacaggaaagaaacaaaatattgcccaaatgtaggcaaaggcattcggctgccttg
acgagtggttgtggcgcgtggcggggccgggtcggacgtcgtgcgcgacgtttgtcctttctttgttttataacgggtttacatccgtttccgtaagccgacggaac
Beta-actin promoter

440 460 480 500 520

acctccgccgggccgggccctgcctgactcagctccttactcagcgctcgcttcctccctccggctgccaccgccgcagcgcacaccctgacaaagagtggccctta
tggaggcggcccggcccgggacggactgagtcgaggaatgagtcgcgagcgaaggagggaggccgacggtggcggcgtcgcgtgtgggactgtttctcaccgggaat
Beta-actin promoter

540 560 580 600 620 640

acgggctctgaggtgcacccagcagtgcactcagcagtccaagggccggcctggaggtttgcaccgctacgtgctgacattagcattgaacttggccctgggtagtg
tgcccgagactccacgtgggtcgtcacgtgagtcgtcaggttcccggccggacctccaaacgtggcgatgcacgactgtaatcgtaacttgaaccgggacccatcac
Beta-actin promoter

660 680 700 720 740

ctgcaggccgggcggggtgggtgtagagagtgcagcgcgcgttgcacccggtgccccttcccctcccttgcatcccagcaggctgcaccccagcaccaggcccgtgc
gacgtccggcccgccccacccacatctctcacgtcgcgcgcaacgtgggccacggggaaggggagggaacgtagggtcgtccgacgtggggtcgtggtccgggcacg
Beta-actin promoter

760 780 800 820 840

Pbeta actin eGFP::MAPH-1.1 (9407 bp) (from 857-1712 bp)

atgcatgctcctggtgttattgcagcctggtgcatgcatgcgtcttagtggtgcagcgctgtgcatgcatcctccttggtgtgtagcagcttagtgcatgcataccc
tacgtacgaggaccacaataacgtcggaccacgtacgtacgcagaatcaccacgtcgcgacacgtacgtaggaggaaccacacatcgtcgaatcacgtacgtatggg
Beta-actin promoter
860 880 900 920 940 960

ctcggtgttattgctgctctgtgcaggcacgctcattgtatcacttcatcccagtgcatgcactcacactggagcgattgctgctcggtgcacgcacactcattgta
gagccacaataacgacgagacacgtccgtgcgagtaacatagtgaagtagggtcacgtacgtgagtgtgacctcgctaacgacgagccacgtgcgtgtgagtaacat
Beta-actin promoter
980 1,000 1,020 1,040 1,060

tcacgtcagctcagtggctgcacgcacaccggtgttattgctgctcggtgcgtgcatgcacatcagtgtcgctgcagctcagtgcatgcacgctcattgcccatcgc
agtgcagtcgagtcaccgacgtgcgtgtggccacaataacgacgagccacgcacgtacgtgtagtcacagcgacgtcgagtcacgtacgtgcgagtaacgggtagcg
Beta-actin promoter
1,080 1,100 1,120 1,140 1,160

tatccctgcctctcctgctggcgctccccgggaggtgacttcaaggggaccgcaggaccacctcgggggtgggggggagggctgcacacgcggaccccgctccccctc
atagggacggagaggacgaccgcgaggggccctccactgaagttcccctggcgtcctggtggagcccccaccccccctcccgacgtgtgcgcctggggcgagggggag
Beta-actin promoter
1,180 1,200 1,220 1,240 1,260 1,280

cccaacaaagcactgtggaatcaaaaagggggggaggggggatggaggggcgcgtcacacccccgccccacaccctcacctcgaggtgagccccacgttctgcttcac
gggttgtttcgtgacaccttagtttttccccccctcccccctacctccccgcgcagtgtgggggcggggtgtgggagtggagctccactcggggtgcaagacgaagtg
Beta-actin promoter
1,300 1,320 1,340 1,360 1,380

tctccccatctcccccccctccccacccccaattttgtatttatttattttttaattattttgtgcagcgatgggggcggggggggggggggcgcgcgccaggcggg
agaggggtagaggggggggaggggtgggggttaaaacataaataaataaaaaattaataaaacacgtcgctacccccgccccccccccccccgcgcgcggtccgccc
Beta-actin promoter
1,400 1,420 1,440 1,460 1,480

gcggggcggggcgaggggcggggcggggcgaggcggagaggtgcggcggcagccaatcagagcggcgcgctccgaaagtttccttttatggcgaggcggcggcggcg
cgccccgccccgctccccgccccgccccgctccgcctctccacgccgccgtcggttagtctcgccgcgcgaggctttcaaaggaaaataccgctccgccgccgccgc
Beta-actin promoter
1,500 1,520 1,540 1,560 1,580 1,600

gcggccctataaaaagcgaagcgcgcggcgggcgggagtcgctgcgttgccttcgccccgtgccccgctccgcgccgcctcgcgccgcccgccccggctctgactga
cgccgggatatttttcgcttcgcgcgccgcccgccctcagcgacgcaacggaagcggggcacggggcgaggcgcggcggagcgcggcgggcggggccgagactgact
Beta-actin promoter
1,620 1,640 1,660 1,680 1,700

Pbeta actin eGFP::MAPH-1.1 (9407 bp) (from 1713-2568 bp)

ccgcgttactcccacaggtgagcgggcgggacggcccttctcctccgggctgtaattagcgcttggtttaatgacggctcgtttcttttctgtggctgcgtgaaagc
ggcgcaatgagggtgtccactcgcccgccctgccgggaagaggaggcccgacattaatcgcgaaccaaattactgccgagcaaagaaaagacaccgacgcactttcg
Beta-actin promoter
1,720 1,740 1,760 1,780 1,800

cttaaagggctccgggagggccctttgtgcgggggggagcggctcgggggggtgcgtgcgtgtgtgtgtgcgtggggagcgccgcgtgcggcccgcgctgcccggcgg
gaatttcccgaggccctcccgggaaacacgcccccccctcgccgagcccccccacgcacgcacacacacacgcacccctcgcggcgcacgccgggcgcgacgggccgcc
Beta-actin promoter
1,820 1,840 1,860 1,880 1,900 1,920

ctgtgagcgctgcgggcgcggcgcggggctttgtgcgctccgcgtgtgcgcgaggggagcgcggccgggggcggtgccccgcggtgcgggggggctgcgaggggaac
gacactcgcgacgcccgcgccgcgccccgaaacacgcgaggcgcacacgcgctcccctcgcgccggcccccgccacggggcgccacgcccccccgacgctccccttg
Beta-actin promoter
1,940 1,960 1,980 2,000 2,020

aaaggctgcgtgcggggtgtgtgcgtgggggggtgagcagggggtgtgggcgcggcggtcgggctgtaacccccccctgcacccccctccccgagttgctgagcacg
tttccgacgcacgccccacacacgcacccccccactcgtcccccacacccgcgccgccagcccgacattgggggggacgtggggggaggggctcaacgactcgtgc
Beta-actin promoter
2,040 2,060 2,080 2,100 2,120 2,140

gcccggcttcgggtgcggggctccgtgcggggcgtggcgcggggctcgccgtgccgggcggggggtggcggcaggtgggggtgccgggcggggcggggccgcctcgg
cgggccgaagcccacgccccgaggcacgccccgcaccgcgccccgagcggcacggcccgcccccccaccgccgtccacccccacggcccgccccgccccggcggagcc
Beta-actin promoter
2,160 2,180 2,200 2,220 2,240

gccggggagggctcgggggaggggcgcggcggcccccggagcgccggcggctgtcgaggcgcggcgagccgcagccattgccttttatggtaatcgtgcgagagggcg
cggcccctcccgagccccctccccgcgccgccggggcctcgcggccgccgacagctccgcgccgctcggcgtcggtaacggaaaataccattagcacgctctcccgc
Beta-actin promoter
2,260 2,280 2,300 2,320 2,340

cagggacttcctttgtcccaaatctggcggagccgaaatctgggaggcgccgccgcacccccctctagcgggcgcgggcgaagcggtgcggcgccggcaggaaggaaa
gtccctgaaggaaacagggtttagaccgcctcggctttagaccctccgcggcggcgtggggggagatcgcccgcgcccgcttcgccacgccgcggccgtccttccttt
Beta-actin promoter
2,360 2,380 2,400 2,420 2,440 2,460

tgggcggggagggccttcgtgcgtcgccgcgccgccgtccccttctccatctccagcctcggggctgccgcagggggacggctgccttcgggggggacggggcaggg
acccgcccctcccggaagcacgcagcggcgcggcggcaggggaagaggtagaggtcggagccccgacggcgtccccctgccgacggaagccccccctgccccgtccc
Beta-actin promoter
2,480 2,500 2,520 2,540 2,560

Pbeta actin eGFP::MAPH-1.1 (9407 bp) (from 2569-3424 bp)

cggggttcggcttctggcgtgtgaccggcggggtttatatcttcccttctctgttcctccgcagcccccaagctagcgctaagcttATGGTGAGCAAGGGCGAGGAG
gccccaagccgaagaccgcacactggccgccccaaatatagaagggaagagacaaggaggcgtcgggggttcgatcgcgattcgaaTACCACTCGTTCCCGCTCCTC

Beta-actin promoter | eGFP

2,580 2,600 2,620 2,640 2,660

CTGTTCACCGGGGTGGTGCCCATCCTGGTCGAGCTGGACGGCGACGTAAACGGCCACAAGTTCAGCGTGTCCGGCGAGGGCGAGGGCGATGCCACCTACGGCAAGCT
GACAAGTGGCCCCACCACGGGTAGGACCAGCTCGACCTGCCGCTGCATTTGCCGGTGTTCAAGTCGCACAGGCCGCTCCCGCTCCCGCTACGGTGGATGCCGTTCGA

eGFP

2,680 2,700 2,720 2,740 2,760 2,780

GACCCTGAAGTTCATCTGCACCACCGGCAAGCTGCCCGTGCCCTGGCCCACCCTCGTGACCACCCTGACCTACGGCGTGCAGTGCTTCAGCCGCTACCCCGACCACA
CTGGGACTTCAAGTAGACGTGGTGGCCGTTCGACGGGCACGGGACCGGGTGGGAGCACTGGTGGGACTGGATGCCGCACGTCACGAAGTCGGCGATGGGGCTGGTGT

eGFP

2,800 2,820 2,840 2,860 2,880

TGAAGCAGCACGACTTCTTCAAGTCCGCCATGCCCGAAGGCTACGTCCAGGAGCGCACCATCTTCTTCAAGGACGACGGCAACTACAAGACCCGCGCCGAGGTGAAG
ACTTCGTCGTGCTGAAGAAGTTCAGGCGGTACGGGCTTCCGATGCAGGTCCTCGCGTGGTAGAAGAAGTTCCTGCTGCCGTTGATGTTCTGGGCGCGGCTCCACTTC

eGFP

2,900 2,920 2,940 2,960 2,980

TTCGAGGGCGACACCCTGGTGAACCGCATCGAGCTGAAGGGCATCGACTTCAAGGAGGACGGCAACATCCTGGGGCACAAGCTGGAGTACAACTACAACAGCCACAA
AAGCTCCCGCTGTGGGACCACTTGGCGTAGCTCGACTTCCCGTAGCTGAAGTTCCTCCTGCCGTTGTAGGACCCCGTGTTCGACCTCATGTTGATGTTGTCGGTGTT

eGFP

3,000 3,020 3,040 3,060 3,080 3,100

CGTCTATATCATGGCCGACAAGCAGAAGAACGGCATCAAGGTGAACTTCAAGATCCGCCACAACATCGAGGACGGCAGCGTGCAGCTCGCCGACCACTACCAGCAGA
GCAGATATAGTACCGGCTGTTCGTCTTCTTGCCGTAGTTCCACTTGAAGTTCTAGGCGGTGTTGTAGCTCCTGCCGTCGCACGTCGAGCGGCTGGTGATGGTCGTCT

eGFP

3,120 3,140 3,160 3,180 3,200

ACACCCCCATCGGCGACGGCCCCGTGCTGCTGCCCGACAACCACTACCTGAGCACCCAGTCCAAGCTGAGCAAAGACCCCAACGAGAAGCGCGATCACATGGTCCTG
TGTGGGGGTAGCCGCTGCCGGGGCACGACGACGGGCTGTTGGTGATGGACTCGTGGGTCAGGTTCGACTCGTTTCTGGGGTTGCTCTTCGCGCTAGTGTACCAGGAC

eGFP

3,220 3,240 3,260 3,280 3,300

CTGGAGTTCGTGACCGCCGCCGGGATCACTCTCGGCATGGACGAGCTGTACAAGggcgcgccaATGCCGGAGGAATATATCATGTCGTCAAAGGCGTGCATCTACGT
GACCTCAAGCACTGGCGGCGGCCCTAGTGAGAGCCGTACCTGCTCGACATGTTCccgcgcggtTACGGCCTCCTTATATAGTACAGCAGTTTCCGCACGTAGATGCA

eGFP | MAPH-1.1

3,320 3,340 3,360 3,380 3,400 3,420

Pbeta actin eGFP::MAPH-1.1 (9407 bp) (from 3425-4280 bp)

CTTGGGCGGTGCGGCCAACACGGCCGCCCTGTTTGATTTCGACGGCGTCTACATTCTCGATGGGGGATTCGCCGAAAAGAACCCGGGATTTGTGGCTCACGTCAGGG
GAACCCGCCACGCCGGTTGTGCCGGCGGGACAAACTAAAGCTGCCGCAGATGTAAGAGCTACCCCCTAAGCGGCTTTTCTTGGGCCCTAAACACCGAGTGCAGTCCC
MAPH-1.1
3,440 3,460 3,480 3,500 3,520

ACGTCTCTGCCGTTCTGCTCGCCGCTCCGACGCTGGGAAATCTTGGAACCACATCTGCTCTGTTGGAACAGGGAAAACCGTTGCCGGTGTTCACCAACACGAAGCCG
TGCAGAGACGGCAAGACGAGCGGCGAGGCTGCGACCCTTTAGAACCTTGGTGTAGACGAGACAACCTTGTCCCTTTTGGCAACGGCCACAAGTGGTTGTGCTTCGGC
MAPH-1.1
3,540 3,560 3,580 3,600 3,620

TTCAAGACTGCAAAGCCAGGAAGCAGTGGAGAGATTGCCAAAGCAATTCAAGAAGCCAACTCGAAAATCCTCTCCGTGGCTCCACCACTGTTCAATCCAAAATACCC
AAGTTCTGACGTTTCGGTCCTTCGTCACCTCTCTAACGGTTTCGTTAAGTTCTTCGGTTGAGCTTTTAGGAGAGGCACCGAGGTGGTGACAAGTTAGGTTTTATGGG
MAPH-1.1
3,640 3,660 3,680 3,700 3,720 3,740

GGCAAACATCATATACCAGTCAGCTGCAAAAGGAGTTCTCTCACTGTATATTCTGGCTGGTGACGTCAAGGATGCCGAAGTGATCACAAAGGCGTTGGCTGGAGGAA
CCGTTTGTAGTATATGGTCAGTCGACGTTTTCCTCAAGAGAGTGACATATAAGACCGACCACTGCAGTTCCTACGGCTTCACTAGTGTTTCCGCAACCGACCTCCTT
MAPH-1.1
3,760 3,780 3,800 3,820 3,840

ACGAGGCTGAAGTGGAGAAGGCGGCCGCTGAGCATGGAACCATCGGCGTCCTGCTCTGGAGACCTGCGATGACTGATCAATCGGTGGTCAGAGTGCTCATTTCGGGA
TGCTCCGACTTCACCTCTTCCGCCGGCGACTCGTACCTTGGTAGCCGCAGGACGAGACCTCTGGACGCTACTGACTAGTTAGCCACCAGTCTCACGAGTAAAGCCCT
MAPH-1.1
3,860 3,880 3,900 3,920 3,940

ACTAGTTCACTTTCGAGGATCCAGCAATCGCTTGACAAGGCAGCGAAATCACTTCCATTCCTCAACGTGCCTACCGTGAAGTCGAAGGATGCGCTGTCGGATATTCC
TGATCAAGTGAAAGCTCCTAGGTCGTTAGCGAACTGTTCCGTCGCTTTAGTGAAGGTAAGGAGTTGCACGGATGGCACTTCAGCTTCCTACGCGACAGCCTATAAGG
MAPH-1.1
3,960 3,980 4,000 4,020 4,040 4,060

TGCTCCTGCTGTGCCACGACCTGTTGCCGGAAAACCATCGGCGAGACCTGCAACCACAACAGGCACCGCAACTCGTCCAACTCGTCCAGCAGTCCCTGCAGCCAGTG
ACGAGGACGACACGGTGCTGGACAACGGCCTTTTGGTAGCCGCTCTGGACGTTGGTGTTGTCCGTGGCGTTGAGCAGGTTGAGCAGGTCGTCAGGGACGTCGGTCAC
MAPH-1.1
4,080 4,100 4,120 4,140 4,160

CTCCCAGAGCTTTGACTTCTCGTGCTCCAGCAGGCCCATCTCGTCCGACGACTACACGAAATGCAGCTCCTGCTCCCAGAACAGCTGTCCCATCGCGAGCAACTGTA
GAGGGTCTCGAAACTGAAGAGCACGAGGTCGTCCGGGTAGAGCAGGCTGCTGATGTGCTTTACGTCGAGGACGAGGGTCTTGTCGACAGGGTAGCGCTCGTTGACAT
MAPH-1.1
4,180 4,200 4,220 4,240 4,260 4,280

Pbeta actin eGFP::MAPH-1.1 (9407 bp) (from 4281-5136 bp)

CTCACAAAGACTGCTCCAGCATCGAAGGCTCCAACGAGAGCTCCAGTCCCAGCGAGATCTGCACCAGCGCCACCACGAGGAGCCCCTGCGAAGCCGGCCGCGAACAC
GAGTGTTTCTGACGAGGTCGTAGCTTCCGAGGTTGCTCTCGAGGTCAGGGTCGCTCTAGACGTGGTCGCGGTGGTGCTCCTCGGGGACGCTTCGGCCGGCGCTTGTG
MAPH-1.1
4,300 4,320 4,340 4,360 4,380

AGCCAAGGCCGAACCAATAGCTCAGAAGAAGACCGTTGGAAAAGTTCAAGGAACTGCTCCATCAAAACCTGCTCCGGCGGCTCCAGCTTCGGCAGCCACGTCTCCAG
TCGGTTCCGGCTTGGTTATCGAGTCTTCTTCTGGCAACCTTTTCAAGTTCCTTGACGAGGTAGTTTTGGACGAGGCCGCCGAGGTCGAAGCCGTCGGTGCAGAGGTC
MAPH-1.1
4,400 4,420 4,440 4,460 4,480

CTCCAGCTCCAGAAGCTCCTCGAAGGGATCCGAACAACGTCACCATCGTTCTCGACGATTCGCTCTCTCCAGAAGATTTCAACCAAGGGTCGGCTCCGATGGATATT
GAGGTCGAGGTCTTCGAGGAGCTTCCCTAGGCTTGTTGCAGTGGTAGCAAGAGCTGCTAAGCGAGAGAGGTCTTCTAAAGTTGGTTCCCAGCCGAGGCTACCTATAA
MAPH-1.1
4,500 4,520 4,540 4,560 4,580 4,600

GTCGTGATTCCACCAACACCGGAGCCACCACGTCATGAGGTGGCTCAGGCCACGCATCCAGAGGAGTCGATAATTGACGCGGAGGACTTGGCAAAGGCTCCACTGGA
CAGCACTAAGGTGGTTGTGGCCTCGGTGGTGCAGTACTCCACCGAGTCCGGTGCGTAGGTCTCCTCAGCTATTAACTGCGCCTCCTGAACCGTTTCCGAGGTGACCT
MAPH-1.1
4,620 4,640 4,660 4,680 4,700

AGTCGATGATGTCGCAAATCTGGCTGACGTCGAAGACGAAATTCCGCCACCAGTTGACGCGTTCAAGAAGCCAGAGCCACATCCAGAGCCGAATGTTTCTGGCGGTT
TCAGCTACTACAGCGTTTAGACCGACTGCAGCTTCTGCTTTAAGGCGGTGGTCAACTGCGCAAGTTCTTCGGTCTCGGTGTAGGTCTCGGCTTACAAAGACCGCCAA
MAPH-1.1
4,720 4,740 4,760 4,780 4,800

CCGAGGAAGACAAGATACCAGAGCCAGTCGATGCTTTCAAGAAGCCAGATCCAGTTGAGCTGGACGATTTCGATCCACTAAAGCCATCGCATCCCGAGCCATCTGCG
GGCTCCTTCTGTTCTATGGTCTCGGTCAGCTACGAAAGTTCTTCGGTCTAGGTCAACTCGACCTGCTAAAGCTAGGTGATTTCGGTAGCGTAGGGCTCGGTAGACGC
MAPH-1.1
4,820 4,840 4,860 4,880 4,900 4,920

CCAGTTGTGCCAAGTGACCATATCATCATCGCTACACCTGATCCAGAGCTCCCCGACATTGTTGCTGCTGTTCCATTCGTTTCTCCATCACCAAAAGGTCCAAATGA
GGTCAACACGGTTCACTGGTATAGTAGTAGCGATGTGGACTAGGTCTCGAGGGGCTGTAACAACGACGACAAGGTAAGCAAAGAGGTAGTGGTTTTCCAGGTTTACT
MAPH-1.1
4,940 4,960 4,980 5,000 5,020

TGGACTTGTGAAGTTGGACGATGAGCTGGAGAAGATTGCTCCAGGATTTGAGGAACCACTGATCCCACAAGCTCCACGTGACGATGGCACTCTTGCCGAATGCAGTG
ACCTGAACACTTCAACCTGCTACTCGACCTCTTCTAACGAGGTCCTAAACTCCTTGGTGACTAGGGTGTTCGAGGTGCACTGCTACCGTGAGAACGGCTTACGTCAC
MAPH-1.1
5,040 5,060 5,080 5,100 5,120

Pbeta actin eGFP::MAPH-1.1 (9407 bp) (from 5137-5992 bp)

AAGAAGTGTCCAAATTGGTTGAGATCAGCATGGATACTGATAACAGCGCTGAAGTTGCAGCGGATCTTGCCAAGGCCGTTGGAGAAGTCACTCAGTTGTCGGCAGAT
TTCTTCACAGGTTTAACCAACTCTAGTCGTACCTATGACTATTGTCGCGACTTCAACGTCGCCTAGAACGGTTCCGGCAACCTCTTCAGTGAGTCAACAGCCGTCTA
MAPH-1.1
5,140 5,160 5,180 5,200 5,220 5,240

CTTCAGAATCTGGGTCTCGACGAGAAGACGGATGAGTATGTGCGAAAGCTGAGCAATCAGATGATTGAAGACGCCACACTGCCATTCACCAGTGCACTTGCCAGCAG
GAAGTCTTAGACCCAGAGCTGCTCTTCTGCCTACTCATACACGCTTTCGACTCGTTAGTCTACTAACTTCTGCGGTGTGACGGTAAGTGGTCACGTGAACGGTCGTC
MAPH-1.1
5,260 5,280 5,300 5,320 5,340

TATTGTCACTTCGAACGGATCGGAAACAAACGGCCACGGGGAACAAGCACATGCAGCACAAAATGGAGGAATCGATCATCAGAAGGAGATTCCGAAGCATGATTTAA
ATAACAGTGAAGCTTGCCTAGCCTTTGTTTGCCGGTGCCCCTTGTTCGTGTACGTCGTGTTTTACCTCCTTAGCTAGTAGTCTTCCTCTAAGGCTTCGTACTAAATT
MAPH-1.1
5,360 5,380 5,400 5,420 5,440

TGCAATCGCGCTCCAGTGTGATTGAGAATGGAGCTGCTGTGCAGTACGAGAAGACTGATCCAGCATTGGATGATGTGCTCAATGCATGTGCTCAGGAGAGTGAAAAG
ACGTTAGCGCGAGGTCACACTAACTCTTACCTCGACGACACGTCATGCTCTTCTGACTAGGTCGTAACCTACTACACGAGTTACGTACACGAGTCCTCTCACTTTTC
MAPH-1.1
5,460 5,480 5,500 5,520 5,540 5,560

ATCGATGCTTCCCATCCAGATAATCTTCACATGCCAGCTGCTCCAGGATCCGCCGCCCCAGCGAAGCCAGTCAAGTTCGCGCGCCCATATTATTTCGACGTGGTGAC
TAGCTACGAAGGGTAGGTCTATTAGAAGTGTACGGTCGACGAGGTCCTAGGCGGCGGGGTCGCTTCGGTCAGTTCAAGCGCGCGGGTATAATAAAGCTGCACCACTG
MAPH-1.1
5,580 5,600 5,620 5,640 5,660

AGTGCCAAGGAACGAGAAATTGGAGACCTCTGTTGCAGCAGACGGCCTTCAAGAGTTCATCTCAAAAGTTCGCTCCAGAAATGTGATCCTCGCGTCGAAGGATATCT
TCACGGTTCCTTGCTCTTTAACCTCTGGAGACAACGTCGTCTGCCGGAAGTTCTCAAGTAGAGTTTTCAAGCGAGGTCTTTACACTAGGAGCGCAGCTTCCTATAGA
MAPH-1.1
5,680 5,700 5,720 5,740 5,760

CCGGAGAGCAGCTGCAGGCGATTCTTTGCGGAAAGCAAACATGGTGTGATAGTGCTCACCCATGCAACGTGATCCCGACGCATTCCTCTCCGATGCTACTCGATTTC
GGCCTCTCGTCGACGTCCGCTAAGAAACGCCTTTCGTTTGTACCACACTATCACGAGTGGGTACGTTGCACTAGGGCTGCGTAAGGAGAGGCTACGATGAGCTAAAG
MAPH-1.1
5,780 5,800 5,820 5,840 5,860 5,880

CGCCAAAAGAACGAGGAACAGTTTGCCGCGAATAATCTGCAATTTTCGATTCCCGTGGAGAAGCAACGGACCACAGTGAGCTCGGACGCCGGAGCCATCGAGTATGA
GCGGTTTTCTTGCTCCTTGTCAAACGGCGCTTATTAGACGTTAAAAGCTAAGGGCACCTCTTCGTTGCCTGGTGTCACTCGAGCCTGCGGCCTCGGTAGCTCATACT
MAPH-1.1
5,900 5,920 5,940 5,960 5,980

Pbeta actin eGFP::MAPH-1.1 (9407 bp) (from 5993-7062 bp)

GTTGGCCAGAGTCGATTTGCTCTAAgcggccgccatatgcatcctaggcctattaatattccggagtatacgtagccggctaacaaccggtacctctagataagtaa
CAACCGGTCTCAGCTAAACGAGATTcgccggcggtatacgtaggatccggataattataaggcctcatatgcatcggccgattgttggccatggagatctattcatt
MAPH-1.1
6,000 6,020 6,040 6,060 6,080

tgatcataatcagccatatcacatctgtagaggttttacttgctttaaaaaacctcccacacctccccctgaacctgaaacataaaatgaatgcaattgttgttgtt
actagtattagtcggtatagtgtagacatctccaaaatgaacgaaattttttggagggtgtggagggggacttggactttgtattttacttacgttaacaacaacaa
SV40 late polyA
6,100 6,120 6,140 6,160 6,180 6,200

aacttgtttattgcagcttataatggttacaaataaagcaatagcatcacaaatttcacaaataaagcatttttttcactgcattctagttgtggtttgtccaaact
ttgaacaaataacgtcgaatattaccaatgtttatttcgttatcgtagtgtttaaagtgtttatttcgtaaaaaaagtgacgtaagatcaacaccaaacaggtttga
SV40 late polyA
6,220 6,240 6,260 6,280 6,300

catcaatgtatcttatcatgtctggatcaattcgtaatcatggtcatagctgtttcctgtgtgaaattgttatccgctcacaattccacacaacatacgagccggaa
gtagttacatagaatagtacagacctagttaagcattagtaccagtatcgacaaaggacacactttaacaataggcgagtgttaaggtgtgttgtatgctcggcctt
SV40 late polyA M13-rev LacO
6,320 6,340 6,360 6,380 6,400 6,420

gcataaagtgtaaagcctggggtgcctaatgagtgagctaactcacattaattgcgttgcgctcactgcccgctttccagtcgggaaacctgtcgtgccagctgcat
cgtatttcacatttcggaccccacggattactcactcgattgagtgtaattaacgcaacgcgagtgacgggcgaaaggtcagccctttggacagcacggtcgacgta
6,440 6,460 6,480 6,500 6,520

taatgaatcggccaacgcgcggggagaggcggtttgcgtattgggcgctcttccgcttcctcgctcactgactcgctgcgctcggtcgttcggctgcggcgagcggt
attacttagccggttgcgcgcccctctccgccaaacgcataacccgcgagaaggcgaaggagcgagtgactgagcgacgcgagccagcaagccgacgccgctcgcca
6,540 6,560 6,580 6,600 6,620

atcagctcactcaaaggcggtaatacggttatccacagaatcaggggataacgcaggaaagaacatgtgagcaaaaggccagcaaaaggccaggaaccgtaaaaagg
tagtcgagtgagtttccgccattatgccaataggtgtcttagtcccctattgcgtcctttcttgtacactcgttttccggtcgttttccggtccttggcatttttcc
6,640 6,660 6,680 6,700 6,720 6,740

ccgcgttgctggcgtttttccataggctccgcccccctgacgagcatcacaaaaatcgacgctcaagtcagaggtggcgaaacccgacaggactataaagataccag
ggcgcaacgaccgcaaaaaggtatccgaggcggggggactgctcgtagtgtttttagctgcgagttcagtctccaccgctttgggctgtcctgatatttctatggtc
6,760 6,780 6,800 6,820 6,840

gcgtttccccctggaagctccctcgtgcgctctcctgttccgaccctgccgcttaccggatacctgtccgcctttctcccttcgggaagcgtggcgctttctcatag
cgcaaagggggaccttcgagggagcacgcgagaggacaaggctgggacggcgaatggcctatggacaggcggaaagagggaagcccttcgcaccgcgaaagagtatc
6,860 6,880 6,900 6,920 6,940

ctcacgctgtaggtatctcagttcggtgtaggtcgttcgctccaagctgggctgtgtgcacgaaccccccgttcagcccgaccgctgcgccttatccggtaactatc
gagtgcgacatccatagagtcaagccacatccagcaagcgaggttcgacccgacacacgtgcttggggggcaagtcgggctggcgacgcggaataggccattgatag
6,960 6,980 7,000 7,020 7,040 7,060

gtcttgagtccaacccggtaagacacgacttatcgccactggcagcagccactggtaacaggattagcagagcgaggtatgtaggcggtgctacagagttcttgaag
cagaactcaggttgggccattctgtgctgaatagcggtgaccgtcgtcggtgaccattgtcctaatcgtctcgctccatacatccgccacgatgtctcaagaacttc

7,080 7,100 7,120 7,140 7,160

tggtggcctaactacggctacagtagaaggacagtatttggtatctgcgctctgctgaagccagttaccttcggaaaaagagttggtagctcttgatccggcaaaca
accaccggattgatgccgatgtcatcttcctgtcataaaccatagacgcgagacgacttcggtcaatggaagcctttttctcaaccatcgagaactaggccgtttgt

7,180 7,200 7,220 7,240 7,260

aaccaccgctggtagcggtggtttttttgtttgcaagcagcagattacgcgcagaaaaaaaggatctcaagaagatcctttgatcttttctacggggtctgacgctc
ttggtggcgaccatcgccaccaaaaaaacaaacgttcgtcgtctaatgcgcgtcttttttttcctagagttcttctaggaaactagaaaagatgccccagactgcgag

7,280 7,300 7,320 7,340 7,360 7,380

agtggaacgaaaactcacgttaagggattttggtcatgagattatcaaaaaggatcttcacctagatccttttaaattaaaaatgaagttttaaatcaatctaaagt
tcaccttgcttttgagtgcaattccctaaaaccagtactctaatagtttttcctagaagtggatctaggaaaatttaatttttacttcaaaatttagttagatttca

7,400 7,420 7,440 7,460 7,480

atatatgagtaaacttggtctgacagttaccaatgcttaatcagtgaggcacctatctcagcgatctgtctatttcgttcatccatagttgcctgactccccgtcgt
tatatactcatttgaaccagactgtcaatggttacgaattagtcactccgtggatagagtcgctagacagataaagcaagtaggtatcaacggactgaggggcagca

AmpR

7,500 7,520 7,540 7,560 7,580

gtagataactacgatacgggagggcttaccatctggccccagtgctgcaatgataccgcgagacccacgctcaccggctccagatttatcagcaataaaccagccag
catctattgatgctatgccctcccgaatggtagaccggggtcacgacgttactatggcgctctgggtgcgagtggccgaggtctaaatagtcgttatttggtcggtc

AmpR

7,600 7,620 7,640 7,660 7,680 7,700

ccggaagggccgagcgcagaagtggtcctgcaactttatccgcctccatccagtctattaattgttgccgggaagctagagtaagtagttcgccagttaatagtttg
ggccttcccggctcgcgtcttcaccaggacgttgaaataggcggaggtaggtcagataattaacaacggcccttcgatctcattcatcaagcggtcaattatcaaac

AmpR

7,720 7,740 7,760 7,780 7,800

cgcaacgttgttgccattgctacaggcatcgtggtgtcacgctcgtcgtttggtatggcttcattcagctccggttcccaacgatcaaggcgagttacatgatcccc
gcgttgcaacaacggtaacgatgtccgtagcaccacagtgcgagcagcaaaccataccgaagtaagtcgaggccaagggttgctagttccgctcaatgtactagggg

AmpR

7,820 7,840 7,860 7,880 7,900

catgttgtgcaaaaaagcggttagctccttcggtcctccgatcgttgtcagaagtaagttggccgcagtgttatcactcatggttatggcagcactgcataattctc
gtacaacacgttttttcgccaatcgaggaagccaggaggctagcaacagtcttcattcaaccggcgtcacaatagtgagtaccaataccgtcgtgacgtattaagag

AmpR

7,920 7,940 7,960 7,980 8,000 8,020

Pbeta actin eGFP::MAPH-1.1 (9407 bp) (from 8026-9095 bp)

ttactgtcatgccatccgtaagatgcttttctgtgactggtgagtactcaaccaagtcattctgagaatagtgtatgcggcgaccgagttgctcttgcccggcgtca
aatgacagtacggtaggcattctacgaaaagacactgaccactcatgagttggttcagtaagactcttatcacatacgccgctggctcaacgagaacgggccgcagt

AmpR

8,040 8,060 8,080 8,100 8,120

atacgggataataccgcgccacatagcagaactttaaaagtgctcatcattggaaaacgttcttcggggcgaaaactctcaaggatcttaccgctgttgagatccag
tatgccctattatggcgcggtgtatcgtcttgaaattttcacgagtagtaaccttttgcaagaagccccgcttttgagagttcctagaatggcgacaactctaggtc

AmpR

8,140 8,160 8,180 8,200 8,220

ttcgatgtaacccactcgtgcacccaactgatcttcagcatcttttactttcaccagcgtttctgggtgagcaaaaacaggaaggcaaaatgccgcaaaaaagggaa
aagctacattgggtgagcacgtgggttgactagaagtcgtagaaaatgaaagtggtcgcaaagacccactcgtttttgtccttccgttttacggcgttttttccctt

8,240 8,260 8,280 8,300 8,320 8,340

taagggcgacacggaaatgttgaatactcatactcttcctttttcaatattattgaagcatttatcagggttattgtctcatgagcggatacatatttgaatgtatt
attcccgctgtgcctttacaacttatgagtatgagaaggaaaaagttataataacttcgtaaatagtcccaataacagagtactcgcctatgtataaacttacataa

8,360 8,380 8,400 8,420 8,440

tagaaaaataaacaaataggggttccgcgcacatttccccgaaaagtgccacctgacgtctaagaaaccattattatcatgacattaacctataaaaataggcgtat
atctttttatttgtttatccccaaggcgcgtgtaaaggggcttttcacggtggactgcagattctttggtaataatagtactgtaattggatatttttatccgcata

8,460 8,480 8,500 8,520 8,540 8,560

cacgaggccctttcgtctcgcgcgtttcggtgatgacggtgaaaacctctgacacatgcagctcccggagacggtcacagcttgtctgtaagcggatgccgggagca
gtgctccgggaaagcagagcgcgcaaagccactactgccacttttggagactgtgtacgtcgagggcctctgccagtgtcgaacagacattcgcctacggccctcgt

8,580 8,600 8,620 8,640 8,660

gacaagcccgtcagggcgcgtcagcgggtgttggcgggtgtcggggttggcttaactatgcggcatcagagcagattgtactgagagtgcaccatatgcggtgtgaa
ctgttcgggcagtcccgcgcagtcgcccacaaccgcccacagccccaaccgaattgatacgccgtagtctcgtctaacatgactctcacgtggtatacgccacactt

8,680 8,700 8,720 8,740 8,760

ataccgcacagatgcgtaaggagaaaataccgcatcaggcgccattcgccattcaggctgcgcaactgttgggaagggcgatcggtgcgggcctcttcgctattacg
tatggcgtgtctacgcattcctcttttatggcgtagtccgcggtaagcggtaagtccgacgcgttgacaacccttcccgctagccacgcccggagaagcgataatgc

LacZ alpha

8,780 8,800 8,820 8,840 8,860 8,880

ccagctggcgaaagggggatgtgctgcaaggcgattaagttgggtaacgccagggttttcccagtcacgacgttgtaaaacgacggccagtgccaagttgggatctt
ggtcgaccgctttccccctacacgacgttccgctaattcaacccattgcggtcccaaaagggtcagtgctgcaacattttgctgccggtcacggttcaaccctagaa

M13-fwd

8,900 8,920 8,940 8,960 8,980

tgcattggcccacggctctcaggatggggatgctccccttcagcacccggttccccttggaaactgatggtcctggctctgtggcatggcagtggcactgtgaggag
acgtaaccgggtgccgagagtcctacccctacgaggggaagtcgtgggccaaggggaacctttgactaccaggaccgagacaccgtaccgtcaccgtgacactcctc

9,000 9,020 9,040 9,060 9,080

Pbeta actin eGFP::MAPH-1.1 (9407 bp) (from 9096-9407 bp)

```
cccctaccagcagcacacagtgggtttggcactgccacgctccggatgccgcgctctgatccaaccccataatcaagggaacccgaattgccccatcattgccccca
ggggatggtcgtcgtgtgtcacccaaaccgtgacggtgcgaggcctacggcgcgagactaggttggggtattagttcccttgggcttaacggggtagtaacgggggt
9,100  9,120  9,140  9,160  9,180  9,200

ccacccccatcctgccgggccctcacaccccacgctgccttgtggtgacattccccagcccaaacccacggcttcatggctaccgcggggcatttcccattgccgcc
ggtgggggtaggacggcccgggagtgtggggtgcgacggaacaccactgtaaggggtcgggtttgggtgccgaagtaccgatggcgccccgtaaagggtaacggcgg
9,220  9,240  9,260  9,280  9,300

ccattatcagctctgcacacctcccgctgtacccatgcctcgtggctgcccttctttgacgtataatcttctaattaatacccggccttgtcaaagtg
ggtaatagtcgagacgtgtggagggcgacatgggtacggagcaccgacgggaagaaactgcatattagaagattaattatgggccggaacagtttcac
9,310  9,320  9,330  9,340  9,350  9,360  9,370  9,380  9,390  9,400
```

Pbeta actin DLG-1::mCherry (9662 bp)

ctgcagtgtcaccgcctccgaggtgacagtattgtccctgcggtgtccctgcagctcagctctgtccacagggccacctccagtttggaggggacacaatgcagccc
gacgtcacagtggcggaggctccactgtcataacagggacgccacagggacgtcgagtcgagacaggtgtcccggtggaggtcaaacctcccctgtgttacgtcggg
beta-actin promoter
20 40 60 80 100

cgatgcaacccatcctcgcagcatcccagggacaaagaccccactgcaagaccgcacacagggctgggtcccgctcccctaatatctacagtgcttttgcatggccc
gctacgttgggtaggagcgtcgtagggtccctgtttctggggtgacgttctggcgtgtgtcccgacccagggcgaggggattatagatgtcacgaaaacgtaccggg
beta-actin promoter
120 140 160 180 200

cttaatcaatgcagttaatcagcatgcgctcatgcaccgctctggagctgcaaagcccctcgcagcgctgctcaccaacaccgcgcaccgccccggcccagcctgca
gaattagttacgtcaattagtcgtacgcgagtacgtggcgagacctcgacgtttcggggagcgtcgcgacgagtggttgtggcgcgtggcggggccgggtcggacgt
beta-actin promoter
220 240 260 280 300 320

gcacgcgctgcaaacaggaaagaaacaaaatattgcccaaatgtaggcaaaggcattcggctgccttgacctccgccgggccgggccctgcctgactcagctcctta
cgtgcgcgacgtttgtcctttctttgttttataacgggtttacatccgtttccgtaagccgacggaactggaggcggcccggcccgggacggactgagtcgaggaat
beta-actin promoter
340 360 380 400 420

ctcagcgctcgcttcctccctccggctgccaccgccgcagcgcacaccctgacaaagagtggcccttaacgggctctgaggtgcacccagcagtgcactcagcagtc
gagtcgcgagcgaaggagggaggccgacggtggcggcgtcgcgtgtgggactgtttctcaccgggaattgcccgagactccacgtgggtcgtcacgtgagtcgtcag
beta-actin promoter
440 460 480 500 520

caagggccggcctggaggtttgcaccgctacgtgctgacattagcattgaacttggccctgggtagtgctgcaggccgggcggggtgggtgtagagagtgcagcgcg
gttcccggccggacctccaaacgtggcgatgcacgactgtaatcgtaacttgaaccgggacccatcacgacgtccggcccgccccacccacatctctcacgtcgcgc
beta-actin promoter
540 560 580 600 620 640

cgttgcacccggtgccccttcccctcccttgcatcccagcaggctgcaccccagcaccaggcccgtgcatgcatgctcctggtgttattgcagcctggtgcatgcat
gcaacgtgggccacggggaaggggagggaacgtagggtcgtccgacgtggggtcgtggtccgggcacgtacgtacgaggaccacaataacgtcggaccacgtacgta
beta-actin promoter
660 680 700 720 740

gcgtcttagtggtgcagcgctgtgcatgcatcctccttggtgtgtagcagcttagtgcatgcataccccctcggtgttattgctgctctgtgcaggcacgctcattgt
cgcagaatcaccacgtcgcgacacgtacgtaggaggaaccacacatcgtcgaatcacgtacgtatggggagccacaataacgacgagacacgtccgtgcgagtaaca
beta-actin promoter
760 780 800 820 840

Pbeta actin DLG-1::mCherry (9662 bp) (from 857-1712 bp)

atcacttcatcccagtgcatgcactcacactggagcgattgctgctcggtgcacgcacactcattgtatcacgtcagctcagtggctgcacgcacaccggtgttatt
tagtgaagtagggtcacgtacgtgagtgtgacctcgctaacgacgagccacgtgcgtgtgagtaacatagtgcagtcgagtcaccgacgtgcgtgtggccacaataa
beta-actin promoter
860 880 900 920 940 960

gctgctcggtgcgtgcatgcacatcagtgtcgctgcagctcagtgcatgcacgctcattgcccatcgctatccctgcctctcctgctggcgctccccgggaggtgac
cgacgagccacgcacgtacgtgtagtcacagcgacgtcgagtcacgtacgtgcgagtaacgggtagcgatagggacggagaggacgaccgcgaggggccctccactg
beta-actin promoter
980 1,000 1,020 1,040 1,060

ttcaaggggaccgcaggaccacctcgggggtggggggagggctgcacacgcggaccccgctcccctccccaacaaagcactgtggaatcaaaaaggggggaggggg
aagttcccctggcgtcctggtggagcccccacccccctcccgacgtgtgcgcctggggcgaggggaggggttgtttcgtgacaccttagtttttcccccctccccc
beta-actin promoter
1,080 1,100 1,120 1,140 1,160

gatggaggggcgcgtcacacccccgccccacaccctcacctcgaggtgagccccacgttctgcttcactctccccatctcccccccctccccacccccaattttgta
ctacctccccgcgcagtgtgggggcggggtgtgggagtggagctccactcggggtgcaagacgaagtgagaggggtagaggggggggaggggtgggggttaaaacat
beta-actin promoter
1,180 1,200 1,220 1,240 1,260 1,280

tttatttattttttaattattttgtgcagcgatgggggcggggggggggggggcgcgcgccaggcggggcggggcggggcgaggggcggggcggggcgaggcggaga
aaataaataaaaaattaataaaacacgtcgctacccccgcccccccccccccccgcgcgcggtccgccccgccccgccccgctccccgccccgccccgctccgcctct
beta-actin promoter
1,300 1,320 1,340 1,360 1,380

ggtgcggcggcagccaatcagagcggcgcgctccgaaagtttccttttatggcgaggcggcggcggcggcggccctataaaaagcgaagcgcgcggcgggcgggagt
ccacgccgccgtcggttagtctcgccgcgcgaggctttcaaaggaaaataccgctccgccgccgccgccgccgggatatttttcgcttcgcgcgccgcccgccctca
beta-actin promoter
1,400 1,420 1,440 1,460 1,480

cgctgcgttgccttcgccccgtgccccgctccgcgccgcctcgcgccgcccgccccggctctgactgaccgcgttactcccacaggtgagcgggcgggacggccctt
gcgacgcaacggaagcggggcacggggcgaggcgcggcggagcgcggcgggcggggccgagactgactggcgcaatgagggtgtccactcgcccgccctgccgggaa
beta-actin promoter
1,500 1,520 1,540 1,560 1,580 1,600

ctcctccgggctgtaattagcgcttggtttaatgacggctcgtttcttttctgtggctgcgtgaaagccttaaagggctccgggagggccctttgtgcggggggggag
gaggaggcccgacattaatcgcgaaccaaattactgccgagcaaagaaaagacaccgacgcactttcggaatttcccgaggccctcccgggaaacacgcccccccctc
beta-actin promoter
1,620 1,640 1,660 1,680 1,700

Pbeta actin DLG-1::mCherry (9662 bp) (from 1713-2568 bp)

cggctcgggggggtgcgtgcgtgtgtgtgtgcgtggggagcgccgcgtgcggcccgcgctgcccggcggctgtgagcgctgcgggcgcggcgcggggctttgtgcgct
gccgagcccccccacgcacgcacacacacacgcacccctcgcggcgcacgccgggcgcgacgggccgccgacactcgcgacgcccgcgccgcgccccgaaacacgcga

beta-actin promoter

1,720 1,740 1,760 1,780 1,800

ccgcgtgtgcgcgaggggagcgcggccgggggcggtgccccgcggtgcgggggggctgcgaggggaacaaaggctgcgtgcggggtgtgtgcgtgggggggtgagca
ggcgcacacgcgctcccctcgcgccggcccccgccacggggcgccacgcccccccgacgctccccttgtttccgacgcacgccccacacacgcacccccccactcgt

beta-actin promoter

1,820 1,840 1,860 1,880 1,900 1,920

gggggtgtgggcgcggcggtcgggctgtaacccccccctgcacccccctccccgagttgctgagcacggcccggcttcgggtgcggggctccgtgcggggcgtggcg
cccccacacccgcgccgccagcccgacattggggggggacgtggggggaggggctcaacgactcgtgccgggccgaagcccacgccccgaggcacgccccgcaccgc

beta-actin promoter

1,940 1,960 1,980 2,000 2,020

cggggctcgccgtgccgggcgggggggtggcggcaggtgggggtgccgggcggggcggggccgcctcgggccggggagggctcgggggaggggcgcggcggccccgga
gccccgagcggcacggcccgcccccccaccgccgtccacccccacggcccgccccgccccggcggagcccggcccctcccgagccccctccccgcgccgccggggcct

beta-actin promoter

2,040 2,060 2,080 2,100 2,120 2,140

gcgccggcggctgtcgaggcgcggcgagccgcagccattgccttttatggtaatcgtgcgagagggcgcagggacttcctttgtcccaaatctggcggagccgaaat
cgcggccgccgacagctccgcgccgctcggcgtcggtaacggaaaataccattagcacgctctcccgcgtccctgaaggaaacagggtttagaccgcctcggcttta

beta-actin promoter

2,160 2,180 2,200 2,220 2,240

ctgggaggcgccgccgcaccccctctagcgggcgcgggcgaagcggtgcggcgccggcaggaaggaaatgggcggggagggccttcgtgcgtcgccgcgccgccgtc
gaccctccgcggcggcgtgggggagatcgcccgcgcccgcttcgccacgccgcggccgtccttcctttacccgcccctcccggaagcacgcagcggcgcggcggcag

beta-actin promoter

2,260 2,280 2,300 2,320 2,340

cccttctccatctccagcctcggggctgccgcagggggacggctgccttcgggggggacggggcagggcggggttcggcttctggcgtgtgaccggcggggtttata
gggaagaggtagaggtcggagccccgacggcgtccccctgccgacggaagccccccctgccccgtcccgccccaagccgaagaccgcacactggccgccccaaatat

beta-actin promoter

2,360 2,380 2,400 2,420 2,440 2,460

tcttcccttctctgttcctccgcagcccccaagctagcgctaagcttATGTCCCACGAGTCATCGGAAAAAGCGCATAAAGCGATAGAAAATGTGGAGGACTACTGC
agaagggaagagacaaggaggcgtcgggggttcgatcgcgattcgaaTACAGGGTGCTCAGTAGCCTTTTTCGCGTATTTCGCTATCTTTTACACCTCCTGATGACG

beta-actin promoter DLG-1

2,480 2,500 2,520 2,540 2,560

Pbeta actin DLG-1::mCherry (9662 bp) (from 2569-3424 bp)

CAGACACTGACAAGACACGGCAATGAGGAGCTACGCACAAACCTGGAACGAGTTATCACCACATTCAAATCAAACCTGATGCATTCGTTGCTCGACATTCACGATCT
GTCTGTGACTGTTCTGTGCCGTTACTCCTCGATGCGTGTTTGGACCTTGCTCAATAGTGGTGTAAGTTTAGTTTGGACTACGTAAGCAACGAGCTGTAAGTGCTAGA
DLG-1
2,580 2,600 2,620 2,640 2,660

GTACGAGCAGACGCTGCTCAGCGAGCGAAAATCAGATGCAGAGAAAAATATGGAAGTAAGGCGTGTGATTGAGCGACTCGAAGGTGGACCACACTCCTACAACAGTC
CATGCTCGTCTGCGACGAGTCGCTCGCTTTTAGTCTACGTCTCTTTTTATACCTTCATTCCGCACACTAACTCGCTGAGCTTCCACCTGGTGTGAGGATGTTGTCAG
DLG-1
2,680 2,700 2,720 2,740 2,760 2,780

GTCCGGCTGCGACGACTTCCACGTCGAACTACAACCTTTCAAGCACAACTCCACTTATTTCAGATCTGAGAGATCGGGGAGGTTTCTCGTATTTGAACGGCGGAGGC
CAGGCCGACGCTGCTGAAGGTGCAGCTTGATGTTGGAAAGTTCGTGTTGAGGTGAATAAAGTCTAGACTCTCTAGCCCCTCCAAAGAGCATAAACTTGCCGCCTCCG
DLG-1
2,800 2,820 2,840 2,860 2,880

CTTGGAAACGGGCTTGGGAATGGACTTGGAAACGGACTTCTGTCTTCTCCGTACAACTCATCCAGCACTCACTACCTTCATGAGCGTCAACGTCAGACCAGTCATGA
GAACCTTTGCCCGAACCCTTACCTGAACCTTTGCCTGAAGACAGAAGAGGCATGTTGAGTAGGTCGTGAGTGATGGAAGTACTCGCAGTTGCAGTCTGGTCAGTACT
DLG-1
2,900 2,920 2,940 2,960 2,980

TGGAACATGGCGGGAAACAACCACAAGAACAGTTGACACTCCAAGTGGTCTTGAAAGACGTGTGGTTGAACATACTGGAGTTATCGATGATCATGGTCGTAAATGGG
ACCTTGTACCGCCCTTTGTTGGTGTTCTTGTCAACTGTGAGGTTCACCAGAACTTTCTGCACACCAACTTGTATGACCTCAATAGCTACTAGTACCAGCATTTACCC
DLG-1
3,000 3,020 3,040 3,060 3,080 3,100

AGCTTGAGAATATTGTCTTGGAGAAAGGTCACACCGGCCTTGGATTCTCGATTACTGGAGGTATGGACCAACCAACAGAAGACGGAGATACTTCTATCTATGTCACC
TCGAACTCTTATAACAGAACCTCTTTCCAGTGTGGCCGGAACCTAAGAGCTAATGACCTCCATACCTGGTTGGTTGTCTTCTGCCTCTATGAAGATAGATACAGTGG
DLG-1
3,120 3,140 3,160 3,180 3,200

AATATTATTGAAGGAGGTGCCGCACTTGCTGATGGGCGTATGAGAAAGAATGATATTATCACTGCAGTCAACAATACAAACTGTGAAAATGTGAAGCATGAGGTTGC
TTATAATAACTTCCTCCACGGCGTGAACGACTACCCGCATACTCTTTCTTACTATAATAGTGACGTCAGTTGTTATGTTTGACACTTTTACACTTCGTACTCCAACG
DLG-1
3,220 3,240 3,260 3,280 3,300

AGTCAACGCATTGAAAAGCTCCGGAAACGTTGTTTCGTTGAGTTTGAAACGGCGTAAAGATGAAGCCTTTCTTCCAATTGGAGGAAACTTTGGCGGGTCGACTTCTT
TCAGTTGCGTAACTTTTCGAGGCCTTTGCAACAAAGCAACTCAAACTTTGCCGCATTTCTACTTCGGAAAGAAGGTTAACCTCCTTTGAAACCGCCCAGCTGAAGAA
DLG-1
3,320 3,340 3,360 3,380 3,400 3,420

Pbeta actin DLG-1::mCherry (9662 bp) (from 3425-4280 bp)

ACCTCAGATCAGGAGTCACTCCATCAGTGAGCGCCGGCAACTTGCAACACGCAATTCATTCTCCATCGGCTCCGATTCATCCACCACCGCCACCACCAGTTCATCAC
TGGAGTCTAGTCCTCAGTGAGGTAGTCACTCGCGGCCGTTGAACGTTGTGCGTTAAGTAAGAGGTAGCCGAGGCTAAGTAGGTGGTGGCGGTGGTGGTCAAGTAGTG
DLG-1
3,440 3,460 3,480 3,500 3,520

GGATCATTGAGCCAATTATCTGTTGGCCAATATCGTTCCACACGACCGAATACATCCGTCATTGATCTGGTTAAGGGAGCACGTGGACTTGGTTTCTCTATTGCTGG
CCTAGTAACTCGGTTAATAGACAACCGGTTATAGCAAGGTGTGCTGGCTTATGTAGGCAGTAACTAGACCAATTCCCTCGTGCACCTGAACCAAAGAGATAACGACC
DLG-1
3,540 3,560 3,580 3,600 3,620

TGGTCAAGGAAACGAGCACGTTAAAGGAGATACCGATATCTATGTGACGAAAATCATTGAGGAGGGAGCAGCTGAATTAGACGGAAGATTAAGAGTCGGAGATAAGA
ACCAGTTCCTTTGCTCGTGCAATTTCCTCTATGGCTATAGATACACTGCTTTTAGTAACTCCTCCCTCGTCGACTTAATCTGCCTTCTAATTCTCAGCCTCTATTCT
DLG-1
3,640 3,660 3,680 3,700 3,720 3,740

TTCTTGAAGTTGATCATCACTCTTTGATTAACACGACTCATGAAAATGCGGTCAATGTTCTCAAAAACACTGGAAATCGTGTTCGACTTCTTATTCAGCAAGGAACT
AAGAACTTCAACTAGTAGTGAGAAACTAATTGTGCTGAGTACTTTTACGCCAGTTACAAGAGTTTTTGTGACCTTTAGCACAAGCTGAAGAATAAGTCGTTCCTTGA
DLG-1
3,760 3,780 3,800 3,820 3,840

GGGGCAATTTTCAATGACTCGGCGAGCCAACAATTCATGCCAACTACCCCAATTCTCAGACCTAGCTCCGTTCAAGACTACAACCGCTCTCAAATGGGATCTCAAAG
CCCCGTTAAAAGTTACTGAGCCGCTCGGTTGTTAAGTACGGTTGATGGGGTTAAGAGTCTGGATCGAGGCAAGTTCTGATGTTGGCGAGAGTTTACCCTAGAGTTTC
DLG-1
3,860 3,880 3,900 3,920 3,940

TCATTTGTCTTACGGAGGACCACTGAACACCAGCTACAGTTCACAAGCCCCCATCGCAATTCCACTCGAACCCCGTCCAGTACAACTTGTAAAGGGCCAAAATGGAC
AGTAAACAGAATGCCTCCTGGTGACTTGTGGTCGATGTCAAGTGTTCGGGGGTAGCGTTAAGGTGAGCTTGGGGCAGGTCATGTTGAACATTTCCCGGTTTTACCTG
DLG-1
3,960 3,980 4,000 4,020 4,040 4,060

TTGGATTCAACATTGTTGGCGGAGAAGACAATGAGCCCATCTACATCAGTTTTGTTCTCCCAGGAGGTGTTGCTGATCTTAGTGGAAACGTGAAGACTGGAGACGTT
AACCTAAGTTGTAACAACCGCCTCTTCTGTTACTCGGGTAGATGTAGTCAAAACAAGAGGGTCCTCCACAACGACTAGAATCACCTTTGCACTTCTGACCTCTGCAA
DLG-1
4,080 4,100 4,120 4,140 4,160

CTTCTTGAAGTGAACGGAGTTGTTTTGAGAAATGCAACCCATAAGGAAGCAGCTGAAGCATTGAGAAACGCTGGAAATCCCGTTTATCTCACTCTTCAGTACAGACC
GAAGAACTTCACTTGCCTCAACAAAACTCTTTACGTTGGGTATTCCTTCGTCGACTTCGTAACTCTTTGCGACCTTTAGGGCAAATAGAGTGAGAAGTCATGTCTGG
DLG-1
4,180 4,200 4,220 4,240 4,260 4,280

Pbeta actin DLG-1::mCherry (9662 bp) (from 4281-5136 bp)

ACAAGAGTATCAAATTTTTGAGTCCAAAATTGAGAAGCTGAGAAACGACGTGATTGCTCAATCTAGAATGGGAACTTTGAGCAGAAAGTCTGAATATGTTAGAGCAC
TGTTCTCATAGTTTAAAAACTCAGGTTTTAACTCTTCGACTCTTTGCTGCACTAACGAGTTAGATCTTACCCTTGAAACTCGTCTTTCAGACTTATACAATCTCGTG
DLG-1
4,300 4,320 4,340 4,360 4,380

TTTTCGATTATGATCCATCACGTGAAAACTCTGTTGCTCCCCATAGATCAATGGGATTTAACTATGGTGACATCCTACATATCATCAACTCATCTGACGATGAATGG
AAAAGCTAATACTAGGTAGTGCACTTTTGAGACAACGAGGGGTATCTAGTTACCCTAAATTGATACCACTGTAGGATGTATAGTAGTTGAGTAGACTGCTACTTACC
DLG-1
4,400 4,420 4,440 4,460 4,480

TGGACAGCTAGAAAAGTTCACGAAAACGGAGAAGAAACAGCTGAAGGAGTGATTCCATCAAAGAAACGTGTCGAGAAGAGAGAACGACTTCGTCGGAAGCAAGTCAA
ACCTGTCGATCTTTTCAAGTGCTTTTGCCTCTTCTTTGTCGACTTCCTCACTAAGGTAGTTTCTTTGCACAGCTCTTCTCTCTTGCTGAAGCAGCCTTCGTTCAGTT
DLG-1
4,500 4,520 4,540 4,560 4,580 4,600

CTTCAACTCGGGATCGCAGTCTCTTGGAAGAAACTCGAGCACTACTGGATTGGAAAACCGAAGAGGAAGCCGCAGTCAGCTTTCCTTCTCCAGAAAGTTCCCATTCG
GAAGTTGAGCCCTAGCGTCAGAGAACCTTCTTTGAGCTCGTGATGACCTAACCTTTTGGCTTCTCCTTCGGCGTCAGTCGAAAGGAAGAGGTCTTTCAAGGGTAAGC
DLG-1
4,620 4,640 4,660 4,680 4,700

TCAAAAGTACCGACCGGCTCAACGATCTTAATGAGGAATCCTCAAATGTAGCTGAAGAGCCAGTGTGGTCCTACCAGGCAGTTGAGCAACAGGCGATCAACTACGTT
AGTTTTCATGGCTGGCCGAGTTGCTAGAATTACTCCTTAGGAGTTTACATCGACTTCTCGGTCACACCAGGATGGTCCGTCAACTCGTTGTCCGCTAGTTGATGCAA
DLG-1
4,720 4,740 4,760 4,780 4,800

CGTCCAGTGATCATTCTTGGAGCTCTCAAAGACCGAATCAACGATGAACTTGTCAACCGCGACCCTTCGAAATTCAGTAGCTGCGTTCCACACACTTCCCGACCACC
GCAGGTCACTAGTAAGAACCTCGAGAGTTTCTGGCTTAGTTGCTACTTGAACAGTTGGCGCTGGGAAGCTTTAAGTCATCGACGCAAGGTGTGTGAAGGGCTGGTGG
DLG-1
4,820 4,840 4,860 4,880 4,900 4,920

ACGCGAGGGAGAGGTTAACGGACGTGACTACTACTTTGTCAACAAGCACAATATGGAGGAAGATGTCAAGAATAATCTCTTCATTGAAGCGGGACAATTCCAAAACA
TGCGCTCCCTCTCCAATTGCCTGCACTGATGATGAAACAGTTGTTCGTGTTATACCTCCTTCTACAGTTCTTATTAGAGAAGTAACTTCGCCCTGTTAAGGTTTTGT
DLG-1
4,940 4,960 4,980 5,000 5,020

ATCTCTACGGAACTAGCATTCAAAGCGTCCGAGATGTCGCCAACCAAGGAAGACACTGTATTCTGGACGTCAGTGGAAATGCTATCCGACGACTTCAAAGCAATGCC
TAGAGATGCCTTGATCGTAAGTTTCGCAGGCTCTACAGCGGTTGGTTCCTTCTGTGACATAAGACCTGCAGTCACCTTTACGATAGGCTGCTGAAGTTTCGTTACGG
DLG-1
5,040 5,060 5,080 5,100 5,120

Pbeta actin DLG-1::mCherry (9662 bp) (from 5137-5992 bp)

AATATTCAACCAATCTCGATCTTCATCAAGCCCAGCTCTGCACAACAGATTCTTGAATTGGACAGCCAACTTGCCACCAACCGACAAGACGATCGAGCGATGAGTGG
TTATAAGTTGGTTAGAGCTAGAAGTAGTTCGGGTCGAGACGTGTTGTCTAAGAACTTAACCTGTCGGTTGAACGGTGGTTGGCTGTTCTGCTAGCTCGCTACTCACC
DLG-1
5,140 5,160 5,180 5,200 5,220 5,240

CGAAGAAGCTCAAGCGCAGTATTCCAGATGTCATAGAATTGAGCAAACTTTCGGGGATTTGTTCACGCAAGAAATCTCAAATGTGCACAGCGCGAATGACGTTTTGA
GCTTCTTCGAGTTCGCGTCATAAGGTCTACAGTATCTTAACTCGTTTGAAAGCCCCTAAACAAGTGCGTTCTTTAGAGTTTACACGTGTCGCGCTTACTGCAAAACT
DLG-1
5,260 5,280 5,300 5,320 5,340

GCAAAGTGTACTCCATCATCAGCCGTGAATCGCAGACGCCAATTTGGGTGCCACGTCATaagtcgacagtgagcaagggcgaggaggataacatggccatcatcaag
CGTTTCACATGAGGTAGTAGTCGGCACTTAGCGTCTGCGGTTAAACCCACGGTGCAGTAttcagctgtcactcgttcccgctcctcctattgtaccggtagtagttc
DLG-1 mCherry
5,360 5,380 5,400 5,420 5,440

gagttcatgcgcttcaaggtgcacatggagggctccgtgaacggccacgagttcgagatcgagggcgagggcgagggccgcccctacgagggcacccagaccgccaa
ctcaagtacgcgaagttccacgtgtacctcccgaggcacttgccggtgctcaagctctagctcccgctcccgctcccggcggggatgctcccgtgggtctggcggtt
mCherry
5,460 5,480 5,500 5,520 5,540 5,560

gctgaaggtgaccaagggtggcccccctgcccttcgcctgggacatcctgtcccctcagttcatgtacggctccaaggcctacgtgaagcaccccgccgacatccccg
cgacttccactggttcccaccgggggacgggaagcggaccctgtaggacaggggagtcaagtacatgccgaggttccggatgcacttcgtggggcggctgtaggggc
mCherry
5,580 5,600 5,620 5,640 5,660

actacttgaagctgtccttccccgagggcttcaagtgggagcgcgtgatgaacttcgaggacggcggcgtggtgaccgtgacccaggactcctccctgcaggacggc
tgatgaacttcgacaggaaggggctcccgaagttcaccctcgcgcactacttgaagctcctgccgccgcaccactggcactgggtcctgaggagggacgtcctgccg
mCherry
5,680 5,700 5,720 5,740 5,760

gagttcatctacaaggtgaagctgcgcggcaccaacttcccctccgacggccccgtaatgcagaagaagaccatgggctgggaggcctcctccgagcggatgtaccc
ctcaagtagatgttccacttcgacgcgccgtggttgaaggggaggctgccggggcattacgtcttcttctggtacccgaccctccggaggaggctcgcctacatggg
mCherry
5,780 5,800 5,820 5,840 5,860 5,880

cgaggacggcgccctgaagggcgagatcaagcagaggctgaagctgaaggacggcggccactacgacgctgaggtcaagaccacctacaaggccaagaagcccgtgc
gctcctgccgcgggacttcccgctctagttcgtctccgacttcgacttcctgccgccggtgatgctgcgactccagttctggtggatgttccggttcttcgggcacg
mCherry
5,900 5,920 5,940 5,960 5,980

Pbeta actin DLG-1::mCherry (9662 bp) (from 5993-6955 bp)

agctgcccggcgcctacaacgtcaacatcaagttggacatcacctcccacaacgaggactacaccatcgtggaacagtacgaacgcgccgagggccgccactccacc
tcgacgggccgcggatgttgcagttgtagttcaacctgtagtggagggtgttgctcctgatgtggtagcaccttgtcatgcttgcgcggctcccggcggtgaggtgg

mCherry

6,000 6,020 6,040 6,060 6,080

ggcggcatggacgagctgtacaagTGAgcggccgccatatgcatcctaggcctattaatattccggagtatacgtagccggctaacaaccggtacctctagataagt
ccgccgtacctgctcgacatgttcACTcgccggcggtatacgtaggatccggataattataaggcctcatatgcatcggccgattgttggccatggagatctattca

mCherry

6,100 6,120 6,140 6,160 6,180 6,200

aatgatcataatcagccatatcacatctgtagaggttttacttgctttaaaaaacctcccacacctccccctgaacctgaaacataaaatgaatgcaattgttgttg
ttactagtattagtcggtatagtgtagacatctccaaaatgaacgaaattttttggagggtgtggaggggggacttggactttgtattttacttacgttaacaacaac

SV40 late polyA

6,220 6,240 6,260 6,280 6,300

ttaacttgtttattgcagcttataatggttacaaataaagcaatagcatcacaaatttcacaaataaagcatttttttcactgcattctagttgtggtttgtccaaa
aattgaacaaataacgtcgaatattaccaatgtttatttcgttatcgtagtgtttaaagtgtttatttcgtaaaaaaagtgacgtaagatcaacaccaaacaggttt

SV40 late polyA

6,320 6,340 6,360 6,380 6,400 6,420

ctcatcaatgtatcttatcatgtctggatcaattcgtaatcatggtcatagctgtttcctgtgtgaaattgttatccgctcacaattccacacaacatacgagccgg
gagtagttacatagaatagtacagacctagttaagcattagtaccagtatcgacaaaggacacactttaacaataggcgagtgttaaggtgtgttgtatgctcggcc

SV40 late polyA M13-rev

6,440 6,460 6,480 6,500 6,520

aagcataaagtgtaaagcctggggtgcctaatgagtgagctaactcacattaattgcgttgcgctcactgcccgctttccagtcgggaaacctgtcgtgccagctgc
ttcgtatttcacatttcggaccccacggattactcactcgattgagtgtaattaacgcaacgcgagtgacgggcgaaaggtcagccctttggacagcacggtcgacg

6,540 6,560 6,580 6,600 6,620

attaatgaatcggccaacgcgcggggagaggcggtttgcgtattgggcgctcttccgcttcctcgctcactgactcgctgcgctcggtcgttcggctgcggcgagcg
taattacttagccggttgcgcgcccctctccgccaaacgcataacccgcgagaaggcgaaggagcgagtgactgagcgacgcgagccagcaagccgacgccgctcgc

6,640 6,660 6,680 6,700 6,720 6,740

gtatcagctcactcaaaggcggtaatacggttatccacagaatcaggggataacgcaggaaagaacatgtgagcaaaaggccagcaaaaggccaggaaccgtaaaaa
catagtcgagtgagtttccgccattatgccaataggtgtcttagtcccctattgcgtcctttcttgtacactcgttttccggtcgttttccggtccttggcattttt

6,760 6,780 6,800 6,820 6,840

ggccgcgttgctggcgtttttccataggctccgcccccctgacgagcatcacaaaaatcgacgctcaagtcagaggtggcgaaacccgacaggactataaagatacc
ccggcgcaacgaccgcaaaaaggtatccgaggcggggggactgctcgtagtgtttttagctgcgagttcagtctccaccgctttgggctgtcctgatatttctatgg

6,860 6,880 6,900 6,920 6,940

Pbeta actin DLG-1::mCherry (9662 bp) (from 6956-8025 bp)

```
aggcgtttccccctggaagctccctcgtgcgctctcctgttccgaccctgccgcttaccggatacctgtccgcctttctcccttcgggaagcgtggcgctttctcat
tccgcaaagggggaccttcgagggagcacgcgagaggacaaggctgggacggcgaatggcctatggacaggcggaaagagggaagcccttcgcaccgcgaaagagta
   6,960               6,980               7,000               7,020               7,040               7,060

agctcacgctgtaggtatctcagttcggtgtaggtcgttcgctccaagctgggctgtgtgcacgaaccccccgttcagcccgaccgctgcgccttatccggtaacta
tcgagtgcgacatccatagagtcaagccacatccagcaagcgaggttcgacccgacacacgtgcttggggggcaagtcgggctggcgacgcggaataggccattgat
                 7,080               7,100               7,120               7,140               7,160

tcgtcttgagtccaacccggtaagacacgacttatcgccactggcagcagccactggtaacaggattagcagagcgaggtatgtaggcggtgctacagagttcttga
agcagaactcaggttgggccattctgtgctgaatagcggtgaccgtcgtcggtgaccattgtcctaatcgtctcgctccatacatccgccacgatgtctcaagaact
          7,180               7,200               7,220               7,240               7,260

agtggtggcctaactacggctacagtagaaggacagtatttggtatctgcgctctgctgaagccagttaccttcggaaaaagagttggtagctcttgatccggcaaa
tcaccaccggattgatgccgatgtcatcttcctgtcataaaccatagacgcgagacgacttcggtcaatggaagcctttttctcaaccatcgagaactaggccgttt
  7,280               7,300               7,320               7,340               7,360               7,380

caaaccaccgctggtagcggtggtttttttgtttgcaagcagcagattacgcgcagaaaaaaaggatctcaagaagatcctttgatcttttctacggggtctgacgc
gtttggtggcgaccatcgccaccaaaaaaacaaacgttcgtcgtctaatgcgcgtcttttttttcctagagttcttctaggaaactagaaaagatgccccagactgcg
                7,400               7,420               7,440               7,460               7,480

tcagtggaacgaaaactcacgttaagggattttggtcatgagattatcaaaaaggatcttcacctagatccttttaaattaaaaatgaagttttaaatcaatctaaa
agtcaccttgcttttgagtgcaattccctaaaaccagtactctaatagtttttcctagaagtggatctaggaaaatttaatttttacttcaaaatttagttagattt
         7,500               7,520               7,540               7,560               7,580

gtatatatgagtaaacttggtctgacagttaccaatgcttaatcagtgaggcacctatctcagcgatctgtctatttcgttcatccatagttgcctgactccccgtc
catatatactcatttgaaccagactgtcaatggttacgaattagtcactccgtggatagagtcgctagacagataaagcaagtaggtatcaacggactgaggggcag
                               <------------------------------- AmpR -------------------------------<<
 7,600               7,620               7,640               7,660               7,680               7,700

gtgtagataactacgatacgggagggcttaccatctggccccagtgctgcaatgataccgcgagacccacgctcaccggctccagatttatcagcaataaaccagcc
cacatctattgatgctatgccctcccgaatggtagaccggggtcacgacgttactatggcgctctgggtgcgagtggccgaggtctaaatagtcgttatttggtcgg
<<------------------------------------------------ AmpR ------------------------------------------------<<
              7,720               7,740               7,760               7,780               7,800

agccggaagggccgagcgcagaagtggtcctgcaactttatccgcctccatccagtctattaattgttgccgggaagctagagtaagtagttcgccagttaatagtt
tcggccttcccggctcgcgtcttcaccaggacgttgaaataggcggaggtaggtcagataattaacaacggcccttcgatctcattcatcaagcggtcaattatcaa
<<------------------------------------------------ AmpR ------------------------------------------------<<
       7,820               7,840               7,860               7,880               7,900

tgcgcaacgttgttgccattgctacaggcatcgtggtgtcacgctcgtcgtttggtatggcttcattcagctccggttcccaacgatcaaggcgagttacatgatcc
acgcgttgcaacaacggtaacgatgtccgtagcaccacagtgcgagcagcaaaccataccgaagtaagtcgaggccaagggttgctagttccgctcaatgtactagg
<<------------------------------------------------ AmpR ------------------------------------------------<<
7,920               7,940               7,960               7,980               8,000               8,020
```

Pbeta actin DLG-1::mCherry (9662 bp) (from 8026-9095 bp)

cccatgttgtgcaaaaaagcggttagctccttcggtcctccgatcgttgtcagaagtaagttggccgcagtgttatcactcatggttatggcagcactgcataattc
gggtacaacacgttttttcgccaatcgaggaagccaggaggctagcaacagtcttcattcaaccggcgtcacaatagtgagtaccaataccgtcgtgacgtattaag

AmpR

8,040 8,060 8,080 8,100 8,120

tcttactgtcatgccatccgtaagatgcttttctgtgactggtgagtactcaaccaagtcattctgagaatagtgtatgcggcgaccgagttgctcttgcccggcgt
agaatgacagtacggtaggcattctacgaaaagacactgaccactcatgagttggttcagtaagactcttatcacatacgccgctggctcaacgagaacgggccgca

AmpR

8,140 8,160 8,180 8,200 8,220

caatacgggataataccgcgccacatagcagaactttaaaagtgctcatcattggaaaacgttcttcggggcgaaaactctcaaggatcttaccgctgttgagatcc
gttatgccctattatggcgcggtgtatcgtcttgaaattttcacgagtagtaaccttttgcaagaagccccgcttttgagagttcctagaatggcgacaactctagg

AmpR

8,240 8,260 8,280 8,300 8,320 8,340

agttcgatgtaacccactcgtgcacccaactgatcttcagcatcttttactttcaccagcgtttctgggtgagcaaaaacaggaaggcaaaatgccgcaaaaaaggg
tcaagctacattgggtgagcacgtgggttgactagaagtcgtagaaaatgaaagtggtcgcaaagacccactcgtttttgtccttccgttttacggcgttttttccc

8,360 8,380 8,400 8,420 8,440

aataagggcgacacggaaatgttgaatactcatactcttcctttttcaatattattgaagcatttatcagggttattgtctcatgagcggatacatatttgaatgta
ttattcccgctgtgcctttacaacttatgagtatgagaaggaaaaagttataataacttcgtaaatagtcccaataacagagtactcgcctatgtataaacttacat

8,460 8,480 8,500 8,520 8,540 8,560

tttagaaaaataaacaaataggggttccgcgcacatttccccgaaaagtgccacctgacgtctaagaaaccattattatcatgacattaacctataaaaataggcgt
aaatctttttatttgtttatccccaaggcgcgtgtaaaggggcttttcacggtggactgcagattctttggtaataatagtactgtaattggatatttttatccgca

8,580 8,600 8,620 8,640 8,660

atcacgaggccctttcgtctcgcgcgtttcggtgatgacggtgaaaacctctgacacatgcagctcccggagacggtcacagcttgtctgtaagcggatgccgggag
tagtgctccgggaaagcagagcgcgcaaagccactactgccacttttggagactgtgtacgtcgagggcctctgccagtgtcgaacagacattcgcctacggccctc

8,680 8,700 8,720 8,740 8,760

cagacaagcccgtcagggcgcgtcagcgggtgttggcgggtgtcggggttggcttaactatgcggcatcagagcagattgtactgagagtgcaccatatgcggtgtg
gtctgttcgggcagtcccgcgcagtcgcccacaaccgcccacagccccaaccgaattgatacgccgtagtctcgtctaacatgactctcacgtggtatacgccacac

8,780 8,800 8,820 8,840 8,860 8,880

aaataccgcacagatgcgtaaggagaaaataccgcatcaggcgccattcgccattcaggctgcgcaactgttgggaagggcgatcggtgcgggcctcttcgctatta
tttatggcgtgtctacgcattcctcttttatggcgtagtccgcggtaagcggtaagtccgacgcgttgacaacccttcccgctagccacgcccggagaagcgataat

8,900 8,920 8,940 8,960 8,980

cgccagctggcgaaagggggatgtgctgcaaggcgattaagttgggtaacgccagggttttcccagtcacgacgttgtaaaacgacggccagtgccaagttgggatc
gcggtcgaccgctttccccctacacgacgttccgctaattcaacccattgcggtcccaaaagggtcagtgctgcaacattttgctgccggtcacggttcaaccctag

M13-fwd

9,000 9,020 9,040 9,060 9,080

Pbeta actin DLG-1::mCherry (9662 bp) (from 9096-9662 bp)

```
tttgcattggcccacggctctcaggatggggatgctccccttcagcacccggttccccttggaaactgatggtcctggctctgtggcatggcagtggcactgtgagg
aaacgtaaccgggtgccgagagtcctacccctacgaggggaagtcgtgggccaaggggaacctttgactaccaggaccgagacaccgtaccgtcaccgtgacactcc
   9,100                9,120                9,140                9,160                9,180                9,200

agcccctaccagcagcacacagtgggtttggcactgccacgctccggatgccgcgctctgatccaaccccataatcaagggaacccgaattgccccatcattgcccc
tcggggatggtcgtcgtgtgtcacccaaaccgtgacggtgcgaggcctacggcgcgagactaggttggggtattagttcccttgggcttaacggggtagtaacgggg
               9,220                9,240                9,260                9,280                9,300

caccacccccatcctgccgggccctcacaccccacgctgccttgtggtgacattccccagcccaaacccacggcttcatggctaccgcggggcatttcccattgccg
gtggtgggggtaggacggcccgggagtgtggggtgcgacggaacaccactgtaaggggtcgggtttgggtgccgaagtaccgatggcgccccgtaaagggtaacggc
        9,320                9,340                9,360                9,380                9,400

ccccattatcagctctgcacacctcccgctgtacccatgcctcgtggctgcccttctttgacgtataatcttctaattaatacccggccttgtcaaagtggagcaca
ggggtaatagtcgagacgtgtggagggcgacatgggtacggagcaccgacgggaagaaactgcatattagaagattaattatgggccggaacagtttcacctcgtgt
 9,420                9,440                9,460                9,480                9,500                9,520

aacgttaattaattccccagcaggcaggtaattaacagtgtgactccctttttgctgcgagtggggctgatacagagagatgtggcactatggagcccacggggtcc
ttgcaattaattaaggggtcgtccgtccattaattgtcacactgagggaaaaacgacgctcaccccgactatgtctctctacaccgtgatacctcgggtgccccagg
              9,540                9,560                9,580                9,600                9,620

tggcactgggtgcccacggaggtccccatgtg
accgtgacccacgggtgcctccaggggtacac
         9,640     9,650     9,660
```

pBT331 (Prgef-1::BirA) (8349 bp)

aagcttgcatgcctgcagcgtttccgatacccccttatatcagcacacattcagtcatcataccacaaaatgttgtgatagtgataggcaaaactaccgtatttcct
ttcgaacgtacggacgtcgcaaaggctatgggggaatatagtcgtgtgtaagtcagtagtatggtgttttacaacactatcactatccgttttgatggcataaagga

rgef-1 promoter

20 40 60 80 100

ctattaatcttgcatgcaagactaattttcgattaacccgtaggggtgcaagactaatagagactgcaagactattagaggctgaaatactaattttcgtatgctca
gataattagaacgtacgttctgattaaaagctaattgggcatccccacgttctgattatctctgacgttctgataatctccgactttatgattaaaagcatacgagt

rgef-1 promoter

120 140 160 180 200

ataattttggaaattggcctatttttttgtagaaacttgataccgtttaaacaaggaaaaatacacacttttttaatatttcatcaataatttgaacgattttgtgatt
tattaaaacctttaaccggataaaaaaacatctttgaactatggcaaatttgttcctttttatgtgtgaaaaattataaagtagttattaaacttgctaaaacactaa

rgef-1 promoter

220 240 260 280 300 320

ttaagttcaatttgcccaaaaaaagacaatttttctgacgttaccgcaaagtaatgctggtcaggcaaaaagagcggtgcaaaaatataagagactgcaatactaat
aattcaagttaaacgggtttttttctgttaaaaagactgcaatggcgtttcattacgaccagtccgtttttctcgccacgtttttatattctctgacgttatgatta

rgef-1 promoter

340 360 380 400 420

agaggaaatacggtaattgaattttagtgaaacttgcgacagttttcctcatttttttgttattccggcatacgagtgtggcatacgagtgaggtcatcttttgttt
tctcctttatgccattaacttaaaatcactttgaacgctgtcaaaaggagtaaaaaaacaataaggccgtatgctcacaccgtatgctcactccagtagaaaacaaa

rgef-1 promoter

440 460 480 500 520

cttccgtttcttcatcgcttttgaaaaaaaatgttgaagaaaacttcgtagccgatagccggataatgtttggcacggcgttccaacatatatcattggaaattttt
gaaggcaaagaagtagcgaaaacttttttttacaacttcttttgaagcatcggctatcggcctattacaaaccgtgccgcaaggttgtatatagtaacctttaaaaa

rgef-1 promoter

540 560 580 600 620 640

taagatttttgcgaaaaatcacattcttcacgatgagaacacgttattgaaggaatataaatcaagaataacatatagttatatttctctattactttaacgttaaa
attctaaaaacgctttttagtgtaagaagtgctactcttgtgcaataacttccttatatttagttcttattgtatatcaatataaagagataatgaaattgcaattt

rgef-1 promoter

660 680 700 720 740

tatgagcaaatttgagcattttgattgcgatgaaaagcagaatcgagtcaactgaaatccgttcaaaaacctaagctcggttcgcccgaaagtcgatttttccaata
atactcgtttaaactcgtaaaactaacgctacttttcgtcttagctcagttgactttaggcaagtttttggattcgagccaagcgggctttcagctaaaaaggttat

rgef-1 promoter

760 780 800 820 840

pBT331 (Prgef-1::BirA) (8349 bp) (from 857-1712 bp)

gccgaacagcactgcttctttattttccaggtatttttggggtgtaagaattatatcttctttgtttgggtatttacgtttccgaattccctttcttgatctgtcgc
cggcttgtcgtgacgaagaaataaaaggtccataaaaaccccacattcttaatatagaagaaacaaacccataaatgcaaaggcttaagggaaagaactagacagcg
rgef-1 promoter
860 880 900 920 940 960

tatttttcagaaaccagaacagtttatccttttttcatgagaacctgaatagctcaaaaacccgtttaatttcttttcatcatctcatttgaaactttcctagtcttc
ataaaaagtctttggtcttgtcaaataggaaaaagtactcttggacttatcgagtttttgggcaaattaaagaaaagtagtagagtaaactttgaaaggatcagaag
rgef-1 promoter
980 1,000 1,020 1,040 1,060

ttaatgatttccaggctcctcctacatttgtatctcaagatgcatacactttatttccgctccaattcgaatcatctacttctctttttttcttcaactttctacttt
aattactaaaggtccgaggaggatgtaaacatagagttctacgtatgtgaaataaaggcgaggttaagcttagtagatgaagagaaaaaagaagttgaaagatgaaa
rgef-1 promoter
1,080 1,100 1,120 1,140 1,160

tttccattcttttctttcaaatgttcgacgtcttcaagtgactgctcttcattctcctcatttttctcgaatttcattcttgtctctttttttgctcaaaaatggaaa
aaaggtaagaaaagaaagtttacaagctgcagaagttcactgacgagaagtaagaggagtaaaaagagcttaaagtaagaacagagaaaaaacgagtttttaccttt
rgef-1 promoter
1,180 1,200 1,220 1,240 1,260 1,280

atgaaagtgacgtgagatttggacggcgggacacgggggcagtagaagcagcaaaaaggagagaaagaggacacaaataagaagaacgaattcaaaaataagcggag
tactttcactgcactctaaacctgccgccctgtgcccccgtcatcttcgtcgtttttcctctctttctcctgtgtttattcttcttgcttaagtttttattcgcctc
rgef-1 promoter
1,300 1,320 1,340 1,360 1,380

aggagctatttccgtcaattctacctccccaatcttcatcaattcggctcaattgaatgacgtcacagggagataaaccgtttggatgagcgccgtccatcactgac
tcctcgataaaggcagttaagatggaggggttagaagtagttaagccgagttaacttactgcagtgtccctctatttggcaaacctactcgcggcaggtagtgactg
rgef-1 promoter
1,400 1,420 1,440 1,460 1,480

gccatcccgtttgggacaagaaaagagaaaaaagagcacaaagtttttggtgacggatcttgtcaatcatatgaaagttgttctgattgattgtcagtttttttcct
cggtagggcaaaccctgttcttttctcttttttctcgtgtttcaaaaaccactgcctagaacagttagtatactttcaacaagactaactaacagtcaaaaaaagga
rgef-1 promoter
1,500 1,520 1,540 1,560 1,580 1,600

actttttttggattctatccacttctgaacttttgacaagtttcaaactttctgaatcattttctatgcattttcctggaattctttttatgtaaaatatgaaataga
tgaaaaaaacctaagataggtgaagacttgaaaactgttcaaagtttgaaagacttagtaaaagatacgtaaaaggaccttaagaaaaatacattttatactttatct
rgef-1 promoter
1,620 1,640 1,660 1,680 1,700

pBT331 (Prgef-1::BirA) (8349 bp) (from 1713-2568 bp)

atgtttttgaattcaagttctgctttttttcttctttttttgttctgttcgggcttgggtatgctttttttaaaaattattttgcacatcgaccaataagtgcgcaact
tacaaaaacttaagttcaagacgaaaaaagaagaaaaaacaagacaagcccgaacccatacgaaaaaaatttttaataaaacgtgtagctggttattcacgcgttga

rgef-1 promoter

1,720 1,740 1,760 1,780 1,800

tataaaattaatttatttttgttaatttttgaaatacttgtattgctttaagtgatctgacctcgccctgagctttccacgtagttatcaaatacaaatcctccaag
atattttaattaaataaaaacaattaaaaactttatgaacataacgaaattcactagactggagcgggactcgaaaggtgcatcaatagtttatgtttaggaggttc

rgef-1 promoter

1,820 1,840 1,860 1,880 1,900 1,920

ggtaacgtacctatattactgatctttataataactttatcacctgtccagttccagaggaattctgttaagcttataggcacagaaggagtcatttctgctggttt
ccattgcatggatataatgactagaaatattattgaaatagtggacaggtcaaggtctccttaagacaattcgaatatccgtgtcttcctcagtaaagacgaccaaa

rgef-1 promoter

1,940 1,960 1,980 2,000 2,020

ttaatgatccaaatctttatttcaagtaaaaaactgaacacttgcgaataaaactatcagattaaccattcaccaaaaatgtgtttgaatctaaactttctcagta
aattactaggtttagaaataaagttcattttttgacttgtgaacgcttattttgatagtctaattggtaagtggtttttacacaaacttagatttgaaagagtcat

rgef-1 promoter

2,040 2,060 2,080 2,100 2,120 2,140

ttccaaatatagaaataaataaccacgacattgctaaaatctgtctgaattgtgtactccttaccgtgaagtaataatggatataatgaatcgtttgaaatgaatga
aaggtttatatctttatttattggtgctgtaacgattttagacagacttaacacatgaggaatggcacttcattattacctatattacttagcaaactttacttact

rgef-1 promoter

2,160 2,180 2,200 2,220 2,240

tcagcacatttttggtgaaagatcacaaataaggaataagcgacggaaaataaaacgattatttcggatcaaaaatttgttgaagatcatatacactcgagaccaag
agtcgtgtaaaaaccactttctagtgtttattccttattcgctgccttttattttgctaataaagcctagtttttaaacaacttctagtatatgtgagctctggttc

rgef-1 promoter

2,260 2,280 2,300 2,320 2,340

attattctagacaattttcaaattggcttctttgtttgcaaatcttcataataatctgtgaagtttggaaatttgaatttttaatctttttttcatagattatagttt
taataagatctgttaaaagtttaaccgaagaaacaaacgtttagaagtattattagacacttcaaacctttaaacttaaaaattagaaaaaagtatctaatatcaaa

rgef-1 promoter

2,360 2,380 2,400 2,420 2,440 2,460

ttatttctttgcaaaactatattaaaaaccgatgcattgttttagggaaattaatgagccttttgttcaacactaaaaacaataaaattaaaattttggcttcatca
aataaagaaacgttttgatataatttttggctacgtaacaaaatccctttaattactcggaaaacaagttgtgatttttgttattttaattttaaaaccgaagtagt

rgef-1 promoter

2,480 2,500 2,520 2,540 2,560

pBT331 (Prgef-1::BirA) (8349 bp) (from 2569-3424 bp)

tttgacctttttttaagttcgaaaacttttctcgtatttctgaaccgccaatttttttcacacatctctagacttttggtgcccgttccagaaagttaagtaattgcta
aaactggaaaaaattcaagcttttgaaaagagcataaagacttggcggttaaaaaagtgtgtagagatctgaaaaccacgggcaaggtctttcaattcattaacgat
rgef-1 promoter
2,580 2,600 2,620 2,640 2,660

ttctaagaaagttctcaacattgtttttttagttctgattgaattctgatgttccaggaatatattttaaatttaatattctttgcactatttctatactaactaaat
aagattctttcaagagttgtaacaaaaaatcaagactaacttaagactacaaggtccttatataaaatttaaattataagaaacgtgataaagatatgattgattta
rgef-1 promoter
2,680 2,700 2,720 2,740 2,760 2,780

aataaatagtctaagtatgttcaatgagaacaaaaaagctctatctatatttttctatcctatttcatttatatccttttcattttgaactcaccagcttctcttct
ttatttatcagattcatacaagttactcttgttttttcgagatagatataaaaagataggataaagtaaatataggaaaagtaaaacttgagtggtcgaagagaaga
rgef-1 promoter
2,800 2,820 2,840 2,860 2,880

tcttcttgtccatatacttcttaaaagtctcgaacttctctctctcactctccatcaatttctttatggctaccgcttgcgctctgccgcttccgaagaaagaaggc
agaagaacaggtatatgaagaattttcagagcttgaagagagagagtgagaggtagttaaagaaataccgatggcgaacgcgagacggcgaaggcttctttcttccg
rgef-1 promoter
2,900 2,920 2,940 2,960 2,980

gttggcagagctctcaattctatttttttctcgccgtaggctatcgatttctcagcttctcttctctcttcctcactccaccaccaccacttcgggcttcttctttttc
caaccgtctcgagagttaagataaaaaaagagcggcatccgatagctaaagagtcgaagagaagagagaaggagtgaggtggtggtggtgaagcccgaagaagaaaag
rgef-1 promoter
3,000 3,020 3,040 3,060 3,080 3,100

ttcattttcgtcttcttcttcatcctcttcttttttttttcagttccccctcactcctccccttatatgcgcgtgcgagaggggtgcaaaagcagcgcgcatccgaga
aagtaaaagcagaagaagaagtaggagaagaaaaaaaaaagtcaagggggagtgaggaggggaatatacgcgcacgctctccccacgttttcgtcgcgcgtaggctct
rgef-1 promoter
3,120 3,140 3,160 3,180 3,200

attgaaacgagaaaacggagacgcagcagcagttcgtcctcagaaaaatagccagtaaaaagagaaaaagatagagagagacctatcgcattttattttcaaattgc
taactttgctcttttgcctctgcgtcgtcgtcaagcaggagtctttttatcggtcatttttctctttttctatctctctctggatagcgtaaaataaaagtttaacg
rgef-1 promoter
3,220 3,240 3,260 3,280 3,300

aattcctgcaattcatgtgtgcgtgtgcccattttcaattcttcccgttatttttcagaccattaccaacgttctttctagatcttgagacaatcttccttctgctc
ttaaggacgttaagtacacacgcacacgggtaaaagttaagaagggcaataaaaagtctggtaatggttgcaagaaagatctagaactctgttagaaggaagacgag
rgef-1 promoter
3,320 3,340 3,360 3,380 3,400 3,420

pBT331 (Prgef-1::BirA) (8349 bp) (from 3425-4280 bp)

```
aatcgttcgtcgtgaagacggcatcgacgacgacgacgatcagcagtaaaggatccccgggattggccaaaggacccaaaggtatgtttcgaatgatactaacataa
ttagcaagcagcacttctgccgtagctgctgctgctgctagtcgtcatttcctaggggccctaaccggtttcctgggtttccatacaaagcttactatgattgtatt
```

rgef-1 promoter

3,440 3,460 3,480 3,500 3,520

```
catagaacattttcaggaggacccttgagggtaccggtagaaaaaATGACCGCTCCAAAGAAGAAACGCAAAGTACCGAGCTCAGAAAAGACCGGTATGGAGCAGAA
gtatcttgtaaaagtcctcctgggaactcccatggccatcttttTACTGGCGAGGTTTCTTCTTTGCGTTTCATGGCTCGAGTCTTTTCTGGCCATACCTCGTCTT
```

SV40 NLS

3,540 3,560 3,580 3,600 3,620

```
GCTCATCTCTGAGGAAGACCTCGGAGGAGAGCAGAAACTCATCTCTGAAGAGGACCTCATGAAGGATAATACTGTTCCATTGAAGTTGATTGCTTTGCTTGCAAATG
CGAGTAGAGACTCCTTCTGGAGCCTCCTCTCGTCTTTGAGTAGAGACTTCTCCTGGAGTACTTCCTATTATGACAAGGTAACTTCAACTAACGAAACGAACGTTTAC
```

Myc tag · Myc tag 2 · BirA

3,640 3,660 3,680 3,700 3,720 3,740

```
GAGAATTTCATTCTGGAGAACAACTTGGAGAGACGTTGGGAATGTCGCGTGCTGCAATTAACAAGCATATTCAAACAgtaagtttaaacagttcggtactaactaac
CTCTTAAAGTAAGACCTCTTGTTGAACCTCTCTGCAACCCTTACAGCGCACGACGTTAATTGTTCGTATAAGTTTGTcattcaaatttgtcaagccatgattgattg
```

BirA

3,760 3,780 3,800 3,820 3,840

```
catacatatttaaattttcagTTGAGAGACTGGGGAGTTGACGTTTTTACGGTTCCAGGAAAGGGATATTCGCTTCCAGAGCCAATTCAGCTCTTGAATGCAAAGCA
gtatgtataaatttaaaagtcAACTCTCTGACCCCTCAACTGCAAAAATGCCAAGGTCCTTTCCCTATAAGCGAAGGTCTCGGTTAAGTCGAGAACTTACGTTTCGT
```

BirA

3,860 3,880 3,900 3,920 3,940

```
AATTTTGGGACAGCTTGATGGTGGTAGTGTTGCAGTACTTCCGGTTATAGATAGTACAAATCAGTACTTGCTTGATCGAATAGGAGAATTGAAATCAGGAGATGCTT
TTAAAACCCTGTCGAACTACCACCATCACAACGTCATGAAGGCCAATATCTATCATGTTTAGTCATGAACGAACTAGCTTATCCTCTTAACTTTAGTCCTCTACGAA
```

BirA

3,960 3,980 4,000 4,020 4,040 4,060

```
GCATCGCTGAGTATCAGCAGGCTGGACGAGGACGAAGAGGACGTAAGTGGTTCTgtaagtttaaacatatatatactaactaaccctgattatttaaattttcagCG
CGTAGCGACTCATAGTCGTCCGACCTGCTCCTGCTTCTCCTGCATTCACCAAGAcattcaaatttgtatatatatgattgattgggactaataaatttaaaagtcGC
```

BirA

4,080 4,100 4,120 4,140 4,160

```
CCTTTCGGAGCCAATCTCTATTTGAGTATGTTCTGGCGTCTTGAACAAGGTCCGGCCGCTGCTATCGGACTTTCATTGGTTATTGGAATTGTGATGGCAGAAGTTCT
GGAAAGCCTCGGTTAGAGATAAACTCATACAAGACCGCAGAACTTGTTCCAGGCCGGCGACGATAGCCTGAAAGTAACCAATAACCTTAACACTACCGTCTTCAAGA
```

BirA

4,180 4,200 4,220 4,240 4,260 4,280

pBT331 (Prgef-1::BirA) (8349 bp) (from 4281-5136 bp)

CCGAAAACTTGGTGCAGATAAGGTGAGAGTCAAGTGGCCTAACGATCTTTATCTTCAAGATAGAAAATTGGCCGGAATATTGgtaagtttaaacatatatatactaa
GGCTTTTGAACCACGTCTATTCCACTCTCAGTTCACCGGATTGCTAGAAATAGAAGTTCTATCTTTTAACCGGCCTTATAACcattcaaatttgtatatatatgatt

BirA

4,300 4,320 4,340 4,360 4,380

ctaaccctgattatttaaattttcagGTTGAGCTTACTGGAAAGACGGGAGATGCTGCTCAAATTGTGATTGGAGCTGGTATCAATATGGCAATGAGAAGAGTAGAA
gattgggactaataaatttaaaagtcCAACTCGAATGACCTTTCTGCCCTCTACGACGAGTTTAACACTAACCTCGACCATAGTTATACCGTTACTCTTCTCATCTT

BirA

4,400 4,420 4,440 4,460 4,480

GAGTCTGTTGTTAATCAAGGTTGGATCACACTTCAAGAGGCAGGAATCAACCTTGATAGAAATACGTTGGCCGCCATGCTTATCCGAGAATTGCGAGCTGCACTCGA
CTCAGACAACAATTAGTTCCAACCTAGTGTGAAGTTCTCCGTCCTTAGTTGGAACTATCTTTATGCAACCGGCGGTACGAATAGGCTCTTAACGCTCGACGTGAGCT

BirA

4,500 4,520 4,540 4,560 4,580 4,600

ACTCTTCGAACAAGAAGGTCTTGTAAGTTTAAACATATATATACTAACTAACCCTGATTATTTAAATTTTCAGGCCCCTTACCTCAGTAGATGGGAGAAACTTGACA
TGAGAAGCTTGTTCTTCCAGAACATTCAAATTTGTATATATATGATTGATTGGGACTAATAAATTTAAAAGTCCGGGGAATGGAGTCATCTACCCTCTTTGAACTGT

BirA

4,620 4,640 4,660 4,680 4,700

ATTTTATCAATCGACCAGTGAAGTTGATAATTGGAGATAAGGAAATTTTTGGTATCAGTCGAGGAATTGATAAGCAAGGAGCTCTTCTCCTTGAGCAAGATGGAATC
TAAAATAGTTAGCTGGTCACTTCAACTATTAACCTCTATTCCTTTAAAAACCATAGTCAGCTCCTTAACTATTCGTTCCTCGAGAAGAGGAACTCGTTCTACCTTAG

BirA

4,720 4,740 4,760 4,780 4,800

ATTAAACCATGGATGGGTGGAGAGATTTCTTTGCGATCTGCTGAAAAGTAAgaattccaactgagcgccggtcgctaccattaccaacttgtctggtgtcaaaaata
TAATTTGGTACCTACCCACCTCTCTAAAGAAACGCTAGACGACTTTTCATTcttaaggttgactcgcggccagcgatggtaatggttgaacagaccacagtttttat

BirA unc-54 3'-UTR

4,820 4,840 4,860 4,880 4,900 4,920

ataggggccgctgtcatcagagtaagtttaaactgagttctactaactaacgagtaatatttaaattttcagcatctcgcgcccgtgcctctgacttctaagtccaa
tatccccggcgacagtagtctcattcaaatttgactcaagatgattgattgctcattataaatttaaaagtcgtagagcgcgggcacggagactgaagattcaggtt

unc-54 3'-UTR

4,940 4,960 4,980 5,000 5,020

ttactcttcaacatccctacatgctctttctccctgtgctcccaccccctattttgttattatcaaaaaaacttcttcttaatttctttgtttttagcttctttt
aatgagaagttgtagggatgtacgagaaagagggacacgagggtgggggataaaacaataatagtttttttgaagaagaattaaagaaacaaaaatcgaagaaaa

unc-54 3'-UTR

5,040 5,060 5,080 5,100 5,120

pBT331 (Prgef-1::BirA) (8349 bp) (from 5137-6099 bp)

aagtcacctctaacaatgaaattgtgtagattcaaaaatagaattaattcgtaataaaaagtcgaaaaaaattgtgctccctccccccattaataataattctatcc
ttcagtggagattgttactttaacacatctaagtttttatcttaattaagcattatttttcagctttttttaacacgagggaggggggtaattattattaagatagg

unc-54 3'-UTR

5,140 5,160 5,180 5,200 5,220 5,240

caaaatctacacaatgttctgtgtacacttcttatgtttttttacttctgataaattttttttgaaacatcatagaaaaaaccgcacacaaaataccttatcatat
gttttagatgtgttacaagacacatgtgaagaatacaaaaaaatgaagactatttaaaaaaaactttgtagtatcttttttggcgtgtgttttatggaatagtata

unc-54 3'-UTR

5,260 5,280 5,300 5,320 5,340

gttacgtttcagtttatgaccgcaatttttatttcttcgcacgtctgggcctctcatgacgtcaaatcatgctcatcgtgaaaaagttttggagtatttttggaatt
caatgcaaagtcaaatactggcgttaaaaataaagaagcgtgcagacccggagagtactgcagtttagtacgagtagcactttttcaaaacctcataaaaaccttaa

unc-54 3'-UTR

5,360 5,380 5,400 5,420 5,440

tttcaatcaagtgaaagtttatgaaattaattttcctgcttttgctttttgggggtttcccctattgtttgtcaagagtttcgaggacggcgtttttcttgctaaaa
aaagttagttcactttcaaatactttaattaaaaggacgaaaacgaaaaacccccaaaggggataacaaacagttctcaaagctcctgccgcaaaaagaacgatttt

unc-54 3'-UTR

5,460 5,480 5,500 5,520 5,540 5,560

tcacaagtattgatgagcacgatgcaagaaagatcggaagaaggtttgggtttgaggctcagtggaaggtgagtagaagttgataatttgaaagtggagtagtgtct
agtgttcataactactcgtgctacgttctttctagccttcttccaaacccaaactccgagtcaccttccactcatcttcaactattaaactttcacctcatcacaga

unc-54 3'-UTR

5,580 5,600 5,620 5,640 5,660

atggggtttttgccttaaatgacagaatacattcccaatataccaaacataactgtttcctactagtcggccgtacgggccctttcgtctcgcgcgtttcggtgatg
taccccaaaaacggaatttactgtcttatgtaagggttatatggtttgtattgacaaaggatgatcagccggcatgcccgggaaagcagagcgcgcaaagccactac

unc-54 3'-UTR

5,680 5,700 5,720 5,740 5,760

acggtgaaaacctctgacacatgcagctcccggagacggtcacagcttgtctgtaagcggatgccgggagcagacaagcccgtcagggcgcgtcagcgggtgttggc
tgccacttttggagactgtgtacgtcgagggcctctgccagtgtcgaacagacattcgcctacggccctcgtctgttcgggcagtcccgcgcagtcgcccacaaccg

5,780 5,800 5,820 5,840 5,860 5,880

gggtgtcggggctggcttaactatgcggcatcagagcagattgtactgagagtgcaccatatgcggtgtgaaataccgcacagatgcgtaaggagaaaataccgcat
cccacagccccgaccgaattgatacgccgtagtctcgtctaacatgactctcacgtggtatacgccacactttatggcgtgtctacgcattcctcttttatggcgta

5,900 5,920 5,940 5,960 5,980

caggcggccttaagggcctcgtgatacgcctatttttataggttaatgtcatgataataatggtttcttagacgtcaggtggcacttttcggggaaatgtgcgcgga
gtccgccggaattcccggagcactatgcggataaaaatatccaattacagtactattattaccaaagaatctgcagtccaccgtgaaaagcccctttacacgcgcct

6,000 6,020 6,040 6,060 6,080

pBT331 (Prgef-1::BirA) (8349 bp) (from 6100-7062 bp)

```
acccctatttgtttatttttctaaatacattcaaatatgtatccgctcatgagacaataaccctgataaatgcttcaataatattgaaaaaggaagagtatgagtat
tggggataaacaaataaaaagatttatgtaagtttatacataggcgagtactctgttattgggactatttacgaagttattataactttttccttctcatactcata
6,100  6,120  6,140  6,160  6,180  6,200

tcaacatttccgtgtcgcccttattcccttttttgcggcattttgccttcctgtttttgctcacccagaaacgctggtgaaagtaaaagatgctgaagatcagttgg
agttgtaaaggcacagcgggaataagggaaaaaacgccgtaaaacggaaggacaaaaacgagtgggtctttgcgaccactttcattttctacgacttctagtcaacc
6,220  6,240  6,260  6,280  6,300

gtgcacgagtgggttacatcgaactggatctcaacagcggtaagatccttgagagttttcgccccgaagaacgttttccaatgatgagcacttttaaagttctgcta
cacgtgctcacccaatgtagcttgacctagagttgtcgccattctaggaactctcaaaagcggggcttcttgcaaaaggttactactcgtgaaaatttcaagacgat
AmpR
6,320  6,340  6,360  6,380  6,400  6,420

tgtggcgcggtattatcccgtattgacgccgggcaagagcaactcggtcgccgcatacactattctcagaatgacttggttgagtactcaccagtcacagaaaagca
acaccgcgccataatagggcataactgcggcccgttctcgttgagccagcggcgtatgtgataagagtcttactgaaccaactcatgagtggtcagtgtcttttcgt
AmpR
6,440  6,460  6,480  6,500  6,520

tcttacggatggcatgacagtaagagaattatgcagtgctgccataaccatgagtgataacactgcggccaacttacttctgacaacgatcggaggaccgaaggagc
agaatgcctaccgtactgtcattctcttaatacgtcacgacggtattggtactcactattgtgacgccggttgaatgaagactgttgctagcctcctggcttcctcg
AmpR
6,540  6,560  6,580  6,600  6,620

taaccgcttttttgcacaacatgggggatcatgtaactcgccttgatcgttgggaaccggagctgaatgaagccataccaaacgacgagcgtgacaccacgatgcct
attggcgaaaaaacgtgttgtaccccctagtacattgagcggaactagcaacccttggcctcgacttacttcggtatggtttgctgctcgcactgtggtgctacgga
AmpR
6,640  6,660  6,680  6,700  6,720  6,740

gtagcaatggcaacaacgttgcgcaaactattaactggcgaactacttactctagcttcccggcaacaattaatagactggatggaggcggataaagttgcaggacc
catcgttaccgttgttgcaacgcgtttgataattgaccgcttgatgaatgagatcgaagggccgttgttaattatctgacctacctccgcctatttcaacgtcctgg
AmpR
6,760  6,780  6,800  6,820  6,840

acttctgcgctcggcccttccggctggctggtttattgctgataaatctggagccggtgagcgtgggtctcgcggtatcattgcagcactggggccagatggtaagc
tgaagacgcgagccgggaaggccgaccgaccaaataacgactatttagacctcggccactcgcacccagagcgccatagtaacgtcgtgaccccggtctaccattcg
AmpR
6,860  6,880  6,900  6,920  6,940

cctcccgtatcgtagttatctacacgacggggagtcaggcaactatggatgaacgaaatagacagatcgctgagataggtgcctcactgattaagcattggtaactg
ggagggcatagcatcaatagatgtgctgcccctcagtccgttgatacctacttgctttatctgtctagcgactctatccacggagtgactaattcgtaaccattgac
AmpR
6,960  6,980  7,000  7,020  7,040  7,060
```

pBT331 (Prgef-1::BirA) (8349 bp) (from 7063-8025 bp)

tcagaccaagtttactcatatatactttagattgatttaaaacttcatttttaatttaaaaggatctaggtgaagatcctttttgataatctcatgaccaaaatccc
agtctggttcaaatgagtatatatgaaatctaactaaattttgaagtaaaaattaaattttcctagatccacttctaggaaaaactattagagtactggttttaggg

7,080 7,100 7,120 7,140 7,160

ttaacgtgagttttcgttccactgagcgtcagaccccgtagaaaagatcaaaggatcttcttgagatcctttttttctgcgcgtaatctgctgcttgcaaacaaaaa
aattgcactcaaaagcaaggtgactcgcagtctggggcatcttttctagtttcctagaagaactctaggaaaaaaagacgcgcattagacgacgaacgtttgttttt
ColE1 origin

7,180 7,200 7,220 7,240 7,260

aaccaccgctaccagcggtggtttgtttgccggatcaagagctaccaactctttttccgaaggtaactggcttcagcagagcgcagataccaaatactgtccttcta
ttggtggcgatggtcgccaccaaacaaacggcctagttctcgatggttgagaaaaaggcttccattgaccgaagtcgtctcgcgtctatggtttatgacaggaagat
ColE1 origin

7,280 7,300 7,320 7,340 7,360 7,380

gtgtagccgtagttaggccaccacttcaagaactctgtagcaccgcctacatacctcgctctgctaatcctgttaccagtggctgctgccagtggcgataagtcgtg
cacatcggcatcaatccggtggtgaagttcttgagacatcgtggcggatgtatggagcgagacgattaggacaatggtcaccgacgacggtcaccgctattcagcac
ColE1 origin

7,400 7,420 7,440 7,460 7,480

tcttaccgggttggactcaagacgatagttaccggataaggcgcagcggtcgggctgaacggggggttcgtgcacacagcccagcttggagcgaacgacctacaccg
agaatggcccaacctgagttctgctatcaatggcctattccgcgtcgccagcccgacttgccccccaagcacgtgtgtcgggtcgaacctcgcttgctggatgtggc
ColE1 origin

7,500 7,520 7,540 7,560 7,580

aactgagatacctacagcgtgagcattgagaaagcgccacgcttcccgaagggagaaaggcggacaggtatccggtaagcggcagggtcggaacaggagagcgcacg
ttgactctatggatgtcgcactcgtaactctttcgcggtgcgaagggcttccctctttccgcctgtccataggccattcgccgtcccagccttgtcctctcgcgtgc
ColE1 origin

7,600 7,620 7,640 7,660 7,680 7,700

agggagcttccagggggaaacgcctggtatctttatagtcctgtcgggtttcgccacctctgacttgagcgtcgatttttgtgatgctcgtcaggggggcggagcct
tccctcgaaggtccccctttgcggaccatagaaatatcaggacagcccaaagcggtggagactgaactcgcagctaaaaacactacgagcagtccccccgcctcgga
ColE1 origin

7,720 7,740 7,760 7,780 7,800

atggaaaaacgccagcaacgcggcctttttacggttcctggccttttgctggccttttgctcacatgttctttcctgcgttatcccctgattctgtggataaccgta
tacctttttgcggtcgttgcgccggaaaaatgccaaggaccggaaaacgaccggaaaacgagtgtacaagaaaggacgcaataggggactaagacacctattggcat
ColE1 origin

7,820 7,840 7,860 7,880 7,900

ttaccgcctttgagtgagctgataccgctcgccgcagccgaacgaccgagcgcagcgagtcagtgagcgaggaagcggaagagcgcccaatacgcaaaccgcctctc
aatggcggaaactcactcgactatggcgagcggcgtcggcttgctggctcgcgtcgctcagtcactcgctccttcgccttctcgcgggttatgcgtttggcggagag

7,920 7,940 7,960 7,980 8,000 8,020

pBT331 (Prgef-1::BirA) (8349 bp) (from 8026-8349 bp)

```
cccgcgcgttggccgattcattaatgcagctggcacgacaggtttcccgactggaaagcgggcagtgagcgcaacgcaattaatgtgagttagctcactcattaggc
gggcgcgcaaccggctaagtaattacgtcgaccgtgctgtccaaagggctgacctttcgcccgtcactcgcgttgcgttaattacactcaatcgagtgagtaatccg
         8,040                8,060                8,080                8,100                8,120

accccaggctttacactttatgcttccggctcgtatgttgtgtggaattgtgagcggataacaatttcacacaggaaacagctatgaccatgattacgccaagctgt
tggggtccgaaatgtgaaatacgaaggccgagcatacaacacaccttaacactcgcctattgttaaagtgtgtcctttgtcgatactggtactaatgcggttcgaca
                                          LacO                    M13-rev
    8,140                8,160                8,180                8,200                8,220

aagtttaaacatgatcttactaactaactattctcatttaaattttcagagcttaaaaatggctgaaatcactcacaacgatggatacgctaacaacttggaaatga
ttcaaatttgtactagaatgattgattgataagagtaaatttaaaagtctcgaatttttaccgactttagtgagtgttgctacctatgcgattgttgaacctttact
8,240                8,260                8,280                8,300                8,320                8,340

aat
tta
8,349
```

pJJR50 (3425 bp)

tgggtaacgccagggttttcccagtcacgacgttgtaaaacgacggccagtgaattgtaatacgactcactatagggcgaattgggtaccgggccccccctcgaggt
acccattgcggtcccaaaagggtcagtgctgcaacattttgctgccggtcacttaacattatgctgagtgatatcccgcttaacccatggcccgggggggagctcca

T7
M13-fwd

20 40 60 80 100

cgacggtatcgataagcttgatcaaaaaaaactagcaataaaggaataaaaaactgtacaccttaaaggcgcacactctgttttgcaaattttatttttagttgtga
gctgccatagctattcgaactagttttttttgatcgttatttccttattttttgacatgtggaatttccgcgtgtgagacaaaacgtttaaaataaaaatcaacact

U6 promoter - R07E5.16

120 140 160 180 200

atttctgctgagacctgaaaatagcaactttagtactactataatttgtcaaccttttcaaaaaaagcatgcaatttttgagaaactcttataaaagctattatta
taaagacgactctggacttttatcgttgaaatcatgatgatattaaacagttggaaaagtttttttcgtacgttaaaaactctttgagaatattttcgataataat

U6 promoter - R07E5.16

220 240 260 280 300 320

aaaaaacacctttttttccaaaattattccacaaaaaatatgttatgaaatgcctacaccctctcacacacactctttatactactctgtcaaactcacgagatgtct
ttttttgtggaaaaaaaggttttaataaggtgttttttatacaatactttacggatgtgggagagtgtgtgtgagaaatatgatgagacagtttgagtgctctacaga

U6 promoter - R07E5.16

340 360 380 400 420

gccgcctcttgtgttgcccctatataaacacctcctattgcgagatgtcttATGTCTTCGAGAGAGGAAGACTAGTTTAAGAGCTATGCTGGAAACAGCATAGCAAG
cggcggagaacacaacggggatatatttgtggaggataacgctctacagaaTACAGAAGCTCTCTCCTTCTGATCAAATTCTCGATACGACCTTTGTCGTATCGTTC

U6 promoter - R07E5.16
sgRNA(F+E)

440 460 480 500 520

TTTAAATAAGGCTAGTCCGTTATCAACTTGAAAAAGTGGCACCGAGTCGGTGCTTTTTTTTatcgaattcctgcagcccgggggatccactagttctagagcggccg
AAATTTATTCCGATCAGGCAATAGTTGAACTTTTTCACCGTGGCTCAGCCACGAAAAAAAAtagcttaaggacgtcgggccccctaggtgatcaagatctcgccggc

sgRNA(F+E)

540 560 580 600 620 640

ccaccgcggtggagctccagcttttgttccctttagtgagggttaatttcgagcttggcgtaatcatggtcatagctgtttcctgtgtgaaattgttatccgctcac
ggtggcgccacctcgaggtcgaaaacaagggaaatcactcccaattaaagctcgaaccgcattagtaccagtatcgacaaaggacacactttaacaataggcgagtg

T3
M13-rev
LacO

660 680 700 720 740

aattccacacaacatacgagccggaagcataaagtgtaaagcctggggtgcctaatgagtgagctaactcacattaattgcgttgcgctcactgcccgctttccagt
ttaaggtgtgttgtatgctcggccttcgtatttcacatttcggaccccacggattactcactcgattgagtgtaattaacgcaacgcgagtgacgggcgaaaggtca

760 780 800 820 840

pJJR50 (3425 bp) (from 857-1819 bp)

```
cgggaaacctgtcgtgccagctgcattaatgaatcggccaacgcgcggggagaggcggtttgcgtattgggcgctcttccgcttcctcgctcactgactcgctgcgc
gccctttggacagcacggtcgacgtaattacttagccggttgcgcgcccctctccgccaaacgcataacccgcgagaaggcgaaggagcgagtgactgagcgacgcg
860 880 900 920 940 960

tcggtcgttcggctgcggcgagcggtatcagctcactcaaaggcggtaatacggttatccacagaatcaggggataacgcaggaaagaacatgtgagcaaaaggcca
agccagcaagccgacgccgctcgccatagtcgagtgagtttccgccattatgccaataggtgtcttagtcccctattgcgtcctttcttgtacactcgttttccggt
980 1,000 1,020 1,040 1,060

gcaaaaggccaggaaccgtaaaaaggccgcgttgctggcgtttttccataggctccgcccccctgacgagcatcacaaaaatcgacgctcaagtcagaggtggcgaa
cgttttccggtccttggcatttttccggcgcaacgaccgcaaaaaggtatccgaggcggggggactgctcgtagtgtttttagctgcgagttcagtctccaccgctt
ColE1 origin
1,080 1,100 1,120 1,140 1,160

acccgacaggactataaagataccaggcgtttccccctggaagctccctcgtgcgctctcctgttccgaccctgccgcttaccggatacctgtccgcctttctccct
tgggctgtcctgatatttctatggtccgcaaagggggaccttcgagggagcacgcgagaggacaaggctgggacggcgaatggcctatggacaggcggaaagaggga
ColE1 origin
1,180 1,200 1,220 1,240 1,260 1,280

tcgggaagcgtggcgctttctcatagctcacgctgtaggtatctcagttcggtgtaggtcgttcgctccaagctgggctgtgtgcacgaaccccccgttcagcccga
agcccttcgcaccgcgaaagagtatcgagtgcgacatccatagagtcaagccacatccagcaagcgaggttcgacccgacacacgtgcttggggggcaagtcgggct
ColE1 origin
1,300 1,320 1,340 1,360 1,380

ccgctgcgccttatccggtaactatcgtcttgagtccaacccggtaagacacgacttatcgccactggcagcagccactggtaacaggattagcagagcgaggtatg
ggcgacgcggaataggccattgatagcagaactcaggttgggccattctgtgctgaatagcggtgaccgtcgtcggtgaccattgtcctaatcgtctcgctccatac
ColE1 origin
1,400 1,420 1,440 1,460 1,480

taggcggtgctacagagttcttgaagtggtggcctaactacggctacactagaaggacagtatttggtatctgcgctctgctgaagccagttaccttcggaaaaaga
atccgccacgatgtctcaagaacttcaccaccggattgatgccgatgtgatcttcctgtcataaaccatagacgcgagacgacttcggtcaatggaagcctttttct
ColE1 origin
1,500 1,520 1,540 1,560 1,580 1,600

gttggtagctcttgatccggcaaacaaaccaccgctggtagcggtggtttttttgtttgcaagcagcagattacgcgcagaaaaaaaggatctcaagaagatccttt
caaccatcgagaactaggccgtttgtttggtggcgaccatcgccaccaaaaaaacaaacgttcgtcgtctaatgcgcgtcttttttttcctagagttcttctaggaaa
ColE1 origin
1,620 1,640 1,660 1,680 1,700

gatcttttctacggggtctgacgctcagtggaacgaaaactcacgttaagggattttggtcatgagattatcaaaaaggatcttcacctagatccttttaaattaaa
ctagaaaagatgccccagactgcgagtcaccttgcttttgagtgcaattccctaaaaccagtactctaatagtttttcctagaagtggatctaggaaaatttaattt
ColE1 origin
1,720 1,740 1,760 1,780 1,800
```

pJJR50 (3425 bp) (from 1820-2782 bp)

aatgaagttttaaatcaatctaaagtatatatgagtaaacttggtctgacagttaccaatgcttaatcagtgaggcacctatctcagcgatctgtctatttcgttca
ttacttcaaaatttagttagatttcatatatactcatttgaaccagactgtcaatggttacgaattagtcactccgtggatagagtcgctagacagataaagcaagt

AmpR

1,820 1,840 1,860 1,880 1,900 1,920

tccatagttgcctgactccccgtcgtgtagataactacgatacgggagggcttaccatctggccccagtgctgcaatgataccgcgagacccacgctcaccggctcc
aggtatcaacggactgaggggcagcacatctattgatgctatgccctcccgaatggtagaccggggtcacgacgttactatggcgctctgggtgcgagtggccgagg

AmpR

1,940 1,960 1,980 2,000 2,020

agatttatcagcaataaaccagccagccggaagggccgagcgcagaagtggtcctgcaactttatccgcctccatccagtctattaattgttgccgggaagctagag
tctaaatagtcgttatttggtcggtcggccttcccggctcgcgtcttcaccaggacgttgaaataggcggaggtaggtcagataattaacaacggcccttcgatctc

AmpR

2,040 2,060 2,080 2,100 2,120 2,140

taagtagttcgccagttaatagtttgcgcaacgttgttgccattgctacaggcatcgtggtgtcacgctcgtcgtttggtatggcttcattcagctccggttcccaa
attcatcaagcggtcaattatcaaacgcgttgcaacaacggtaacgatgtccgtagcaccacagtgcgagcagcaaaccataccgaagtaagtcgaggccaagggtt

AmpR

2,160 2,180 2,200 2,220 2,240

cgatcaaggcgagttacatgatcccccatgttgtgcaaaaaagcggttagctccttcggtcctccgatcgttgtcagaagtaagttggccgcagtgttatcactcat
gctagttccgctcaatgtactagggggtacaacacgttttttcgccaatcgaggaagccaggaggctagcaacagtcttcattcaaccggcgtcacaatagtgagta

AmpR

2,260 2,280 2,300 2,320 2,340

ggttatggcagcactgcataattctcttactgtcatgccatccgtaagatgcttttctgtgactggtgagtactcaaccaagtcattctgagaatagtgtatgcggc
ccaataccgtcgtgacgtattaagagaatgacagtacggtaggcattctacgaaaagacactgaccactcatgagttggttcagtaagactcttatcacatacgccg

AmpR

2,360 2,380 2,400 2,420 2,440 2,460

gaccgagttgctcttgcccggcgtcaatacgggataataccgcgccacatagcagaactttaaaagtgctcatcattggaaaacgttcttcggggcgaaaactctca
ctggctcaacgagaacgggccgcagttatgccctattatggcgcggtgtatcgtcttgaaattttcacgagtagtaaccttttgcaagaagccccgcttttgagagt

AmpR

2,480 2,500 2,520 2,540 2,560

aggatcttaccgctgttgagatccagttcgatgtaacccactcgtgcacccaactgatcttcagcatcttttactttcaccagcgtttctgggtgagcaaaaacagg
tcctagaatggcgacaactctaggtcaagctacattgggtgagcacgtgggttgactagaagtcgtagaaaatgaaagtggtcgcaaagacccactcgtttttgtcc

2,580 2,600 2,620 2,640 2,660

aaggcaaaatgccgcaaaaaagggaataagggcgacacggaaatgttgaatactcatactcttcctttttcaatattattgaagcatttatcagggttattgtctca
ttccgttttacggcgttttttcccttattcccgctgtgcctttacaacttatgagtatgagaaggaaaaagttataataacttcgtaaatagtcccaataacagagt

2,680 2,700 2,720 2,740 2,760 2,780

pJJR50 (3425 bp) (from 2783-3425 bp)

```
tgagcggatacatatttgaatgtatttagaaaaataaacaaataggggttccgcgcacatttccccgaaaagtgccacctaaattgtaagcgttaatattttgttaa
actcgcctatgtataaacttacataaatcttttttatttgtttatccccaaggcgcgtgtaaaggggcttttcacggtggatttaacattcgcaattataaaacaatt
        2,800               2,820               2,840               2,860               2,880

aattcgcgttaaatttttgttaaatcagctcattttttaaccaataggccgaaatcggcaaaatcccttataaatcaaaagaatagaccgagatagggttgagtgtt
ttaagcgcaatttaaaaacaatttagtcgagtaaaaaattggttatccggctttagccgttttagggaatatttagttttcttatctggctctatcccaactcacaa
    2,900               2,920               2,940               2,960               2,980

gttccagtttggaacaagagtccactattaaagaacgtggactccaacgtcaaagggcgaaaaaccgtctatcagggcgatggcccactacgtgaaccatcaccctaа
caaggtcaaaccttgttctcaggtgataatttcttgcacctgaggttgcagtttcccgctttttggcagatagtcccgctaccgggtgatgcacttggtagtgggat
F1 ori
3,000               3,020               3,040               3,060               3,080               3,100

atcaagttttttggggtcgaggtgccgtaaagcactaaatcggaaccctaaagggagcccccgatttagagcttgacggggaaagccggcgaacgtggcgagaaagg
tagttcaaaaaaccccagctccacggcatttcgtgatttagccttgggatttccctcgggggctaaatctcgaactgcccctttcggccgcttgcaccgctctttcc
F1 ori
        3,120               3,140               3,160               3,180               3,200

aagggaagaaagcgaaaggagcgggcgctagggcgctggcaagtgtagcggtcacgctgcgcgtaaccaccacacccgccgcgcttaatgcgccgctacagggcgcg
ttcccttctttcgctttcctcgcccgcgatcccgcgaccgttcacatcgccagtgcgacgcgcattggtggtgtgggcggcgcgaattacgcggcgatgtcccgcgc
F1 ori
    3,220               3,240               3,260               3,280               3,300

tcccattcgccattcaggctgcgcaactgttgggaagggcgatcggtgcgggcctcttcgctattacgccagctggcgaaagggggatgtgctgcaaggcgattaag
agggtaagcggtaagtccgacgcgttgacaacccttcccgctagccacgcccggagaagcgataatgcggtcgaccgctttccccctacacgacgttccgctaattc
LacZ alpha
3,320               3,340               3,360               3,380               3,400               3,420

t
a
3,425
```

pMB37 (Prps-27::BirA) (5164 bp)

AAGCTTGGGCTGCAGttcaatcggtttttccttgcttgcgcccaattctgatggttcttccatttctaaattttagtttaataaaaaaatttaaaataatacgagt
TTCGAACCCGACGTCaagttagccaaaaaggaacgaacgcgggttaagactaccaagaaggtaaagatttaaaatcaaattatttttttaaaattttattatgctca
Prps-27
20 40 60 80 100

tttgagactaactttcaataaaaaccaaaaaaaattgcttcaaaaaagagttcgcgccgatttacgataaacacagcgcgctacgacactccgaaaaccgcgccgca
aaactctgattgaaagttatttttggttttttttaacgaagttttttctcaagcgcggctaaatgctatttgtgtcgcgcgatgctgtgaggcttttggcgcggcgt
Prps-27
120 140 160 180 200

ccattacattctccgccccaccgcacggtttgcggaaggcgatgaaattgctgggcctaaaattttaaccctttttttcccgtttttcaactgttttcccgattttt
ggtaatgtaagaggcggggtggcgtgccaaacgccttccgctactttaacgacccggattttaaaattgggaaaaaaagggcaaaaagttgacaaaagggctaaaaa
Prps-27
220 240 260 280 300 320

tcgatatttttttcaggtttttagttagaaaagtatataaaaattgcattatttgtagttttcgctcgtaatttgtgatagaaaacggccaatttgcctagatttgt
agctataaaaaaagtccaaaaatcaatcttttcatatatttttaacgtaataaacatcaaaagcgagcattaaacactatcttttgccggttaaacggatctaaaca
Prps-27
340 360 380 400 420

gattctaccaagtggaatttttgggaaaatatcaatattccacgatttttattcaaatcataacgtttcgaaattatcagcttaaataaaactaaaataaataataa
ctaagatggttcaccttaaaaacccttttatagttataaggtgctaaaaataagtttagtattgcaaagctttaatagtcgaatttattttgattttatttattatt
Prps-27
440 460 480 500 520

aatactaaaaaccctgaatttctcggttctcggttcagcttgagttcgttctatacacgttatcgatttttgtcatttcgtttaatcatgggttttgattcatttta
ttatgatttttgggacttaaagagccaagagccaagtcgaactcaagcaagatatgtgcaatagctaaaaacagtaaagcaaattagtacccaaaactaagtaaaat
Prps-27
540 560 580 600 620 640

aatatgaaatttgatttattcctcgtttcgaagttggtttgtcttgtccggccggtaaacaaatgttattaaaaaaataataaatttcgaaatttttcgtaatgttc
ttatactttaaactaaataaggagcaaagcttcaaccaaacagaacaggccggccatttgtttacaataatttttttattatttaaagctttaaaaagcattacaag
Prps-27
660 680 700 720 740

cgcaatcgtgtttttccgtttatctcgtcttcccttctttcaaatatactatttattttctcaattcacatattttcagccagccccgctcaacaagtggaatGGTA
gcgttagcacaaaaaggcaaatagagcagaagggaagaaagtttatatgataaataaaagagttaagtgtataaaagtcggtcggggcgagttgttcaccttaCCAT
Prps-27
760 780 800 820 840

pMB37 (Prps-27::BirA) (5164 bp) (from 857-1712 bp)

CCaaaaATGGAGCAGAAGCTCATCTCTGAGGAAGACCTCGGAGGAGCAGAAACTCATCTCTGAAGAGGACCTCATGAAGGATAATACTGTTCCATTGAAGTTGAT
GGttttTACCTCGTCTTCGAGTAGAGACTCCTTCTGGAGCCTCCTCTCGTCTTTGAGTAGAGACTTCTCCTGGAGTACTTCCTATTATGACAAGGTAACTTCAACTA
MYC-tag 1 MYC-tag 2 BirA
860 880 900 920 940 960

TGCTTTGCTTGCAAATGGAGAATTTCATTCTGGAGAACAACTTGGAGAGACGTTGGGAATGTCGCGTGCTGCAATTAACAAGCATATTCAAACAgtaagtttaaaca
ACGAAACGAACGTTTACCTCTTAAAGTAAGACCTCTTGTTGAACCTCTCTGCAACCCTTACAGCGCACGACGTTAATTGTTCGTATAAGTTTGTcattcaaatttgt
BirA
980 1,000 1,020 1,040 1,060

gttcggtactaactaaccatacatatttaaattttcagTTGAGAGACTGGGGAGTTGACGTTTTTACGGTTCCAGGAAAGGGATATTCGCTTCCAGAGCCAATTCAG
caagccatgattgattggtatgtataaatttaaaagtcAACTCTCTGACCCCTCAACTGCAAAAATGCCAAGGTCCTTTCCCTATAAGCGAAGGTCTCGGTTAAGTC
BirA
1,080 1,100 1,120 1,140 1,160

CTCTTGAATGCAAAGCAAATTTTGGGACAGCTTGATGGTGGTAGTGTTGCAGTACTTCCGGTTATAGATAGTACAAATCAGTACTTGCTTGATCGAATAGGAGAATT
GAGAACTTACGTTTCGTTTAAAACCCTGTCGAACTACCACCATCACAACGTCATGAAGGCCAATATCTATCATGTTTAGTCATGAACGAACTAGCTTATCCTCTTAA
BirA
1,180 1,200 1,220 1,240 1,260 1,280

GAAATCAGGAGATGCTTGCATCGCTGAGTATCAGCAGGCTGGACGAGGACGAAGAGGACGTAAGTGGTTCTgtaagtttaaacatatatatactaactaaccctgat
CTTTAGTCCTCTACGAACGTAGCGACTCATAGTCGTCCGACCTGCTCCTGCTTCTCCTGCATTCACCAAGAcattcaaatttgtatatatatgattgattgggacta
BirA
1,300 1,320 1,340 1,360 1,380

tatttaaattttcagCGCCTTTCGGAGCCAATCTCTATTTGAGTATGTTCTGGCGTCTTGAACAAGGTCCGGCCGCTGCTATCGGACTTTCATTGGTTATTGGAATT
ataaatttaaaagtcGCGGAAAGCCTCGGTTAGAGATAAACTCATACAAGACCGCAGAACTTGTTCCAGGCCGGCGACGATAGCCTGAAAGTAACCAATAACCTTAA
BirA
1,400 1,420 1,440 1,460 1,480

GTGATGGCAGAAGTTCTCCGAAAACTTGGTGCAGATAAGGTGAGAGTCAAGTGGCCTAACGATCTTTATCTTCAAGATAGAAAATTGGCCGGAATATTGgtaagttt
CACTACCGTCTTCAAGAGGCTTTTGAACCACGTCTATTCCACTCTCAGTTCACCGGATTGCTAGAAATAGAAGTTCTATCTTTTAACCGGCCTTATAACcattcaaa
BirA
1,500 1,520 1,540 1,560 1,580 1,600

aaacatatatatactaactaaccctgattatttaaattttcagGTTGAGCTTACTGGAAAGACGGGAGATGCTGCTCAAATTGTGATTGGAGCTGGTATCAATATGG
tttgtatatatatgattgattgggactaataaatttaaaagtcCAACTCGAATGACCTTTCTGCCCTCTACGACGAGTTTAACACTAACCTCGACCATAGTTATACC
BirA
1,620 1,640 1,660 1,680 1,700

pMB37 (Prps-27::BirA) (5164 bp) (from 1713-2675 bp)

CAATGAGAAGAGTAGAAGAGTCTGTTGTTAATCAAGGTTGGATCACACTTCAAGAGGCAGGAATCAACCTTGATAGAAATACGTTGGCCGCCATGCTTATCCGAGAA
GTTACTCTTCTCATCTTCTCAGACAACAATTAGTTCCAACCTAGTGTGAAGTTCTCCGTCCTTAGTTGGAACTATCTTTATGCAACCGGCGGTACGAATAGGCTCTT

BirA

1,720 1,740 1,760 1,780 1,800

TTGCGAGCTGCACTCGAACTCTTCGAACAAGAAGGTCTTgtaagtttaaacatatatatactaactaaccctgattatttaaattttcagGCCCCTTACCTCAGTAG
AACGCTCGACGTGAGCTTGAGAAGCTTGTTCTTCCAGAAcattcaaatttgtatatatatgattgattgggactaataaatttaaaagtcCGGGGAATGGAGTCATC

BirA BirA

1,820 1,840 1,860 1,880 1,900 1,920

ATGGGAGAAACTTGACAATTTTATCAATCGACCAGTGAAGTTGATAATTGGAGATAAGGAAATTTTTGGTATCAGTCGAGGAATTGATAAGCAAGGAGCTCTTCTCC
TACCCTCTTTGAACTGTTAAAATAGTTAGCTGGTCACTTCAACTATTAACCTCTATTCCTTTAAAAACCATAGTCAGCTCCTTAACTATTCGTTCCTCGAGAAGAGG

BirA

1,940 1,960 1,980 2,000 2,020

TTGAGCAAGATGGAATCATTAAACCATGGATGGGTGGAGAGATTTCTTTGCGATCTGCTGAAAAGTAAGAATTCGCTAGCCGGCCATACAAGTAATCCGGATGATCG
AACTCGTTCTACCTTAGTAATTTGGTACCTACCCACCTCTCTAAAGAAACGCTAGACGACTTTTCATTCTTAAGCGATCGGCCGGTATGTTCATTAGGCCTACTAGC

BirA

2,040 2,060 2,080 2,100 2,120 2,140

ACGCCaACGTCGTTGAATTTTCAAATTTTAAATACTGAATATTTGTTTTTTTTCCTATTATTTATTTATTCTCTTTGTGTTTTTTTTCTTGCTTTCTAAAAAATTAA
TGCGGtTGCAGCAACTTAAAAGTTTAAAATTTATGACTTATAAACAAAAAAAAGGATAATAAATAAATAAGAGAAACACAAAAAAAAGAACGAAAGATTTTTTAATT

let-858 UTR

2,160 2,180 2,200 2,220 2,240

TTCAATCCAAATCTAAACATTTTTTTTTCTCTTTCCGTCTCCCAATTCGTATTCCGCTCCTCTCATCTGAACACAATGTGCAAGTTTATTTATCTTCTCGCTTTCAT
AAGTTAGGTTTAGATTTGTAAAAAAAAAGAGAAAGGCAGAGGGTTAAGCATAAGGCGAGGAGAGTAGACTTGTGTTACACGTTCAAATAAATAGAAGAGCGAAAGTA

let-858 UTR

2,260 2,280 2,300 2,320 2,340

TTCATTAGGACGTGGGGGGAATTGGTGGAAGGGGGAAACACACAAAAGGATGATGGAAATGAAATAAGGACACACAATATGCAACAACATTCAATTCAGAAATATGG
AAGTAATCCTGCACCCCCCTTAACCACCTTCCCCCTTTGTGTGTTTTCCTACTACCTTTACTTTATTCCTGTGTGTTATACGTTGTTGTAAGTTAAGTCTTTATACC

let-858 UTR

2,360 2,380 2,400 2,420 2,440 2,460

AGGAAGGTTTAAAAGAAAACATAAAAATATATAGAGGAGGAAGGAAAACTAGTAAAAAATAAGCAAAGAAATTAGGCGAACGATGAGAATTGTCCTCGCTTgggccc
TCCTTCCAAATTTTCTTTTGTATTTTTATATATCTCCTCCTTCCTTTTGATCATTTTTTATTCGTTTCTTTAATCCGCTTGCTACTCTTAACAGGAGCGAAcccggg

let-858 UTR

2,480 2,500 2,520 2,540 2,560

tttcgtctcgcgcgtttcggtgatgacggtgaaaacctctgacacatgcagctcccggagacggtcacagcttgtctgtaagcggatgccgggagcagacaagcccg
aaagcagagcgcgcaaagccactactgccacttttggagactgtgtacgtcgagggcctctgccagtgtcgaacagacattcgcctacggccctcgtctgttcgggc

2,580 2,600 2,620 2,640 2,660

pMB37 (Prps-27::BirA) (5164 bp) (from 2676-3638 bp)

tcagggcgcgtcagcgggtgttggcgggtgtcggggctggcttaactatgcggcatcagagcagattgtactgagagtgcaccatatgcggtgtgaaataccgcaca
agtcccgcgcagtcgcccacaaccgcccacagccccgaccgaattgatacgccgtagtctcgtctaacatgactctcacgtggtatacgccacactttatggcgtgt

2,680 2,700 2,720 2,740 2,760 2,780

gatgcgtaaggagaaaataccgcatcaggcggccttaagggcctcgtgatacgcctatttttataggttaatgtcatgataataatggtttcttagacgtcaggtgg
ctacgcattcctcttttatggcgtagtccgccggaattcccggagcactatgcggataaaaatatccaattacagtactattattaccaaagaatctgcagtccacc

2,800 2,820 2,840 2,860 2,880

cacttttcggggaaatgtgcgcggaacccctatttgtttatttttctaaatacattcaaatatgtatccgctcatgagacaataaccctgataaatgcttcaataat
gtgaaaagcccctttacacgcgccttggggataaacaaataaaaagatttatgtaagtttatacataggcgagtactctgttattgggactatttacgaagttatta

2,900 2,920 2,940 2,960 2,980

attgaaaaaggaagagtatgagtattcaacatttccgtgtcgcccttattcccttttttgcggcattttgccttcctgtttttgctcacccagaaacgctggtgaaa
taactttttccttctcatactcataagttgtaaaggcacagcgggaataagggaaaaaacgccgtaaaacggaaggacaaaaacgagtgggtctttgcgaccacttt

3,000 3,020 3,040 3,060 3,080 3,100

gtaaaagatgctgaagatcagttgggtgcacgagtgggttacatcgaactggatctcaacagcggtaagatccttgagagttttcgccccgaagaacgttttccaat
cattttctacgacttctagtcaacccacgtgctcacccaatgtagcttgacctagagttgtcgccattctaggaactctcaaaagcggggcttcttgcaaaaggtta

3,120 3,140 3,160 3,180 3,200

gATGAGCACTTTTAAAGTTCTGCTATGTGGCGCGGTATTATCCCGTATTGACGCCGGGCAAGAGCAACTCGGTCGCCGCATACACTATTCTCAGAATGACTTGGTTG
cTACTCGTGAAAATTTCAAGACGATACACCGCGCCATAATAGGGCATAACTGCGGCCCGTTCTCGTTGAGCCAGCGGCGTATGTGATAAGAGTCTTACTGAACCAAC

AmpR

3,220 3,240 3,260 3,280 3,300

AGTACTCACCAGTCACAGAAAAGCATCTTACGGATGGCATGACAGTAAGAGAATTATGCAGTGCTGCCATAACCATGAGTGATAACACTGCGGCCAACTTACTTCTG
TCATGAGTGGTCAGTGTCTTTTCGTAGAATGCCTACCGTACTGTCATTCTCTTAATACGTCACGACGGTATTGGTACTCACTATTGTGACGCCGGTTGAATGAAGAC

AmpR

3,320 3,340 3,360 3,380 3,400 3,420

ACAACGATCGGAGGACCGAAGGAGCTAACCGCTTTTTTGCACAACATGGGGGATCATGTAACTCGCCTTGATCGTTGGGAACCGGAGCTGAATGAAGCCATACCAAA
TGTTGCTAGCCTCCTGGCTTCCTCGATTGGCGAAAAAACGTGTTGTACCCCCTAGTACATTGAGCGGAACTAGCAACCCTTGGCCTCGACTTACTTCGGTATGGTTT

AmpR

3,440 3,460 3,480 3,500 3,520

CGACGAGCGTGACACCACGATGCCTGTAGCAATGGCAACAACGTTGCGCAAACTATTAACTGGCGAACTACTTACTCTAGCTTCCCGGCAACAATTAATAGACTGGA
GCTGCTCGCACTGTGGTGCTACGGACATCGTTACCGTTGTTGCAACGCGTTTGATAATTGACCGCTTGATGAATGAGATCGAAGGGCCGTTGTTAATTATCTGACCT

AmpR

3,540 3,560 3,580 3,600 3,620

pMB37 (Prps-27::BirA) (5164 bp) (from 3639-4494 bp)

TGGAGGCGGATAAAGTTGCAGGACCACTTCTGCGCTCGGCCCTTCCGGCTGGCTGGTTTATTGCTGATAAATCTGGAGCCGGTGAGCGTGGGTCTCGCGGTATCATT
ACCTCCGCCTATTTCAACGTCCTGGTGAAGACGCGAGCCGGGAAGGCCGACCGACCAAATAACGACTATTTAGACCTCGGCCACTCGCACCCAGAGCGCCATAGTAA
AmpR
3,640 3,660 3,680 3,700 3,720 3,740

GCAGCACTGGGGCCAGATGGTAAGCCCTCCCGTATCGTAGTTATCTACACGACGGGGAGTCAGGCAACTATGGATGAACGAAATAGACAGATCGCTGAGATAGGTGC
CGTCGTGACCCCGGTCTACCATTCGGGAGGGCATAGCATCAATAGATGTGCTGCCCCTCAGTCCGTTGATACCTACTTGCTTTATCTGTCTAGCGACTCTATCCACG
AmpR
3,760 3,780 3,800 3,820 3,840

CTCACTGATTAAGCATTGGTAACTGTCAGACCAAGTTTACTCATATATACTTTAGATTGATTTAAAACTTCATTTTTAATTTAAAAGGATCTAGGTGAAGATCCTTT
GAGTGACTAATTCGTAACCATTGACAGTCTGGTTCAAATGAGTATATATGAAATCTAACTAAATTTTGAAGTAAAAATTAAATTTTCCTAGATCCACTTCTAGGAAA
AmpR
3,860 3,880 3,900 3,920 3,940

TTGATAATCTCATGACCAAAATCCCTTAACGTGAGTTTTCGTTCCACTGAGCGTCAGACCCCGTAGAAAAGATCAAAGGATCTTCTTGAGATCCTTTTTTTCTGCGC
AACTATTAGAGTACTGGTTTTAGGGAATTGCACTCAAAAGCAAGGTGACTCGCAGTCTGGGGCATCTTTTCTAGTTTCCTAGAAGAACTCTAGGAAAAAAAGACGCG
ColE1 origin
3,960 3,980 4,000 4,020 4,040 4,060

GTAATCTGCTGCTTGCAAACAAAAAAACCACCGCTACCAGCGGTGGTTTGTTTGCCGGATCAAGAGCTACCAACTCTTTTTCCGAAGGTAACTGGCTTCAGCAGAGC
CATTAGACGACGAACGTTTGTTTTTTTGGTGGCGATGGTCGCCACCAAACAAACGGCCTAGTTCTCGATGGTTGAGAAAAAGGCTTCCATTGACCGAAGTCGTCTCG
ColE1 origin
4,080 4,100 4,120 4,140 4,160

GCAGATACCAAATACTGTCCTTCTAGTGTAGCCGTAGTTAGGCCACCACTTCAAGAACTCTGTAGCACCGCCTACATACCTCGCTCTGCTAATCCTGTTACCAGTGG
CGTCTATGGTTTATGACAGGAAGATCACATCGGCATCAATCCGGTGGTGAAGTTCTTGAGACATCGTGGCGGATGTATGGAGCGAGACGATTAGGACAATGGTCACC
ColE1 origin
4,180 4,200 4,220 4,240 4,260 4,280

CTGCTGCCAGTGGCGATAAGTCGTGTCTTACCGGGTTGGACTCAAGACGATAGTTACCGGATAAGGCGCAGCGGTCGGGCTGAACGGGGGGTTCGTGCACACAGCCC
GACGACGGTCACCGCTATTCAGCACAGAATGGCCCAACCTGAGTTCTGCTATCAATGGCCTATTCCGCGTCGCCAGCCCGACTTGCCCCCCAAGCACGTGTGTCGGG
ColE1 origin
4,300 4,320 4,340 4,360 4,380

AGCTTGGAGCGAACGACCTACACCGAACTGAGATACCTACAGCGTGAGCATTGAGAAAGCGCCACGCTTCCCGAAGGGAGAAAGGCGGACAGGTATCCGGTAAGCGG
TCGAACCTCGCTTGCTGGATGTGGCTTGACTCTATGGATGTCGCACTCGTAACTCTTTCGCGGTGCGAAGGGCTTCCCTCTTTCCGCCTGTCCATAGGCCATTCGCC
ColE1 origin
4,400 4,420 4,440 4,460 4,480

pMB37 (Prps-27::BirA) (5164 bp) (from 4495-5164 bp)

CAGGGTCGGAACAGGAGAGCGCACGAGGGAGCTTCCAGGGGGAAACGCCTGGTATCTTTATAGTCCTGTCGGGTTTCGCCACCTCTGACTTGAGCGTCGATTTTTGT
GTCCCAGCCTTGTCCTCTCGCGTGCTCCCTCGAAGGTCCCCCTTTGCGGACCATAGAAATATCAGGACAGCCCAAAGCGGTGGAGACTGAACTCGCAGCTAAAAACA

ColE1 origin

4,500 4,520 4,540 4,560 4,580 4,600

GATGCTCGTCAGGGGGGCGGAGCCTATGGAAAAACGCCAGCAACGCGGCCTTTTTACGGTTCCTGGCCTTTTGCTGGCCTTTTGCTCACATGTTCTTTCCTGCGTTA
CTACGAGCAGTCCCCCCGCCTCGGATACCTTTTTGCGGTCGTTGCGCCGGAAAAATGCCAAGGACCGGAAAACGACCGGAAAACGAGTGTACAAGAAAGGACGCAAT

ColE1 origin

4,620 4,640 4,660 4,680 4,700

TCCCCTGATTCTGTGGATAACCGTATTACCGCCTTTGAGTGAGCTGATACCGCTCGCCGCAGCCGAACGACCGAGCGCAGCGAGTCAGTGAGCGAGGAAGCGGAAGA
AGGGGACTAAGACACCTATTGGCATAATGGCGGAAACTCACTCGACTATGGCGAGCGGCGTCGGCTTGCTGGCTCGCGTCGCTCAGTCACTCGCTCCTTCGCCTTCT

4,720 4,740 4,760 4,780 4,800

GCGCCCAATACGCAAACCGCCTCTCCCCGCGCGTTGGCCGATTCATTAATGCAGCTGGCACGACAGGTTTCCCGACTGGAAAGCGGGCAGTGAGCGCAACGCAATTA
CGCGGGTTATGCGTTTGGCGGAGAGGGGCGCGCAACCGGCTAAGTAATTACGTCGACCGTGCTGTCCAAAGGGCTGACCTTTCGCCCGTCACTCGCGTTGCGTTAAT

4,820 4,840 4,860 4,880 4,900 4,920

ATGTGAGTTAGCTCACTCATTAGGCACCCCAGGCTTTACACTTTATGCTTCCGGCTCGTATGTTGTGTGGAATTGTGAGCGGATAACAATTTCACACAGGAAACAGC
TACACTCAATCGAGTGAGTAATCCGTGGGGTCCGAAATGTGAAATACGAAGGCCGAGCATACAACACACCTTAACACTCGCCTATTGTTAAAGTGTGTCCTTTGTCG

LacO

4,940 4,960 4,980 5,000 5,020

TATGACCATGATTACGCCAAGCTgtaagtttaaacatgatcttactaactaactattctcatttaaattttcagAGCTTAAAAATGGCTGAAATCACTCACAACGAT
ATACTGGTACTAATGCGGTTCGAcattcaaatttgtactagaatgattgattgataagagtaaatttaaaagtcTCGAATTTTTACCGACTTTAGTGAGTGTTGCTA

5,040 5,060 5,080 5,100 5,120

GGATACGCTAACAACTTGGAAATGAAAT
CCTATGCGATTGTTGAACCTTTACTTTA

5,140 5,150 5,160

pMB41 (N-term Avi-2xTEV-GFP) (5052 bp)

TCGCGCGTTTCGGTGATGACGGTGAAAACCTCTGACACATGCAGCTCCCGGAGACGGTCACAGCTTGTCTGTAAGCGGATGCCGGGAGCAGACAAGCCCGTCAGGGC
AGCGCGCAAAGCCACTACTGCCACTTTTGGAGACTGTGTACGTCGAGGGCCTCTGCCAGTGTCGAACAGACATTCGCCTACGGCCCTCGTCTGTTCGGGCAGTCCCG

20 40 60 80 100

GCGTCAGCGGGTGTTGGCGGGTGTCGGGGCTGGCTTAACTATGCGGCATCAGAGCAGATTGTACTGAGAGTGCACCATATGCGGTGTGAAATACCGCACAGATGCGT
CGCAGTCGCCCACAACCGCCCACAGCCCCGACCGAATTGATACGCCGTAGTCTCGTCTAACATGACTCTCACGTGGTATACGCCACACTTTATGGCGTGTCTACGCA

120 140 160 180 200

AAGGAGAAAATACCGCATCAGGCGCCATTCGCCATTCAGGCTGCGCAACTGTTGGGAAGGGCGATCGGTGCGGGCCTCTTCGCTATTACGCCAGCTGGCGAAAGGGG
TTCCTCTTTTATGGCGTAGTCCGCGGTAAGCGGTAAGTCCGACGCGTTGACAACCCTTCCCGCTAGCCACGCCCGGAGAAGCGATAATGCGGTCGACCGCTTTCCCC

LacZ alpha

220 240 260 280 300 320

GATGTGCTGCAAGGCGATTAAGTTGGGTAACGCCAGGGTTTTCCCAGTCACGACGTTGTAAAACGACGGCCAGTGAATTCGAGCTCttAGATCTGGACTTAATGATA
CTACACGACGTTCCGCTAATTCAACCCATTGCGGTCCCAAAAGGGTCAGTGCTGCAACATTTTGCTGCCGGTCACTTAAGCTCGAGaaTCTAGACCTGAATTACTAT

M13-fwd Avi tag

340 360 380 400 420

TTTTCGAAGCTCAgAAgATTGAATGGCATGAAtctggaggaggatctGAgAAcCTTTAcTTCCAAGGaGAgAAcCTTTAcTTCCAAGGatctggaggaggatctATG
AAAAGCTTCGAGTcTTcTAACTTACCGTACTTagacctcctcctagaCTcTTgGAAATgAAGGTTCCtCTcTTgGAAATgAAGGTTCCtagacctcctcctagaTAC

Avi tag 2 TEV sites

440 460 480 500 520

AGTAAAGGAGAAGAACTTTTCACTGGAGTTGTCCCAATTCTTGTTGAATTAGATGGTGATGTTAATGGGCACAAATTTTCTGTCAGTGGAGAGGGTGAAGGTGATGC
TCATTTCCTCTTCTTGAAAAGTGACCTCAACAGGGTTAAGAACAACTTAATCTACCACTACAATTACCCGTGTTTAAAAGACAGTCACCTCTCCCACTTCCACTACG

GFP

540 560 580 600 620 640

AACATACGGAAAACTTACCCTTAAATTTATTTGCACTACTGGAAAACTACCTGTTCCATGGgtaagtttaaacatatatatactaactaaccctgattatttaaatt
TTGTATGCCTTTTGAATGGGAATTTAAATAAACGTGATGACCTTTTGATGGACAAGGTACCcattcaaatttgtatatatatgattgattgggactaataaatttaa

GFP

660 680 700 720 740

ttcagCCAACACTTGTCACTACTTTCTGTTATGGTGTTCAATGCTTCTCGAGATACCCAGATCATATGAAACGGCATGACTTTTTCAAGAGTGCCATGCCCGAAGGT
aagtcGGTTGTGAACAGTGATGAAAGACAATACCACAAGTTACGAAGAGCTCTATGGGTCTAGTATACTTTGCCGTACTGAAAAAGTTCTCACGGTACGGGCTTCCA

GFP

760 780 800 820 840

TATGTACAGGAAAGAACTATATTTTTCAAAGATGACGGGAACTACAAGACACgtaagtttaaacagttcggtacgaagttcctatactttctagagaataggaactt
ATACATGTCCTTTCTTGATATAAAAAGTTTCTACTGCCCTTGATGTTCTGTGcattcaaatttgtcaagccatgcttcaaggatatgaaagatctcttatccttgaa

GFP FRT site 1

860 880 900 920 940 960

pMB41 (N-term Avi-2xTEV-GFP) (5052 bp) (from 964-1819 bp)

ccctgttgacaattaatcatcggcatagtatatcggcatagtataatacgacaaggtgaggaactaaacccaggaggcagatcatgagtctgaaagaaaaaacacaa
gggacaactgttaattagtagccgtatcatatagccgtatcatattatgctgttccactccttgatttgggtcctccgtctagtactcagactttcttttttgtgtt

galK

980 1,000 1,020 1,040 1,060

tctctgtttgccaacgcatttggctaccctgccactcacaccattcaggcgcctggccgcgtgaatttgattggtgaacacaccgactacaacgacggtttcgttct
agagacaaacggttgcgtaaaccgatgggacggtgagtgtggtaagtccgcggaccggcgcacttaaactaaccacttgtgtggctgatgttgctgccaaagcaaga

galK

1,080 1,100 1,120 1,140 1,160

gccctgcgcgattgattatcaaaccgtgatcagttgtgcaccacgcgatgaccgtaaagttcgcgtgatggcagccgattatgaaaatcagctcgacgagttttccc
cgggacgcgctaactaatagtttggcactagtcaacacgtggtgcgctactggcatttcaagcgcactaccgtcggctaatacttttagtcgagctgctcaaaaggg

galK

1,180 1,200 1,220 1,240 1,260 1,280

tcgatgcgcccattgtcgcacatgaaaactatcaatgggctaactacgttcgtggcgtggtgaaacatctgcaactgcgtaacaacagcttcggcggcgtggacatg
agctacgcgggtaacagcgtgtacttttgatagttacccgattgatgcaagcaccgcaccactttgtagacgttgacgcattgttgtcgaagccgccgcacctgtac

galK

1,300 1,320 1,340 1,360 1,380

gtgatcagcggcaatgtgccgcagggtgccgggttaagttcttccgcttcactggaagtcgcggtcggaaccgtattgcagcagctttatcatctgccgctggacgg
cactagtcgccgttacacggcgtcccacggcccaattcaagaaggcgaagtgaccttcagcgccagccttggcataacgtcgtcgaaatagtagacggcgacctgcc

galK

1,400 1,420 1,440 1,460 1,480

cgcacaaatcgcgcttaacggtcaggaagcagaaaaccagtttgtaggctgtaactgcgggatcatggatcagctaatttccgcgctcggcaagaaagatcatgcct
gcgtgtttagcgcgaattgccagtccttcgtcttttggtcaaacatccgacattgacgccctagtacctagtcgattaaaggcgcgagccgttctttctagtacgga

galK

1,500 1,520 1,540 1,560 1,580 1,600

tgctgatcgattgccgctcactggggaccaaagcagtttccatgcccaaaggtgtggctgtcgtcatcatcaacagtaacttcaaacgtaccctggttggcagcgaa
acgactagctaacggcgagtgacccctggtttcgtcaaaggtacgggtttccacaccgacagcagtagtagttgtcattgaagtttgcatgggaccaaccgtcgctt

galK

1,620 1,640 1,660 1,680 1,700

tacaacacccgtcgtgaacagtgcgaaaccggtgcgcgtttcttccagcagccagccctgcgtgatgtcaccattgaagagttcaacgctgttgcgcatgaactgga
atgttgtgggcagcacttgtcacgctttggccacgcgcaaagaaggtcgtcggtcgggacgcactacagtggtaacttctcaagttgcgacaacgcgtacttgacct

galK

1,720 1,740 1,760 1,780 1,800

pMB41 (N-term Avi-2xTEV-GFP) (5052 bp) (from 1820-2675 bp)

```
cccgatcgtggcaaaacgcgtgcgtcatatactgactgaaaacgcccgcaccgttgaagctgccagcgcgctggagcaaggcgacctgaaacgtatgggcgagttga
gggctagcaccgttttgcgcacgcagtatatgactgacttttgcgggcgtggcaacttcgacggtcgcgcgacctcgttccgctggactttgcatacccgctcaact
galK
1,820  1,840  1,860  1,880  1,900  1,920

tggcggagtctcatgcctctatgcgcgatgatttcgaaatcaccgtgccgcaaattgacactctggtagaaatcgtcaaagctgtgattggcgacaaaggtggcgta
accgcctcagagtacggagatacgcgctactaaagctttagtggcacggcgtttaactgtgagaccatctttagcagtttcgacactaaccgctgtttccaccgcat
galK
1,940  1,960  1,980  2,000  2,020

cgcatgaccggcggcggatttggcggctgtatcgtcgcgctgatcccggaagagctggtgcctgccgtacagcaagctgtcgctgaacaatatgaagcaaaaacagg
gcgtactggccgccgcctaaaccgccgacatagcagcgcgactagggccttctcgaccacggacggcatgtcgttcgacagcgacttgttatacttcgtttttgtcc
galK
2,040  2,060  2,080  2,100  2,120  2,140

tattaaagagactttttacgtttgtaaaccatcacaaggagcaggacagtgctgaggatccactagttctagagcggccgcgaagttcctatactttctagagaata
ataatttctctgaaaaatgcaaacatttggtagtgttcctcgtcctgtcacgactcctaggtgatcaagatctcgccggcgcttcaaggatatgaaagatctcttat
galK  FRT site 2
2,160  2,180  2,200  2,220  2,240

ggaacttctaactaaccatacatatttaaattttcagGTGCTGAAGTCAAGTTTGAAGGTGATACCCTTGTTAATAGAATCGAGTTAAAAGGTATTGATTTTAAAGA
ccttgaagattgattggtatgtataaatttaaaagtcCACGACTTCAGTTCAAACTTCCACTATGGGAACAATTATCTTAGCTCAATTTTCCATAACTAAAATTTCT
GFP
2,260  2,280  2,300  2,320  2,340

AGATGGAAACATTCTTGGACACAAATTGGAATACAACTATAACTCACACAATGTATACATCATGGCAGACAAACAAAAGAATGGAATCAAAGTTgtaagtttaaaca
TCTACCTTTGTAAGAACCTGTGTTTAACCTTATGTTGATATTGAGTGTGTTACATATGTAGTACCGTCTGTTTGTTTTCTTACCTTAGTTTCAAcattcaaatttgt
GFP
2,360  2,380  2,400  2,420  2,440  2,460

tgattttactaactaactaatctgatttaaattttcagAACTTCAAAATTAGACACAACATTGAAGATGGAAGCGTTCAACTAGCAGACCATTATCAACAAAATACT
actaaaatgattgattgattagactaaatttaaaagtcTTGAAGTTTTAATCTGTGTTGTAACTTCTACCTTCGCAAGTTGATCGTCTGGTAATAGTTGTTTTATGA
GFP
2,480  2,500  2,520  2,540  2,560

CCAATTGGCGATGGCCCTGTCCTTTTACCAGACAACCATTACCTGTCCACACAATCTGCCCTTTCGAAAGATCCCAACGAAAAGAGAGACCACATGGTCCTTCTTGA
GGTTAACCGCTACCGGGACAGGAAAATGGTCTGTTGGTAATGGACAGGTGTGTTAGACGGGAAAGCTTTCTAGGGTTGCTTTTCTCTCTGGTGTACCAGGAAGAACT
GFP
2,580  2,600  2,620  2,640  2,660
```

pMB41 (N-term Avi-2xTEV-GFP) (5052 bp) (from 2676-3638 bp)

GTTTGTAACAGCTGCTGGGATTACACATGGCATGGATGAACTATACAAAggaggaggatctggaggaggaggatctggaggaggaggtTCCGGATTCCATCGGATCC
CAAACATTGTCGACGACCCTAATGTGTACCGTACCTACTTGATATGTTTcctcctcctagacctcctcctcctagacctcctcctccaAGGCCTAAGGTAGCCTAGG

GFP Flexible linker

2,680 2,700 2,720 2,740 2,760 2,780

CGGGCCCGTCGACTGCAGAGGCCTGCATGCAAGCTTGGCGTAATCATGGTCATAGCTGTTTCCTGTGTGAAATTGTTATCCGCTCACAATTCCACACAACATACGAG
GCCCGGGCAGCTGACGTCTCCGGACGTACGTTCGAACCGCATTAGTACCAGTATCGACAAAGGACACACTTTAACAATAGGCGAGTGTTAAGGTGTGTTGTATGCTC

M13-rev LacO

2,800 2,820 2,840 2,860 2,880

CCGGAAGCATAAAGTGTAAAGCCTGGGGTGCCTAATGAGTGAGCTAACTCACATTAATTGCGTTGCGCTCACTGCCCGCTTTCCAGTCGGGAAACCTGTCGTGCCAG
GGCCTTCGTATTTCACATTTCGGACCCCACGGATTACTCACTCGATTGAGTGTAATTAACGCAACGCGAGTGACGGGCGAAAGGTCAGCCCTTTGGACAGCACGGTC

2,900 2,920 2,940 2,960 2,980

CTGCATTAATGAATCGGCCAACGCGCGGGGAGAGGCGGTTTGCGTATTGGGCGCTCTTCCGCTTCCTCGCTCACTGACTCGCTGCGCTCGGTCGTTCGGCTGCGGCG
GACGTAATTACTTAGCCGGTTGCGCGCCCCTCTCCGCCAAACGCATAACCCGCGAGAAGGCGAAGGAGCGAGTGACTGAGCGACGCGAGCCAGCAAGCCGACGCCGC

3,000 3,020 3,040 3,060 3,080 3,100

AGCGGTATCAGCTCACTCAAAGGCGGTAATACGGTTATCCACAGAATCAGGGGATAACGCAGGAAAGAACATGTGAGCAAAAGGCCAGCAAAAGGCCAGGAACCGTA
TCGCCATAGTCGAGTGAGTTTCCGCCATTATGCCAATAGGTGTCTTAGTCCCCTATTGCGTCCTTTCTTGTACACTCGTTTTCCGGTCGTTTTCCGGTCCTTGGCAT

3,120 3,140 3,160 3,180 3,200

AAAAGGCCGCGTTGCTGGCGTTTTTCCATAGGCTCCGCCCCCCTGACGAGCATCACAAAAATCGACGCTCAAGTCAGAGGTGGCGAAACCCGACAGGACTATAAAGA
TTTTCCGGCGCAACGACCGCAAAAAGGTATCCGAGGCGGGGGGACTGCTCGTAGTGTTTTTAGCTGCGAGTTCAGTCTCCACCGCTTTGGGCTGTCCTGATATTTCT

ColE1 origin

3,220 3,240 3,260 3,280 3,300

TACCAGGCGTTTCCCCCTGGAAGCTCCCTCGTGCGCTCTCCTGTTCCGACCCTGCCGCTTACCGGATACCTGTCCGCCTTTCTCCCTTCGGGAAGCGTGGCGCTTTC
ATGGTCCGCAAAGGGGGACCTTCGAGGGAGCACGCGAGAGGACAAGGCTGGGACGGCGAATGGCCTATGGACAGGCGGAAAGAGGGAAGCCCTTCGCACCGCGAAAG

ColE1 origin

3,320 3,340 3,360 3,380 3,400 3,420

TCATAGCTCACGCTGTAGGTATCTCAGTTCGGTGTAGGTCGTTCGCTCCAAGCTGGGCTGTGTGCACGAACCCCCCGTTCAGCCCGACCGCTGCGCCTTATCCGGTA
AGTATCGAGTGCGACATCCATAGAGTCAAGCCACATCCAGCAAGCGAGGTTCGACCCGACACACGTGCTTGGGGGGCAAGTCGGGCTGGCGACGCGGAATAGGCCAT

ColE1 origin

3,440 3,460 3,480 3,500 3,520

ACTATCGTCTTGAGTCCAACCCGGTAAGACACGACTTATCGCCACTGGCAGCAGCCACTGGTAACAGGATTAGCAGAGCGAGGTATGTAGGCGGTGCTACAGAGTTC
TGATAGCAGAACTCAGGTTGGGCCATTCTGTGCTGAATAGCGGTGACCGTCGTCGGTGACCATTGTCCTAATCGTCTCGCTCCATACATCCGCCACGATGTCTCAAG

ColE1 origin

3,540 3,560 3,580 3,600 3,620

pMB41 (N-term Avi-2xTEV-GFP) (5052 bp) (from 3639-4494 bp)

TTGAAGTGGTGGCCTAACTACGGCTACACTAGAAGAACAGTATTTGGTATCTGCGCTCTGCTGAAGCCAGTTACCTTCGGAAAAAGAGTTGGTAGCTCTTGATCCGG
AACTTCACCACCGGATTGATGCCGATGTGATCTTCTTGTCATAAACCATAGACGCGAGACGACTTCGGTCAATGGAAGCCTTTTTCTCAACCATCGAGAACTAGGCC

ColE1 origin

3,640 3,660 3,680 3,700 3,720 3,740

CAAACAAACCACCGCTGGTAGCGGTGGTTTTTTTGTTTGCAAGCAGCAGATTACGCGCAGAAAAAAAGGATCTCAAGAAGATCCTTTGATCTTTTCTACGGGGTCTG
GTTTGTTTGGTGGCGACCATCGCCACCAAAAAAACAAACGTTCGTCGTCTAATGCGCGTCTTTTTTTCCTAGAGTTCTTCTAGGAAACTAGAAAAGATGCCCCAGAC

ColE1 origin

3,760 3,780 3,800 3,820 3,840

ACGCTCAGTGGAACGAAAACTCACGTTAAGGGATTTTGGTCATGAGATTATCAAAAAGGATCTTCACCTAGATCCTTTTAAATTAAAAATGAAGTTTTAAATCAATC
TGCGAGTCACCTTGCTTTTGAGTGCAATTCCCTAAAACCAGTACTCTAATAGTTTTTCCTAGAAGTGGATCTAGGAAAATTTAATTTTTACTTCAAAATTTAGTTAG

ColE1 origin

3,860 3,880 3,900 3,920 3,940

TAAAGTATATATGAGTAAACTTGGTCTGACAGTTACCAATGCTTAATCAGTGAGGCACCTATCTCAGCGATCTGTCTATTTCGTTCATCCATAGTTGCCTGACTCCC
ATTTCATATATACTCATTTGAACCAGACTGTCAATGGTTACGAATTAGTCACTCCGTGGATAGAGTCGCTAGACAGATAAAGCAAGTAGGTATCAACGGACTGAGGG

AmpR

3,960 3,980 4,000 4,020 4,040 4,060

CGTCGTGTAGATAACTACGATACGGGAGGGCTTACCATCTGGCCCCAGTGCTGCAATGATACCGCGAGACCCACGCTCACCGGCTCCAGATTTATCAGCAATAAACC
GCAGCACATCTATTGATGCTATGCCCTCCCGAATGGTAGACCGGGGTCACGACGTTACTATGGCGCTCTGGGTGCGAGTGGCCGAGGTCTAAATAGTCGTTATTTGG

AmpR

4,080 4,100 4,120 4,140 4,160

AGCCAGCCGGAAGGGCCGAGCGCAGAAGTGGTCCTGCAACTTTATCCGCCTCCATCCAGTCTATTAATTGTTGCCGGGAAGCTAGAGTAAGTAGTTCGCCAGTTAAT
TCGGTCGGCCTTCCCGGCTCGCGTCTTCACCAGGACGTTGAAATAGGCGGAGGTAGGTCAGATAATTAACAACGGCCCTTCGATCTCATTCATCAAGCGGTCAATTA

AmpR

4,180 4,200 4,220 4,240 4,260 4,280

AGTTTGCGCAACGTTGTTGCCATTGCTACAGGCATCGTGGTGTCACGCTCGTCGTTTGGTATGGCTTCATTCAGCTCCGGTTCCCAACGATCAAGGCGAGTTACATG
TCAAACGCGTTGCAACAACGGTAACGATGTCCGTAGCACCACAGTGCGAGCAGCAAACCATACCGAAGTAAGTCGAGGCCAAGGGTTGCTAGTTCCGCTCAATGTAC

AmpR

4,300 4,320 4,340 4,360 4,380

ATCCCCCATGTTGTGCAAAAAAGCGGTTAGCTCCTTCGGTCCTCCGATCGTTGTCAGAAGTAAGTTGGCCGCAGTGTTATCACTCATGGTTATGGCAGCACTGCATA
TAGGGGGTACAACACGTTTTTTCGCCAATCGAGGAAGCCAGGAGGCTAGCAACAGTCTTCATTCAACCGGCGTCACAATAGTGAGTACCAATACCGTCGTGACGTAT

AmpR

4,400 4,420 4,440 4,460 4,480

pMB41 (N-term Avi-2xTEV-GFP) (5052 bp) (from 4495-5052 bp)

ATTCTCTTACTGTCATGCCATCCGTAAGATGCTTTTCTGTGACTGGTGAGTACTCAACCAAGTCATTCTGAGAATAGTGTATGCGGCGACCGAGTTGCTCTTGCCCG
TAAGAGAATGACAGTACGGTAGGCATTCTACGAAAAGACACTGACCACTCATGAGTTGGTTCAGTAAGACTCTTATCACATACGCCGCTGGCTCAACGAGAACGGGC

AmpR

4,500 4,520 4,540 4,560 4,580 4,600

GCGTCAATACGGGATAATACCGCGCCACATAGCAGAACTTTAAAAGTGCTCATCATTGGAAAACGTTCTTCGGGGCGAAAACTCTCAAGGATCTTACCGCTGTTGAG
CGCAGTTATGCCCTATTATGGCGCGGTGTATCGTCTTGAAATTTTCACGAGTAGTAACCTTTTGCAAGAAGCCCCGCTTTTGAGAGTTCCTAGAATGGCGACAACTC

AmpR

4,620 4,640 4,660 4,680 4,700

ATCCAGTTCGATGTAACCCACTCGTGCACCCAACTGATCTTCAGCATCTTTTACTTTCACCAGCGTTTCTGGGTGAGCAAAAACAGGAAGGCAAAATGCCGCAAAAA
TAGGTCAAGCTACATTGGGTGAGCACGTGGGTTGACTAGAAGTCGTAGAAAATGAAAGTGGTCGCAAAGACCCACTCGTTTTTGTCCTTCCGTTTTACGGCGTTTTT

4,720 4,740 4,760 4,780 4,800

AGGGAATAAGGGCGACACGGAAATGTTGAATACTCATACTCTTCCTTTTTCAATATTATTGAAGCATTTATCAGGGTTATTGTCTCATGAGCGGATACATATTTGAA
TCCCTTATTCCCGCTGTGCCTTTACAACTTATGAGTATGAGAAGGAAAAAGTTATAATAACTTCGTAAATAGTCCCAATAACAGAGTACTCGCCTATGTATAAACTT

4,820 4,840 4,860 4,880 4,900 4,920

TGTATTTAGAAAAATAAACAAATAGGGGTTCCGCGCACATTTCCCCGAAAAGTGCCACCTGACGTCTAAGAAACCATTATTATCATGACATTAACCTATAAAAATAG
ACATAAATCTTTTTATTTGTTTATCCCCAAGGCGCGTGTAAAGGGGCTTTTCACGGTGGACTGCAGATTCTTTGGTAATAATAGTACTGTAATTGGATATTTTTATC

4,940 4,960 4,980 5,000 5,020

GCGTATCACGAGGCCCTTTCGTC
CGCATAGTGCTCCGGGAAAGCAG

5,030 5,040 5,050

pMB43 (4876 bp)

AAGCTTGGGCTGCAGttcaatcggtttttccttgcttgcgcccaattctgatggttcttccatttctaaattttagtttaataaaaaaatttttaaaataatacgagt
TTCGAACCCGACGTCaagttagccaaaaaggaacgaacgcgggttaagactaccaagaaggtaaagatttaaaatcaaattatttttttaaaattttattatgctca

Prps-27

20 40 60 80 100

tttgagactaactttcaataaaaaccaaaaaaaattgcttcaaaaaagagttcgcgccgatttacgataaacacagcgcgctacgacactccgaaaaccgcgccgca
aaactctgattgaaagttatttttggttttttttaacgaagttttttctcaagcgcggctaaatgctatttgtgtcgcgcgatgctgtgaggcttttggcgcggcgt

Prps-27

120 140 160 180 200

ccattacattctccgccccaccgcacggtttgcggaaggcgatgaaattgctgggcctaaaattttaacccttttttcccgtttttcaactgttttcccgattttt
ggtaatgtaagaggcggggtggcgtgccaaacgccttccgctactttaacgacccggattttaaaattgggaaaaaagggcaaaaagttgacaaaagggctaaaaa

Prps-27

220 240 260 280 300 320

tcgatattttttttcaggtttttagttagaaaagtatataaaaattgcattatttgtagttttcgctcgtaatttgtgatagaaaacggccaatttgcctagatttgt
agctataaaaaaagtccaaaaatcaatcttttcatatatttttaacgtaataaacatcaaaagcgagcattaaacactatcttttgccggttaaacggatctaaaca

Prps-27

340 360 380 400 420

gattctaccaagtggaatttttgggaaaatatcaatattccacgatttttattcaaatcataacgtttcgaaattatcagcttaaataaaactaaaataaataataa
ctaagatggttcaccttaaaaacccttttatagttataaggtgctaaaaataagtttagtattgcaaagctttaatagtcgaatttattttgattttatttattatt

Prps-27

440 460 480 500 520

aatactaaaaaccctgaatttctcggttctcggttcagcttgagttcgttctatacacgttatcgatttttgtcatttcgtttaatcatgggttttgattcatttta
ttatgatttttgggacttaaagagccaagagccaagtcgaactcaagcaagatatgtgcaatagctaaaaacagtaaagcaaattagtacccaaaactaagtaaaat

Prps-27

540 560 580 600 620 640

aatatgaaatttgatttattcctcgtttcgaagttggtttgtcttgtccggccggtaaacaaatgttattaaaaaaataataaatttcgaaatttttcgtaatgttc
ttatactttaaactaaataaggagcaaagcttcaaccaaacagaacaggccggccatttgtttacaataatttttttattatttaaagctttaaaaagcattacaag

Prps-27

660 680 700 720 740

cgcaatcgtgtttttccgtttatctcgtcttcccttctttcaaatatactatttattttctcaattcacatattttcagccagccccgctcaacaagtggaatGGTA
gcgttagcacaaaaaggcaaatagagcagaagggaagaaagtttatatgataaataaaagagttaagtgtataaaagtcggtcggggcgagttgttcaccttaCCAT

Prps-27

760 780 800 820 840

pMB43 (4876 bp) (from 857-1712 bp)

CCGGTAGAAAAAATGAGTAAAGGAGAAGAACTTTTCACTGGAGTTGTCCCAATTCTTGTTGAATTAGATGGTGATGTTAATGGGCACAAATTTTCTGTCAGTGGAGA
GGCCATCTTTTTTACTCATTTCCTCTTCTTGAAAAGTGACCTCAACAGGGTTAAGAACAACTTAATCTACCACTACAATTACCCGTGTTTAAAAGACAGTCACCTCT
GFP
860 880 900 920 940 960

GGGTGAAGGTGATGCAACATACGGAAAACTTACCCTTAAATTTATTTGCACTACTGGAAAACTACCTGTTCCATGGgtaagtttaaacatatatatactaactaacc
CCCACTTCCACTACGTTGTATGCCTTTTGAATGGGAATTTAAATAAACGTGATGACCTTTTGATGGACAAGGTACCcattcaaatttgtatatatatgattgattgg
GFP
980 1,000 1,020 1,040 1,060

ctgattatttaaattttcagccaacacttgtcactactttctgttatggtgttcaatgcttctcgagatacccagatcatatgaaacggcatgactttttcaagagt
gactaataaatttaaaagtcggttgtgaacagtgatgaaagacaataccacaagttacgaagagctctatgggtctagtatactttgccgtactgaaaaagttctca
GFP
1,080 1,100 1,120 1,140 1,160

gccatgcccgaaggttatgtacaggaaagaactatatttttcaaagatgacgggaactacaagacacgtaagtttaaacagttcggtacgaagttcctatactttct
cggtacgggcttccaatacatgtcctttcttgatataaaaagtttctactgcccttgatgttctgtgcattcaaatttgtcaagccatgcttcaaggatatgaaaga
GFP FRT site
1,180 1,200 1,220 1,240 1,260 1,280

agagaataggaacttctaactaaccatacatatttaaattttcaggtgctgaagtcaagtttgaaggtgatacccttgttaatagaatcgagttaaaaggtattgat
tctcttatccttgaagattgattggtatgtataaatttaaaagtccacgacttcagttcaaacttccactatgggaacaattatcttagctcaattttccataacta
FRT site GFP
1,300 1,320 1,340 1,360 1,380

tttaaagaagatggaaacattcttggacacaaattggaatacaactataactcacacaatgtatacatcatggcagacaaacaaaagaatggaatcaaagttgtaag
aaatttcttctacctttgtaagaacctgtgtttaaccttatgttgatattgagtgtgttacatatgtagtaccgtctgtttgttttcttaccttagtttcaacattc
GFP
1,400 1,420 1,440 1,460 1,480

tttaaacatgattttactaactaactaatctgatttaaattttcagaacttcaaaattagacacaacattgaagatggaagcgttcaactagcagaccattatcaac
aaatttgtactaaaatgattgattgattagactaaatttaaaagtcttgaagttttaatctgtgttgtaacttctaccttcgcaagttgatcgtctggtaatagttg
GFP
1,500 1,520 1,540 1,560 1,580 1,600

aaaatactcCAATTGGCGATGGCCCTGTCCTTTTACCAGACAACCATTACCTGTCCACACAATCTGCCCTTTCGAAAGATCCCAACGAAAAGAGAGACCACATGGTC
ttttatgagGTTAACCGCTACCGGGACAGGAAAATGGTCTGTTGGTAATGGACAGGTGTGTTAGACGGGAAAGCTTTCTAGGGTTGCTTTTCTCTCTGGTGTACCAG
GFP GFP
1,620 1,640 1,660 1,680 1,700

pMB43 (4876 bp) (from 1713-2675 bp)

CTTCTTGAGTTTGTAACAGCTGCTGGGATTACACATGGCATGGATGAACTATACAAAtctggaggaggatctGAAAATCTTTATTTCCAAGGAtccGAgAAcCTTTA
GAAGAACTCAAACATTGTCGACGACCCTAATGTGTACCGTACCTACTTGATATGTTTagacctcctcctagaCTTTTAGAAATAAAGGTTCCTaggCTcTTgGAAAT

GFP
2 TEV sites

1,720 1,740 1,760 1,780 1,800

cTTCCAAGGAtctggaggaggatctGGACTTAATGATATTTTCGAAGCTCAgAAgATTGAATGGCATGAAtaaGCTAGCatgcaagatcctttcaagcattcccttc
gAAGGTTCCTagacctcctcctagaCCTGAATTACTATAAAAGCTTCGAGTcTTcTAACTTACCGTACTTattCGATCGtacgttctaggaaagttcgtaagggaag

Avi tag
tbb-2 UTR

1,820 1,840 1,860 1,880 1,900 1,920

ttctctatcactcttctttctttttgtcaaaaaattctctcgctaatttatttgctttttttaatgttattattttatgactttttatagtcactgaaaagtttgcat
aagagatagtgagaagaaagaaaaacagtttttttaagagagcgattaaataaacgaaaaaattacaataataaaatactgaaaaatatcagtgacttttcaaacgta

tbb-2 UTR

1,940 1,960 1,980 2,000 2,020

ctgagtgaagtgaatgctatcaaaatgtgattctgtctgatgtactttcacaatctctcttcaattccatttgaagtgctttaaacccgaaaggttgagaaaaatg
gactcacttcacttacgatagttttacactaagacagactacatgaaagtgttagagagaagttaaggtaaaacttcacgaaatttgggctttccaactctttttac

tbb-2 UTR

2,040 2,060 2,080 2,100 2,120 2,140

cgagcgctcaaatatttgtattgtgttcgttgagtgacccaacaaaaagaggaaactttattgtgccgccaagaaaaaagtctcattgcctaagagaaacatatgtg
gctcgcgagtttataaacataacacaagcaactcactgggttgtttttctcctttgaaataacacggcggttcttttttcagagtaacggattctctttgtatacac

tbb-2 UTR

2,160 2,180 2,200 2,220 2,240

gttggaaatcttccagatcgtggatcaGGGCCCTTTCGTCTCGCGCGTTTCGGTGATGACGGTGAAAACCTCTGACACATGCAGCTCCCGGAGACGGTCACAGCTTG
caacctttagaaggtctagcacctagtCCCGGGAAAGCAGAGCGCGCAAAGCCACTACTGCCACTTTTGGAGACTGTGTACGTCGAGGGCCTCTGCCAGTGTCGAAC

tbb-2 UTR

2,260 2,280 2,300 2,320 2,340

TCTGTAAGCGGATGCCGGGAGCAGACAAGCCCGTCAGGGCGCGTCAGCGGGTGTTGGCGGGTGTCGGGGCTGGCTTAACTATGCGGCATCAGAGCAGATTGTACTGA
AGACATTCGCCTACGGCCCTCGTCTGTTCGGGCAGTCCCGCGCAGTCGCCCACAACCGCCCACAGCCCCGACCGAATTGATACGCCGTAGTCTCGTCTAACATGACT

2,360 2,380 2,400 2,420 2,440 2,460

GAGTGCACCATATGCGGTGTGAAATACCGCACAGATGCGTAAGGAGAAAATACCGCATCAGGCGGCCTTAAGGGCCTCGTGATACGCCTATTTTTATAGGTTAATGT
CTCACGTGGTATACGCCACACTTTATGGCGTGTCTACGCATTCCTCTTTTATGGCGTAGTCCGCCGGAATTCCCGGAGCACTATGCGGATAAAAATATCCAATTACA

2,480 2,500 2,520 2,540 2,560

CATGATAATAATGGTTTCTTAGACGTCAGGTGGCACTTTTCGGGGAAATGTGCGCGGAACCCCTATTTGTTTATTTTTCTAAATACATTCAAATATGTATCCGCTCA
GTACTATTATTACCAAAGAATCTGCAGTCCACCGTGAAAAGCCCCTTTACACGCGCCTTGGGGATAAACAAATAAAAAGATTTATGTAAGTTTATACATAGGCGAGT

2,580 2,600 2,620 2,640 2,660

pMB43 (4876 bp) (from 2676-3638 bp)

TGAGACAATAACCCTGATAAATGCTTCAATAATATTGAAAAAGGAAGAGTATGAGTATTCAACATTTCCGTGTCGCCCTTATTCCCTTTTTTGCGGCATTTTGCCTT
ACTCTGTTATTGGGACTATTTACGAAGTTATTATAACTTTTTCCTTCTCATACTCATAAGTTGTAAAGGCACAGCGGGAATAAGGGAAAAAACGCCGTAAAACGGAA

2,680 2,700 2,720 2,740 2,760 2,780

CCTGTTTTTGCTCACCCAGAAACGCTGGTGAAAGTAAAAGATGCTGAAGATCAGTTGGGTGCACGAGTGGGTTACATCGAACTGGATCTCAACAGCGGTAAGATCCT
GGACAAAAACGAGTGGGTCTTTGCGACCACTTTCATTTTCTACGACTTCTAGTCAACCCACGTGCTCACCCAATGTAGCTTGACCTAGAGTTGTCGCCATTCTAGGA

2,800 2,820 2,840 2,860 2,880

TGAGAGTTTTCGCCCCGAAGAACGTTTTCCAATGATGAGCACTTTTAAAGTTCTGCTATGTGGCGCGGTATTATCCCGTATTGACGCCGGGCAAGAGCAACTCGGTC
ACTCTCAAAAGCGGGGCTTCTTGCAAAAGGTTACTACTCGTGAAAATTTCAAGACGATACACCGCGCCATAATAGGGCATAACTGCGGCCCGTTCTCGTTGAGCCAG

AmpR

2,900 2,920 2,940 2,960 2,980

GCCGCATACACTATTCTCAGAATGACTTGGTTGAGTACTCACCAGTCACAGAAAAGCATCTTACGGATGGCATGACAGTAAGAGAATTATGCAGTGCTGCCATAACC
CGGCGTATGTGATAAGAGTCTTACTGAACCAACTCATGAGTGGTCAGTGTCTTTTCGTAGAATGCCTACCGTACTGTCATTCTCTTAATACGTCACGACGGTATTGG

AmpR

3,000 3,020 3,040 3,060 3,080 3,100

ATGAGTGATAACACTGCGGCCAACTTACTTCTGACAACGATCGGAGGACCGAAGGAGCTAACCGCTTTTTTGCACAACATGGGGGATCATGTAACTCGCCTTGATCG
TACTCACTATTGTGACGCCGGTTGAATGAAGACTGTTGCTAGCCTCCTGGCTTCCTCGATTGGCGAAAAAACGTGTTGTACCCCCTAGTACATTGAGCGGAACTAGC

AmpR

3,120 3,140 3,160 3,180 3,200

TTGGGAACCGGAGCTGAATGAAGCCATACCAAACGACGAGCGTGACACCACGATGCCTGTAGCAATGGCAACAACGTTGCGCAAACTATTAACTGGCGAACTACTTA
AACCCTTGGCCTCGACTTACTTCGGTATGGTTTGCTGCTCGCACTGTGGTGCTACGGACATCGTTACCGTTGTTGCAACGCGTTTGATAATTGACCGCTTGATGAAT

AmpR

3,220 3,240 3,260 3,280 3,300

CTCTAGCTTCCCGGCAACAATTAATAGACTGGATGGAGGCGGATAAAGTTGCAGGACCACTTCTGCGCTCGGCCCTTCCGGCTGGCTGGTTTATTGCTGATAAATCT
GAGATCGAAGGGCCGTTGTTAATTATCTGACCTACCTCCGCCTATTTCAACGTCCTGGTGAAGACGCGAGCCGGGAAGGCCGACCGACCAAATAACGACTATTTAGA

AmpR

3,320 3,340 3,360 3,380 3,400 3,420

GGAGCCGGTGAGCGTGGGTCTCGCGGTATCATTGCAGCACTGGGGCCAGATGGTAAGCCCTCCCGTATCGTAGTTATCTACACGACGGGGAGTCAGGCAACTATGGA
CCTCGGCCACTCGCACCCAGAGCGCCATAGTAACGTCGTGACCCCGGTCTACCATTCGGGAGGGCATAGCATCAATAGATGTGCTGCCCCTCAGTCCGTTGATACCT

AmpR

3,440 3,460 3,480 3,500 3,520

TGAACGAAATAGACAGATCGCTGAGATAGGTGCCTCACTGATTAAGCATTGGTAACTGTCAGACCAAGTTTACTCATATATACTTTAGATTGATTTAAAACTTCATT
ACTTGCTTTATCTGTCTAGCGACTCTATCCACGGAGTGACTAATTCGTAACCATTGACAGTCTGGTTCAAATGAGTATATATGAAATCTAACTAAATTTTGAAGTAA

AmpR

3,540 3,560 3,580 3,600 3,620

pMB43 (4876 bp) (from 3639-4601 bp)

TTTAATTTAAAAGGATCTAGGTGAAGATCCTTTTTGATAATCTCATGACCAAAATCCCTTAACGTGAGTTTTCGTTCCACTGAGCGTCAGACCCCGTAGAAAAGATC
AAATTAAATTTTCCTAGATCCACTTCTAGGAAAAACTATTAGAGTACTGGTTTTAGGGAATTGCACTCAAAAGCAAGGTGACTCGCAGTCTGGGGCATCTTTTCTAG
ColE1 origin
3,640 3,660 3,680 3,700 3,720 3,740

AAAGGATCTTCTTGAGATCCTTTTTTTCTGCGCGTAATCTGCTGCTTGCAAACAAAAAAACCACCGCTACCAGCGGTGGTTTGTTTGCCGGATCAAGAGCTACCAAC
TTTCCTAGAAGAACTCTAGGAAAAAAAGACGCGCATTAGACGACGAACGTTTGTTTTTTTGGTGGCGATGGTCGCCACCAAACAAACGGCCTAGTTCTCGATGGTTG
ColE1 origin
3,760 3,780 3,800 3,820 3,840

TCTTTTTCCGAAGGTAACTGGCTTCAGCAGAGCGCAGATACCAAATACTGTCCTTCTAGTGTAGCCGTAGTTAGGCCACCACTTCAAGAACTCTGTAGCACCGCCTA
AGAAAAAGGCTTCCATTGACCGAAGTCGTCTCGCGTCTATGGTTTATGACAGGAAGATCACATCGGCATCAATCCGGTGGTGAAGTTCTTGAGACATCGTGGCGGAT
ColE1 origin
3,860 3,880 3,900 3,920 3,940

CATACCTCGCTCTGCTAATCCTGTTACCAGTGGCTGCTGCCAGTGGCGATAAGTCGTGTCTTACCGGGTTGGACTCAAGACGATAGTTACCGGATAAGGCGCAGCGG
GTATGGAGCGAGACGATTAGGACAATGGTCACCGACGACGGTCACCGCTATTCAGCACAGAATGGCCCAACCTGAGTTCTGCTATCAATGGCCTATTCCGCGTCGCC
ColE1 origin
3,960 3,980 4,000 4,020 4,040 4,060

TCGGGCTGAACGGGGGGTTCGTGCACACAGCCCAGCTTGGAGCGAACGACCTACACCGAACTGAGATACCTACAGCGTGAGCATTGAGAAAGCGCCACGCTTCCCGA
AGCCCGACTTGCCCCCCAAGCACGTGTGTCGGGTCGAACCTCGCTTGCTGGATGTGGCTTGACTCTATGGATGTCGCACTCGTAACTCTTTCGCGGTGCGAAGGGCT
ColE1 origin
4,080 4,100 4,120 4,140 4,160

AGGGAGAAAGGCGGACAGGTATCCGGTAAGCGGCAGGGTCGGAACAGGAGAGCGCACGAGGGAGCTTCCAGGGGGAAACGCCTGGTATCTTTATAGTCCTGTCGGGT
TCCCTCTTTCCGCCTGTCCATAGGCCATTCGCCGTCCCAGCCTTGTCCTCTCGCGTGCTCCCTCGAAGGTCCCCCTTTGCGGACCATAGAAATATCAGGACAGCCCA
ColE1 origin
4,180 4,200 4,220 4,240 4,260 4,280

TTCGCCACCTCTGACTTGAGCGTCGATTTTTGTGATGCTCGTCAGGGGGGCGGAGCCTATGGAAAAACGCCAGCAACGCGGCCTTTTTACGGTTCCTGGCCTTTTGC
AAGCGGTGGAGACTGAACTCGCAGCTAAAAACACTACGAGCAGTCCCCCCGCCTCGGATACCTTTTTGCGGTCGTTGCGCCGGAAAAATGCCAAGGACCGGAAAACG
ColE1 origin
4,300 4,320 4,340 4,360 4,380

TGGCCTTTTGCTCACATGTTCTTTCCTGCGTTATCCCCTGATTCTGTGGATAACCGTATTACCGCCTTTGAGTGAGCTGATACCGCTCGCCGCAGCCGAACGACCGA
ACCGGAAAACGAGTGTACAAGAAAGGACGCAATAGGGGACTAAGACACCTATTGGCATAATGGCGGAAACTCACTCGACTATGGCGAGCGGCGTCGGCTTGCTGGCT
4,400 4,420 4,440 4,460 4,480

GCGCAGCGAGTCAGTGAGCGAGGAAGCGGAAGAGCGCCCAATACGCAAACCGCCTCTCCCCGCGCGTTGGCCGATTCATTAATGCAGCTGGCACGACAGGTTTCCCG
CGCGTCGCTCAGTCACTCGCTCCTTCGCCTTCTCGCGGGTTATGCGTTTGGCGGAGAGGGGCGCGCAACCGGCTAAGTAATTACGTCGACCGTGCTGTCCAAAGGGC
4,500 4,520 4,540 4,560 4,580 4,600

pMB43 (4876 bp) (from 4602-4876 bp)

ACTGGAAAGCGGGCAGTGAGCGCAACGCAATTAATGTGAGTTAGCTCACTCATTAGGCACCCCAGGCTTTACACTTTATGCTTCCGGCTCGTATGTTGTGTGGAATT
TGACCTTTCGCCCGTCACTCGCGTTGCGTTAATTACACTCAATCGAGTGAGTAATCCGTGGGGTCCGAAATGTGAAATACGAAGGCCGAGCATACAACACACCTTAA

4,620 4,640 4,660 4,680 4,700

GTGAGCGGATAACAATTTCACACAGGAAACAGCTATGACCATGATTACGCCAAGCTgtaagtttaaacatgatcttactaactaactattctcatttaaattttcag
CACTCGCCTATTGTTAAAGTGTGTCCTTTGTCGATACTGGTACTAATGCGGTTCGAcattcaaatttgtactagaatgattgattgataagagtaaatttaaaagtc

LacO M13-rev

4,720 4,740 4,760 4,780 4,800

AGCTTAAAAATGGCTGAAATCACTCACAACGATGGATACGCTAACAACTTGGAAATGAAAT
TCGAATTTTTACCGACTTTAGTGAGTGTTGCTACCTATGCGATTGTTGAACCTTTACTTTA

4,820 4,830 4,840 4,850 4,860 4,870

pMB71 (Pelt-2::BirA) (7993 bp)

AAGCTTGGGCTGCAGTAATTTCGAAATGTATGAACTCCAATTCTTGATAACCAATTCCTGACTCTGTAACTATTATTATTATAATCCTATTACCCACCTTTTTTCCC
TTCGAACCCGACGTCATTAAAGCTTTACATACTTGAGGTTAAGAACTATTGGTTAAGGACTGAGACATTGATAATAATAATATTAGGATAATGGGTGGAAAAAAGGG
elt-2 promoter
20 40 60 80 100

ATTCTGCTCTTAGTTCTCCCCCAACCCGTACATTTTCTCAACTAATgAAGACAATGAGAAAGTGAGAGAGAATAAGACGAACTGAAAAGAATGTGTAAACAGTTTAT
TAAGACGAGAATCAAGAGGGGGTTGGGCATGTAAAAGAGTTGATTAcTTCTGTTACTCTTTCACTCTCTCTTATTCTGCTTGACTTTTCTTACACATTTGTCAAATA
elt-2 promoter elt-2 promoter
120 140 160 180 200

TGCCTCTCGATTGTACCATCTAGTGTCTTGTCGTGTGCATAACTACTGATAACAGATCGAATGTGAAAGACCCAATAAATGCTTGGAAAGTATCAAATATGTTTAGA
ACGGAGAGCTAACATGGTAGATCACAGAACAGCACACGTATTGATGACTATTGTCTAGCTTACACTTTCTGGGTTATTTACGAACCTTTCATAGTTTATACAAATCT
elt-2 promoter
220 240 260 280 300 320

AATAGTATTTAGAGACTTATGTGGGACGAAAAAACAGGAGCATTAAGAATACAGTTGCAAACATTCATCAAGTTATAATCTAATAATATAAATcCAAAAATGTTAAT
TTATCATAAATCTCTGAATACACCCTGCTTTTTTGTCCTCGTAATTCTTATGTCAACGTTTGTAAGTAGTTCAATATTAGATTATTATATTTAgGTTTTTACAATTA
elt-2 promoter
340 360 380 400 420

GATAAAAAATGATGATCAAAGTAGGAAATTAGTGGCGAGAAAACAATAAATCATCAGAAAATGACTGATCTTGAGTAGAGTGCTTTTTTTGTAGATTTCTGATTCTA
CTATTTTTTACTACTAGTTTCATCCTTTAATCACCGCTCTTTTGTTATTTAGTAGTCTTTTACTGACTAGAACTCATCTCACGAAAAAAACATCTAAAGACTAAGAT
elt-2 promoter
440 460 480 500 520

AAACTGAAAAAAAAACTAAAAATGCTCGATGAGTCATACTAATTGAAAAATAATCAGATTGAAAACGTTAAAAATGTACTTGCTAACATACTTCCATGGTTATTTTT
TTTGACTTTTTTTTTGATTTTTACGAGCTACTCAGTATGATTAACTTTTTATTAGTCTAACTTTTGCAATTTTTACATGAACGATTGTATGAAGGTACCAATAAAAA
elt-2 promoter
540 560 580 600 620 640

GAACACAAATAAAGTTATATACATCTTTACCGGCACCAGAAGAAATCACCGCTAACCAATGTTCAGTTTTTACCTGAATATCCCTGAAAGTACAAAAAAATCAATGT
CTTGTGTTTATTTCAATATATGTAGAAATGGCCGTGGTCTTCTTTAGTGGCGATTGGTTACAAGTCAAAAATGGACTTATAGGGACTTTCATGTTTTTTTAGTTACA
elt-2 promoter
660 680 700 720 740

TCAATCAAGTTTTTGCATGCCTTCGTGTGATGGCTCGCACCAAGTATTCTGACAGTTATAAAATGTTTCCTGTGATCATGAATTTGAAAAAAAAAGAACTGATGGGA
AGTTAGTTCAAAAACGTACGGAAGCACACTACCGAGCGTGGTTCATAAGACTGTCAATATTTTACAAAGGACACTAGTACTTAAACTTTTTTTTTCTTGACTACCCT
elt-2 promoter
760 780 800 820 840

pMB71 (Pelt-2::BirA) (7993 bp) (from 857-1712 bp)

```
AAATGTGATATTCATAAATGAAATGTTTAATAAACAAAAAAGAACCGTCATCACAAATCTACTCTGAAACGTTCACAGTTCCTAATTGACCAATTCAGAATTTTTTG
TTTACACTATAAGTATTTACTTTACAAATTATTTGTTTTTTCTTGGCAGTAGTGTTTAGATGAGACTTTGCAAGTGTCAAGGATTAACTGGTTAAGTCTTAAAAAAC
elt-2 promoter
860 880 900 920 940 960

TACTGACTTAATAAATATAAATTTACAGTTTTAAGAAACATAAATTGAATTATATAAATAAAAAAAAAAAAAACTAAAATGAAGACGTGATGGAATGTTTCACGTATT
ATGACTGAATTATTTATATTTAAATGTCAAAATTCTTTGTATTTAACTTAATATATTTATTTTTTTTTTTTTTGATTTTACTTCTGCACTACCTTACAAAGTGCATAA
elt-2 promoter
980 1,000 1,020 1,040 1,060

AAAAAAGCTCTCGGACCGTTTGACATCCAGAAATTATGTGAAAGTGATTTTCTAACCCTTTGACGTCAAAAAAGTTTTTCTTTTTTGAAAGATACGCTGTTTAGAAA
TTTTTTCGAGAGCCTGGCAAACTGTAGGTCTTTAATACACTTTCACTAAAAGATTGGGAAACTGCAGTTTTTTCAAAAAGAAAAAACTTTCTATGCGACAAATCTTT
elt-2 promoter
1,080 1,100 1,120 1,140 1,160

TACTTATACAGCTGATTTTAGAATCTCCAAAAAACCTTCAATTACTTAACAAACTTTCTAAATAAAGAATTTTGATTTTGATTTTCTTTGAACTTCAAACCCCCCGT
ATGAATATGTCGACTAAAATCTTAGAGGTTTTTTGGAAGTTAATGAATTGTTTGAAAGATTTATTTCTTAAAACTAAAACTAAAAGAAACTTGAAGTTTGGGGGGCA
elt-2 promoter
1,180 1,200 1,220 1,240 1,260 1,280

TAAAAAACCAAATTAAAAAAAAGATACCAGATTAATTTTAAAGTGAAAAATATAAATTTATTCCCGACTGTTAAAAACGGTTTATAAACGGAGTCACAACACTCATA
ATTTTTTGGTTTAATTTTTTTTCTATGGTCTAATTAAAATTTCACTTTTTATATTTAAATAAGGGCTGACAATTTTTGCCAAATATTTGCCTCAGTGTTGTGAGTAT
elt-2 promoter
1,300 1,320 1,340 1,360 1,380

TACAACTATCGACACCAAATAAATAACTTCGAAAGCTAATTGTGTCATTTCGGATATAGAGAGTAGTGTGAGCAGATGTGTTTTTGCAGAACTAAATTGATGACACT
ATGTTGATAGCTGTGGTTTATTTATTGAAGCTTTCGATTAACACAGTAAAGCCTATATCTCTCATCACACTCGTCTACACAAAAACGTCTTGATTTAACTACTGTGA
elt-2 promoter
1,400 1,420 1,440 1,460 1,480

ATTATGGAATATAATGACCAAATGTTAAATGTGTTAAGGTTTGATATCAAAACCTGTATTTTCTTTTTATACAGAATTGATAATGTTATCTTCAATTGATTTCTACT
TAATACCTTATATTACTGGTTTACAATTTACACAATTCCAAACTATAGTTTTGGACATAAAAGAAAAATATGTCTTAACTATTACAATAGAAGTTAACTAAAGATGA
elt-2 promoter
1,500 1,520 1,540 1,560 1,580 1,600

TCTGAGCTACGGCGATACGAGGACGCATTCTCAACGATAATGTTGCCATTTTGTCCTGATAATTTTTTTACTGATTGTTTCAGAACACCCATAGTTTTTCTCTATTA
AGACTCGATGCCGCTATGCTCCTGCGTAAGAGTTGCTATTACAACGGTAAAACAGGACTATTAAAAAAATGACTAACAAAGTCTTGTGGGTATCAAAAAGAGATAAT
elt-2 promoter
1,620 1,640 1,660 1,680 1,700
```

pMB71 (Pelt-2::BirA) (7993 bp) (from 1713-2568 bp)

AACGTTCATCCTTGACTTCCCCGAGTTTGCTGGCTGAATAGGAAATTTGAAGACAAAAAGGAAAGAATCGGCTCAAACGTCATGCAACTGATAAGGCGACCGTACTT
TTGCAAGTAGGAACTGAAGGGGCTCAAACGACCGACTTATCCTTTAAACTTCTGTTTTTCCTTTCTTAGCCGAGTTTGCAGTACGTTGACTATTCCGCTGGCATGAA
elt-2 promoter
1,720 1,740 1,760 1,780 1,800

CATTTCAAAAGAAGCTCACTTACTGAGCGCAAACATTGAGAAATGAGAGCAAAAGAAGTGGTTTTACAACATGCTAATGTTTTTAGACCTTGTACCCAATAATATTA
GTAAAGTTTTCTTCGAGTGAATGACTCGCGTTTGTAACTCTTTACTCTCGTTTTCTTCACCAAAATGTTGTACGATTACAAAAATCTGGAACATGGGTTATTATAAT
elt-2 promoter
1,820 1,840 1,860 1,880 1,900 1,920

CTGTAGTATACAGTTCGGAGAGCATATGGTTGAAATCTTGAAATACCAATTTATCACTAGTTTGATTGTGTTATCGATGTATAAAGATATATTTTATCATTTTTGAT
GACATCATATGTCAAGCCTCTCGTATACCAACTTTAGAACTTTATGGTTAAATAGTGATCAAACTAACACAATAGCTACATATTTCTATATAAAATAGTAAAAACTA
elt-2 promoter
1,940 1,960 1,980 2,000 2,020

TATTATCTGATATTGTGGGGTGTGAAGTAATATTATGTGCGTGTGTGGCTGATTATCGAAAAAAACTGAAAATTATCAATTTTTCTACAGGTTATCTTTTTTTGTTT
ATAATAGACTATAACACCCCACACTTCATTATAATACACGCACACACCGACTAATAGCTTTTTTTGACTTTTAATAGTTAAAAAGATGTCCAATAGAAAAAAACAAA
elt-2 promoter
2,040 2,060 2,080 2,100 2,120 2,140

TATTTTTCATTATTGTATTCTTCATACTCCTTATCCTGCCCGAATCACAAAAGTTCCAGACAACTTGAAGGTCGCATCACGTTTTGTTTATAAGAATGTTGAACTGG
ATAAAAAGTAATAACATAAGAAGTATGAGGAATAGGACGGGCTTAGTGTTTTCAAGGTCTGTTGAACTTCCAGCGTAGTGCAAAACAAATATTCTTACAACTTGACC
elt-2 promoter
2,160 2,180 2,200 2,220 2,240

TCAGCTTTGGAGACAGAACTGTCCTCCAAATGTTGAATATGTTTCCGTAAAACACACTGTGTAACAAAAAGCGTACCCTTTGATCCGGTATTCTTGAAAAAAAACAT
AGTCGAAACCTCTGTCTTGACAGGAGGTTTACAACTTATACAAAGGCATTTTGTGTGACACATTGTTTTTCGCATGGGAAACTAGGCCATAAGAACTTTTTTTTGTA
elt-2 promoter
2,260 2,280 2,300 2,320 2,340

AGAAGGGCGTACTTTTCATATCTCACAAATATGCGTTTTTTATTTTATAATAATATCATTTATTGATAATTATATGTATTTGTATGTTTTCTTGTCTTTAAAATTAT
TCTTCCCGCATGAAAAGTATAGAGTGTTTATACGCAAAAAATAAAATATTATTATAGTAAATAACTATTAATATACATAAACATACAAAAGAACAGAAATTTTAATA
elt-2 promoter
2,360 2,380 2,400 2,420 2,440 2,460

GGTGTGACATCACAGTTTAAAAATTATATGAAAACTAATGAGTGAAAAGTTAAAAATGTATAGTTTGCCGTATTTTCTCCATTAGTATTGTATTCAAAACTTATTTC
CCACACTGTAGTGTCAAATTTTTAATATACTTTTGATTACTCACTTTTCAATTTTTACATATCAAACGGCATAAAAGAGGTAATCATAACATAAGTTTTGAATAAAG
elt-2 promoter
2,480 2,500 2,520 2,540 2,560

pMB71 (Pelt-2::BirA) (7993 bp) (from 2569-3424 bp)

CAATTGGTGAGCACTATAAACTTTGTAGGGTAATTGAGGTTCAATACTAATTTTTGAATATCTAGCTTTTGACAAATTATTCAAGTTTTGACAATGATGCCTTATCA
GTTAACCACTCGTGATATTTGAAACATCCCATTAACTCCAAGTTATGATTAAAAACTTATAGATCGAAAACTGTTTAATAAGTTCAAAACTGTTACTACGGAATAGT

elt-2 promoter

2,580 2,600 2,620 2,640 2,660

AATTAAAAAAAATTACCATAAATTCTTGAAAGCTTTTGATTTTGTTTCACTCTGTGAAGGATAGTAATTTATCAACATTTTAACTAGATACATCTGTTCTGGCAATA
TTAATTTTTTTTAATGGTATTTAAGAACTTTCGAAAACTAAAACAAAGTGAGACACTTCCTATCATTAAATAGTTGTAAAATTGATCTATGTAGACAAGACCGTTAT

elt-2 promoter

2,680 2,700 2,720 2,740 2,760 2,780

AATTAAAGCAAGACTATTACTATGTGCAGTATTAATAGCTGCATTGCAACTTGTTTGAAAGTTTCAGTGAAAACGCAAAAACTCAATTTAATTTTCAAGTTTGTTTG
TTAATTTCGTTCTGATAATGATACACGTCATAATTATCGACGTAACGTTGAACAAACTTTCAAAGTCACTTTTGCGTTTTTGAGTTAAATTAAAAGTTCAAACAAAC

elt-2 promoter

2,800 2,820 2,840 2,860 2,880

CAAAATTGGATATATGTATCCATCAAAAGTCTGATCGCCTGTTTTATCTACTTATTGTCTCCTCAGTTGATTTTTTTGTAGTTATTATAAACGCTACTTTGAAACCA
GTTTTAACCTATATACATAGGTAGTTTTCAGACTAGCGGACAAAATAGATGAATAACAGAGGAGTCAACTAAAAAAACATCAATAATATTTGCGATGAAACTTTGGT

elt-2 promoter

2,900 2,920 2,940 2,960 2,980

ATATTTTCTTCCTCATTCCACTTTTTTCAATTACCAACTACCGTACATGCAATGATGGGCGGACCCGAGTCCCATGTTTGGCTGGAAGTGGGTGGTTGTCTGCGTAT
TATAAAAGAAGGAGTAAGGTGAAAAAAGTTAATGGTTGATGGCATGTACGTTACTACCCGCCTGGGCTCAGGGTACAAACCGACCTTCACCCACCAACAGACGCATA

elt-2 promoter

3,000 3,020 3,040 3,060 3,080 3,100

ATGAGCGACAGAGGTCGGGGCTGAAACTGATAAGAATAGTCGACACTAACGCCATAATCGCTAGCCAGCCATCATGCACACCGAGCTCGGTGTGCACACCATCTTTC
TACTCGCTGTCTCCAGCCCCGACTTTGACTATTCTTATCAGCTGTGATTGCGGTATTAGCGATCGGTCGGTAGTACGTGTGGCTCGAGCCACACGTGTGGTAGAAAG

elt-2 promoter

3,120 3,140 3,160 3,180 3,200

TTTTCAAACCAATACGCTTTGTGCCTTCATTGACAATTTTCTTTTTGATAAAATCAGCCTATCTATACTTCCCAATCATTTTTAGTCTTATCGTTGAACAGCTATCG
AAAAGTTTGGTTATGCGAAACACGGAAGTAACTGTTAAAAGAAAAACTATTTTAGTCGGATAGATATGAAGGGTTAGTAAAAATCAGAATAGCAACTTGTCGATAGC

elt-2 promoter

3,220 3,240 3,260 3,280 3,300

AGGTGCCACTGTTTTCACTGATATCTTCTAAGTTACTATGGCATTAACATCTTTGTGTTCTTATTTTATGGGTTATTTTAATTAATTTTTGCAGTTAATTTTTGGAA
TCCACGGTGACAAAAGTGACTATAGAAGATTCAATGATACCGTAATTGTAGAAACACAAGAATAAAATACCCAATAAAATTAATTAAAAACGTCAATTAAAAACCTT

elt-2 promoter

3,320 3,340 3,360 3,380 3,400 3,420

pMB71 (Pelt-2::BirA) (7993 bp) (from 3425-4280 bp)

TGAGCAAGAAAATGTTAATTGTAATATCTTCGTCTGAAAATTGTCTTCAAATAGTTTTAATTTTAAAGGCAGTATTTAAGAAAATACACTTCTCGAAGCATTTTTGA
ACTCGTTCTTTTACAATTAACATTATAGAAGCAGACTTTTAACAGAAGTTTATCAAAATTAAAATTTCCGTCATAAATTCTTTTATGTGAAGAGCTTCGTAAAAACT

elt-2 promoter

3,440 3,460 3,480 3,500 3,520

ATTTTTGAATTTTTAAACTGCTTGATGTTTTAGGTGCCACTGTTTTCATTGATAAGTTTTGATGTATAAATGCTTGATTTTCTTGGCATTCTAATAAAATAGAAACT
TAAAAACTTAAAAATTTGACGAACTACAAAATCCACGGTGACAAAAGTAACTATTCAAAACTACATATTTACGAACTAAAAGAACCGTAAGATTATTTTATCTTTGA

elt-2 promoter

3,540 3,560 3,580 3,600 3,620

AGAAAATAGATTATAGCCCGGGTGCCCTATAGTGAGTCGTATTGGTACCaaaaATGGAGCAGAAGCTCATCTCTGAGGAAGACCTCGGAGGAGAGCAGAAACTCATC
TCTTTTATCTAATATCGGGCCCACGGGATATCACTCAGCATAACCATGGttttTACCTCGTCTTCGAGTAGAGACTCCTTCTGGAGCCTCCTCTCGTCTTTGAGTAG

MYC-tag 1 MYC-tag 2

3,640 3,660 3,680 3,700 3,720 3,740

TCTGAAGAGGACCTCATGAAGGATAATACTGTTCCATTGAAGTTGATTGCTTTGCTTGCAAATGGAGAATTTCATTCTGGAGAACAACTTGGAGAGACGTTGGGAAT
AGACTTCTCCTGGAGTACTTCCTATTATGACAAGGTAACTTCAACTAACGAAACGAACGTTTACCTCTTAAAGTAAGACCTCTTGTTGAACCTCTCTGCAACCCTTA

MYC-tag 2 BirA

3,760 3,780 3,800 3,820 3,840

GTCGCGTGCTGCAATTAACAAGCATATTCAAACAgtaagtttaaacagttcggtactaactaaccatacatatttaaattttcagTTGAGAGACTGGGGAGTTGACG
CAGCGCACGACGTTAATTGTTCGTATAAGTTTGTcattcaaatttgtcaagccatgattgattggtatgtataaatttaaaagtcAACTCTCTGACCCCTCAACTGC

BirA BirA

3,860 3,880 3,900 3,920 3,940

TTTTTACGGTTCCAGGAAAGGGATATTCGCTTCCAGAGCCAATTCAGCTCTTGAATGCAAAGCAAATTTTGGGACAGCTTGATGGTGGTAGTGTTGCAGTACTTCCG
AAAAATGCCAAGGTCCTTTCCCTATAAGCGAAGGTCTCGGTTAAGTCGAGAACTTACGTTTCGTTTAAAACCCTGTCGAACTACCACCATCACAACGTCATGAAGGC

BirA

3,960 3,980 4,000 4,020 4,040 4,060

GTTATAGATAGTACAAATCAGTACTTGCTTGATCGAATAGGAGAATTGAAATCAGGAGATGCTTGCATCGCTGAGTATCAGCAGGCTGGACGAGGACGAAGAGGACG
CAATATCTATCATGTTTAGTCATGAACGAACTAGCTTATCCTCTTAACTTTAGTCCTCTACGAACGTAGCGACTCATAGTCGTCCGACCTGCTCCTGCTTCTCCTGC

BirA

4,080 4,100 4,120 4,140 4,160

TAAGTGGTTCTgtaagtttaaacatatatatactaactaaccctgattatttaaattttcagCGCCTTTCGGAGCCAATCTCTATTTGAGTATGTTCTGGCGTCTTG
ATTCACCAAGAcattcaaatttgtatatatatgattgattgggactaataaatttaaaagtcGCGGAAAGCCTCGGTTAGAGATAAACTCATACAAGACCGCAGAAC

BirA BirA

4,180 4,200 4,220 4,240 4,260 4,280

pMB71 (Pelt-2::BirA) (7993 bp) (from 4281-5243 bp)

AACAAGGTCCGGCCGCTGCTATCGGACTTTCATTGGTTATTGGAATTGTGATGGCAGAAGTTCTCCGAAAACTTGGTGCAGATAAGGTGAGAGTCAAGTGGCCTAAC
TTGTTCCAGGCCGGCGACGATAGCCTGAAAGTAACCAATAACCTTAACACTACCGTCTTCAAGAGGCTTTTGAACCACGTCTATTCCACTCTCAGTTCACCGGATTG
BirA
4,300 4,320 4,340 4,360 4,380

GATCTTTATCTTCAAGATAGAAAATTGGCCGGAATATTGgtaagtttaaacatatatatactaactaaccctgattatttaaattttcagGTTGAGCTTACTGGAAA
CTAGAAATAGAAGTTCTATCTTTTAACCGGCCTTATAACcattcaaatttgtatatatatgattgattgggactaataaatttaaaagtcCAACTCGAATGACCTTT
BirA BirA
4,400 4,420 4,440 4,460 4,480

GACGGGAGATGCTGCTCAAATTGTGATTGGAGCTGGTATCAATATGGCAATGAGAAGAGTAGAAGAGTCTGTTGTTAATCAAGGTTGGATCACACTTCAAGAGGCAG
CTGCCCTCTACGACGAGTTTAACACTAACCTCGACCATAGTTATACCGTTACTCTTCTCATCTTCTCAGACAACAATTAGTTCCAACCTAGTGTGAAGTTCTCCGTC
BirA
4,500 4,520 4,540 4,560 4,580 4,600

GAATCAACCTTGATAGAAATACGTTGGCCGCCATGCTTATCCGAGAATTGCGAGCTGCACTCGAACTCTTCGAACAAGAAGGTCTTgtaagtttaaacatatatata
CTTAGTTGGAACTATCTTTATGCAACCGGCGGTACGAATAGGCTCTTAACGCTCGACGTGAGCTTGAGAAGCTTGTTCTTCCAGAAcattcaaatttgtatatatat
BirA
4,620 4,640 4,660 4,680 4,700

ctaactaaccctgattatttaaattttcagGCCCCTTACCTCAGTAGATGGGAGAAACTTGACAATTTTATCAATCGACCAGTGAAGTTGATAATTGGAGATAAGGA
gattgattgggactaataaatttaaaagtcCGGGGAATGGAGTCATCTACCCTCTTTGAACTGTTAAAATAGTTAGCTGGTCACTTCAACTATTAACCTCTATTCCT
BirA
4,720 4,740 4,760 4,780 4,800

AATTTTTGGTATCAGTCGAGGAATTGATAAGCAAGGAGCTCTTCTCCTTGAGCAAGATGGAATCATTAAACCATGGATGGGTGGAGAGATTTCTTTGCGATCTGCTG
TTAAAAACCATAGTCAGCTCCTTAACTATTCGTTCCTCGAGAAGAGGAACTCGTTCTACCTTAGTAATTTGGTACCTACCCACCTCTCTAAAGAAACGCTAGACGAC
BirA
4,820 4,840 4,860 4,880 4,900 4,920

AAAAGTAAGAATTCGCTAGCCGGCCATACAAGTAATCCGGATGATCGACGCCaACGTCGTTGAATTTTCAAATTTTAAATACTGAATATTTGTTTTTTTTCCTATTA
TTTTCATTCTTAAGCGATCGGCCGGTATGTTCATTAGGCCTACTAGCTGCGGtTGCAGCAACTTAAAAGTTTAAAATTTATGACTTATAAACAAAAAAAAGGATAAT
let-858 UTR
4,940 4,960 4,980 5,000 5,020

TTTATTTATTCTCTTTGTGTTTTTTTTCTTGCTTTCTAAAAAATTAATTCAATCCAAATCTAAacatttttttttctctttccgtctcccaattcgtattccgctcc
AAATAAATAAGAGAAACACAAAAAAAAGAACGAAAGATTTTTTAATTAAGTTAGGTTTAGATTtgtaaaaaaaaagagaaaggcagagggttaagcataaggcgagg
let-858 UTR
5,040 5,060 5,080 5,100 5,120

tctcatctgaacacaatgtgcaagtttatttatcttctcgctttcatttcattaggacgtgggggaattggtggaagggggaaacacacaaaaggatgatggaaat
agagtagacttgtgttacacgttcaaataaatagaagagcgaaagtaaagtaatcctgcacccccttaaccaccttccccctttgtgtgttttcctactacctttta
5,140 5,160 5,180 5,200 5,220 5,240

gaaataaggacacacaatatgcaacaacattcaattcagaaatatggaggaaggtttaaaagaaaacataaaaatatatagaggaggaaggaaaactagtaaaaaat
ctttattcctgtgtgttatacgttgttgtaagttaagtctttatacctccttccaaattttcttttgtatttttatatatctcctccttccttttgatcattttttta

5,260 5,280 5,300 5,320 5,340

aagcaaagaaattaggcgaacgatgAGAATTGTCCTCGCTTGGGCCCTTTCGTCTCGCGCGTTTCGGTGATGACGGTGAAAACCTCTGACACATGCAGCTCCCGGAG
ttcgtttctttaatccgcttgctacTCTTAACAGGAGCGAACCCGGGAAAGCAGAGCGCGCAAAGCCACTACTGCCACTTTTGGAGACTGTGTACGTCGAGGGCCTC

5,360 5,380 5,400 5,420 5,440

ACGGTCACAGCTTGTCTGTAAGCGGATGCCGGGAGCAGACAAGCCCGTCAGGGCGCGTCAGCGGGTGTTGGCGGGTGTCGGGGCTGGCTTAACTATGCGGCATCAGA
TGCCAGTGTCGAACAGACATTCGCCTACGGCCCTCGTCTGTTCGGGCAGTCCCGCGCAGTCGCCCACAACCGCCCACAGCCCCGACCGAATTGATACGCCGTAGTCT

5,460 5,480 5,500 5,520 5,540 5,560

GCAGATTGTACTGAGAGTGCACCATATGCGGTGTGAAATACCGCACAGATGCGTAAGGAGAAAATACCGCATCAGGCGGCCTTAAGGGCCTCGTGATACGCCTATTT
CGTCTAACATGACTCTCACGTGGTATACGCCACACTTTATGGCGTGTCTACGCATTCCTCTTTTATGGCGTAGTCCGCCGGAATTCCCGGAGCACTATGCGGATAAA

5,580 5,600 5,620 5,640 5,660

TTATAGGTTAATGTCATGATAATAATGGTTTCTTAGACGTCAGGTGGCACTTTTCGGGGAAATGTGCGCGGAACCCCTATTTGTTTATTTTTCTAAATACATTCAAA
AATATCCAATTACAGTACTATTATTACCAAAGAATCTGCAGTCCACCGTGAAAAGCCCCTTTACACGCGCCTTGGGGATAAACAAATAAAAAGATTTATGTAAGTTT

5,680 5,700 5,720 5,740 5,760

TATGTATCCGCTCATGAGACAATAACCCTGATAAATGCTTCAATAATATTGAAAAAGGAAGAGTATGAGTATTCAACATTTCCGTGTCGCCCTTATTCCCTTTTTTG
ATACATAGGCGAGTACTCTGTTATTGGGACTATTTACGAAGTTATTATAACTTTTTCCTTCTCATACTCATAAGTTGTAAAGGCACAGCGGGAATAAGGGAAAAAAC

5,780 5,800 5,820 5,840 5,860 5,880

CGGCATTTTGCCTTCCTGTTTTTGCTCACCCAGAAACGCTGGTGAAAGTAAAAGATGCTGAAGATCAGTTGGGTGCACGAGTGGGTTACATCGAACTGGATCTCAAC
GCCGTAAAACGGAAGGACAAAAACGAGTGGGTCTTTGCGACCACTTTCATTTTCTACGACTTCTAGTCAACCCACGTGCTCACCCAATGTAGCTTGACCTAGAGTTG

5,900 5,920 5,940 5,960 5,980

AGCGGTAAGATCCTTGAGAGTTTTCGCCCCGAAGAACGTTTTCCAATGATGAGCACTTTTAAAGTTCTGCTATGTGGCGCGGTATTATCCCGTATTGACGCCGGGCA
TCGCCATTCTAGGAACTCTCAAAAGCGGGGCTTCTTGCAAAAGGTTACTACTCGTGAAAATTTCAAGACGATACACCGCGCCATAATAGGGCATAACTGCGGCCCGT

AmpR

6,000 6,020 6,040 6,060 6,080

AGAGCAACTCGGTCGCCGCATACACTATTCTCAGAATGACTTGGTTGAGTACTCACCAGTCACAGAAAAGCATCTTACGGATGGCATGACAGTAAGAGAATTATGCA
TCTCGTTGAGCCAGCGGCGTATGTGATAAGAGTCTTACTGAACCAACTCATGAGTGGTCAGTGTCTTTTCGTAGAATGCCTACCGTACTGTCATTCTCTTAATACGT

AmpR

6,100 6,120 6,140 6,160 6,180 6,200

GTGCTGCCATAACCATGAGTGATAACACTGCGGCCAACTTACTTCTGACAACGATCGGAGGACCGAAGGAGCTAACCGCTTTTTTGCACAACATGGGGGATCATGTA
CACGACGGTATTGGTACTCACTATTGTGACGCCGGTTGAATGAAGACTGTTGCTAGCCTCCTGGCTTCCTCGATTGGCGAAAAAACGTGTTGTACCCCCTAGTACAT

AmpR

6,220 6,240 6,260 6,280 6,300

pMB71 (Pelt-2::BirA) (7993 bp) (from 6314-7169 bp)

ACTCGCCTTGATCGTTGGGAACCGGAGCTGAATGAAGCCATACCAAACGACGAGCGTGACACCACGATGCCTGTAGCAATGGCAACAACGTTGCGCAAACTATTAAC
TGAGCGGAACTAGCAACCCTTGGCCTCGACTTACTTCGGTATGGTTTGCTGCTCGCACTGTGGTGCTACGGACATCGTTACCGTTGTTGCAACGCGTTTGATAATTG

AmpR

6,320 6,340 6,360 6,380 6,400 6,420

TGGCGAACTACTTACTCTAGCTTCCCGGCAACAATTAATAGACTGGATGGAGGCGGATAAAGTTGCAGGACCACTTCTGCGCTCGGCCCTTCCGGCTGGCTGGTTTA
ACCGCTTGATGAATGAGATCGAAGGGCCGTTGTTAATTATCTGACCTACCTCCGCCTATTTCAACGTCCTGGTGAAGACGCGAGCCGGGAAGGCCGACCGACCAAAT

AmpR

6,440 6,460 6,480 6,500 6,520

TTGCTGATAAATCTGGAGCCGGTGAGCGTGGGTCTCGCGGTATCATTGCAGCACTGGGGCCAGATGGTAAGCCCTCCCGTATCGTAGTTATCTACACGACGGGGAGT
AACGACTATTTAGACCTCGGCCACTCGCACCCAGAGCGCCATAGTAACGTCGTGACCCCGGTCTACCATTCGGGAGGGCATAGCATCAATAGATGTGCTGCCCCTCA

AmpR

6,540 6,560 6,580 6,600 6,620

CAGGCAACTATGGATGAACGAAATAGACAGATCGCTGAGATAGGTGCCTCACTGATTAAGCATTGGTAACTGTCAGACCAAGTTTACTCATATATACTTTAGATTGA
GTCCGTTGATACCTACTTGCTTTATCTGTCTAGCGACTCTATCCACGGAGTGACTAATTCGTAACCATTGACAGTCTGGTTCAAATGAGTATATATGAAATCTAACT

AmpR

6,640 6,660 6,680 6,700 6,720 6,740

TTTAAAACTTCATTTTTAATTTAAAAGGATCTAGGTGAAGATCCTTTTTGATAATCTCATGACCAAAATCCCTTAACGTGAGTTTTCGTTCCACTGAGCGTCAGACC
AAATTTTGAAGTAAAAATTAAATTTTCCTAGATCCACTTCTAGGAAAAACTATTAGAGTACTGGTTTTAGGGAATTGCACTCAAAAGCAAGGTGACTCGCAGTCTGG

ColE1 origin

6,760 6,780 6,800 6,820 6,840

CCGTAGAAAAGATCAAAGGATCTTCTTGAGATCCTTTTTTTCTGCGCGTAATCTGCTGCTTGCAAACAAAAAAACCACCGCTACCAGCGGTGGTTTGTTTGCCGGAT
GGCATCTTTTCTAGTTTCCTAGAAGAACTCTAGGAAAAAAAGACGCGCATTAGACGACGAACGTTTGTTTTTTTGGTGGCGATGGTCGCCACCAAACAAACGGCCTA

ColE1 origin

6,860 6,880 6,900 6,920 6,940

CAAGAGCTACCAACTCTTTTTCCGAAGGTAACTGGCTTCAGCAGAGCGCAGATACCAAATACTGTCCTTCTAGTGTAGCCGTAGTTAGGCCACCACTTCAAGAACTC
GTTCTCGATGGTTGAGAAAAAGGCTTCCATTGACCGAAGTCGTCTCGCGTCTATGGTTTATGACAGGAAGATCACATCGGCATCAATCCGGTGGTGAAGTTCTTGAG

ColE1 origin

6,960 6,980 7,000 7,020 7,040 7,060

TGTAGCACCGCCTACATACCTCGCTCTGCTAATCCTGTTACCAGTGGCTGCTGCCAGTGGCGATAAGTCGTGTCTTACCGGGTTGGACTCAAGACGATAGTTACCGG
ACATCGTGGCGGATGTATGGAGCGAGACGATTAGGACAATGGTCACCGACGACGGTCACCGCTATTCAGCACAGAATGGCCCAACCTGAGTTCTGCTATCAATGGCC

ColE1 origin

7,080 7,100 7,120 7,140 7,160

pMB71 (Pelt-2::BirA) (7993 bp) (from 7170-7993 bp)

ATAAGGCGCAGCGGTCGGGCTGAACGGGGGGTTCGTGCACACAGCCCAGCTTGGAGCGAACGACCTACACCGAACTGAGATACCTACAGCGTGAGCATTGAGAAAGC
TATTCCGCGTCGCCAGCCCGACTTGCCCCCCAAGCACGTGTGTCGGGTCGAACCTCGCTTGCTGGATGTGGCTTGACTCTATGGATGTCGCACTCGTAACTCTTTCG

ColE1 origin

7,180 7,200 7,220 7,240 7,260

GCCACGCTTCCCGAAGGGAGAAAGGCGGACAGGTATCCGGTAAGCGGCAGGGTCGGAACAGGAGAGCGCACGAGGGAGCTTCCAGGGGGAAACGCCTGGTATCTTTA
CGGTGCGAAGGGCTTCCCTCTTTCCGCCTGTCCATAGGCCATTCGCCGTCCCAGCCTTGTCCTCTCGCGTGCTCCCTCGAAGGTCCCCCTTTGCGGACCATAGAAAT

ColE1 origin

7,280 7,300 7,320 7,340 7,360 7,380

TAGTCCTGTCGGGTTTCGCCACCTCTGACTTGAGCGTCGATTTTTGTGATGCTCGTCAGGGGGGCGGAGCCTATGGAAAAACGCCAGCAACGCGGCCTTTTTACGGT
ATCAGGACAGCCCAAAGCGGTGGAGACTGAACTCGCAGCTAAAAACACTACGAGCAGTCCCCCCGCCTCGGATACCTTTTTGCGGTCGTTGCGCCGGAAAAATGCCA

ColE1 origin

7,400 7,420 7,440 7,460 7,480

TCCTGGCCTTTTGCTGGCCTTTTGCTCACATGTTCTTTCCTGCGTTATCCCCTGATTCTGTGGATAACCGTATTACCGCCTTTGAGTGAGCTGATACCGCTCGCCGC
AGGACCGGAAAACGACCGGAAAACGAGTGTACAAGAAAGGACGCAATAGGGGACTAAGACACCTATTGGCATAATGGCGGAAACTCACTCGACTATGGCGAGCGGCG

7,500 7,520 7,540 7,560 7,580

AGCCGAACGACCGAGCGCAGCGAGTCAGTGAGCGAGGAAGCGGAAGAGCGCCCAATACGCAAACCGCCTCTCCCCGCGCGTTGGCCGATTCATTAATGCAGCTGGCA
TCGGCTTGCTGGCTCGCGTCGCTCAGTCACTCGCTCCTTCGCCTTCTCGCGGGTTATGCGTTTGGCGGAGAGGGGCGCGCAACCGGCTAAGTAATTACGTCGACCGT

7,600 7,620 7,640 7,660 7,680 7,700

CGACAGGTTTCCCGACTGGAAAGCGGGCAGTGAGCGCAACGCAATTAATGTGAGTTAGCTCACTCATTAGGCACCCCAGGCTTTACACTTTATGCTTCCGGCTCGTA
GCTGTCCAAAGGGCTGACCTTTCGCCCGTCACTCGCGTTGCGTTAATTACACTCAATCGAGTGAGTAATCCGTGGGGTCCGAAATGTGAAATACGAAGGCCGAGCAT

7,720 7,740 7,760 7,780 7,800

TGTTGTGTGGAATTGTGAGCGGATAACAATTTCACACAGGAAACAGCTATGACCATGATTACGCCAAGCTgtaagtttaaacatgatcttactaactaactattctc
ACAACACACCTTAACACTCGCCTATTGTTAAAGTGTGTCCTTTGTCGATACTGGTACTAATGCGGTTCGAcattcaaatttgtactagaatgattgattgataagag

LacO M13-rev

7,820 7,840 7,860 7,880 7,900

atttaaattttcagAGCTTAAAAATGGCTGAAATCACTCACAACGATGGATACGCTAACAACTTGGAAATGAAAT
taaatttaaaagtcTCGAATTTTTACCGACTTTAGTGAGTGTTGCTACCTATGCGATTGTTGAACCTTTACTTTA

7,920 7,930 7,940 7,950 7,960 7,970 7,980 7,990

pMB72 (C-term GFP-2xTEV-Avi) (5052 bp)

TCGCGCGTTTCGGTGATGACGGTGAAAACCTCTGACACATGCAGCTCCCGGAGACGGTCACAGCTTGTCTGTAAGCGGATGCCGGGAGCAGACAAGCCCGTCAGGGC
AGCGCGCAAAGCCACTACTGCCACTTTTGGAGACTGTGTACGTCGAGGGCCTCTGCCAGTGTCGAACAGACATTCGCCTACGGCCCTCGTCTGTTCGGGCAGTCCCG

20 40 60 80 100

GCGTCAGCGGGTGTTGGCGGGTGTCGGGGCTGGCTTAACTATGCGGCATCAGAGCAGATTGTACTGAGAGTGCACCATATGCGGTGTGAAATACCGCACAGATGCGT
CGCAGTCGCCCACAACCGCCCACAGCCCCGACCGAATTGATACGCCGTAGTCTCGTCTAACATGACTCTCACGTGGTATACGCCACACTTTATGGCGTGTCTACGCA

120 140 160 180 200

AAGGAGAAAATACCGCATCAGGCGCCATTCGCCATTCAGGCTGCGCAACTGTTGGGAAGGGCGATCGGTGCGGGCCTCTTCGCTATTACGCCAGCTGGCGAAAGGGG
TTCCTCTTTTATGGCGTAGTCCGCGGTAAGCGGTAAGTCCGACGCGTTGACAACCCTTCCCGCTAGCCACGCCCGGAGAAGCGATAATGCGGTCGACCGCTTTCCCC

LacZ alpha

220 240 260 280 300 320

GATGTGCTGCAAGGCGATTAAGTTGGGTAACGCCAGGGTTTTCCCAGTCACGACGTTGTAAAACGACGGCCAGTGAATTCGAGCTCGGTACCCGGGGATCCTCTAGA
CTACACGACGTTCCGCTAATTCAACCCATTGCGGTCCCAAAAGGGTCAGTGCTGCAACATTTTGCTGCCGGTCACTTAAGCTCGAGCCATGGGCCCCTAGGAGATCT

M13-fwd

340 360 380 400 420

TCCGGAggaggaggatctggaggaggaggatctggaggaggaggaATGAGTAAAGGAGAAGAACTTTTCACTGGAGTTGTCCCAATTCTTGTTGAATTAGATGGTGA
AGGCCTcctcctcctagacctcctcctcctagacctcctcctcctTACTCATTTCCTCTTCTTGAAAAGTGACCTCAACAGGGTTAAGAACAACTTAATCTACCACT

GFP

440 460 480 500 520

TGTTAATGGGCACAAATTTTCTGTCAGTGGAGAGGGTGAAGGTGATGCAACATACGGAAAACTTACCCTTAAATTTATTTGCACTACTGGAAAACTACCTGTTCCAT
ACAATTACCCGTGTTTAAAAGACAGTCACCTCTCCCACTTCCACTACGTTGTATGCCTTTTGAATGGGAATTTAAATAAACGTGATGACCTTTTGATGGACAAGGTA

GFP

540 560 580 600 620 640

GGgtaagtttaaacatatatatactaactaaccctgattatttaaattttcagccaacacttgtcactactttctgttatggtgttcaatgcttctcgagataccca
CCcattcaaatttgtatatatatgattgattgggactaataaatttaaaagtcggttgtgaacagtgatgaaagacaataccacaagttacgaagagctctatgggt

GFP

660 680 700 720 740

gatcatatgaaacggcatgactttttcaagagtgccatgcccgaaggttatgtacaggaaagaactatatttttcaaagatgacgggaactacaagacacgtaagtt
ctagtatactttgccgtactgaaaaagttctcacggtacgggcttccaatacatgtcctttcttgatataaaaagtttctactgcccttgatgttctgtgcattcaa

GFP

760 780 800 820 840

taaacagttcggtacgaagttcctatactttctagagaataggaacttccctgttgacaattaatcatcggcatagtatatcggcatagtataatacgacaaggtga
atttgtcaagccatgcttcaaggatatgaaagatctcttatccttgaagggacaactgttaattagtagccgtatcatatagccgtatcatattatgctgttccact

FRT site galK

860 880 900 920 940 960

pMB72 (C-term GFP-2xTEV-Avi) (5052 bp) (from 964-1819 bp)

ggaactaaacccaggaggcagatcatgagtctgaaagaaaaaacacaatctctgtttgccaacgcatttggctaccctgccactcacaccattcaggcgcctggccg
ccttgatttgggtcctccgtctagtactcagactttcttttttgtgttagagacaaacggttgcgtaaaccgatgggacggtgagtgtggtaagtccgcggaccggc
galK
980 1,000 1,020 1,040 1,060

cgtgaatttgattggtgaacacaccgactacaacgacggtttcgttctgccctgcgcgattgattatcaaaccgtgatcagttgtgcaccacgcgatgaccgtaaag
gcacttaaactaaccacttgtgtggctgatgttgctgccaaagcaagacgggacgcgctaactaatagtttggcactagtcaacacgtggtgcgctactggcatttc
galK
1,080 1,100 1,120 1,140 1,160

ttcgcgtgatggcagccgattatgaaaatcagctcgacgagttttccctcgatgcgcccattgtcgcacatgaaaactatcaatgggctaactacgttcgtggcgtg
aagcgcactaccgtcggctaatacttttagtcgagctgctcaaaagggagctacgcgggtaacagcgtgtacttttgatagttacccgattgatgcaagcaccgcac
galK
1,180 1,200 1,220 1,240 1,260 1,280

gtgaaacatctgcaactgcgtaacaacagcttcggcggcgtggacatggtgatcagcggcaatgtgccgcagggtgccgggttaagttcttccgcttcactggaagt
cactttgtagacgttgacgcattgttgtcgaagccgccgcacctgtaccactagtcgccgttacacggcgtcccacggcccaattcaagaaggcgaagtgaccttca
galK
1,300 1,320 1,340 1,360 1,380

cgcggtcggaaccgtattgcagcagctttatcatctgccgctggacggcgcacaaatcgcgcttaacggtcaggaagcagaaaaccagtttgtaggctgtaactgcg
gcgccagccttggcataacgtcgtcgaaatagtagacggcgacctgccgcgtgtttagcgcgaattgccagtccttcgtcttttggtcaaacatccgacattgacgc
galK
1,400 1,420 1,440 1,460 1,480

ggatcatggatcagctaatttccgcgctcggcaagaaagatcatgccttgctgatcgattgccgctcactggggaccaaagcagtttccatgcccaaaggtgtggct
cctagtacctagtcgattaaaggcgcgagccgttctttctagtacggaacgactagctaacggcgagtgacccctggtttcgtcaaaggtacgggtttccacaccga
galK
1,500 1,520 1,540 1,560 1,580 1,600

gtcgtcatcatcaacagtaacttcaaacgtaccctggttggcagcgaatacaacacccgtcgtgaacagtgcgaaaccggtgcgcgtttcttccagcagccagccct
cagcagtagtagttgtcattgaagtttgcatgggaccaaccgtcgcttatgttgtgggcagcacttgtcacgctttggccacgcgcaaagaaggtcgtcggtcggga
galK
1,620 1,640 1,660 1,680 1,700

gcgtgatgtcaccattgaagagttcaacgctgttgcgcatgaactggacccgatcgtggcaaaacgcgtgcgtcatatactgactgaaaacgcccgcaccgttgaag
cgcactacagtggtaacttctcaagttgcgacaacgcgtacttgacctgggctagcaccgttttgcgcacgcagtatatgactgacttttgcgggcgtggcaacttc
galK
1,720 1,740 1,760 1,780 1,800

pMB72 (C-term GFP-2xTEV-Avi) (5052 bp) (from 1820-2675 bp)

ctgccagcgcgctggagcaaggcgacctgaaacgtatgggcgagttgatggcggagtctcatgcctctatgcgcgatgatttcgaaatcaccgtgccgcaaattgac
gacggtcgcgcgacctcgttccgctggactttgcatacccgctcaactaccgcctcagagtacggagatacgcgctactaaagctttagtggcacggcgtttaactg

galK

1,820 1,840 1,860 1,880 1,900 1,920

actctggtagaaatcgtcaaagctgtgattggcgacaaaggtggcgtacgcatgaccggcggcggatttggcggctgtatcgtcgcgctgatcccggaagagctggt
tgagaccatctttagcagtttcgacactaaccgctgtttccaccgcatgcgtactggccgccgcctaaaccgccgacatagcagcgcgactagggccttctcgacca

galK

1,940 1,960 1,980 2,000 2,020

gcctgccgtacagcaagctgtcgctgaacaatatgaagcaaaaacaggtattaaagagactttttacgtttgtaaaccatcacaaggagcaggacagtgctgaggat
cggacggcatgtcgttcgacagcgacttgttatacttcgtttttgtccataatttctctgaaaaatgcaaacatttggtagtgttcctcgtcctgtcacgactccta

galK

2,040 2,060 2,080 2,100 2,120 2,140

ccactagttctagagcggccgcgaagttcctatactttctagagaataggaacttctaactaaccatacatatttaaattttcaggtgctgaagtcaagtttgaagg
ggtgatcaagatctcgccggcgcttcaaggatatgaaagatctcttatccttgaagattgattggtatgtataaatttaaaagtccacgacttcagttcaaacttcc

galK FRT site GFP

2,160 2,180 2,200 2,220 2,240

tgatacccttgttaatagaatcgagttaaaaggtattgattttaaagaagatggaaacattcttggacacaaattggaatacaactataactcacacaatgtataca
actatgggaacaattatcttagctcaattttccataactaaaatttcttctacctttgtaagaacctgtgtttaaccttatgttgatattgagtgtgttacatatgt

GFP

2,260 2,280 2,300 2,320 2,340

tcatggcagacaaacaaaagaatggaatcaaagttgtaagtttaaacatgattttactaactaactaatctgatttaaattttcagaacttcaaaattagacacaac
agtaccgtctgtttgttttcttaccttagtttcaacattcaaatttgtactaaaatgattgattgattagactaaatttaaaagtcttgaagttttaatctgtgttg

GFP GFP

2,360 2,380 2,400 2,420 2,440 2,460

attgaagatggaagcgttcaactagcagaccattatcaacaaaatactcCAATTGGCGATGGCCCTGTCCTTTTACCAGACAACCATTACCTGTCCACACAATCTGC
taacttctaccttcgcaagttgatcgtctggtaatagttgttttatgagGTTAACCGCTACCGGGACAGGAAAATGGTCTGTTGGTAATGGACAGGTGTGTTAGACG

GFP GFP

2,480 2,500 2,520 2,540 2,560

CCTTTCGAAAGATCCCAACGAAAAGAGAGACCACATGGTCCTTCTTGAGTTTGTAACAGCTGCTGGGATTACACATGGCATGGATGAACTATACAAAtctggaggag
GGAAAGCTTTCTAGGGTTGCTTTTCTCTCTGGTGTACCAGGAAGAACTCAAACATTGTCGACGACCCTAATGTGTACCGTACCTACTTGATATGTTTagacctcctc

GFP

2,580 2,600 2,620 2,640 2,660

pMB72 (C-term GFP-2xTEV-Avi) (5052 bp) (from 2676-3638 bp)

gatctGAAAATCTTTATTTCCAAGGAtccGAgAAcCTTTAcTTCCAAGGAtctggaggaggatctGGACTTAATGATATTTTCGAAGCTCAgAAgATTGAATGGCAT
ctagaCTTTTAGAAATAAAGGTTCCTaggCTcTTgGAAATgAAGGTTCCTagacctcctcctagaCCTGAATTACTATAAAAGCTTCGAGTcTTcTAACTTACCGTA

2 TEV sites | Avi tag

2,680 2,700 2,720 2,740 2,760 2,780

GAAtaaAGATCTGTCGACCTGCAGGCATGCAAGCTTGGCGTAATCATGGTCATAGCTGTTTCCTGTGTGAAATTGTTATCCGCTCACAATTCCACACAACATACGAG
CTTattTCTAGACAGCTGGACGTCCGTACGTTCGAACCGCATTAGTACCAGTATCGACAAAGGACACACTTTAACAATAGGCGAGTGTTAAGGTGTGTTGTATGCTC

M13-rev | LacO

2,800 2,820 2,840 2,860 2,880

CCGGAAGCATAAAGTGTAAAGCCTGGGGTGCCTAATGAGTGAGCTAACTCACATTAATTGCGTTGCGCTCACTGCCCGCTTTCCAGTCGGGAAACCTGTCGTGCCAG
GGCCTTCGTATTTCACATTTCGGACCCCACGGATTACTCACTCGATTGAGTGTAATTAACGCAACGCGAGTGACGGGCGAAAGGTCAGCCCTTTGGACAGCACGGTC

2,900 2,920 2,940 2,960 2,980

CTGCATTAATGAATCGGCCAACGCGCGGGGAGAGGCGGTTTGCGTATTGGGCGCTCTTCCGCTTCCTCGCTCACTGACTCGCTGCGCTCGGTCGTTCGGCTGCGGCG
GACGTAATTACTTAGCCGGTTGCGCGCCCCTCTCCGCCAAACGCATAACCCGCGAGAAGGCGAAGGAGCGAGTGACTGAGCGACGCGAGCCAGCAAGCCGACGCCGC

3,000 3,020 3,040 3,060 3,080 3,100

AGCGGTATCAGCTCACTCAAAGGCGGTAATACGGTTATCCACAGAATCAGGGGATAACGCAGGAAAGAACATGTGAGCAAAAGGCCAGCAAAAGGCCAGGAACCGTA
TCGCCATAGTCGAGTGAGTTTCCGCCATTATGCCAATAGGTGTCTTAGTCCCCTATTGCGTCCTTTCTTGTACACTCGTTTTCCGGTCGTTTTCCGGTCCTTGGCAT

3,120 3,140 3,160 3,180 3,200

AAAAGGCCGCGTTGCTGGCGTTTTTCCATAGGCTCCGCCCCCCTGACGAGCATCACAAAAATCGACGCTCAAGTCAGAGGTGGCGAAACCCGACAGGACTATAAAGA
TTTTCCGGCGCAACGACCGCAAAAAGGTATCCGAGGCGGGGGGACTGCTCGTAGTGTTTTTAGCTGCGAGTTCAGTCTCCACCGCTTTGGGCTGTCCTGATATTTCT

ColE1 origin

3,220 3,240 3,260 3,280 3,300

TACCAGGCGTTTCCCCCTGGAAGCTCCCTCGTGCGCTCTCCTGTTCCGACCCTGCCGCTTACCGGATACCTGTCCGCCTTTCTCCCTTCGGGAAGCGTGGCGCTTTC
ATGGTCCGCAAAGGGGGACCTTCGAGGGAGCACGCGAGAGGACAAGGCTGGGACGGCGAATGGCCTATGGACAGGCGGAAAGAGGGAAGCCCTTCGCACCGCGAAAG

ColE1 origin

3,320 3,340 3,360 3,380 3,400 3,420

TCATAGCTCACGCTGTAGGTATCTCAGTTCGGTGTAGGTCGTTCGCTCCAAGCTGGGCTGTGTGCACGAACCCCCCGTTCAGCCCGACCGCTGCGCCTTATCCGGTA
AGTATCGAGTGCGACATCCATAGAGTCAAGCCACATCCAGCAAGCGAGGTTCGACCCGACACACGTGCTTGGGGGGCAAGTCGGGCTGGCGACGCGGAATAGGCCAT

ColE1 origin

3,440 3,460 3,480 3,500 3,520

ACTATCGTCTTGAGTCCAACCCGGTAAGACACGACTTATCGCCACTGGCAGCAGCCACTGGTAACAGGATTAGCAGAGCGAGGTATGTAGGCGGTGCTACAGAGTTC
TGATAGCAGAACTCAGGTTGGGCCATTCTGTGCTGAATAGCGGTGACCGTCGTCGGTGACCATTGTCCTAATCGTCTCGCTCCATACATCCGCCACGATGTCTCAAG

ColE1 origin

3,540 3,560 3,580 3,600 3,620

pMB72 (C-term GFP-2xTEV-Avi) (5052 bp) (from 3639-4494 bp)

TTGAAGTGGTGGCCTAACTACGGCTACACTAGAAGAACAGTATTTGGTATCTGCGCTCTGCTGAAGCCAGTTACCTTCGGAAAAAGAGTTGGTAGCTCTTGATCCGG
AACTTCACCACCGGATTGATGCCGATGTGATCTTCTTGTCATAAACCATAGACGCGAGACGACTTCGGTCAATGGAAGCCTTTTTCTCAACCATCGAGAACTAGGCC

ColE1 origin

3,640 3,660 3,680 3,700 3,720 3,740

CAAACAAACCACCGCTGGTAGCGGTGGTTTTTTTGTTTGCAAGCAGCAGATTACGCGCAGAAAAAAAGGATCTCAAGAAGATCCTTTGATCTTTTCTACGGGGTCTG
GTTTGTTTGGTGGCGACCATCGCCACCAAAAAAACAAACGTTCGTCGTCTAATGCGCGTCTTTTTTTCCTAGAGTTCTTCTAGGAAACTAGAAAAGATGCCCCAGAC

ColE1 origin

3,760 3,780 3,800 3,820 3,840

ACGCTCAGTGGAACGAAAACTCACGTTAAGGGATTTTGGTCATGAGATTATCAAAAAGGATCTTCACCTAGATCCTTTTAAATTAAAAATGAAGTTTTAAATCAATC
TGCGAGTCACCTTGCTTTTGAGTGCAATTCCCTAAAACCAGTACTCTAATAGTTTTTCCTAGAAGTGGATCTAGGAAAATTTAATTTTTACTTCAAAATTTAGTTAG

ColE1 origin

3,860 3,880 3,900 3,920 3,940

TAAAGTATATATGAGTAAACTTGGTCTGACAGTTACCAATGCTTAATCAGTGAGGCACCTATCTCAGCGATCTGTCTATTTCGTTCATCCATAGTTGCCTGACTCCC
ATTTCATATATACTCATTTGAACCAGACTGTCAATGGTTACGAATTAGTCACTCCGTGGATAGAGTCGCTAGACAGATAAAGCAAGTAGGTATCAACGGACTGAGGG

AmpR

3,960 3,980 4,000 4,020 4,040 4,060

CGTCGTGTAGATAACTACGATACGGGAGGGCTTACCATCTGGCCCCAGTGCTGCAATGATACCGCGAGACCCACGCTCACCGGCTCCAGATTTATCAGCAATAAACC
GCAGCACATCTATTGATGCTATGCCCTCCCGAATGGTAGACCGGGGTCACGACGTTACTATGGCGCTCTGGGTGCGAGTGGCCGAGGTCTAAATAGTCGTTATTTGG

AmpR

4,080 4,100 4,120 4,140 4,160

AGCCAGCCGGAAGGGCCGAGCGCAGAAGTGGTCCTGCAACTTTATCCGCCTCCATCCAGTCTATTAATTGTTGCCGGGAAGCTAGAGTAAGTAGTTCGCCAGTTAAT
TCGGTCGGCCTTCCCGGCTCGCGTCTTCACCAGGACGTTGAAATAGGCGGAGGTAGGTCAGATAATTAACAACGGCCCTTCGATCTCATTCATCAAGCGGTCAATTA

AmpR

4,180 4,200 4,220 4,240 4,260 4,280

AGTTTGCGCAACGTTGTTGCCATTGCTACAGGCATCGTGGTGTCACGCTCGTCGTTTGGTATGGCTTCATTCAGCTCCGGTTCCCAACGATCAAGGCGAGTTACATG
TCAAACGCGTTGCAACAACGGTAACGATGTCCGTAGCACCACAGTGCGAGCAGCAAACCATACCGAAGTAAGTCGAGGCCAAGGGTTGCTAGTTCCGCTCAATGTAC

AmpR

4,300 4,320 4,340 4,360 4,380

ATCCCCCATGTTGTGCAAAAAAGCGGTTAGCTCCTTCGGTCCTCCGATCGTTGTCAGAAGTAAGTTGGCCGCAGTGTTATCACTCATGGTTATGGCAGCACTGCATA
TAGGGGGTACAACACGTTTTTTCGCCAATCGAGGAAGCCAGGAGGCTAGCAACAGTCTTCATTCAACCGGCGTCACAATAGTGAGTACCAATACCGTCGTGACGTAT

AmpR

4,400 4,420 4,440 4,460 4,480

pMB72 (C-term GFP-2xTEV-Avi) (5052 bp) (from 4495-5052 bp)

```
ATTCTCTTACTGTCATGCCATCCGTAAGATGCTTTTCTGTGACTGGTGAGTACTCAACCAAGTCATTCTGAGAATAGTGTATGCGGCGACCGAGTTGCTCTTGCCCG
TAAGAGAATGACAGTACGGTAGGCATTCTACGAAAAGACACTGACCACTCATGAGTTGGTTCAGTAAGACTCTTATCACATACGCCGCTGGCTCAACGAGAACGGGC
AmpR
4,500 4,520 4,540 4,560 4,580 4,600

GCGTCAATACGGGATAATACCGCGCCACATAGCAGAACTTTAAAAGTGCTCATCATTGGAAAACGTTCTTCGGGGCGAAAACTCTCAAGGATCTTACCGCTGTTGAG
CGCAGTTATGCCCTATTATGGCGCGGTGTATCGTCTTGAAATTTTCACGAGTAGTAACCTTTTGCAAGAAGCCCCGCTTTTGAGAGTTCCTAGAATGGCGACAACTC
AmpR
4,620 4,640 4,660 4,680 4,700

ATCCAGTTCGATGTAACCCACTCGTGCACCCAACTGATCTTCAGCATCTTTTACTTTCACCAGCGTTTCTGGGTGAGCAAAAACAGGAAGGCAAAATGCCGCAAAAA
TAGGTCAAGCTACATTGGGTGAGCACGTGGGTTGACTAGAAGTCGTAGAAAATGAAAGTGGTCGCAAAGACCCACTCGTTTTTGTCCTTCCGTTTTACGGCGTTTTT
4,720 4,740 4,760 4,780 4,800

AGGGAATAAGGGCGACACGGAAATGTTGAATACTCATACTCTTCCTTTTTCAATATTATTGAAGCATTTATCAGGGTTATTGTCTCATGAGCGGATACATATTTGAA
TCCCTTATTCCCGCTGTGCCTTTACAACTTATGAGTATGAGAAGGAAAAAGTTATAATAACTTCGTAAATAGTCCCAATAACAGAGTACTCGCCTATGTATAAACTT
4,820 4,840 4,860 4,880 4,900 4,920

TGTATTTAGAAAAATAAACAAATAGGGGTTCCGCGCACATTTCCCCGAAAAGTGCCACCTGACGTCTAAGAAACCATTATTATCATGACATTAACCTATAAAAATAG
ACATAAATCTTTTTATTTGTTTATCCCCAAGGCGCGTGTAAAGGGGCTTTTCACGGTGGACTGCAGATTCTTTGGTAATAATAGTACTGTAATTGGATATTTTTATC
4,940 4,960 4,980 5,000 5,020

GCGTATCACGAGGCCCTTTCGTC
CGCATAGTGCTCCGGGAAAGCAG
5,030 5,040 5,050
```

pMB73 (Pwrt-2::BirA) (5999 bp)

AAGCTTGGGCTGCAGCAGGTCGACTCCAcGTAATTtcacaacatccgggaaaagaagtgaaaaaaggactcgagcaactgtacaagaaaattgagaagaacctggtt
TTCGAACCCGACGTCGTCCAGCTGAGGTgCATTAAagtgttgtaggcccttttcttcacttttttcctgagctcgttgacatgttcttttaactcttcttggaccaa

pwrt-2

20 40 60 80 100

gccaattcatcactttgcaagttgtatggagagatatgcaagagcaatttgtcaagcaaattgcggaatacaacaaactgattctcacttgttacccaggctcgaa
cggttaagtagtgaaaacgttcaacatacctctctatacgttctcgttaaacagttcgtttaacgccttatgttgtttgactaagagtgaacaatgggtccgagctt

pwrt-2

120 140 160 180 200

gattgagctcgaagtttccactgacactgtacttcagttcttctcagaaattgctcaacaacactaatattacgacgaataattttattgaattttgttgttttaat
ctaactcgagcttcaaaggtgactgtgacatgaagtcaagaagagtctttaacgagttgttgtgattataatgctgcttattaaaataacttaaaacaacaaaatta

pwrt-2

220 240 260 280 300 320

tcatttaaaatttagttttaatttatcagaactctaatacttactgaaccatgctttgtaatatatttatttatctattctgtaataggtgaacttcgttacattac
agtaaattttaaatcaaaattaaatagtcttgagattatgaatgacttggtacgaaacattatataaataaatagataagacattatccacttgaagcaatgtaatg

pwrt-2

340 360 380 400 420

tgatgtctgtaatataaatttacgggtgttccccattcgaaaatagtagtaatgcttgctttttgaaaaataaatgtcttgcttggtgacttttatttcacgtatt
actacagacattatatttaaatgcccacaaggggtaagcttttatcatcattacgaacgaaaaactttttatttacagaacgaaccactgaaaataaagtgcataa

pwrt-2

440 460 480 500 520

ttcaaatgctcacaaaaatttcacggaactgacttcttggttacacatttgtagaaattttgactgaatgtttgcattgaactggtcaacttaagaatctgacatgc
aagtttacgagtgtttttaaagtgccttgactgaagaaccaatgtgtaaacatctttaaaactgacttacaaacgtaacttgaccagttgaattcttagactgtacg

pwrt-2

540 560 580 600 620 640

aaatggtgtgttttctgaagaacacactttggcaacttttaataaaaaaggggtgtaacacgatttaaatctaataaaaacaacttctataatgttattccgtttat
tttaccacacaaaagacttcttgtgtgaaaccgttgaaaattatttttccccacattgtgctaaatttagattatttttgttgaagatattacaataaggcaaata

pwrt-2

660 680 700 720 740

atctcactttatgttttacattttactcttatatacatttttttgactcatttatacagggtggtccataattatggaccaccctgtacttatatcttatttaaaact
tagagtgaaatacaaaatgtaaaatgagaatatatgtaaaaaaactgagtaaatatgtcccaccaggtattaatacctggtgggacatgaatatagaataaattttga

pwrt-2

760 780 800 820 840

pMB73 (Pwrt-2::BirA) (5999 bp) (from 857-1712 bp)

```
atttcatgtaagaattgttttaacagcgcatttttttttcatcactcgatttaaaggtaaaattgataggccgcaatctattttggaaaacttcagaacaaaatttaa
taaagtacattcttaacaaaattgtcgcgtaaaaaaaaagtagtgagctaaatttccattttaactatccggcgttagataaaaccttttgaagtcttgttttaaatt
                                  pwrt-2
  860        880        900        920        940        960

cattttaagaggtaactgtgaaggtttataagagaattataaaccgatctaaccactaaacatggtaacgtagaaaaagtaaaattaggggagggggggatgtttatt
gtaaaattctccattgacacttccaaatattctcttaatatttggctagattggtgatttgtaccattgcatctttttcattttaatcccctccccccctacaaataa
                                  pwrt-2
        980        1,000        1,020        1,040        1,060

tattcttggttttcaattcttatcagactctgtaaagtaaaatgaacataacttgttcgaatagtgtaggaatttcaaacaagctacaaatctagctccacatacgc
ataagaaccaaaagttaagaatagtctgagacatttcattttacttgtattgaacaagcttatcacatccttaaagtttgttcgatgtttagatcgaggtgtatgcg
                                  pwrt-2
     1,080        1,100        1,120        1,140        1,160

gtggaatagccggtcataaatcgtcttccgttaaccgcatggctagatccacaccgattccatttttcttgaataaaacaatagcttgatgaaaattgggtagccac
caccttatcggccagtatttagcagaaggcaattggcgtaccgatctaggtgtggctaaggtaaaaagaacttattttgttatcgaactacttttaacccatcggtg
                                  pwrt-2
 1,180        1,200        1,220        1,240        1,260        1,280

cgaaaaaactgattcgcgatccggattattggtgacaaacatacggcaagtgaaagtgttgcactgatagaaaccgatacgagaatatgccttttgggcccccccccc
gctttttttgactaagcgctaggcctaataaccactgtttgtatgccgttcactttcacaacgtgactatctttggctatgctcttatacggaaaacccgggggggggg
                                  pwrt-2
          1,300        1,320        1,340        1,360        1,380

cccccccccctacggcttcactcccggtgttgtagatctgactttcacttgccttatctcgaggaatctggagaggagaaggagggacaaccgatccgcgcgcgcgta
ggggggggggatgccgaagtgagggccacaacatctagactgaaagtgaacggaatagagctccttagacctctcctcttcctccctgttggctaggcgcgcgcgcat
                                  pwrt-2
     1,400        1,420        1,440        1,460        1,480

acaatacgggagatatagaggaaaagcagcaaaaagacattttgatgaccttattttcttgtcgttgtcgagacggccattgtcccttccacgcccagcttttcatt
tgttatgccctctatatctccttttcgtcgtttttctgtaaaactactggaataaaagaacagcaacagctctgccggtaacagggaaggtgcgggtcgaaaagtaa
                                  pwrt-2
 1,500        1,520        1,540        1,560        1,580        1,600

gtagttcaaatcgcttctcattgacaatCAACCTGCCAATTGTTTCTCGGgatcCCCGGGTGCCCTATAGTGAGTCGTATTGGTACCaaaaATGGAGCAGAAGCTC
catcaagtttagcgaagagtaactgttaGTTGGACGGTTAACAAAGAGCCctagGGGCCCACGGGATATCACTCAGCATAACCATGGttttTACCTCGTCTTCGAG
                 pwrt-2
        1,620        1,640        1,660        1,680        1,700
```

pMB73 (Pwrt-2::BirA) (5999 bp) (from 1713-2568 bp)

ATCTCTGAGGAAGACCTCGGAGGAGAGCAGAAACTCATCTCTGAAGAGGACCTCATGAAGGATAATACTGTTCCATTGAAGTTGATTGCTTTGCTTGCAAATGGAGA
TAGAGACTCCTTCTGGAGCCTCCTCTCGTCTTTGAGTAGAGACTTCTCCTGGAGTACTTCCTATTATGACAAGGTAACTTCAACTAACGAAACGAACGTTTACCTCT

MYC-tag 1 MYC-tag 2 BirA

1,720 1,740 1,760 1,780 1,800

ATTTCATTCTGGAGAACAACTTGGAGAGACGTTGGGAATGTCGCGTGCTGCAATTAACAAGCATATTCAAACAgtaagtttaaacagttcggtactaactaaccata
TAAAGTAAGACCTCTTGTTGAACCTCTCTGCAACCCTTACAGCGCACGACGTTAATTGTTCGTATAAGTTTGTcattcaaatttgtcaagccatgattgattggtat

BirA

1,820 1,840 1,860 1,880 1,900 1,920

catatttaaattttcagTTGAGAGACTGGGGAGTTGACGTTTTTACGGTTCCAGGAAAGGGATATTCGCTTCCAGAGCCAATTCAGCTCTTGAATGCAAAGCAAATT
gtataaatttaaaagtcAACTCTCTGACCCCTCAACTGCAAAAATGCCAAGGTCCTTTCCCTATAAGCGAAGGTCTCGGTTAAGTCGAGAACTTACGTTTCGTTTAA

BirA

1,940 1,960 1,980 2,000 2,020

TTGGGACAGCTTGATGGTGGTAGTGTTGCAGTACTTCCGGTTATAGATAGTACAAATCAGTACTTGCTTGATCGAATAGGAGAATTGAAATCAGGAGATGCTTGCAT
AACCCTGTCGAACTACCACCATCACAACGTCATGAAGGCCAATATCTATCATGTTTAGTCATGAACGAACTAGCTTATCCTCTTAACTTTAGTCCTCTACGAACGTA

BirA

2,040 2,060 2,080 2,100 2,120 2,140

CGCTGAGTATCAGCAGGCTGGACGAGGACGAAGAGGACGTAAGTGGTTCTgtaagtttaaacatatatatactaactaaccctgattatttaaattttcagCGCCTT
GCGACTCATAGTCGTCCGACCTGCTCCTGCTTCTCCTGCATTCACCAAGAcattcaaatttgtatatatatgattgattgggactaataaatttaaaagtcGCGGAA

BirA

2,160 2,180 2,200 2,220 2,240

TCGGAGCCAATCTCTATTTGAGTATGTTCTGGCGTCTTGAACAAGGTCCGGCCGCTGCTATCGGACTTTCATTGGTTATTGGAATTGTGATGGCAGAAGTTCTCCGA
AGCCTCGGTTAGAGATAAACTCATACAAGACCGCAGAACTTGTTCCAGGCCGGCGACGATAGCCTGAAAGTAACCAATAACCTTAACACTACCGTCTTCAAGAGGCT

BirA

2,260 2,280 2,300 2,320 2,340

AAACTTGGTGCAGATAAGGTGAGAGTCAAGTGGCCTAACGATCTTTATCTTCAAGATAGAAAATTGGCCGGAATATTGgtaagtttaaacatatatatactaactaa
TTTGAACCACGTCTATTCCACTCTCAGTTCACCGGATTGCTAGAAATAGAAGTTCTATCTTTTAACCGGCCTTATAACcattcaaatttgtatatatatgattgatt

BirA

2,360 2,380 2,400 2,420 2,440 2,460

ccctgattatttaaattttcagGTTGAGCTTACTGGAAAGACGGGAGATGCTGCTCAAATTGTGATTGGAGCTGGTATCAATATGGCAATGAGAAGAGTAGAAGAGT
gggactaataaatttaaaagtcCAACTCGAATGACCTTTCTGCCCTCTACGACGAGTTTAACACTAACCTCGACCATAGTTATACCGTTACTCTTCTCATCTTCTCA

BirA

2,480 2,500 2,520 2,540 2,560

pMB73 (Pwrt-2::BirA) (5999 bp) (from 2569-3531 bp)

```
CTGTTGTTAATCAAGGTTGGATCACACTTCAAGAGGCAGGAATCAACCTTGATAGAAATACGTTGGCCGCCATGCTTATCCGAGAATTGCGAGCTGCACTCGAACTC
GACAACAATTAGTTCCAACCTAGTGTGAAGTTCTCCGTCCTTAGTTGGAACTATCTTTATGCAACCGGCGGTACGAATAGGCTCTTAACGCTCGACGTGAGCTTGAG
                                              BirA
         2,580                        2,600                     2,620                     2,640                     2,660

TTCGAACAAGAAGGTCTTgtaagtttaaacatatatatactaactaaccctgattatttaaattttcagGCCCCTTACCTCAGTAGATGGGAGAAACTTGACAATTT
AAGCTTGTTCTTCCAGAAcattcaaatttgtatatatatgattgattgggactaataaatttaaaagtcCGGGGAATGGAGTCATCTACCCTCTTTGAACTGTTAAA
      BirA                                                                    BirA
   2,680                     2,700                     2,720                     2,740                     2,760                     2,780

TATCAATCGACCAGTGAAGTTGATAATTGGAGATAAGGAAATTTTTGGTATCAGTCGAGGAATTGATAAGCAAGGAGCTCTTCTCCTTGAGCAAGATGGAATCATTA
ATAGTTAGCTGGTCACTTCAACTATTAACCTCTATTCCTTTAAAAACCATAGTCAGCTCCTTAACTATTCGTTCCTCGAGAAGAGGAACTCGTTCTACCTTAGTAAT
                                              BirA
             2,800                     2,820                     2,840                     2,860                     2,880

AACCATGGATGGGTGGAGAGATTTCTTTGCGATCTGCTGAAAAGTAAGAATTCGCTAGCCGGCCATACAAGTAATCCGGATGATCGACGCCaACGTCGTTGAATTTT
TTGGTACCTACCCACCTCTCTAAAGAAACGCTAGACGACTTTTCATTCTTAAGCGATCGGCCGGTATGTTCATTAGGCCTACTAGCTGCGGtTGCAGCAACTTAAAA
                  BirA
       2,900                     2,920                     2,940                     2,960                     2,980

CAAATTTTAAATACTGAATATTTGTTTTTTTTCCTATTATTTATTTATTCTCTTTGTGTTTTTTTTCTTGCTTTCTAAAAAATTAATTCAATCCAAATCTAAacatt
GTTTAAAATTTATGACTTATAAACAAAAAAAAGGATAATAAATAAATAAGAGAAACACAAAAAAAAGAACGAAAGATTTTTTAATTAAGTTAGGTTTAGATTtgtaa
                                           let-858 UTR
3,000                     3,020                     3,040                     3,060                     3,080                     3,100

tttttttctctttccgtctcccaattcgtattccgctcctctcatctgaacacaatgtgcaagtttatttatcttctcgctttcatttcattaggacgtgggggaa
aaaaaaagagaaaggcagagggttaagcataaggcgaggagagtagacttgtgttacacgttcaaataaatagaagagcgaaagtaaagtaatcctgcaccccctt
            3,120                     3,140                     3,160                     3,180                     3,200

ttggtggaagggggaaacacacaaaaggatgatggaaatgaaataaggacacacaatatgcaacaacattcaattcagaaatatggaggaaggtttaaaagaaaaca
aaccaccttccccctttgtgtgttttcctactacctttactttattcctgtgtgttatacgttgttgtaagttaagtctttatacctccttccaaattttcttttgt
      3,220                     3,240                     3,260                     3,280                     3,300

taaaaatatatagaggaggaaggaaaactagtaaaaaataagcaaagaaattaggcgaacgatgAGAATTGTCCTCGCTTGGGCCCTTTCGTCTCGCGCGTTTCGGT
atttttatatatctcctccttccttttgatcattttttattcgtttctttaatccgcttgctacTCTTAACAGGAGCGAACCCGGGAAAGCAGAGCGCGCAAAGCCA
3,320                     3,340                     3,360                     3,380                     3,400                     3,420

GATGACGGTGAAAACCTCTGACACATGCAGCTCCCGGAGACGGTCACAGCTTGTCTGTAAGCGGATGCCGGGAGCAGACAAGCCCGTCAGGGCGCGTCAGCGGGTGT
CTACTGCCACTTTTGGAGACTGTGTACGTCGAGGGCCTCTGCCAGTGTCGAACAGACATTCGCCTACGGCCCTCGTCTGTTCGGGCAGTCCCGCGCAGTCGCCCACA
            3,440                     3,460                     3,480                     3,500                     3,520
```

pMB73 (Pwrt-2::BirA) (5999 bp) (from 3532-4494 bp)

TGGCGGGTGTCGGGGCTGGCTTAACTATGCGGCATCAGAGCAGATTGTACTGAGAGTGCACCATATGCGGTGTGAAATACCGCACAGATGCGTAAGGAGAAAATACC
ACCGCCCACAGCCCCGACCGAATTGATACGCCGTAGTCTCGTCTAACATGACTCTCACGTGGTATACGCCACACTTTATGGCGTGTCTACGCATTCCTCTTTTATGG

3,540 3,560 3,580 3,600 3,620

GCATCAGGCGGCCTTAAGGGCCTCGTGATACGCCTATTTTTATAGGTTAATGTCATGATAATAATGGTTTCTTAGACGTCAGGTGGCACTTTTCGGGGAAATGTGCG
CGTAGTCCGCCGGAATTCCCGGAGCACTATGCGGATAAAAATATCCAATTACAGTACTATTATTACCAAAGAATCTGCAGTCCACCGTGAAAAGCCCCTTTACACGC

3,640 3,660 3,680 3,700 3,720 3,740

CGGAACCCCTATTTGTTTATTTTTCTAAATACATTCAAATATGTATCCGCTCATGAGACAATAACCCTGATAAATGCTTCAATAATATTGAAAAAGGAAGAGTATGA
GCCTTGGGGATAAACAAATAAAAAGATTTATGTAAGTTTATACATAGGCGAGTACTCTGTTATTGGGACTATTTACGAAGTTATTATAACTTTTTCCTTCTCATACT

3,760 3,780 3,800 3,820 3,840

GTATTCAACATTTCCGTGTCGCCCTTATTCCCTTTTTTGCGGCATTTTGCCTTCCTGTTTTTGCTCACCCAGAAACGCTGGTGAAAGTAAAAGATGCTGAAGATCAG
CATAAGTTGTAAAGGCACAGCGGGAATAAGGGAAAAAACGCCGTAAAACGGAAGGACAAAAACGAGTGGGTCTTTGCGACCACTTTCATTTTCTACGACTTCTAGTC

3,860 3,880 3,900 3,920 3,940

TTGGGTGCACGAGTGGGTTACATCGAACTGGATCTCAACAGCGGTAAGATCCTTGAGAGTTTTCGCCCCGAAGAACGTTTTCCAATGATGAGCACTTTTAAAGTTCT
AACCCACGTGCTCACCCAATGTAGCTTGACCTAGAGTTGTCGCCATTCTAGGAACTCTCAAAAGCGGGGCTTCTTGCAAAAGGTTACTACTCGTGAAAATTTCAAGA
AmpR

3,960 3,980 4,000 4,020 4,040 4,060

GCTATGTGGCGCGGTATTATCCCGTATTGACGCCGGGCAAGAGCAACTCGGTCGCCGCATACACTATTCTCAGAATGACTTGGTTGAGTACTCACCAGTCACAGAAA
CGATACACCGCGCCATAATAGGGCATAACTGCGGCCCGTTCTCGTTGAGCCAGCGGCGTATGTGATAAGAGTCTTACTGAACCAACTCATGAGTGGTCAGTGTCTTT
AmpR

4,080 4,100 4,120 4,140 4,160

AGCATCTTACGGATGGCATGACAGTAAGAGAATTATGCAGTGCTGCCATAACCATGAGTGATAACACTGCGGCCAACTTACTTCTGACAACGATCGGAGGACCGAAG
TCGTAGAATGCCTACCGTACTGTCATTCTCTTAATACGTCACGACGGTATTGGTACTCACTATTGTGACGCCGGTTGAATGAAGACTGTTGCTAGCCTCCTGGCTTC
AmpR

4,180 4,200 4,220 4,240 4,260 4,280

GAGCTAACCGCTTTTTTGCACAACATGGGGGATCATGTAACTCGCCTTGATCGTTGGGAACCGGAGCTGAATGAAGCCATACCAAACGACGAGCGTGACACCACGAT
CTCGATTGGCGAAAAAACGTGTTGTACCCCCTAGTACATTGAGCGGAACTAGCAACCCTTGGCCTCGACTTACTTCGGTATGGTTTGCTGCTCGCACTGTGGTGCTA
AmpR

4,300 4,320 4,340 4,360 4,380

GCCTGTAGCAATGGCAACAACGTTGCGCAAACTATTAACTGGCGAACTACTTACTCTAGCTTCCCGGCAACAATTAATAGACTGGATGGAGGCGGATAAAGTTGCAG
CGGACATCGTTACCGTTGTTGCAACGCGTTTGATAATTGACCGCTTGATGAATGAGATCGAAGGGCCGTTGTTAATTATCTGACCTACCTCCGCCTATTTCAACGTC
AmpR

4,400 4,420 4,440 4,460 4,480

pMB73 (Pwrt-2::BirA) (5999 bp) (from 4495-5350 bp)

GACCACTTCTGCGCTCGGCCCTTCCGGCTGGCTGGTTTATTGCTGATAAATCTGGAGCCGGTGAGCGTGGGTCTCGCGGTATCATTGCAGCACTGGGGCCAGATGGT
CTGGTGAAGACGCGAGCCGGGAAGGCCGACCGACCAAATAACGACTATTTAGACCTCGGCCACTCGCACCCAGAGCGCCATAGTAACGTCGTGACCCCGGTCTACCA
AmpR
4,500 4,520 4,540 4,560 4,580 4,600

AAGCCCTCCCGTATCGTAGTTATCTACACGACGGGGAGTCAGGCAACTATGGATGAACGAAATAGACAGATCGCTGAGATAGGTGCCTCACTGATTAAGCATTGGTA
TTCGGGAGGGCATAGCATCAATAGATGTGCTGCCCCTCAGTCCGTTGATACCTACTTGCTTTATCTGTCTAGCGACTCTATCCACGGAGTGACTAATTCGTAACCAT
AmpR
4,620 4,640 4,660 4,680 4,700

ACTGTCAGACCAAGTTTACTCATATATACTTTAGATTGATTTAAAACTTCATTTTTAATTTAAAAGGATCTAGGTGAAGATCCTTTTTGATAATCTCATGACCAAAA
TGACAGTCTGGTTCAAATGAGTATATATGAAATCTAACTAAATTTTGAAGTAAAAATTAAATTTTCCTAGATCCACTTCTAGGAAAAACTATTAGAGTACTGGTTTT
4,720 4,740 4,760 4,780 4,800

TCCCTTAACGTGAGTTTTCGTTCCACTGAGCGTCAGACCCCGTAGAAAAGATCAAAGGATCTTCTTGAGATCCTTTTTTTCTGCGCGTAATCTGCTGCTTGCAAACA
AGGGAATTGCACTCAAAAGCAAGGTGACTCGCAGTCTGGGGCATCTTTTCTAGTTTCCTAGAAGAACTCTAGGAAAAAAAGACGCGCATTAGACGACGAACGTTTGT
ColE1 origin
4,820 4,840 4,860 4,880 4,900 4,920

AAAAAACCACCGCTACCAGCGGTGGTTTGTTTGCCGGATCAAGAGCTACCAACTCTTTTTCCGAAGGTAACTGGCTTCAGCAGAGCGCAGATACCAAATACTGTCCT
TTTTTTGGTGGCGATGGTCGCCACCAAACAAACGGCCTAGTTCTCGATGGTTGAGAAAAAGGCTTCCATTGACCGAAGTCGTCTCGCGTCTATGGTTTATGACAGGA
ColE1 origin
4,940 4,960 4,980 5,000 5,020

TCTAGTGTAGCCGTAGTTAGGCCACCACTTCAAGAACTCTGTAGCACCGCCTACATACCTCGCTCTGCTAATCCTGTTACCAGTGGCTGCTGCCAGTGGCGATAAGT
AGATCACATCGGCATCAATCCGGTGGTGAAGTTCTTGAGACATCGTGGCGGATGTATGGAGCGAGACGATTAGGACAATGGTCACCGACGACGGTCACCGCTATTCA
ColE1 origin
5,040 5,060 5,080 5,100 5,120

CGTGTCTTACCGGGTTGGACTCAAGACGATAGTTACCGGATAAGGCGCAGCGGTCGGGCTGAACGGGGGGTTCGTGCACACAGCCCAGCTTGGAGCGAACGACCTAC
GCACAGAATGGCCCAACCTGAGTTCTGCTATCAATGGCCTATTCCGCGTCGCCAGCCCGACTTGCCCCCCAAGCACGTGTGTCGGGTCGAACCTCGCTTGCTGGATG
ColE1 origin
5,140 5,160 5,180 5,200 5,220 5,240

ACCGAACTGAGATACCTACAGCGTGAGCATTGAGAAAGCGCCACGCTTCCCGAAGGGAGAAAGGCGGACAGGTATCCGGTAAGCGGCAGGGTCGGAACAGGAGAGCG
TGGCTTGACTCTATGGATGTCGCACTCGTAACTCTTTCGCGGTGCGAAGGGCTTCCCTCTTTCCGCCTGTCCATAGGCCATTCGCCGTCCCAGCCTTGTCCTCTCGC
ColE1 origin
5,260 5,280 5,300 5,320 5,340

pMB73 (Pwrt-2::BirA) (5999 bp) (from 5351-5999 bp)

```
CACGAGGGAGCTTCCAGGGGGAAACGCCTGGTATCTTTATAGTCCTGTCGGGTTTCGCCACCTCTGACTTGAGCGTCGATTTTTGTGATGCTCGTCAGGGGGGCGGA
GTGCTCCCTCGAAGGTCCCCCTTTGCGGACCATAGAAATATCAGGACAGCCCAAAGCGGTGGAGACTGAACTCGCAGCTAAAAACACTACGAGCAGTCCCCCCGCCT
ColE1 origin
     5,360               5,380               5,400               5,420               5,440

GCCTATGGAAAAACGCCAGCAACGCGGCCTTTTTACGGTTCCTGGCCTTTTGCTGGCCTTTTGCTCACATGTTCTTTCCTGCGTTATCCCCTGATTCTGTGGATAAC
CGGATACCTTTTTGCGGTCGTTGCGCCGGAAAAATGCCAAGGACCGGAAAACGACCGGAAAACGAGTGTACAAGAAAGGACGCAATAGGGGACTAAGACACCTATTG
ColE1 origin
5,460               5,480               5,500               5,520               5,540               5,560

CGTATTACCGCCTTTGAGTGAGCTGATACCGCTCGCCGCAGCCGAACGACCGAGCGCAGCGAGTCAGTGAGCGAGGAAGCGGAAGAGCGCCCAATACGCAAACCGCC
GCATAATGGCGGAAACTCACTCGACTATGGCGAGCGGCGTCGGCTTGCTGGCTCGCGTCGCTCAGTCACTCGCTCCTTCGCCTTCTCGCGGGTTATGCGTTTGGCGG
           5,580               5,600               5,620               5,640               5,660

TCTCCCCGCGCGTTGGCCGATTCATTAATGCAGCTGGCACGACAGGTTTCCCGACTGGAAAGCGGGCAGTGAGCGCAACGCAATTAATGTGAGTTAGCTCACTCATT
AGAGGGGCGCGCAACCGGCTAAGTAATTACGTCGACCGTGCTGTCCAAAGGGCTGACCTTTCGCCCGTCACTCGCGTTGCGTTAATTACACTCAATCGAGTGAGTAA
    5,680               5,700               5,720               5,740               5,760

AGGCACCCCAGGCTTTACACTTTATGCTTCCGGCTCGTATGTTGTGTGGAATTGTGAGCGGATAACAATTTCACACAGGAAACAGCTATGACCATGATTACGCCAAG
TCCGTGGGGTCCGAAATGTGAAATACGAAGGCCGAGCATACAACACACCTTAACACTCGCCTATTGTTAAAGTGTGTCCTTTGTCGATACTGGTACTAATGCGGTTC
                                                LacO                 M13-rev
5,780               5,800               5,820               5,840               5,860               5,880

CTgtaagtttaaacatgatcttactaactaactattctcatttaaattttcagAGCTTAAAAATGGCTGAAATCACTCACAACGATGGATACGCTAACAACTTGGAA
GAcattcaaatttgtactagaatgattgattgataagagtaaatttaaaagtcTCGAATTTTTACCGACTTTAGTGAGTGTTGCTACCTATGCGATTGTTGAACCTT
          5,900               5,920               5,940               5,960               5,980

ATGAAAT
TACTTTA
  5,999
```

pMB76 (7778 bp)

AAGCTTGGGCTGCAGTAATTTCGAAATGTATGAACTCCAATTCTTGATAACCAATTCCTGACTCTGTAACTATTATTATTATAATCCTATTACCCACCTTTTTTCCC
TTCGAACCCGACGTCATTAAAGCTTTACATACTTGAGGTTAAGAACTATTGGTTAAGGACTGAGACATTGATAATAATAATATTAGGATAATGGGTGGAAAAAAGGG

elt-2 promoter

20 40 60 80 100

ATTCTGCTCTTAGTTCTCCCCCAACCCGTACATTTTCTCAACTAATgAAGACAATGAGAAAGTGAGAGAGAATAAGACGAACTGAAAAGAATGTGTAAACAGTTTAT
TAAGACGAGAATCAAGAGGGGGTTGGGCATGTAAAAGAGTTGATTAcTTCTGTTACTCTTTCACTCTCTCTTATTCTGCTTGACTTTTCTTACACATTTGTCAAATA

elt-2 promoter elt-2 promoter

120 140 160 180 200

TGCCTCTCGATTGTACCATCTAGTGTCTTGTCGTGTGCATAACTACTGATAACAGATCGAATGTGAAAGACCCAATAAATGCTTGGAAAGTATCAAATATGTTTAGA
ACGGAGAGCTAACATGGTAGATCACAGAACAGCACACGTATTGATGACTATTGTCTAGCTTACACTTTCTGGGTTATTTACGAACCTTTCATAGTTTATACAAATCT

elt-2 promoter

220 240 260 280 300 320

AATAGTATTTAGAGACTTATGTGGGACGAAAAAACAGGAGCATTAAGAATACAGTTGCAAACATTCATCAAGTTATAATCTAATAATATAAATcCAAAAATGTTAAT
TTATCATAAATCTCTGAATACACCCTGCTTTTTTGTCCTCGTAATTCTTATGTCAACGTTTGTAAGTAGTTCAATATTAGATTATTATATTTAgGTTTTTACAATTA

elt-2 promoter

340 360 380 400 420

GATAAAAAATGATGATCAAAGTAGGAAATTAGTGGCGAGAAAACAATAAATCATCAGAAAATGACTGATCTTGAGTAGAGTGCTTTTTTTGTAGATTTCTGATTCTA
CTATTTTTTACTACTAGTTTCATCCTTTAATCACCGCTCTTTTGTTATTTAGTAGTCTTTTACTGACTAGAACTCATCTCACGAAAAAAACATCTAAAGACTAAGAT

elt-2 promoter

440 460 480 500 520

AAACTGAAAAAAAAACTAAAAATGCTCGATGAGTCATACTAATTGAAAAATAATCAGATTGAAAACGTTAAAAATGTACTTGCTAACATACTTCCATGGTTATTTTT
TTTGACTTTTTTTTTGATTTTTACGAGCTACTCAGTATGATTAACTTTTTATTAGTCTAACTTTTGCAATTTTTACATGAACGATTGTATGAAGGTACCAATAAAAA

elt-2 promoter

540 560 580 600 620 640

GAACACAAATAAAGTTATATACATCTTTACCGGCACCAGAAGAAATCACCGCTAACCAATGTTCAGTTTTTACCTGAATATCCCTGAAAGTACAAAAAAATCAATGT
CTTGTGTTTATTTCAATATATGTAGAAATGGCCGTGGTCTTCTTTAGTGGCGATTGGTTACAAGTCAAAAATGGACTTATAGGGACTTTCATGTTTTTTTAGTTACA

elt-2 promoter

660 680 700 720 740

TCAATCAAGTTTTTGCATGCCTTCGTGTGATGGCTCGCACCAAGTATTCTGACAGTTATAAAATGTTTCCTGTGATCATGAATTTGAAAAAAAAAGAACTGATGGGA
AGTTAGTTCAAAAACGTACGGAAGCACACTACCGAGCGTGGTTCATAAGACTGTCAATATTTTACAAAGGACACTAGTACTTAAACTTTTTTTTTCTTGACTACCCT

elt-2 promoter

760 780 800 820 840

pMB76 (7778 bp) (from 857-1712 bp)

AAATGTGATATTCATAAATGAAATGTTTAATAAACAAAAAAGAACCGTCATCACAAATCTACTCTGAAACGTTCACAGTTCCTAATTGACCAATTCAGAATTTTTTG
TTTACACTATAAGTATTTACTTTACAAATTATTTGTTTTTTCTTGGCAGTAGTGTTTAGATGAGACTTTGCAAGTGTCAAGGATTAACTGGTTAAGTCTTAAAAAAC
elt-2 promoter
860 880 900 920 940 960

TACTGACTTAATAAATATAAATTTACAGTTTTAAGAAACATAAATTGAATTATATAAATAAAAAAAAAAAAACTAAAATGAAGACGTGATGGAATGTTTCACGTATT
ATGACTGAATTATTTATATTTAAATGTCAAAATTCTTTGTATTTAACTTAATATATTTATTTTTTTTTTTTTGATTTTACTTCTGCACTACCTTACAAAGTGCATAA
elt-2 promoter
980 1,000 1,020 1,040 1,060

AAAAAAGCTCTCGGACCGTTTGACATCCAGAAATTATGTGAAAGTGATTTTCTAACCCTTTGACGTCAAAAAAGTTTTTCTTTTTTGAAAGATACGCTGTTTAGAAA
TTTTTTCGAGAGCCTGGCAAACTGTAGGTCTTTAATACACTTTCACTAAAAGATTGGGAAACTGCAGTTTTTTCAAAAAGAAAAAACTTTCTATGCGACAAATCTTT
elt-2 promoter
1,080 1,100 1,120 1,140 1,160

TACTTATACAGCTGATTTTAGAATCTCCAAAAAACCTTCAATTACTTAACAAACTTTCTAAATAAAGAATTTTGATTTTGATTTTCTTTGAACTTCAAACCCCCCGT
ATGAATATGTCGACTAAAATCTTAGAGGTTTTTTGGAAGTTAATGAATTGTTTGAAAGATTTATTTCTTAAAACTAAAACTAAAAGAAACTTGAAGTTTGGGGGGCA
elt-2 promoter
1,180 1,200 1,220 1,240 1,260 1,280

TAAAAAACCAAATTAAAAAAAAGATACCAGATTAATTTTAAAGTGAAAAATATAAATTTATTCCCGACTGTTAAAAACGGTTTATAAACGGAGTCACAACACTCATA
ATTTTTTGGTTTAATTTTTTTTCTATGGTCTAATTAAAATTTCACTTTTTATATTTAAATAAGGGCTGACAATTTTTGCCAAATATTTGCCTCAGTGTTGTGAGTAT
elt-2 promoter
1,300 1,320 1,340 1,360 1,380

TACAACTATCGACACCAAATAAATAACTTCGAAAGCTAATTGTGTCATTTCGGATATAGAGAGTAGTGTGAGCAGATGTGTTTTTGCAGAACTAAATTGATGACACT
ATGTTGATAGCTGTGGTTTATTTATTGAAGCTTTCGATTAACACAGTAAAGCCTATATCTCTCATCACACTCGTCTACACAAAAACGTCTTGATTTAACTACTGTGA
elt-2 promoter
1,400 1,420 1,440 1,460 1,480

ATTATGGAATATAATGACCAAATGTTAAATGTGTTAAGGTTTGATATCAAAACCTGTATTTTCTTTTTATACAGAATTGATAATGTTATCTTCAATTGATTTCTACT
TAATACCTTATATTACTGGTTTACAATTTACACAATTCCAAACTATAGTTTTGGACATAAAAGAAAAATATGTCTTAACTATTACAATAGAAGTTAACTAAAGATGA
elt-2 promoter
1,500 1,520 1,540 1,560 1,580 1,600

TCTGAGCTACGGCGATACGAGGACGCATTCTCAACGATAATGTTGCCATTTTGTCCTGATAATTTTTTTACTGATTGTTTCAGAACACCCATAGTTTTTCTCTATTA
AGACTCGATGCCGCTATGCTCCTGCGTAAGAGTTGCTATTACAACGGTAAAACAGGACTATTAAAAAAATGACTAACAAAGTCTTGTGGGTATCAAAAAGAGATAAT
elt-2 promoter
1,620 1,640 1,660 1,680 1,700

pMB76 (7778 bp) (from 1713-2568 bp)

AACGTTCATCCTTGACTTCCCCGAGTTTGCTGGCTGAATAGGAAATTTGAAGACAAAAAGGAAAGAATCGGCTCAAACGTCATGCAACTGATAAGGCGACCGTACTT
TTGCAAGTAGGAACTGAAGGGGCTCAAACGACCGACTTATCCTTTAAACTTCTGTTTTTCCTTTCTTAGCCGAGTTTGCAGTACGTTGACTATTCCGCTGGCATGAA
elt-2 promoter
1,720 1,740 1,760 1,780 1,800

CATTTCAAAAGAAGCTCACTTACTGAGCGCAAACATTGAGAAATGAGAGCAAAAGAAGTGGTTTTACAACATGCTAATGTTTTTAGACCTTGTACCCAATAATATTA
GTAAAGTTTTCTTCGAGTGAATGACTCGCGTTTGTAACTCTTTACTCTCGTTTTCTTCACCAAAATGTTGTACGATTACAAAAATCTGGAACATGGGTTATTATAAT
elt-2 promoter
1,820 1,840 1,860 1,880 1,900 1,920

CTGTAGTATACAGTTCGGAGAGCATATGGTTGAAATCTTGAAATACCAATTTATCACTAGTTTGATTGTGTTATCGATGTATAAAGATATATTTTATCATTTTTGAT
GACATCATATGTCAAGCCTCTCGTATACCAACTTTAGAACTTTATGGTTAAATAGTGATCAAACTAACACAATAGCTACATATTTCTATATAAAATAGTAAAAACTA
elt-2 promoter
1,940 1,960 1,980 2,000 2,020

TATTATCTGATATTGTGGGGTGTGAAGTAATATTATGTGCGTGTGTGGCTGATTATCGAAAAAAACTGAAAATTATCAATTTTTCTACAGGTTATCTTTTTTTGTTT
ATAATAGACTATAACACCCCACACTTCATTATAATACACGCACACACCGACTAATAGCTTTTTTTGACTTTTAATAGTTAAAAAGATGTCCAATAGAAAAAAACAAA
elt-2 promoter
2,040 2,060 2,080 2,100 2,120 2,140

TATTTTTCATTATTGTATTCTTCATACTCCTTATCCTGCCCGAATCACAAAAGTTCCAGACAACTTGAAGGTCGCATCACGTTTTGTTTATAAGAATGTTGAACTGG
ATAAAAAGTAATAACATAAGAAGTATGAGGAATAGGACGGGCTTAGTGTTTTCAAGGTCTGTTGAACTTCCAGCGTAGTGCAAAACAAATATTCTTACAACTTGACC
elt-2 promoter
2,160 2,180 2,200 2,220 2,240

TCAGCTTTGGAGACAGAACTGTCCTCCAAATGTTGAATATGTTTCCGTAAAACACACTGTGTAACAAAAAGCGTACCCTTTGATCCGGTATTCTTGAAAAAAAACAT
AGTCGAAACCTCTGTCTTGACAGGAGGTTTACAACTTATACAAAGGCATTTTGTGTGACACATTGTTTTTCGCATGGGAAACTAGGCCATAAGAACTTTTTTTTGTA
elt-2 promoter
2,260 2,280 2,300 2,320 2,340

AGAAGGGCGTACTTTTCATATCTCACAAATATGCGTTTTTTATTTTATAATAATATCATTTATTGATAATTATATGTATTTGTATGTTTTCTTGTCTTTAAAATTAT
TCTTCCCGCATGAAAAGTATAGAGTGTTTATACGCAAAAAATAAAATATTATTATAGTAAATAACTATTAATATACATAAACATACAAAAGAACAGAAATTTTAATA
elt-2 promoter
2,360 2,380 2,400 2,420 2,440 2,460

GGTGTGACATCACAGTTTAAAAATTATATGAAAACTAATGAGTGAAAAGTTAAAAATGTATAGTTTGCCGTATTTTCTCCATTAGTATTGTATTCAAAACTTATTTC
CCACACTGTAGTGTCAAATTTTTAATATACTTTTGATTACTCACTTTTCAATTTTTACATATCAAACGGCATAAAAGAGGTAATCATAACATAAGTTTTGAATAAAG
elt-2 promoter
2,480 2,500 2,520 2,540 2,560

pMB76 (7778 bp) (from 2569-3424 bp)

CAATTGGTGAGCACTATAAACTTTGTAGGGTAATTGAGGTTCAATACTAATTTTTGAATATCTAGCTTTTGACAAATTATTCAAGTTTTGACAATGATGCCTTATCA
GTTAACCACTCGTGATATTTGAAACATCCCATTAACTCCAAGTTATGATTAAAAACTTATAGATCGAAAACTGTTTAATAAGTTCAAAACTGTTACTACGGAATAGT
elt-2 promoter
2,580 2,600 2,620 2,640 2,660

AATTAAAAAAAATTACCATAAATTCTTGAAAGCTTTTGATTTTGTTTCACTCTGTGAAGGATAGTAATTTATCAACATTTTAACTAGATACATCTGTTCTGGCAATA
TTAATTTTTTTTAATGGTATTTAAGAACTTTCGAAAACTAAAACAAAGTGAGACACTTCCTATCATTAAATAGTTGTAAAATTGATCTATGTAGACAAGACCGTTAT
elt-2 promoter
2,680 2,700 2,720 2,740 2,760 2,780

AATTAAAGCAAGACTATTACTATGTGCAGTATTAATAGCTGCATTGCAACTTGTTTGAAAGTTTCAGTGAAAACGCAAAAACTCAATTTAATTTTCAAGTTTGTTTG
TTAATTTCGTTCTGATAATGATACACGTCATAATTATCGACGTAACGTTGAACAAACTTTCAAAGTCACTTTTGCGTTTTTGAGTTAAATTAAAAGTTCAAACAAAC
elt-2 promoter
2,800 2,820 2,840 2,860 2,880

CAAAATTGGATATATGTATCCATCAAAAGTCTGATCGCCTGTTTTATCTACTTATTGTCTCCTCAGTTGATTTTTTTGTAGTTATTATAAACGCTACTTTGAAACCA
GTTTTAACCTATATACATAGGTAGTTTTCAGACTAGCGGACAAAATAGATGAATAACAGAGGAGTCAACTAAAAAAACATCAATAATATTTGCGATGAAACTTTGGT
elt-2 promoter
2,900 2,920 2,940 2,960 2,980

ATATTTTCTTCCTCATTCCACTTTTTTCAATTACCAACTACCGTACATGCAATGATGGGCGGACCCGAGTCCCATGTTTGGCTGGAAGTGGGTGGTTGTCTGCGTAT
TATAAAAGAAGGAGTAAGGTGAAAAAAGTTAATGGTTGATGGCATGTACGTTACTACCCGCCTGGGCTCAGGGTACAAACCGACCTTCACCCACCAACAGACGCATA
elt-2 promoter
3,000 3,020 3,040 3,060 3,080 3,100

ATGAGCGACAGAGGTCGGGGCTGAAACTGATAAGAATAGTCGACACTAACGCCATAATCGCTAGCCAGCCATCATGCACACCGAGCTCGGTGTGCACACCATCTTTC
TACTCGCTGTCTCCAGCCCCGACTTTGACTATTCTTATCAGCTGTGATTGCGGTATTAGCGATCGGTCGGTAGTACGTGTGGCTCGAGCCACACGTGTGGTAGAAAG
elt-2 promoter
3,120 3,140 3,160 3,180 3,200

TTTTCAAACCAATACGCTTTGTGCCTTCATTGACAATTTTCTTTTTGATAAAATCAGCCTATCTATACTTCCCAATCATTTTTAGTCTTATCGTTGAACAGCTATCG
AAAAGTTTGGTTATGCGAAACACGGAAGTAACTGTTAAAAGAAAAACTATTTTAGTCGGATAGATATGAAGGGTTAGTAAAAATCAGAATAGCAACTTGTCGATAGC
elt-2 promoter
3,220 3,240 3,260 3,280 3,300

AGGTGCCACTGTTTTCACTGATATCTTCTAAGTTACTATGGCATTAACATCTTTGTGTTCTTATTTTATGGGTTATTTTAATTAATTTTTGCAGTTAATTTTTGGAA
TCCACGGTGACAAAAGTGACTATAGAAGATTCAATGATACCGTAATTGTAGAAACACAAGAATAAAATACCCAATAAAATTAATTAAAAACGTCAATTAAAAACCTT
elt-2 promoter
3,320 3,340 3,360 3,380 3,400 3,420

pMB76 (7778 bp) (from 3425-4280 bp)

```
TGAGCAAGAAAATGTTAATTGTAATATCTTCGTCTGAAAATTGTCTTCAAATAGTTTTAATTTTAAAGGCAGTATTTAAGAAAATACACTTCTCGAAGCATTTTTGA
ACTCGTTCTTTTACAATTAACATTATAGAAGCAGACTTTTAACAGAAGTTTATCAAAATTAAAATTTCCGTCATAAATTCTTTTATGTGAAGAGCTTCGTAAAAACT
```
elt-2 promoter

3,440 3,460 3,480 3,500 3,520

```
ATTTTTGAATTTTTAAACTGCTTGATGTTTTAGGTGCCACTGTTTTCATTGATAAGTTTTGATGTATAAATGCTTGATTTTCTTGGCATTCTAATAAAATAGAAACT
TAAAAACTTAAAAATTTGACGAACTACAAAATCCACGGTGACAAAAGTAACTATTCAAAACTACATATTTACGAACTAAAAGAACCGTAAGATTATTTTATCTTTGA
```
elt-2 promoter

3,540 3,560 3,580 3,600 3,620

```
AGAAAATAGATTATAGCCCGGGTGCCCTATAGTGAGTCGTATTGGTACCGGTAGAAAAAATGAGTAAAGGAGAAGAACTTTTCACTGGAGTTGTCCCAATTCTTGTT
TCTTTTATCTAATATCGGGCCCACGGGATATCACTCAGCATAACCATGGCCATCTTTTTTACTCATTTCCTCTTCTTGAAAAGTGACCTCAACAGGGTTAAGAACAA
```
GFP

3,640 3,660 3,680 3,700 3,720 3,740

```
GAATTAGATGGTGATGTTAATGGGCACAAATTTTCTGTCAGTGGAGAGGGTGAAGGTGATGCAACATACGGAAAACTTACCCTTAAATTTATTTGCACTACTGGAAA
CTTAATCTACCACTACAATTACCCGTGTTTAAAAGACAGTCACCTCTCCCACTTCCACTACGTTGTATGCCTTTTGAATGGGAATTTAAATAAACGTGATGACCTTT
```
GFP

3,760 3,780 3,800 3,820 3,840

```
ACTACCTGTTCCATGGgtaagtttaaacatatatatactaactaaccctgattatttaaattttcagccaacacttgtcactactttctgttatggtgttcaatgct
TGATGGACAAGGTACCcattcaaatttgtatatatatgattgattgggactaataaatttaaaagtcggttgtgaacagtgatgaaagacaataccacaagttacga
```
GFP GFP

3,860 3,880 3,900 3,920 3,940

```
tctcgagatacccagatcatatgaaacggcatgacttttttcaagagtgccatgcccgaaggttatgtacaggaaagaactatatttttcaaagatgacgggaactac
agagctctatgggtctagtatactttgccgtactgaaaaagttctcacggtacgggcttccaatacatgtcctttcttgatataaaaagtttctactgcccttgatg
```
GFP

3,960 3,980 4,000 4,020 4,040 4,060

```
aagacacgtaagtttaaacagttcggtacgaagttcctatactttctagagaataggaacttctaactaaccatacatatttaaattttcaggtgctgaagtcaagt
ttctgtgcattcaaatttgtcaagccatgcttcaaggatatgaaagatctcttatccttgaagattgattggtatgtataaatttaaaagtccacgacttcagttca
```
FRT site GFP

4,080 4,100 4,120 4,140 4,160

```
ttgaaggtgatacccttgttaatagaatcgagttaaaaggtattgattttaaagaagatggaaacattcttggacacaaattggaatacaactataactcacacaat
aacttccactatgggaacaattatcttagctcaattttccataactaaaatttcttctacctttgtaagaacctgtgtttaaccttatgttgatattgagtgtgtta
```
GFP

4,180 4,200 4,220 4,240 4,260 4,280

pMB76 (7778 bp) (from 4281-5243 bp)

```
gtatacatcatggcagacaaacaaaagaatggaatcaaagttgtaagtttaaacatgattttactaactaactaatctgatttaaattttcagaacttcaaaattag
catatgtagtaccgtctgtttgttttcttaccttagtttcaacattcaaatttgtactaaaatgattgattgattagactaaatttaaaagtcttgaagttttaatc
   GFP                                                                                        GFP
        4,300         4,320         4,340         4,360         4,380

acacaacattgaagatggaagcgttcaactagcagaccattatcaacaaaatactcCAATTGGCGATGGCCCTGTCCTTTTACCAGACAACCATTACCTGTCCACAC
tgtgttgtaacttctaccttcgcaagttgatcgtctggtaatagttgttttatgagGTTAACCGCTACCGGGACAGGAAAATGGTCTGTTGGTAATGGACAGGTGTG
   GFP                                                    GFP
     4,400         4,420         4,440         4,460         4,480

AATCTGCCCTTTCGAAAGATCCCAACGAAAAGAGAGACCACATGGTCCTTCTTGAGTTTGTAACAGCTGCTGGGATTACACATGGCATGGATGAACTATACAAAtct
TTAGACGGGAAAGCTTTCTAGGGTTGCTTTTCTCTCTGGTGTACCAGGAAGAACTCAAACATTGTCGACGACCCTAATGTGTACCGTACCTACTTGATATGTTTaga
   GFP
   4,500         4,520         4,540         4,560         4,580         4,600

ggaggaggatctGAAAATCTTTATTTCCAAGGAtccGAgAAcCTTTAcTTCCAAGGAtctggaggaggatctGGACTTAATGATATTTTCGAAGCTCAgAAgATTGA
cctcctcctagaCTTTTAGAAATAAAGGTTCCTaggCTcTTgGAAATgAAGGTTCCTagacctcctcctagaCCTGAATTACTATAAAAGCTTCGAGTcTTcTAACT
   2 TEV sites                                                    Avi tag
         4,620         4,640         4,660         4,680         4,700

ATGGCATGAAtaaGCTAGCCGGCCATACAAGTAATCCGGATGATCGACGCCaACGTCGTTGAATTTTCAAATTTTAAATACTGAATATTTGTTTTTTTTCCTATTAT
TACCGTACTTattCGATCGGCCGGTATGTTCATTAGGCCTACTAGCTGCGGtTGCAGCAACTTAAAAGTTTAAAATTTATGACTTATAAACAAAAAAAAGGATAATA
                                                               let-858 UTR
      4,720         4,740         4,760         4,780         4,800

TTATTTATTCTCTTTGTGTTTTTTTTCTTGCTTTCTAAAAAATTAATTCAATCCAAATCTAAacatttttttttctctttccgtctcccaattcgtattccgctcct
AATAAATAAGAGAAACACAAAAAAAAGAACGAAAGATTTTTTAATTAAGTTAGGTTTAGATTtgtaaaaaaaaagagaaaggcagagggttaagcataaggcgagga
   let-858 UTR
   4,820         4,840         4,860         4,880         4,900         4,920

ctcatctgaacacaatgtgcaagtttatttatcttctcgctttcatttcattaggacgtgggggaattggtggaagggggaaacacacaaaaggatgatggaaatg
gagtagacttgtgttacacgttcaaataaatagaagagcgaaagtaaagtaatcctgcacccccttaaccaccttccccctttgtgtgttttcctactacctttac
         4,940         4,960         4,980         5,000         5,020

aaataaggacacacaatatgcaacaacattcaattcagaaatatggaggaaggtttaaaagaaaacataaaaatatatagaggaggaaggaaaactagtaaaaaata
tttattcctgtgtgttatacgttgttgtaagttaagtctttatacctccttccaaattttcttttgtatttttatatatctcctccttccttttgatcatttttttat
     5,040         5,060         5,080         5,100         5,120

agcaaagaaattaggcgaacgatgAGAATTGTCCTCGCTTGGGCCCTTTCGTCTCGCGCGTTTCGGTGATGACGGTGAAAACCTCTGACACATGCAGCTCCCGGAGA
tcgtttctttaatccgcttgctacTCTTAACAGGAGCGAACCCGGGAAAGCAGAGCGCGCAAAGCCACTACTGCCACTTTTGGAGACTGTGTACGTCGAGGGCCTCT
  5,140         5,160         5,180         5,200         5,220         5,240
```

pMB76 (7778 bp) (from 5244-6206 bp)

CGGTCACAGCTTGTCTGTAAGCGGATGCCGGGAGCAGACAAGCCCGTCAGGGCGCGTCAGCGGGTGTTGGCGGGTGTCGGGGCTGGCTTAACTATGCGGCATCAGAG
GCCAGTGTCGAACAGACATTCGCCTACGGCCCTCGTCTGTTCGGGCAGTCCCGCGCAGTCGCCCACAACCGCCCACAGCCCCGACCGAATTGATACGCCGTAGTCTC

5,260 5,280 5,300 5,320 5,340

CAGATTGTACTGAGAGTGCACCATATGCGGTGTGAAATACCGCACAGATGCGTAAGGAGAAAATACCGCATCAGGCGGCCTTAAGGGCCTCGTGATACGCCTATTTT
GTCTAACATGACTCTCACGTGGTATACGCCACACTTTATGGCGTGTCTACGCATTCCTCTTTTATGGCGTAGTCCGCCGGAATTCCCGGAGCACTATGCGGATAAAA

5,360 5,380 5,400 5,420 5,440

TATAGGTTAATGTCATGATAATAATGGTTTCTTAGACGTCAGGTGGCACTTTTCGGGGAAATGTGCGCGGAACCCCTATTTGTTTATTTTTCTAAATACATTCAAAT
ATATCCAATTACAGTACTATTATTACCAAAGAATCTGCAGTCCACCGTGAAAAGCCCCTTTACACGCGCCTTGGGGATAAACAAATAAAAAGATTTATGTAAGTTTA

5,460 5,480 5,500 5,520 5,540 5,560

ATGTATCCGCTCATGAGACAATAACCCTGATAAATGCTTCAATAATATTGAAAAAGGAAGAGTATGAGTATTCAACATTTCCGTGTCGCCCTTATTCCCTTTTTTGC
TACATAGGCGAGTACTCTGTTATTGGGACTATTTACGAAGTTATTATAACTTTTTCCTTCTCATACTCATAAGTTGTAAAGGCACAGCGGGAATAAGGGAAAAAACG

5,580 5,600 5,620 5,640 5,660

GGCATTTTGCCTTCCTGTTTTTGCTCACCCAGAAACGCTGGTGAAAGTAAAAGATGCTGAAGATCAGTTGGGTGCACGAGTGGGTTACATCGAACTGGATCTCAACA
CCGTAAAACGGAAGGACAAAAACGAGTGGGTCTTTGCGACCACTTTCATTTTCTACGACTTCTAGTCAACCCACGTGCTCACCCAATGTAGCTTGACCTAGAGTTGT

5,680 5,700 5,720 5,740 5,760

GCGGTAAGATCCTTGAGAGTTTTCGCCCCGAAGAACGTTTTCCAATGATGAGCACTTTTAAAGTTCTGCTATGTGGCGCGGTATTATCCCGTATTGACGCCGGGCAA
CGCCATTCTAGGAACTCTCAAAAGCGGGGCTTCTTGCAAAAGGTTACTACTCGTGAAAATTTCAAGACGATACACCGCGCCATAATAGGGCATAACTGCGGCCCGTT

AmpR

5,780 5,800 5,820 5,840 5,860 5,880

GAGCAACTCGGTCGCCGCATACACTATTCTCAGAATGACTTGGTTGAGTACTCACCAGTCACAGAAAAGCATCTTACGGATGGCATGACAGTAAGAGAATTATGCAG
CTCGTTGAGCCAGCGGCGTATGTGATAAGAGTCTTACTGAACCAACTCATGAGTGGTCAGTGTCTTTTCGTAGAATGCCTACCGTACTGTCATTCTCTTAATACGTC

AmpR

5,900 5,920 5,940 5,960 5,980

TGCTGCCATAACCATGAGTGATAACACTGCGGCCAACTTACTTCTGACAACGATCGGAGGACCGAAGGAGCTAACCGCTTTTTTGCACAACATGGGGGATCATGTAA
ACGACGGTATTGGTACTCACTATTGTGACGCCGGTTGAATGAAGACTGTTGCTAGCCTCCTGGCTTCCTCGATTGGCGAAAAAACGTGTTGTACCCCCTAGTACATT

AmpR

6,000 6,020 6,040 6,060 6,080

CTCGCCTTGATCGTTGGGAACCGGAGCTGAATGAAGCCATACCAAACGACGAGCGTGACACCACGATGCCTGTAGCAATGGCAACAACGTTGCGCAAACTATTAACT
GAGCGGAACTAGCAACCCTTGGCCTCGACTTACTTCGGTATGGTTTGCTGCTCGCACTGTGGTGCTACGGACATCGTTACCGTTGTTGCAACGCGTTTGATAATTGA

AmpR

6,100 6,120 6,140 6,160 6,180 6,200

pMB76 (7778 bp) (from 6207-7062 bp)

GGCGAACTACTTACTCTAGCTTCCCGGCAACAATTAATAGACTGGATGGAGGCGGATAAAGTTGCAGGACCACTTCTGCGCTCGGCCCTTCCGGCTGGCTGGTTTAT
CCGCTTGATGAATGAGATCGAAGGGCCGTTGTTAATTATCTGACCTACCTCCGCCTATTTCAACGTCCTGGTGAAGACGCGAGCCGGGAAGGCCGACCGACCAAATA

AmpR

6,220 6,240 6,260 6,280 6,300

TGCTGATAAATCTGGAGCCGGTGAGCGTGGGTCTCGCGGTATCATTGCAGCACTGGGGCCAGATGGTAAGCCCTCCCGTATCGTAGTTATCTACACGACGGGGAGTC
ACGACTATTTAGACCTCGGCCACTCGCACCCAGAGCGCCATAGTAACGTCGTGACCCCGGTCTACCATTCGGGAGGGCATAGCATCAATAGATGTGCTGCCCCTCAG

AmpR

6,320 6,340 6,360 6,380 6,400 6,420

AGGCAACTATGGATGAACGAAATAGACAGATCGCTGAGATAGGTGCCTCACTGATTAAGCATTGGTAACTGTCAGACCAAGTTTACTCATATATACTTTAGATTGAT
TCCGTTGATACCTACTTGCTTTATCTGTCTAGCGACTCTATCCACGGAGTGACTAATTCGTAACCATTGACAGTCTGGTTCAAATGAGTATATATGAAATCTAACTA

AmpR

6,440 6,460 6,480 6,500 6,520

TTAAAACTTCATTTTTAATTTAAAAGGATCTAGGTGAAGATCCTTTTTGATAATCTCATGACCAAAATCCCTTAACGTGAGTTTTCGTTCCACTGAGCGTCAGACCC
AATTTTGAAGTAAAAATTAAATTTTCCTAGATCCACTTCTAGGAAAAACTATTAGAGTACTGGTTTTAGGGAATTGCACTCAAAAGCAAGGTGACTCGCAGTCTGGG

ColE1 origin

6,540 6,560 6,580 6,600 6,620

CGTAGAAAAGATCAAAGGATCTTCTTGAGATCCTTTTTTTCTGCGCGTAATCTGCTGCTTGCAAACAAAAAAACCACCGCTACCAGCGGTGGTTTGTTTGCCGGATC
GCATCTTTTCTAGTTTCCTAGAAGAACTCTAGGAAAAAAAGACGCGCATTAGACGACGAACGTTTGTTTTTTTGGTGGCGATGGTCGCCACCAAACAAACGGCCTAG

ColE1 origin

6,640 6,660 6,680 6,700 6,720 6,740

AAGAGCTACCAACTCTTTTTCCGAAGGTAACTGGCTTCAGCAGAGCGCAGATACCAAATACTGTCCTTCTAGTGTAGCCGTAGTTAGGCCACCACTTCAAGAACTCT
TTCTCGATGGTTGAGAAAAAGGCTTCCATTGACCGAAGTCGTCTCGCGTCTATGGTTTATGACAGGAAGATCACATCGGCATCAATCCGGTGGTGAAGTTCTTGAGA

ColE1 origin

6,760 6,780 6,800 6,820 6,840

GTAGCACCGCCTACATACCTCGCTCTGCTAATCCTGTTACCAGTGGCTGCTGCCAGTGGCGATAAGTCGTGTCTTACCGGGTTGGACTCAAGACGATAGTTACCGGA
CATCGTGGCGGATGTATGGAGCGAGACGATTAGGACAATGGTCACCGACGACGGTCACCGCTATTCAGCACAGAATGGCCCAACCTGAGTTCTGCTATCAATGGCCT

ColE1 origin

6,860 6,880 6,900 6,920 6,940

TAAGGCGCAGCGGTCGGGCTGAACGGGGGGTTCGTGCACACAGCCCAGCTTGGAGCGAACGACCTACACCGAACTGAGATACCTACAGCGTGAGCATTGAGAAAGCG
ATTCCGCGTCGCCAGCCCGACTTGCCCCCCAAGCACGTGTGTCGGGTCGAACCTCGCTTGCTGGATGTGGCTTGACTCTATGGATGTCGCACTCGTAACTCTTTCGC

ColE1 origin

6,960 6,980 7,000 7,020 7,040 7,060

pMB76 (7778 bp) (from 7063-7778 bp)

CCACGCTTCCCGAAGGGAGAAAGGCGGACAGGTATCCGGTAAGCGGCAGGGTCGGAACAGGAGAGCGCACGAGGGAGCTTCCAGGGGGAAACGCCTGGTATCTTTAT
GGTGCGAAGGGCTTCCCTCTTTCCGCCTGTCCATAGGCCATTCGCCGTCCCAGCCTTGTCCTCTCGCGTGCTCCCTCGAAGGTCCCCCTTTGCGGACCATAGAAATA

ColE1 origin

7,080 7,100 7,120 7,140 7,160

AGTCCTGTCGGGTTTCGCCACCTCTGACTTGAGCGTCGATTTTTGTGATGCTCGTCAGGGGGGCGGAGCCTATGGAAAAACGCCAGCAACGCGGCCTTTTTACGGTT
TCAGGACAGCCCAAAGCGGTGGAGACTGAACTCGCAGCTAAAAACACTACGAGCAGTCCCCCCGCCTCGGATACCTTTTTGCGGTCGTTGCGCCGGAAAAATGCCAA

ColE1 origin

7,180 7,200 7,220 7,240 7,260

CCTGGCCTTTTGCTGGCCTTTTGCTCACATGTTCTTTCCTGCGTTATCCCCTGATTCTGTGGATAACCGTATTACCGCCTTTGAGTGAGCTGATACCGCTCGCCGCA
GGACCGGAAAACGACCGGAAAACGAGTGTACAAGAAAGGACGCAATAGGGGACTAAGACACCTATTGGCATAATGGCGGAAACTCACTCGACTATGGCGAGCGGCGT

7,280 7,300 7,320 7,340 7,360 7,380

GCCGAACGACCGAGCGCAGCGAGTCAGTGAGCGAGGAAGCGGAAGAGCGCCCAATACGCAAACCGCCTCTCCCCGCGCGTTGGCCGATTCATTAATGCAGCTGGCAC
CGGCTTGCTGGCTCGCGTCGCTCAGTCACTCGCTCCTTCGCCTTCTCGCGGGTTATGCGTTTGGCGGAGAGGGGCGCGCAACCGGCTAAGTAATTACGTCGACCGTG

7,400 7,420 7,440 7,460 7,480

GACAGGTTTCCCGACTGGAAAGCGGGCAGTGAGCGCAACGCAATTAATGTGAGTTAGCTCACTCATTAGGCACCCCAGGCTTTACACTTTATGCTTCCGGCTCGTAT
CTGTCCAAAGGGCTGACCTTTCGCCCGTCACTCGCGTTGCGTTAATTACACTCAATCGAGTGAGTAATCCGTGGGGTCCGAAATGTGAAATACGAAGGCCGAGCATA

7,500 7,520 7,540 7,560 7,580

GTTGTGTGGAATTGTGAGCGGATAACAATTTCACACAGGAAACAGCTATGACCATGATTACGCCAAGCTgtaagtttaaacatgatcttactaactaactattctca
CAACACACCTTAACACTCGCCTATTGTTAAAGTGTGTCCTTTGTCGATACTGGTACTAATGCGGTTCGAcattcaaatttgtactagaatgattgattgataagagt

LacO M13-rev

7,600 7,620 7,640 7,660 7,680 7,700

tttaaattttcagAGCTTAAAAATGGCTGAAATCACTCACAACGATGGATACGCTAACAACTTGGAAATGAAAT
aaatttaaaagtcTCGAATTTTTACCGACTTTAGTGAGTGTTGCTACCTATGCGATTGTTGAACCTTTACTTTA

7,710 7,720 7,730 7,740 7,750 7,760 7,770

pMB77 (5784 bp)

```
AAGCTTGGGCTGCAGCAGGTCGACTCCAcGTAATTtcacaacatccgggaaaagaagtgaaaaaaggactcgagcaactgtacaagaaaattgagaagaacctggtt
TTCGAACCCGACGTCGTCCAGCTGAGGTgCATTAAagtgttgtaggcccttttcttcacttttttcctgagctcgttgacatgttcttttaactcttcttggaccaa
pwrt-2
20 40 60 80 100

gccaattcatcacttttgcaagttgtatggagagatatgcaagagcaatttgtcaagcaaattgcggaatacaacaaactgattctcacttgttacccaggctcgaa
cggttaagtagtgaaaacgttcaacatacctctctatacgttctcgttaaacagttcgtttaacgccttatgttgtttgactaagagtgaacaatgggtccgagctt
pwrt-2
120 140 160 180 200

gattgagctcgaagtttccactgacactgtacttcagttcttctcagaaattgctcaacaacactaatattacgacgaataattttattgaattttgttgttttaat
ctaactcgagcttcaaaggtgactgtgacatgaagtcaagaagagtctttaacgagttgttgtgattataatgctgcttattaaaataacttaaaacaacaaaatta
pwrt-2
220 240 260 280 300 320

tcatttaaaatttagttttaatttatcagaactctaatacttactgaaccatgctttgtaatatatttatttatctattctgtaataggtgaacttcgttacattac
agtaaattttaaatcaaaattaaatagtcttgagattatgaatgacttggtacgaaacattatataaataaatagataagacattatccacttgaagcaatgtaatg
pwrt-2
340 360 380 400 420

tgatgtctgtaatataaatttacgggtgttccccattcgaaaatagtagtaatgcttgctttttgaaaaataaatgtcttgcttggtgacttttatttcacgtatt
actacagacattatatttaaatgcccacaaggggtaagcttttatcatcattacgaacgaaaaactttttatttacagaacgaaccactgaaaataaagtgcataa
pwrt-2
440 460 480 500 520

ttcaaatgctcacaaaaatttcacggaactgacttcttggttacacatttgtagaaattttgactgaatgtttgcattgaactggtcaacttaagaatctgacatgc
aagtttacgagtgtttttaaagtgccttgactgaagaaccaatgtgtaaacatctttaaaactgacttacaaacgtaacttgaccagttgaattcttagactgtacg
pwrt-2
540 560 580 600 620 640

aaatggtgtgttttctgaagaacacactttggcaacttttaataaaaaggggtgtaacacgatttaaatctaataaaaacaacttctataatgttattccgtttat
tttaccacacaaaagacttcttgtgtgaaaccgttgaaaattatttttccccacattgtgctaaatttagattatttttgttgaagatattacaataaggcaaata
pwrt-2
660 680 700 720 740

atctcactttatgttttacattttactcttatatacatttttgactcatttatacagggtggtccataattatggaccaccctgtacttatatcttatttaaaact
tagagtgaaatacaaaatgtaaaatgagaatatatgtaaaaactgagtaaatatgtcccaccaggtattaatacctggtgggacatgaatatagaataaattttga
pwrt-2
760 780 800 820 840
```

pMB77 (5784 bp) (from 857-1712 bp)

```
atttcatgtaagaattgttttaacagcgcatttttttcatcactcgatttaaaggtaaaattgataggccgcaatctattttggaaaacttcagaacaaaatttaa
taaagtacattcttaacaaaattgtcgcgtaaaaaaagtagtgagctaaatttccattttaactatccggcgttagataaaaccttttgaagtcttgttttaaatt
```

pwrt-2

860 880 900 920 940 960

```
cattttaagaggtaactgtgaaggtttataagagaattataaaccgatctaaccactaaacatggtaacgtagaaaaagtaaaattagggagggggggatgtttatt
gtaaaattctccattgacacttccaaatattctcttaatatttggctagattggtgatttgtaccattgcatctttttcattttaatccctccccccctacaaataa
```

pwrt-2

980 1,000 1,020 1,040 1,060

```
tattcttggttttcaattcttatcagactctgtaaagtaaaatgaacataacttgttcgaatagtgtaggaatttcaaacaagctacaaatctagctccacatacgc
ataagaaccaaaagttaagaatagtctgagacatttcattttacttgtattgaacaagcttatcacatccttaaagtttgttcgatgtttagatcgaggtgtatgcg
```

pwrt-2

1,080 1,100 1,120 1,140 1,160

```
gtggaatagccggtcataaatcgtcttccgttaaccgcatggctagatccacaccgattccatttttcttgaataaaacaatagcttgatgaaaattgggtagccac
caccttatcggccagtatttagcagaaggcaattggcgtaccgatctaggtgtggctaaggtaaaaagaacttattttgttatcgaactacttttaacccatcggtg
```

pwrt-2

1,180 1,200 1,220 1,240 1,260 1,280

```
cgaaaaaactgattcgcgatccggattattggtgacaaacatacggcaagtgaaagtgttgcactgatagaaaccgatacgagaatatgccttttgggccccccccc
gctttttttgactaagcgctaggcctaataaccactgtttgtatgccgttcactttcacaacgtgactatctttggctatgctcttatacggaaaacccggggggggg
```

pwrt-2

1,300 1,320 1,340 1,360 1,380

```
ccccccccctacggcttcactcccggtgttgtagatctgactttcacttgccttatctcgaggaatctggagaggagaaggagggacaaccgatccgcgcgcgcgta
gggggggggatgccgaagtgagggccacaacatctagactgaaagtgaacggaatagagctccttagacctctcctcttcctccctgttggctaggcgcgcgcgcat
```

pwrt-2

1,400 1,420 1,440 1,460 1,480

```
acaatacgggagatatagaggaaaagcagcaaaaagacattttgatgaccttattttcttgtcgttgtcgagacggccattgtcccttccacgcccagcttttcatt
tgttatgccctctatatctccttttcgtcgtttttctgtaaaactactggaataaaagaacagcaacagctctgccggtaacagggaaggtgcgggtcgaaaagtaa
```

pwrt-2

1,500 1,520 1,540 1,560 1,580 1,600

```
gtagttcaaatcgcttctcattgacaatCAACCTGCCAATTGTTTCTCGGgatccCCCGGGTGCCCTATAGTGAGTCGTATTGGTACCGGTAGAAAAAATGAGTAAA
catcaagtttagcgaagagtaactgttaGTTGGACGGTTAACAAAGAGCCcctagGGGCCCACGGGATATCACTCAGCATAACCATGGCCATCTTTTTTACTCATTT
```

pwrt-2 GFP

1,620 1,640 1,660 1,680 1,700

pMB77 (5784 bp) (from 1713-2568 bp)

GGAGAAGAACTTTTCACTGGAGTTGTCCCAATTCTTGTTGAATTAGATGGTGATGTTAATGGGCACAAATTTTCTGTCAGTGGAGAGGGTGAAGGTGATGCAACATA
CCTCTTCTTGAAAAGTGACCTCAACAGGGTTAAGAACAACTTAATCTACCACTACAATTACCCGTGTTTAAAAGACAGTCACCTCTCCCACTTCCACTACGTTGTAT
GFP
1,720 1,740 1,760 1,780 1,800

CGGAAAACTTACCCTTAAATTTATTTGCACTACTGGAAAACTACCTGTTCCATGGgtaagtttaaacatatatatactaactaaccctgattatttaaattttcagc
GCCTTTTGAATGGGAATTTAAATAAACGTGATGACCTTTTGATGGACAAGGTACCcattcaaatttgtatatatatgattgattgggactaataaatttaaaagtcg
GFP
1,820 1,840 1,860 1,880 1,900 1,920

caacacttgtcactactttctgttatggtgttcaatgcttctcgagatacccagatcatatgaaacggcatgactttttcaagagtgccatgcccgaaggttatgta
gttgtgaacagtgatgaaagacaataccacaagttacgaagagctctatgggtctagtatactttgccgtactgaaaaagttctcacggtacgggcttccaatacat
GFP
1,940 1,960 1,980 2,000 2,020

caggaaagaactatatttttcaaagatgacgggaactacaagacacgtaagtttaaacagttcggtacgaagttcctatactttctagagaataggaacttctaact
gtcctttcttgatataaaaagtttctactgcccttgatgttctgtgcattcaaatttgtcaagccatgcttcaaggatatgaaagatctcttatccttgaagattga
GFP FRT site
2,040 2,060 2,080 2,100 2,120 2,140

aaccatacatatttaaattttcaggtgctgaagtcaagtttgaaggtgatacccttgttaatagaatcgagttaaaaggtattgattttaaagaagatggaaacatt
ttggtatgtataaatttaaaagtccacgacttcagttcaaacttccactatgggaacaattatcttagctcaattttccataactaaaatttcttctacctttgtaa
GFP
2,160 2,180 2,200 2,220 2,240

cttggacacaaattggaatacaactataactcacacaatgtatacatcatggcagacaaacaaaagaatggaatcaaagttgtaagtttaaacatgattttactaac
gaacctgtgtttaaccttatgttgatattgagtgtgttacatatgtagtaccgtctgtttgttttcttaccttagtttcaacattcaaatttgtactaaaatgattg
GFP
2,260 2,280 2,300 2,320 2,340

taactaatctgatttaaattttcagaacttcaaaattagacacaacattgaagatggaagcgttcaactagcagaccattatcaacaaaatactcCAATTGGCGATG
attgattagactaaatttaaaagtcttgaagttttaatctgtgttgtaacttctaccttcgcaagttgatcgtctggtaatagttgttttatgagGTTAACCGCTAC
GFP GFP
2,360 2,380 2,400 2,420 2,440 2,460

GCCCTGTCCTTTTACCAGACAACCATTACCTGTCCACACAATCTGCCCTTTCGAAAGATCCCAACGAAAAGAGAGACCACATGGTCCTTCTTGAGTTTGTAACAGCT
CGGGACAGGAAAATGGTCTGTTGGTAATGGACAGGTGTGTTAGACGGGAAAGCTTTCTAGGGTTGCTTTTCTCTCTGGTGTACCAGGAAGAACTCAAACATTGTCGA
GFP
2,480 2,500 2,520 2,540 2,560

pMB77 (5784 bp) (from 2569-3638 bp)

GCTGGGATTACACATGGCATGGATGAACTATACAAAtctggaggaggatctGAAAATCTTTATTTCCAAGGAtccGAgAAcCTTTAcTTCCAAGGAtctggaggagg
CGACCCTAATGTGTACCGTACCTACTTGATATGTTTagacctcctcctagaCTTTTAGAAATAAAGGTTCCTaggCTcTTgGAAATgAAGGTTCCTagacctcctcc

GFP 2 TEV sites

2,580 2,600 2,620 2,640 2,660

atctGGACTTAATGATATTTTCGAAGCTCAgAAgATTGAATGGCATGAAtaaGCTAGCCGGCCATACAAGTAATCCGGATGATCGACGCCaACGTCGTTGAATTTTC
tagaCCTGAATTACTATAAAAGCTTCGAGTcTTcTAACTTACCGTACTTattCGATCGGCCGGTATGTTCATTAGGCCTACTAGCTGCGGtTGCAGCAACTTAAAAG

Avi tag

2,680 2,700 2,720 2,740 2,760 2,780

AAATTTTAAATACTGAATATTTGTTTTTTTTCCTATTATTTATTTATTCTCTTTGTGTTTTTTTTCTTGCTTTCTAAAAAATTAATTCAATCCAAATCTAAacattt
TTTAAAATTTATGACTTATAAACAAAAAAAAGGATAATAAATAAATAAGAGAAACACAAAAAAAAGAACGAAAGATTTTTTAATTAAGTTAGGTTTAGATTtgtaaa

let-858 UTR

2,800 2,820 2,840 2,860 2,880

tttttctctttccgtctcccaattcgtattccgctcctctcatctgaacacaatgtgcaagtttatttatcttctcgctttcatttcattaggacgtgggggaat
aaaaagagaaaggcagagggttaagcataaggcgaggagagtagacttgtgttacacgttcaaataaatagaagagcgaaagtaaagtaatcctgcacccccctta

2,900 2,920 2,940 2,960 2,980

tggtggaagggggaaacacacaaaaggatgatggaaatgaaataaggacacacaatatgcaacaacattcaattcagaaatatggaggaaggtttaaaagaaaacat
accaccttccccctttgtgtgttttcctactacctttactttattcctgtgtgttatacgttgttgtaagttaagtctttatacctccttccaaattttcttttgta

3,000 3,020 3,040 3,060 3,080 3,100

aaaaatatatagaggaggaaggaaaactagtaaaaaataagcaaagaaattaggcgaacgatgAGAATTGTCCTCGCTTGGGCCCTTTCGTCTCGCGCGTTTCGGTG
tttttatatatctcctccttccttttgatcattttttattcgtttctttaatccgcttgctacTCTTAACAGGAGCGAACCCGGGAAAGCAGAGCGCGCAAAGCCAC

3,120 3,140 3,160 3,180 3,200

ATGACGGTGAAAACCTCTGACACATGCAGCTCCCGGAGACGGTCACAGCTTGTCTGTAAGCGGATGCCGGGAGCAGACAAGCCCGTCAGGGCGCGTCAGCGGGTGTT
TACTGCCACTTTTGGAGACTGTGTACGTCGAGGGCCTCTGCCAGTGTCGAACAGACATTCGCCTACGGCCCTCGTCTGTTCGGGCAGTCCCGCGCAGTCGCCCACAA

3,220 3,240 3,260 3,280 3,300

GGCGGGTGTCGGGGCTGGCTTAACTATGCGGCATCAGAGCAGATTGTACTGAGAGTGCACCATATGCGGTGTGAAATACCGCACAGATGCGTAAGGAGAAAATACCG
CCGCCCACAGCCCCGACCGAATTGATACGCCGTAGTCTCGTCTAACATGACTCTCACGTGGTATACGCCACACTTTATGGCGTGTCTACGCATTCCTCTTTTATGGC

3,320 3,340 3,360 3,380 3,400 3,420

CATCAGGCGGCCTTAAGGGCCTCGTGATACGCCTATTTTTATAGGTTAATGTCATGATAATAATGGTTTCTTAGACGTCAGGTGGCACTTTTCGGGGAAATGTGCGC
GTAGTCCGCCGGAATTCCCGGAGCACTATGCGGATAAAAATATCCAATTACAGTACTATTATTACCAAAGAATCTGCAGTCCACCGTGAAAAGCCCCTTTACACGCG

3,440 3,460 3,480 3,500 3,520

GGAACCCCTATTTGTTTATTTTTCTAAATACATTCAAATATGTATCCGCTCATGAGACAATAACCCTGATAAATGCTTCAATAATATTGAAAAAGGAAGAGTATGAG
CCTTGGGGATAAACAAATAAAAAGATTTATGTAAGTTTATACATAGGCGAGTACTCTGTTATTGGGACTATTTACGAAGTTATTATAACTTTTTCCTTCTCATACTC

3,540 3,560 3,580 3,600 3,620

pMB77 (5784 bp) (from 3639-4601 bp)

TATTCAACATTTCCGTGTCGCCCTTATTCCCTTTTTTGCGGCATTTTGCCTTCCTGTTTTTGCTCACCCAGAAACGCTGGTGAAAGTAAAAGATGCTGAAGATCAGT
ATAAGTTGTAAAGGCACAGCGGGAATAAGGGAAAAAACGCCGTAAAACGGAAGGACAAAAACGAGTGGGTCTTTGCGACCACTTTCATTTTCTACGACTTCTAGTCA

3,640 3,660 3,680 3,700 3,720 3,740

TGGGTGCACGAGTGGGTTACATCGAACTGGATCTCAACAGCGGTAAGATCCTTGAGAGTTTTCGCCCCGAAGAACGTTTTCCAATGATGAGCACTTTTAAAGTTCTG
ACCCACGTGCTCACCCAATGTAGCTTGACCTAGAGTTGTCGCCATTCTAGGAACTCTCAAAAGCGGGGCTTCTTGCAAAAGGTTACTACTCGTGAAAATTTCAAGAC

AmpR

3,760 3,780 3,800 3,820 3,840

CTATGTGGCGCGGTATTATCCCGTATTGACGCCGGGCAAGAGCAACTCGGTCGCCGCATACACTATTCTCAGAATGACTTGGTTGAGTACTCACCAGTCACAGAAAA
GATACACCGCGCCATAATAGGGCATAACTGCGGCCCGTTCTCGTTGAGCCAGCGGCGTATGTGATAAGAGTCTTACTGAACCAACTCATGAGTGGTCAGTGTCTTTT

AmpR

3,860 3,880 3,900 3,920 3,940

GCATCTTACGGATGGCATGACAGTAAGAGAATTATGCAGTGCTGCCATAACCATGAGTGATAACACTGCGGCCAACTTACTTCTGACAACGATCGGAGGACCGAAGG
CGTAGAATGCCTACCGTACTGTCATTCTCTTAATACGTCACGACGGTATTGGTACTCACTATTGTGACGCCGGTTGAATGAAGACTGTTGCTAGCCTCCTGGCTTCC

AmpR

3,960 3,980 4,000 4,020 4,040 4,060

AGCTAACCGCTTTTTTGCACAACATGGGGGATCATGTAACTCGCCTTGATCGTTGGGAACCGGAGCTGAATGAAGCCATACCAAACGACGAGCGTGACACCACGATG
TCGATTGGCGAAAAAACGTGTTGTACCCCCTAGTACATTGAGCGGAACTAGCAACCCTTGGCCTCGACTTACTTCGGTATGGTTTGCTGCTCGCACTGTGGTGCTAC

AmpR

4,080 4,100 4,120 4,140 4,160

CCTGTAGCAATGGCAACAACGTTGCGCAAACTATTAACTGGCGAACTACTTACTCTAGCTTCCCGGCAACAATTAATAGACTGGATGGAGGCGGATAAAGTTGCAGG
GGACATCGTTACCGTTGTTGCAACGCGTTTGATAATTGACCGCTTGATGAATGAGATCGAAGGGCCGTTGTTAATTATCTGACCTACCTCCGCCTATTTCAACGTCC

AmpR

4,180 4,200 4,220 4,240 4,260 4,280

ACCACTTCTGCGCTCGGCCCTTCCGGCTGGCTGGTTTATTGCTGATAAATCTGGAGCCGGTGAGCGTGGGTCTCGCGGTATCATTGCAGCACTGGGGCCAGATGGTA
TGGTGAAGACGCGAGCCGGGAAGGCCGACCGACCAAATAACGACTATTTAGACCTCGGCCACTCGCACCCAGAGCGCCATAGTAACGTCGTGACCCCGGTCTACCAT

AmpR

4,300 4,320 4,340 4,360 4,380

AGCCCTCCCGTATCGTAGTTATCTACACGACGGGGAGTCAGGCAACTATGGATGAACGAAATAGACAGATCGCTGAGATAGGTGCCTCACTGATTAAGCATTGGTAA
TCGGGAGGGCATAGCATCAATAGATGTGCTGCCCCTCAGTCCGTTGATACCTACTTGCTTTATCTGTCTAGCGACTCTATCCACGGAGTGACTAATTCGTAACCATT

AmpR

4,400 4,420 4,440 4,460 4,480

CTGTCAGACCAAGTTTACTCATATATACTTTAGATTGATTTAAAACTTCATTTTTAATTTAAAAGGATCTAGGTGAAGATCCTTTTTGATAATCTCATGACCAAAAT
GACAGTCTGGTTCAAATGAGTATATATGAAATCTAACTAAATTTTGAAGTAAAAATTAAATTTTCCTAGATCCACTTCTAGGAAAAACTATTAGAGTACTGGTTTTA

4,500 4,520 4,540 4,560 4,580 4,600

pMB77 (5784 bp) (from 4602-5564 bp)

CCCTTAACGTGAGTTTTCGTTCCACTGAGCGTCAGACCCCGTAGAAAAGATCAAAGGATCTTCTTGAGATCCTTTTTTTCTGCGCGTAATCTGCTGCTTGCAAACAA
GGGAATTGCACTCAAAAGCAAGGTGACTCGCAGTCTGGGGCATCTTTTCTAGTTTCCTAGAAGAACTCTAGGAAAAAAAGACGCGCATTAGACGACGAACGTTTGTT

ColE1 origin

4,620 4,640 4,660 4,680 4,700

AAAAACCACCGCTACCAGCGGTGGTTTGTTTGCCGGATCAAGAGCTACCAACTCTTTTTCCGAAGGTAACTGGCTTCAGCAGAGCGCAGATACCAAATACTGTCCTT
TTTTTGGTGGCGATGGTCGCCACCAAACAAACGGCCTAGTTCTCGATGGTTGAGAAAAAGGCTTCCATTGACCGAAGTCGTCTCGCGTCTATGGTTTATGACAGGAA

ColE1 origin

4,720 4,740 4,760 4,780 4,800

CTAGTGTAGCCGTAGTTAGGCCACCACTTCAAGAACTCTGTAGCACCGCCTACATACCTCGCTCTGCTAATCCTGTTACCAGTGGCTGCTGCCAGTGGCGATAAGTC
GATCACATCGGCATCAATCCGGTGGTGAAGTTCTTGAGACATCGTGGCGGATGTATGGAGCGAGACGATTAGGACAATGGTCACCGACGACGGTCACCGCTATTCAG

ColE1 origin

4,820 4,840 4,860 4,880 4,900 4,920

GTGTCTTACCGGGTTGGACTCAAGACGATAGTTACCGGATAAGGCGCAGCGGTCGGGCTGAACGGGGGGTTCGTGCACACAGCCCAGCTTGGAGCGAACGACCTACA
CACAGAATGGCCCAACCTGAGTTCTGCTATCAATGGCCTATTCCGCGTCGCCAGCCCGACTTGCCCCCCAAGCACGTGTGTCGGGTCGAACCTCGCTTGCTGGATGT

ColE1 origin

4,940 4,960 4,980 5,000 5,020

CCGAACTGAGATACCTACAGCGTGAGCATTGAGAAAGCGCCACGCTTCCCGAAGGGAGAAAGGCGGACAGGTATCCGGTAAGCGGCAGGGTCGGAACAGGAGAGCGC
GGCTTGACTCTATGGATGTCGCACTCGTAACTCTTTCGCGGTGCGAAGGGCTTCCCTCTTTCCGCCTGTCCATAGGCCATTCGCCGTCCCAGCCTTGTCCTCTCGCG

ColE1 origin

5,040 5,060 5,080 5,100 5,120

ACGAGGGAGCTTCCAGGGGGAAACGCCTGGTATCTTTATAGTCCTGTCGGGTTTCGCCACCTCTGACTTGAGCGTCGATTTTTGTGATGCTCGTCAGGGGGGCGGAG
TGCTCCCTCGAAGGTCCCCCTTTGCGGACCATAGAAATATCAGGACAGCCCAAAGCGGTGGAGACTGAACTCGCAGCTAAAAACACTACGAGCAGTCCCCCCGCCTC

ColE1 origin

5,140 5,160 5,180 5,200 5,220 5,240

CCTATGGAAAAACGCCAGCAACGCGGCCTTTTTACGGTTCCTGGCCTTTTGCTGGCCTTTTGCTCACATGTTCTTTCCTGCGTTATCCCCTGATTCTGTGGATAACC
GGATACCTTTTTGCGGTCGTTGCGCCGGAAAAATGCCAAGGACCGGAAAACGACCGGAAAACGAGTGTACAAGAAAGGACGCAATAGGGGACTAAGACACCTATTGG

ColE1 origin

5,260 5,280 5,300 5,320 5,340

GTATTACCGCCTTTGAGTGAGCTGATACCGCTCGCCGCAGCCGAACGACCGAGCGCAGCGAGTCAGTGAGCGAGGAAGCGGAAGAGCGCCCAATACGCAAACCGCCT
CATAATGGCGGAAACTCACTCGACTATGGCGAGCGGCGTCGGCTTGCTGGCTCGCGTCGCTCAGTCACTCGCTCCTTCGCCTTCTCGCGGGTTATGCGTTTGGCGGA

5,360 5,380 5,400 5,420 5,440

CTCCCCGCGCGTTGGCCGATTCATTAATGCAGCTGGCACGACAGGTTTCCCGACTGGAAAGCGGGCAGTGAGCGCAACGCAATTAATGTGAGTTAGCTCACTCATTA
GAGGGGCGCGCAACCGGCTAAGTAATTACGTCGACCGTGCTGTCCAAAGGGCTGACCTTTCGCCCGTCACTCGCGTTGCGTTAATTACACTCAATCGAGTGAGTAAT

5,460 5,480 5,500 5,520 5,540 5,560

pMB77 (5784 bp) (from 5565-5784 bp)

GGCACCCCAGGCTTTACACTTTATGCTTCCGGCTCGTATGTTGTGTGGAATTGTGAGCGGATAACAATTTCACACAGGAAACAGCTATGACCATGATTACGCCAAGC
CCGTGGGGTCCGAAATGTGAAATACGAAGGCCGAGCATACAACACACCTTAACACTCGCCTATTGTTAAAGTGTGTCCTTTGTCGATACTGGTACTAATGCGGTTCG

LacO M13-rev

5,580 5,600 5,620 5,640 5,660

TgtaagtttaaacatgatcttactaactaactattctcatttaaattttcagAGCTTAAAAATGGCTGAAATCACTCACAACGATGGATACGCTAACAACTTGGAAA
AcattcaaatttgtactagaatgattgattgataagagtaaatttaaaagtcTCGAATTTTTACCGACTTTAGTGAGTGTTGCTACCTATGCGATTGTTGAACCTTT

5,680 5,700 5,720 5,740 5,760

TGAAAT
ACTTTA

5,780
